# Lessons Learned From the Use of the Most Significant Change Technique for Adaptive Management of Complex Health Interventions

**DOI:** 10.9745/GHSP-D-21-00624

**Published:** 2022-02-28

**Authors:** Saori Ohkubo, Lisa Mwaikambo, Ruwaida M. Salem, Lekan Ajijola, Paul Nyachae, Mukesh Kumar Sharma

**Affiliations:** a The Challenge Initiative, Johns Hopkins Center for Communication Programs, Baltimore, MD, USA.; b The Challenge Initiative, Nigeria Hub, Johns Hopkins Center for Communication Programs, Abuja, Nigeria.; c The Challenge Initiative, East Africa Hub, Jhpiego, Nairobi, Kenya.; d The Challenge Initiative for Healthy Cities, India Hub, Population Services International, Delhi, India.

## Abstract

**Introduction:**

The Most Significant Change (MSC) technique is a complex-aware monitoring and evaluation tool, widely recognized for various adaptive management purposes. The documentation of practical examples using the MSC technique for an ongoing monitoring purpose is limited. We aim to fill the current gap by documenting and sharing the experience and lessons learned of The Challenge Initiative (TCI), which is scaling up evidence-based family planning (FP) and adolescent and youth sexual and reproductive health (AYSRH) interventions in 11 countries in Asia and sub-Saharan Africa.

**Methods:**

The qualitative assessment took place in early 2021 to document TCI’s use and adaptation of MSC and determine its added value in adaptive management, routine monitoring, and cross-learning efforts. Focus group discussions and key informant interviews were conducted virtually with staff members involved in collecting and selecting MSC stories.

**Results:**

TCI has had a positive experience with using MSC to facilitate adaptive management in multiple countries. The use of MSC has created learning opportunities that have helped diffuse evidence-based FP and AYSRH interventions both within and across countries. The responsive feedback step in the MSC process was viewed as indispensable to learning and collaboration. There are several necessary inputs to successful use of the method, including buy-in about the benefits, training on good interviewing techniques and qualitative research, and dedicated staff to manage the process.

**Conclusion:**

Our assessment results suggest that the MSC technique is an effective qualitative data collection tool to strengthen routine monitoring and adaptive management efforts that allows for flexibility in how project stakeholders implement the process. The MSC technique could be an important tool for global health practitioners, policy makers, and researchers working on complex interventions because they continually need to understand stakeholders’ needs and priorities, learn from lessons and evidence-based practices, and be agile about addressing potential challenges.

## Abstract (precis)

The Most Significant Change technique used in monitoring and evaluation has facilitated learning about the project scale-up and adaptive management of evidence-based family planning interventions across diverse project stakeholders in 11 countries in Asia and sub-Saharan Africa.

## Teaser

**Key Findings:**

- Project staff members in East Africa, Francophone West Africa, India, and Nigeria reported that using the Most Significant Change (MSC) technique had resulted in learning within and across countries to diffuse evidence-based interventions, as well as take concrete adaptive management actions.
- Collecting significant change stories allows various voices and perspectives across the health system, from the community level to policy makers, to be captured about the impacts of programs, thus enabling prompt corrective actions.
- Selection meeting discussions and responsive feedback about the significant change stories among various project stakeholders are critical for learning and collaboration.

**Key Implications:**

- The MSC technique could be an important learning and adaptive management tool for global health practitioners, policy makers, and researchers working on complex interventions.
- Although MSC makes use of a systematic and standard process, it is flexible in how the process is institutionalized to meet the needs and unique context of the implementation team.

## INTRODUCTION

Over the last several years, there has been a growing interest among practitioners and researchers in identifying innovative approaches to monitoring and evaluation (M&E) of complex health interventions to facilitate learning and adaptation of evidence-based practices. In 2019, *BMJ Global Health* published a supplement titled, “Complex Health Interventions in Complex Systems: Concepts and Methods for Evidence-Informed Health Decisions.” This effort was led by the World Health Organization to present the broader conceptualization of complexity, add value to global health interventions characterized by complexity, and call for close collaboration among various stakeholders to benefit from a complexity perspective in the evaluation of intervention effectiveness.^1^

The Most Significant Change (MSC) technique is one such complex-aware M&E tool. It was first developed as a means of monitoring changes in a specific development project in the 1990s and introduced to the broader international development sector in 2005.^2^^,^^3^ Instead of using precise indicators, MSC uses broad domains of change and asks project stakeholders to share stories of significant change related to those domains. Stakeholder panels then discuss the reported value of the reported changes, select the most significant stories, and provide feedback about their selections and rationale. The stories are intended to be collected at regular intervals to track changes as they are emerging, rather than waiting until the end of a project cycle when it may be too late to make improvements to the project. As such, it helps to support adaptive management.

Instead of using precise indicators, MSC uses broad domains of change and asks project stakeholders to share stories of significant change related to those domains.

The users of MSC generally report positive experiences with the method, particularly noting unique roles and contributions of the narrative approach. Tonkin et al.^4^ noted that the strength of the MSC technique lies in characterizing the change in complex health interventions and confirmed its plasticity, feasibility, and acceptability across different sociopolitical, cultural, and environmental contexts. Consequently, the MSC approach would work well to help evaluate the scale-up of evidence-based interventions across multiple countries.^5^ The MSC technique is also easy for participants to understand and could help assess the performance of a program as a whole.^6^

MSC has been widely recognized for various adaptive management purposes, for example, to track changes continuously; facilitate learning, responsive feedback, and decision making; and capture program contributions to intended and unintended outcomes.^7^^–^^11^ However, to date, the evidence of the effectiveness of the MSC method is still lacking in the monitoring and adaptive management field. Most well-documented experiences and lessons learned in MSC application come from the final or summative evaluation activities. Like other participatory qualitative research methods, the MSC technique can be time-consuming and may require careful facilitation by skilled researchers to correctly use the method.^6^ Program managers do not necessarily consider the MSC method a go-to tool because of the lack of understanding of its utility. They may be reluctant to spend adequate time and resources to learn how to use and implement it to enhance their monitoring effort. Therefore, the documentation of practical examples using the MSC technique for an ongoing monitoring purpose is limited.

This assessment aims to fill the current gap in the field of MSC application by documenting and sharing the experience and lessons learned of The Challenge Initiative (TCI), which is scaling up evidence-based family planning (FP) and adolescent and youth sexual and reproductive health (AYSRH) interventions in 11 countries in Asia and sub-Saharan Africa. The project has institutionalized the MSC technique as a routine data collection activity to strengthen its monitoring and adaptive management efforts. We will discuss the values of the method, as perceived by program managers who use MSC as they work directly with stakeholders, including government officials and community leaders, as well as the elements of successful integration and localization of the method for adaptive management in a complex global health program.

## TCI PROJECT DESCRIPTION

Since 2017, TCI has aimed to empower local city governments across sub-Saharan Africa and Asia to rapidly scale up evidence-based FP and AYSRH interventions that meet the needs of their communities. TCI currently works in 111 local governments in 11 countries—Benin, Burkina Faso, Côte d’Ivoire, India, Kenya, Niger, Nigeria, the Philippines (joined in 2020 and not included in this analysis since it is new to using MSC), Senegal, Tanzania, and Uganda—with regional support provided by 5 accelerator hubs: Jhpiego in East Africa (EA), IntraHealth International in Francophone West Africa (FWA), Population Services International in India, Johns Hopkins Center for Communication Programs (CCP) in Nigeria, and Zuellig Family Foundation in the Philippines. The global-level management team based at the Johns Hopkins Bloomberg School of Public Health leads TCI’s overall strategy and learning agenda and coordinates the planning, monitoring, evaluation, and reporting efforts. The global-level management team is led by the Bill & Melinda Gates Institute for Population & Reproductive Health with support from CCP to manage MSC data collection and analysis efforts and knowledge management and to provide oversight to the KM coordinators and focal points at each hub team.

TCI nurtures a social learning environment among project staff and partners that thrives on learning by doing and learning to action. Interested local city governments apply for TCI’s support, in the form of financial resources and coaching support, and commit to contributing a portion of the funding needed to adapt, implement, and scale up a diverse range of evidence-based service delivery, demand generation, and advocacy approaches. For scale-up to occur sustainably, TCI affects 4 domains of change: (1) changes in knowledge, attitudes, and practice among project stakeholders; (2) changes in political and financial commitments of local governments; (3) changes in health systems; and (4) changes in service access and quality (Table).^12^^,^^13^ These domains cover aspects of both horizontal (service expansion or replication) and vertical scale-up (institutionalization, e.g., changes to policies, budgets, training, supervision, procurement, and reporting systems),^12^ as well as the necessary precursors to scale-up of knowledge, attitudes, and practices. As capacity and ownership of the evidence-based interventions grow among government officials, TCI gradually reduces its direct financial and coaching support, with these leaders assuming full oversight of implementation within 3–4 years from the inception of the partnership.

**TABLE.** Definitions of The Challenge Initiative’s 4 Domains for Sustainable Scale-Up^a^

**Domain of Change**	**Definition**
Knowledge, attitudes, practice	The progression from awareness of an innovation, to forming positive attitudes about the innovation, to the adoption of knowledge for decision-making purposes or for application in practice and policy.
Political and financial commitments	The multidimensional nature of political commitment is usually captured through the level of spending on intervention since it requires action by both the executive and legislative branches of government—implying commitment of funds and establishment of enabling policies. Statements of leaders are also commonly examined to measure political commitment.
Systems	The building blocks that make health systems function well: leadership and governance, health workforce, medical products and technologies, information and research, including data demand and use.^12^
Access and quality	Elements that impact access to family planning services, such as geographic distance, economic, administrative, awareness of services, and psychosocial issues (e.g., individual attitudes or social norms). Elements that impact the quality of services, such as choice of methods; appropriate client-provider interaction; and availability of competent providers, space, and an appropriate constellation of services.^13^

^a^ The definitions are adapted from The Challenge Initiative’s Global Toolkit on Most Significant Change, Step 2: Selecting the Most Significant Stories (https://tciurbanhealth.org/courses/program-design/lessons/most-significant-change/).

## THE RATIONALE FOR SELECTING MSC

TCI has adapted the MSC technique as its primary qualitative data collection approach, complemented by traditional quantitative data, because the method is known to make sense of complex program impacts in dynamic contexts, such as the ones that TCI operates in. At the same time, MSC, as a story-based method, is easy to communicate across different cultures by asking stakeholders and community members to answer 3 broad questions adapted from Dart and Davies^3^: In the last month/quarter, what were the changes as a result of your engagement with TCI? What was the most significant change? And why do you think it is significant? (Supplement 1 and Supplement 2 include a template of TCI’s MSC interview guide in English and French, respectively.) By deliberately framing these questions broadly, the method prevents overlooking intangible and unexpected outcomes, and it allows for different perspectives from its diverse stakeholders.^5^

TCI adapted the MSC technique as its primary qualitative data collection approach because the method is known to make sense of complex program impacts in dynamic contexts.

Analysis of MSC stories helps TCI identify common themes and trends mapped to its 4 domains of change across hubs and their unique local governments. By triangulating qualitative data from MSC stories with quantitative data from national health management information systems, TCI aims to understand **what** TCI is accomplishing and **how**. The goal is to **use** the data emerging from these data sources to inform decision making and enhance program performance, leading to more efficient processes and effective outcomes. Between July 2018 and June 2021, 260 MSC stories had been collected from all the hubs combined.

The goal is to use the data emerging from these data sources to inform decision making and enhance program performance, leading to more efficient processes and effective outcomes.

## METHODS

### Research Questions

Since TCI first started using MSC in 2018, staff members from the different hubs and the global-level management team have met both virtually and in person as a consortium on several occasions to share experiences using the method. In early 2021, TCI conducted an assessment to document the project’s use and adaptation of MSC and determine its added value in TCI’s adaptive management effort.

The assessment team (i.e., coauthors of this article) aimed to address the following research questions.

1. **Operationalization:** How has each of the hubs operationalized and institutionalized the MSC technique? Are there any notable similarities or differences?
2. **Value-added:** How has the MSC technique added value to TCI’s adaptive management efforts?
3. **Challenges and solutions:** To what extent have the hubs faced any challenges in institutionalizing the MSC technique? How have they resolved the challenges?

### Data Collection and Analysis

Two primary data sources were used to compare and analyze the use of MSC across TCI’s regional hubs. In January 2021, we invited 10 to 25 TCI staff members who were involved in MSC story collection and selection for each session to participate in virtual focus group discussions (FGDs). In the FGDs, participants discussed what has worked well, what could be improved, and specific changes they would like to make to how they implement MSC to strengthen overall project adaptive management. We convened all participants in a combined feedback session to report out and learn about other hubs’ experiences. In total, about 80 TCI program staff members participated in the 4 FGDs.

In February 2021, we conducted 4 virtual key informant interviews (KIIs) with the MSC focal person at each hub who is responsible for knowledge management and learning. These focal persons facilitated the overall MSC process at their respective hubs, including providing training support to collect stories, coordinating MSC data collection, facilitating the MSC selection process at the hub level, and documenting the outcomes from the selection meeting and sharing the report on the selected stories with the global-level management team. The KIIs aimed to capture specific processes that each hub had taken to adapt and implement the MSC technique in its overall monitoring and learning efforts.

We audio-recorded and transcribed or took detailed notes of the FGDs and KIIs. We manually coded the qualitative data, grouped them into main themes corresponding to the research questions, and identified subthemes and trends.

## RESULTS

### Introduction of the MSC Technique to the Hub Teams

The global-level management team first focused on raising interest in and getting buy-in to MSC among the leadership of each hub before reaching out to their staff members working with the local governments. All hub teams learned about the technique and process during a formal kick-off webinar. They discussed various operational issues, for example, how they could request additional funding to support institutionalizing MSC and address staffing needs. The hub teams thought it was critical to create a new position or form a team to manage MSC data collection.

The global-level management team first focused on raising interest in and getting buy-in to the MSC technique.

*Each hub must have a knowledge management team to direct, facilitate, and monitor the entire process and be available to support local staff members to write stories after the interviews*. —India hub team member

Each hub team pilot-tested the MSC interview guide and met virtually to share their internal reflections, experiences, and findings from the pilot phase. The interview guide was further refined, and a 2- to 3-day in-person training was held in each hub. The English and French training agenda and materials used are included in Supplement 3 and Supplement 4, respectively. Participants included hub staff members from the headquarter office and those residing in the cities providing direct coaching support to local governments. The training included interactive and participatory sessions, including role-plays to collect stories via interviews. Participants tried various approaches to select the most significant stories and discussed what would work best for their respective hubs based on their unique cultural context and organizational structure. On the final training day, participants developed an action plan to roll out the technique and process locally. As each hub began collecting and selecting stories, the global-level management team continued to provide virtual support and feedback. It also hosted mini-refresher and follow-up sessions to facilitate continuous learning, reinforce skills, and address staff turnovers during the quarterly project review meetings.

Some of the hub coordinators noted that the initial take-off was not as smooth as anticipated.

*A few states were on board initially, but others felt it was a burden added to their jobs and responsibilities. At the time of the MSC training, many participants did not fully understand how much we could do with the MSC technique to showcase the impact that we are making. It is more than storytelling. —*Nigeria hub team member

*Initially, people were not confident. As they gained more experience and saw the benefits, the confidence had gone up.* —EA hub team member

*To get everyone comfortable, it would be beneficial to have refresher training for story collectors. It could be for just a few hours. We need to ensure that new team members have an opportunity to learn the process.* —FWA hub team member

### Identification and Collection of Stories

The task of identifying and collecting stories became the responsibility of local hub staff who provided coaching support to government stakeholders on implementing and scaling TCI’s evidence-based interventions. All hub teams agreed that the institutionalization of the MSC technique was possible because hub coaches are in regular contact and have well-established relationships with project stakeholders.

*MSC can happen only by having country/city managers identify what is working, what is changing, etc. on the ground. The challenge is that they may not have an eye for detecting potential cases or stories in their busy daily work.* —FWA hub team member

Some hubs noted that the staff members, who identify primarily as program managers and not researchers or M&E officers, needed more practice to strengthen their qualitative data collection skills to sufficiently probe to capture comprehensive stories. It took some time for the hub teams to start receiving an adequate number of stories meeting the criteria of answering all of the MSC questions and providing sufficient context about how the change reported in the story came about.

*We should have included this qualitative data collection as part of the field team's job responsibility so that it was clear that such skills are required and expected, instead of training them later as an add-on task.* —Nigeria hub team member

Regarding the decision on who to interview for potential stories, the participants reported 2 different approaches: ad hoc collection and scheduled collection (Box 1).

BOX 1 **Approaches That TCI Hub Staff Used to Collect MSC Stories**

**Ad Hoc Collection** (**East Africa, Francophone West Africa, Nigeria**)

In EA, FWA, and Nigeria hub teams, city-level hub staff reported looking for opportunities to interview TCI stakeholders after noteworthy events and/or observing changes themselves. Their regular meetings and coaching sessions offered them an opportunity to collect stories. City-level hub staff interviewed stakeholders using the MSC interview guide, drafted the story based on the interviews, and sent the stories to the MSC focal person at the hub headquarters.

**Scheduled Collection (India)**

The India hub team reported using a unique process by which its city-level staff verbally shared observed changes from their assigned areas in 2 minutes or less once quarterly during all-staff review meetings before stories were selected for verification through interviews with stakeholders.

*It is a fantastic team engagement activity. We see immense interest and excitement among cities to present stories.* —India hub team member

*This unique short-pitch format allowed city-level staff to learn quickly from each other’s experience and gave the hub management team an opportunity to listen and make a selection as to which should become MSC stories.* —India hub team member

Abbreviations: MSC, Most Significant Change; TCI, The Challenge Initiative.

The number and type of stories collected from all the hubs, regarding interviewees, local government, evidence-based intervention, and other characteristics, could vary greatly in each quarterly reporting period.

### Selecting and Refining the Stories

MSC uses an iterative process of selecting and pooling the collected stories, typically embedded within an organization’s hierarchical structure, to summarize the large volume of stories into a smaller number of widely valued and applicable stories. Different methods can be used for selection, such as majority rules, iterative voting, scoring, or secret ballot.^3^ Each of the TCI hubs and the final selection committee established within the global-level management team use a combination of methods (Figure).

MSC uses an iterative process of selecting and pooling the collected stories to summarize the large volume stories into a smaller number.

**FIGURE:**
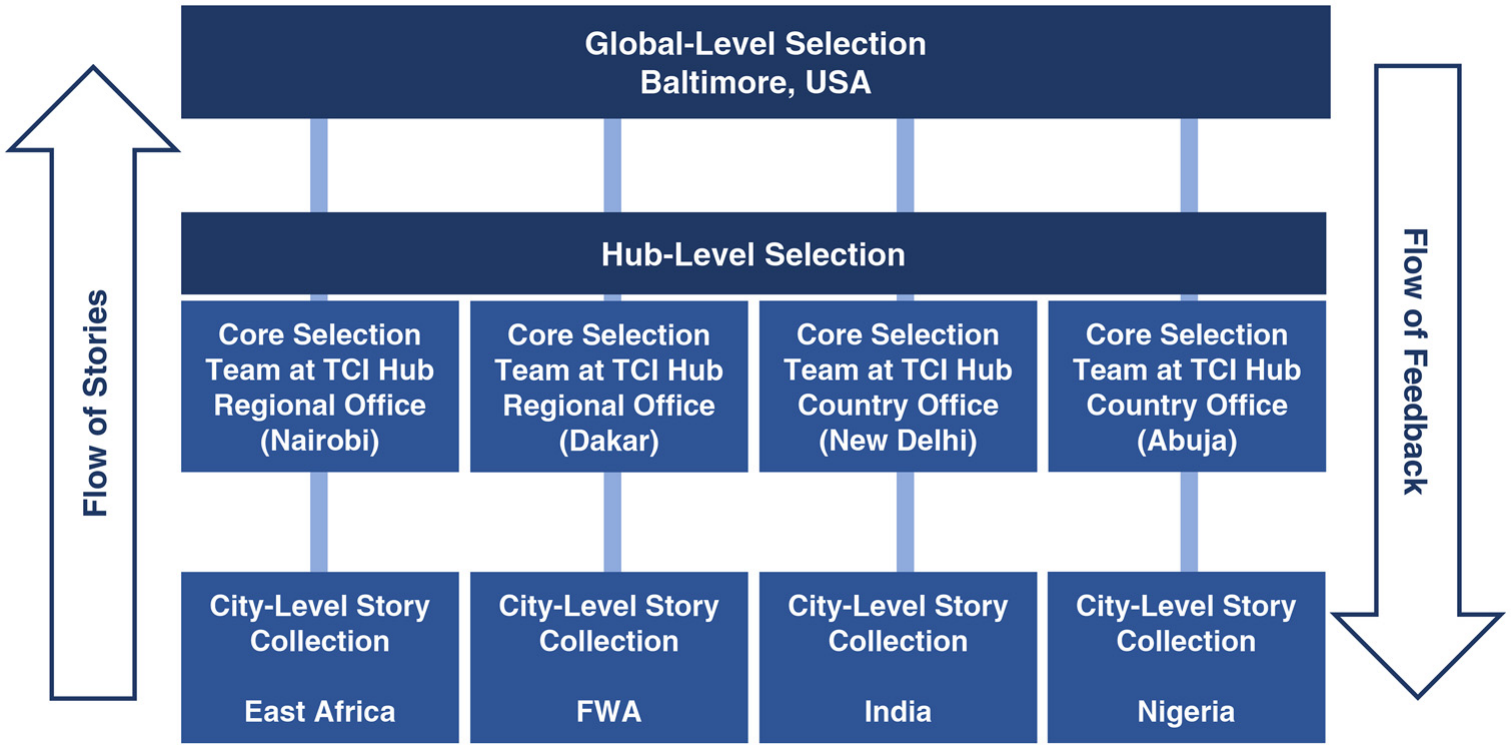
The Challenge Initiative’s Most Significant Change Story Selection Process Abbreviations: FWA, Francophone West Africa; TCI, The Challenge Initiative.

#### Hub Team Experience

All hub teams reported reviewing and selecting the MSC stories quarterly, corresponding to the frequency of TCI progress report requirements. Whenever possible, the teams used quantitative data on monitoring indicators to back up the changes reported in the MSC stories, thereby further validating the MSC story. Data triangulation happened when the reported outcomes related to the routine monitoring data, such as increases in funding or clinic visits. The teams said they spent substantial time and effort gathering missing information from the stories, verifying the outcomes reported in the stories and refining or improving the stories as needed. This clarification process was a critical element in ensuring that each story met the quality and credibility criteria and was ready for the hub selection panel.

The hub-level selection panels have 4–8 members representing various job functions or technical areas. The hubs developed a unique selection process by combining some of the selection methods suitable for them (Box 2). They all included a feedback session, but the timing of holding the session differed (i.e., the beginning, middle, or end of the selection process).

BOX 2 **TCI Hub Teams’ Unique Selection Processes to Choose Stories**

**Combination of scoring, anonymous voting, and feedback session (EA):** The panel uses a scoring form to rate each story anonymously. The rating criteria include the importance, relevance, and learning value of the story. The scores for each story are tallied and shared with all panel members. They review and use the aggregated scores during selection meetings to facilitate open discussion and come to a consensus on which stories to select and why. The selection team also assigns and records relevant domains of change to each story, along with the justifications for selection, in an Excel tracker.

**Combination of feedback session and majority rules (FWA):** The panel members read the stories and then discuss their overall feedback and recommendations for selection based on why they are most significant, with the final decision made based on majority rules.

**Combination of ranking and feedback session (Nigeria):** Each panelist ranks all the stories from the most significant to the least significant and provides feedback on the quality of stories via email. The Nigeria hub staff consolidates all the panelists’ feedback and circulates a brief report on the outcomes from the individual ranking, identifying which stories ranked highest and the reasons for their selection.

**Combination of idea sharing and feedback session (India):** During regular review meetings, a panel of judges composed of 3 TCI hub senior management listen to the 2-minute pitches and select up to 3 finalists each quarter. The panel does not rely on any scoring/ranking protocol but rather discusses how effectively the evidence-based interventions were implemented and if significant learnings could be captured for new and other potential scale-up cities more broadly. Each of the chosen pitches is delegated for an in-depth investigation to become a full MSC story. With support from hub headquarters, city-level staff conduct interviews with different stakeholders for each story to ensure multiple perspectives and insights related to the same observed change. After receiving the full MSC stories from the city, the headquarter team triangulates information with monitoring data. An India team member expressed how the use of qualitative and quantitative data together created more compelling outcomes:

*MSC stories add context to the progress and trends shown by the quantitative data and give good inspiration for local governments and other stakeholders to try the approach [described in the MSC stories] and strengthen their program.* —India hub team member

Abbreviations: MSC, Most Significant Change; TCI, The Challenge Initiative.

#### Final Selection Committee’s Experience

The hubs share the selected stories along with the rationale with the global-level management team’s final selection committee. This global selection committee, composed of 5 project headquarter staff members, conducts the quarterly selection meeting. The members read the stories out loud by each domain of change along with the selection justification provided by the hubs. They discuss the value of each story considering TCI’s overarching goals related to scale, sustainability, impact, and efficiency. The committee members vote for what they think is the top story in a particular domain, and the votes are tallied. On average, the selection committee reviews 14 stories each quarter and selects 4 stories as the most significant, typically 1 story for each of the 4 TCI domains (Table). However, committee members have recorded ties between stories before. If all the members agreed that both stories were equally important, then they might make an exception to select more than 1 story per domain and, therefore, more than 4 stories altogether. Box 3 includes sample extracts from MSC stories.

BOX 3 **Illustrative Extracts From TCI’s MSC Stories**

**Strengthening Community Outreaches in Nairobi, Kenya, Through Coaching**

*We thought that we knew how to conduct outreaches, so we were not using data to guide our decision making. The decision of where to conduct the outreach was based on other factors rather than data. There were no review meetings after the activity and [community health volunteers] were not given targets to work with. This translated into poor planning and preparation.*

*The poor performance of our outreaches prompted us to request for coaching on Outreaches from Tupange Pamoja [how TCI is locally referred to in Kenya]. We were paired with two coaches who coached us on how to conduct high yielding integrated FP outreaches. We were all registered in TCI-U and coached on how to navigate it. We were also coached on the integrated FP outreach and demand creation using the TCI-U toolkit. The project implementation team members participated in this coaching since all of them take part in planning for the outreaches.*

**Mapping and Listing Approach Helps Indore, India, More Accurately Allocate Health Resources**

*We realized that for maximum coverage, we need to list down the complete slum population residing in the urban areas. The urban settings are entirely different from rural settings … It becomes difficult to assess the accurate urban boundaries*.

*Now [after we completed TCI’s proven mapping and listing approach], we will be able to cover the entire slum population as all the facilities have equal distribution of population. … This model is strengthening all aspects of reporting, services and supplies and it is giving magical results, as there is an 18% rise in Madhya Pradesh immunization data during this 4-month duration*.

*The shifting and distribution of responsibilities within existing service providers has enhanced outreach and service quality. In February 2018, there were merely 82 [FP] users. After area segregation, it increased to 1,014 in August 2018. Today, we have baseline data where we can start any program like TB, malaria, etc*.

Abbreviations: FP, family planning; MSC, Most Significant Change; TCI, The Challenge Initiative; TCI-U, TCI University.

### Feedback on MSC Process and Selected Stories

Giving feedback to all stakeholders, including those who provided the significant change stories, is an important step in the MSC process, and should ideally happen every time stories are selected so that subsequent rounds of story collection and selection are informed by feedback from previous rounds. Feedback at different levels also helps to create ongoing dialogue among various stakeholders about what is significant change, providing an indication of where the project should focus its efforts.^3^

Feedback at different levels helps create ongoing dialogue among stakeholders on where the project should focus its efforts.

The global-level management team shares short reports with all hubs on the final selection committee’s decisions, including how the selection process was organized, the selected stories, and reasons why they were selected. The hubs then organize feedback sessions with their stakeholders in their respective locations, leveraging quarterly project review meetings that convene a broad base of stakeholders, including government representatives, local health officials and implementers, and other development partners who initially provided stories from their cities. Other occasions for sharing feedback include city health management team meetings and TCI coaching sessions. During the feedback sessions, the hub teams review the comments and suggestions from the global selection committee. They also discuss feedback on the MSC process itself, such as how they could fulfill all requirements for a “complete” MSC story and how to improve the interviews to ensure thorough responses. Feedback at previous selection levels has not happened as systematically as it does at the global level. All hub teams recognized that feedback from the global level had been an indispensable step in their overall M&E, learning, and collaboration efforts.

As an India hub team member stated, the feedback sessions gave various stakeholders opportunities to “use qualitative evidence from MSC stories to complement quantitative data collected to assess how each city is doing regarding the program maturity.”

Several hub team members pointed out concrete benefits of informing their communities and program beneficiaries when the stories were selected by the global committee. Community members and beneficiaries considered that the global selection committee had valued their efforts and felt motivated to continue to document and share stories of change.

The global-level management team and hub teams use additional tools and communication approaches to facilitate further learning and ongoing dialogue about MSC stories. TCI India writes detailed reports about the MSC selection, including trends data, and periodically shares them with the local-level stakeholders. Additionally, the global-level management team holds biannual virtual meetings with all TCI staff to discuss the MSC technique and findings to date and encourage various cross-learning opportunities. MSC stories are often shared widely via TCI blog posts, internal and external e-newsletters, and social media and community of practice messages.

### Using Stories to Improve the Overall Program

The formal process of feeding stories back at regularly scheduled review meetings had fostered learning opportunities and increased the diffusion and adoption of evidence-based interventions across various local governments.

*MSC stories allow cities to document and highlight significant outcomes. Due to the MSC stories, representatives from nearby cities, including politicians, often visit TCI cities. One good example is the adolescent and youth-focused approach now implemented by many TCI cities. —* EA hub team member

In India, the use of the city coordination committee approach to address staff shortages had become a standard practice in many TCI cities after it was featured as an MSC story.

*Stories are helpful for us to identify what is happening in 1 city and can become great advocacy tools to inspire government counterparts in other cities.* —India hub team member

Another India hub staff reported the successful replication of a proven approach due to the learning exchange from MSC.

*A good example is the MSC story about the mapping and listing of all health facilities in Indore’s defined catchment area. This activity has helped map out the left out and underserved places. Other local governments in Madhya Pradesh replicated the mapping exercise and found out more than 50% of its slum population had been left out of its previous estimates.* —India hub team member

A Nigeria hub team member explained the outcome of the midyear review meeting.

*One state demonstrated how it introduced a simple tool in its supported health facilities to increase data use for decision making and planning for in-reaches and outreaches. As a result, 2 states also introduced this technique in their supported facilities.* —Nigeria hub team member

Another Nigeria team member added how cross-learning happens among stakeholders constantly.

*Local city governments learn from each other through sharing via the WhatsApp group and during the mid-year review and work planning meetings where stories are shared. For example, Niger State shared the MSC story describing how it introduced run charts in its supported facilities to increase data use for decision making and planning for in-reaches and outreaches. As a result, Rivers State and Plateau State also introduced this simple tool/technique.* —Nigeria hub team member

Not all hub team members could provide a specific example of using stories to improve their program efforts. However, they at least recognized the importance of learning from the process and felt that MSC stories had some influence on program activities. Some team members, in particular, those who were new to the project, said that the MSC process was sparking internal discussions but had not yet manifested into concrete action steps.

*We question ourselves about how we might adapt here and there in terms of implementation but not something that we are putting on paper about what we need to do yet. The process of adaptation was happening rather organically, not systematically. Our team members and stakeholders may need more capacity strengthening in the area. —*FWA hub member

Several hub team members discussed specific cases of learning from other hubs. For example, the MSC stories about the health facility makeover approach initially developed and implemented in Nigeria gained popularity in Uganda. After reading the stories from Nigeria, the EA hub team met with Nigeria hub team experts and visited a site to learn how to organize and implement facility makeovers.

*The effective use of photos showing before and after the facility makeovers contributed to successful advocacy efforts undertaken by others. The inclusion of quantitative data, such as the increase in client visitations and satisfaction, in the MSC stories help to justify the approach*. — Nigeria hub team member

The diffusion of proven practices and the shifts in strategic directions due to the MSC technique at various project levels (i.e., at the city, hub, and global coordination) have been well-documented in routine progress reports and as new intervention guidance documents. (Box 4 has illustrative examples of how the MSC technique was used for adaptive management.)

BOX 4 **Illustrative Examples of MSC and Adaptive Management**

**Nigeria: The “Top Learnings From This Quarter” Bulletin**

The TCI Nigeria hub has consolidated critical learnings from MSC stories and other monitoring data into a short document (the bulletin). As a result, other cities and states facing similar challenges could consider and apply solutions that have been tested and proven to work. Below is an excerpt from the bulletin.

***Have you started to see a decrease in FP service uptake?** Consider learning from Taraba, Delta, and Abia States’ experience implementing in-reaches. TCI helped the States to identify facilities with limited uptake of FP services using facility-level data. TCI also provided technical assistance to the states to outsource service providers from facilities with at least 2 providers to support the provision of FP services at in-reach facilities on designated days. More than 480 persons accepted FP services as a result of these in-reaches.*

Based on the learnings, Niger State implemented a similar approach and later documented in the progress report that the state recorded that close to 6,500 young people had accessed contraceptive services from in-reaches conducted across 30 facilities in the states.

**India: The Eight Steps to Activate a New City for FP**

The systematic and routine sharing of the MSC stories led to codifying the lessons learned and good practices into an online training tool for the TCI India hub to onboard new city managers to rapidly establish quality FP services in a city (available at https://tciurbanhealth.org/courses/india-advocacy/lessons/eight-steps-to-activate-a-new-city-for-family-planning/). There is also an accompanying step-by-step infographic (available at https://tciurbanhealth.org/wp-content/uploads/2021/11/TCI-New-City-for-FP-8-steps.pdf). Since its formal launch in November 2019, the training tool has recorded about 750 page views in 2 years.

Abbreviations: FP, family planning; MSC, Most Significant Change; TCI, The Challenge Initiative.

## DISCUSSION

Overall, TCI has had a positive experience with using MSC to facilitate learning and adaptive management across diverse project stakeholders in multiple countries. Learning is a prerequisite for adaptive management, which according to USAID,^14^ is “an intentional approach to making decisions and adjustments in response to new information and changes in context.” TCI staff members reported that the use of MSC has created learning opportunities that have helped diffuse evidence-based health interventions both within and across countries, and many could point to specific concrete actions because of MSC learnings. However, other staff members had more difficulty making that direct link but still felt that MSC stories influenced program activities. The responsive feedback step in the MSC process, in particular, was viewed as indispensable to learning and collaboration. Staff members reported several necessary inputs to successful use of the method, including buy-in about the benefits, training on good interviewing techniques and qualitative research, and dedicated staff to manage the process. In addition, staff members thought that institutionalization of the method was possible because the staff members who collected the stories were in regular contact with project stakeholders from whom they collected the stories.

TCI staff reported that the use of MSC has created learning opportunities that have helped diffuse evidence-based health interventions both within and across countries.

### MSC as a Tool to Facilitate Responsive Feedback, Learning, and Adaptive Management

Numerous aspects of the MSC technique make it a useful and valid method for exploring changes in complex environments. Validity is measured in qualitative methods by presenting “thick” or deeply contextualized descriptions.^15^^,^^16^ MSC stories include not only detailed accounts of what happened before, during, and after change events but also have the selection committees’ reasons for selection, adding to the descriptive data. In addition, MSC uses a systematic selection process—in the case of TCI, at 2 levels—which further enhances its validity. Most importantly, MSC promotes the involvement of a wide range of stakeholders and perspectives and elevates the voices of those directly implementing programs in countries, thereby providing invaluable data.

The MSC technique motivates TCI stakeholders and staff members to identify the fundamental changes and achievements that they have observed due to their involvement in TCI programming in their own words. These changes are often not measurable or meaningful as quantitative measures but extremely valuable to the community, recognizing health workers and demonstrating progress toward achieving strengthened health systems. Furthermore, the collection of stories allows various voices to be heard because most people can tell stories naturally, and thus MSC is an easy method to communicate across cultures and levels of the health system. For example, despite northern Nigeria’s conservative social norms on family size and FP, TCI has found that stakeholders from the community to the state level have been willing to share their stories because the storytelling and narrative approach is an integral part of their cultural traditions.

The multiple levels of selection among TCI and giving feedback on the stories to stakeholders enables everyone involved in the project to learn from the stories. Cross-learning is facilitated not only across the cities and countries in which TCI is implemented but also across different stakeholders, from government officials and religious leaders, to service providers and community health workers, to community members. Providing feedback on the stories helps stakeholders feel that their inputs are appreciated and that the efforts they are putting into achieving program deliverables have tangible value. Feedback also encourages stakeholders to monitor gains continuously and be able to report them subsequently. Finally, feedback from the TCI country office teams to the local governments also helps identify gaps in programming revealed by the MSC stories and subsequently opens a dialogue for strategies for program improvement. If MSC were used only for evaluation, it would be more challenging to analyze, interpret, and share the results in a timely manner to facilitate this learning and feedback process.

Finally, TCI has found that the use of MSC stories as advocacy and accountability tools has helped to facilitate more prompt decision making by local governments and increased their commitments to FP programming. Feedback from the MSC story was often shared with political leadership, facilitating the continued allocation of resources for FP and adolescent and youth sexual and reproductive health. MSC stories are also shared more broadly via multiple channels, including TCI University (the project’s online website that synthesizes practical FP program guidance and tools), TCI’s community of practice, and social media channels, and often serve as the needed evidence for other local officials not directly engaged with TCI to adopt and replicate interventions because they too wanted to witness similar results in their context. Collecting MSC stories also highlighted to TCI which high-impact interventions appeared to be the most valued by its stakeholders and why. Service statistics are often used to measure impact; however, TCI has learned from its MSC stories the importance of creating an enabling environment for FP services is key to ensure that women and couples with unmet needs for FP show up at clinics for services.

## LESSONS LEARNED AND RECOMMENDATIONS FOR USING MSC AS AN ADAPTIVE MANAGEMENT TOOL

The assessment has identified several lessons and recommendations to integrate the MSC technique into routine monitoring and adaptive management. Global health professionals who are embarking on similar initiatives may consider the following enabling factors.

### Ensure Leadership Buy-In and a Shared Vision for Using MSC for Adaptive Management

M&E for adaptive management requires the ability to think strategically, identify emerging patterns, build relationships with stakeholders, communicate with different groups, and persuade others, and such ability is likely to become more important than knowledge and experience of traditional M&E methods.^17^ Project leadership must see the value of using MSC for monitoring and adaptive management, articulate this value, and champion its use with staff. TCI’s hub leadership explained to the teams involved in collecting MSC stories that the stories would help them perform their jobs better and improve project outcomes. Team members knew the importance of hearing directly from stakeholders regularly, providing context to the quantitative data already being collected, and ensuring an opportunity to learn from each other by sharing the collected stories.

Project leadership must see the value of using MSC for monitoring and adaptive management, articulate this value, and champion its use with staff.

### Make Data Collection Part of Everyone’s Job and Strengthen Staff Capacity to Collect Stories With Support and Feedback

TCI has demonstrated that staff members directly implementing programs can effectively collect MSC stories. However, adding additional responsibilities to staff who are already busy fulfilling their primary function of coordinating and implementing activities is not straightforward. To ensure smooth introduction and integration of MSC in their job responsibilities, it is critical to secure adequate time for training and practice until they feel confident. Refresher training is essential because programmatic staff are not always comfortable with probing the interviewees to elicit necessary details.^4^ It is also beneficial to have a dedicated staff responsible for coordinating MSC implementation and supporting story collectors regularly. Field staff members and local government officials who collect and write stories appreciate having input from the hub teams that review the stories. The ongoing feedback process is essential to keep them motivated to strengthen their interviewing and data collection skills continually.

### Develop Standard Guidance to Integrate MSC in Routine Monitoring and Allow Teams to Adapt the Process

MSC data collection, selection, and feedback process should align with routine tasks and regular meetings. The integration of the MSC technique for adaptive management requires a systematic and intentional effort, and therefore it is critical to develop clear and simple guidance, a simple story collection template, and a straightforward process that each team can follow easily. At the same time, each team should have some flexibility to adapt and refine the process, including the data collection frequency and the selection approach, to meet the needs and unique context of the team. Additionally, team members appreciate having some room to address initial challenges and potential mistakes and the opportunity to refine their process continually.

### Ensure Collection and Learning From the Community Level to Higher Levels of the Health System

MSC data collection not only supports and strengthens good working relationships between project staff and stakeholders but also enables learning from the lowest levels of the health system, including from the community to the highest levels of the health system, where resources are allocated and spent on interventions and services. This holistic knowledge exchange strengthens the overall health system to be more responsive, effective, and efficient in how it works. Local languages can be used at the initial story collection stage to capture the voice of stakeholders at the community level as accurately as possible by using their own words and then translating them into a common language. Having the final stories in a common language (English and French in the case of TCI) can ensure consistency for the story review and selection stage and encourage wider sharing and learning.

### Triangulate Data to Support Significant Changes Reported in Stories and Speak to a Range of Decision Makers

Since the MSC technique asks interviewees to focus on the most significant change they experienced, the stories may not represent the average experience. Triangulating qualitative data with quantitative data helps verify whether the reported changes in MSC stories have been documented in other data collection methods and could thus apply to a broader range of stakeholders than just the particular storyteller. At the same time, MSC stories also provide context to the quantitative monitoring data and help the teams to appreciate the impact of the proven practices, providing the evidence needed to identify priority areas and make sound decisions. Findings from the MSC stories can be used during periodic performance reviews to assess progress, corroborate solid and weak areas, and facilitate corrective action.

MSC stories also provide context to the quantitative monitoring data and help the teams to appreciate the impact of the proven practices.

## POTENTIAL CHALLENGES OF USING MSC FOR ADAPTIVE MANAGEMENT

MSC requires well-trained data collectors to solicit the level of detail needed to understand the situation before the change happened, the nature and type of support provided that prompted the change, and the difference that the change made. While data collectors from each regional hub received sufficient training on implementing the MSC technique, their comfort level and experience with conducting interviews, and therefore their skill in doing so, vary greatly. Program managers should know that such skill differences may affect the quality of the stories and provide additional support to those not meeting the required story standard.

Since TCI’s data collectors regularly contact the interviewees through their coaching sessions, there may be selection and/or courtesy bias. Specifically, the data collectors may select to interview only individuals who have had positive experiences. Furthermore, the interviewees tend to report mainly positive changes (even though changes can be either positive or negative). One way we tried to address this issue is by ending the interviews by asking specifically about challenges the storytellers have faced in implementing interventions. However, we have found that the challenges reported by the storytellers tend to focus on the situation before TCI’s involvement. It seems that the data collectors do not probe further to uncover persistent challenges related to implementing TCI’s evidence-based interventions or unintended negative consequences. Additionally, while TCI has used the MSC technique for the adaptive management purpose, we have noted that occasionally positive outcomes from the most significant stories are featured in the project’s promotion and communication materials. This unintended consequence may have contributed to the tendency to report stories attractive for communication purposes.

## CONCLUSION

TCI has institutionalized the use of the MSC technique across multiple countries and diverse stakeholders, and the findings from our assessment suggest that it is an effective qualitative data collection tool to strengthen routine monitoring and adaptive management efforts. While the method makes use of standardized steps, including collecting stories of significant change, selecting the most significant stories by panels of project stakeholders, feeding back the results of the selection process, and using the insights gained about what the project values to improve and adapt implementation, it also allows for flexibility in how project stakeholders implement the process such as how stories are selected. The MSC technique could be a particularly important tool for global health practitioners, policy makers, and researchers working on complex interventions because they continually need to understand stakeholders’ needs and priorities, learn from lessons and evidence-based practices, and be agile about addressing potential challenges.

## Supplementary Material

21-00624-Ohkubo-Supplement1.pdf

21-00624-Ohkubo-Supplement4.pdf

21-00624-Ohkubo-Supplement2.pdf

21-00624-Ohkubo-Supplement3.pdf

## Notes

**Peer Reviewed**

## References

[1] Norris SL Rehfuess EA Smith H. Complex health interventions in complex systems: improving the process and methods for evidence-informed health decisions. BMJ Glob Health. 2019;4(Suppl 1):e000963. 10.1136/bmjgh-2018-000963. 30775018 PMC6350736

[2] Davies R. An evolutionary approach to facilitating organisational learning: an experiment by the Christian Commission for Development in Bangladesh. Impact Assess Proj Apprais. 1998;16(3):243–250. 10.1080/14615517.1998.10590213

[3] Dart J Davies R. A dialogical, story-based evaluation tool: the most significant change technique. Am J Eval. 2003;24(2):137–155. 10.1177/109821400302400202

[4] Tonkin K Silver H Pimentel J. How beneficiaries see complex health interventions: a practice review of the Most Significant Change in ten countries. Arch Public Health. 2021;79(1):18. 10.1186/s13690-021-00536-0. 33557938 PMC7871616

[5] Mwaikambo L Brittingham S Ohkubo S. Key factors to facilitate locally driven family planning programming: a qualitative analysis of urban stakeholder perspectives in Africa and Asia. Global Health. 2021;17(1):75. 10.1186/s12992-021-00717-0. 34217354 PMC8254949

[6] MEASURE Evaluation. Experiences and Lessons Learned: Implementing the Most Significant Change Method. Carolina Population Center, University of North Carolina at Chapel Hill, MEASURE Evaluation; 2020. Accessed February 10, 2022. https://www.measureevaluation.org/resources/publications/fs-19-410.html

[7] Apgar M Hernandez K Ton G. Contribution Analysis for Adaptive Management. Briefing Note. ODI; 2020. Accessed February 10, 2022. http://cdn.odi.org/media/documents/glam_contribution_analysis_final.pdf

[8] Paz-Ybarnegaray R Douthwaite B. Outcome evidencing: a method for enabling and evaluating program intervention in complex systems. Am J Eval. 2017;38(2):275–293. 10.1177/1098214016676573

[9] Pasanen T Barnett I. Supporting Adaptive Management. Working Paper 569. ODI; 2019. Accessed February 10, 2022. https://cdn.odi.org/media/documents/odi-ml-adaptivemanagement-wp569-jan20.pdf

[10] Complexity-aware monitoring: explore monitoring innovations for adaptive management. USAID Learning Lab. Accessed February 10, 2022. https://usaidlearninglab.org/complexity-aware-monitoring/approaches

[11] Viswanath K Synowiec C Agha S. Responsive feedback: towards a new paradigm to enhance intervention effectiveness. Gates Open Res. 2019;3:781. 10.12688/gatesopenres.12937.2. 31131370 PMC6480401

[12] World Health Organization (WHO). *Practical Guidance for Scaling Up Health Service Innovations*. WHO; 2009. Accessed February 10, 2022. https://expandnet.net/PDFs/WHO_ExpandNet_Practical_Guide_published.pdf

[13] Bertrand JT Hardee K Magnani RJ Angle MA. Access, quality of care and medical barriers in family planning programs. Int Fam Plan Perspect. 1995;21(2):64. 10.2307/2133525

[14] USAID. Bureau for Policy, Planning and Learning. *Discussion Note: Adaptive Management*. USAID; 2021. Accessed February 10, 2022. https://usaidlearninglab.org/sites/default/files/resource/files/dn_adaptive_management_final2021.pdf

[15] Geertz C. *The Interpretation of Cultures: Selected Essays*. Basic Books; 1973.

[16] Aspers P Corte U. What is qualitative in qualitative research. Qual Sociol. 2019;42(2):139–160. 10.1007/s11133-019-9413-7. 31105362 PMC6494783

[17] Simister N. Making M&E work for adaptive management requires strategic skills. *INTRAC Blog*. Posted May 25, 2018. Accessed February 10, 2022. https://www.intrac.org/making-work-adaptive-management-requires-strategic-skills

