# Supplementary material for: Lessons Learned From the Use of the Most Significant Change Technique for Adaptive Management of Complex Health Interventions
Source: Glob Health Sci Pract. 2022 Feb 28;10(1):e2100624. doi: 10.9745/GHSP-D-21-00624 (PMC8885358; doi:10.9745/GHSP-D-21-00624)
Supplement: 21-00624-Ohkubo-Supplement1.pdf [file 21-00624-Ohkubo-Supplement1.pdf]

Supplement to: Ohkubo S, Mwaikambo L, Salem R, Ajijola L, Nyachae P, Kumar Sharma M. Lessons learned from the use of most significant change technique for adaptive management of complex health interventions. *Glob Health Sci Pract*. 2022;10(1):e2100624. https://doi.org/10.9745/GHSP-D-21-00624

TCI MSC Interview Guide

Introduction

TCI is collecting stories from various stakeholders to help us better understand *what* TCI is accomplishing and *how* we can replicate successes and improve the initiative and our support to you. We would like to ask you some questions about any significant change you have witnessed or experienced in urban reproductive health programs as a result of your involvement in TCI. We are looking for both positive and negative changes because these will help us to learn, adapt, and ultimately scale up high-quality family planning services in a sustainable way. There are no right or wrong answers. We simply want to know more from your perspective.

The interview should take no more than 15-30 minutes and is completely voluntary. There is no penalty if you decide not to participate or if you quit at any time.

When answering questions, please be as detailed as possible even if you think I may know the background of your story. Since we intend to share these stories with public health professionals globally, please provide as much detail as possible about your context so that others can have a clear understanding of your story. I will be taking notes as we speak and would like to audio record this conversation. First, I would like to obtain your consent before we begin the interview.

Consent Form

We may use your story in reports to our funder and share with other TCI stakeholders in this country and in other TCI regions. We may also share your story more broadly in global publications and resources, such as the TCI website and journal articles.

Do you consent to:

- Having your name and organization associated with the story? ☐ YES ☐ NO
- Having your image or likeness associated with the story? ☐ YES ☐ NO
- Allowing an audio recording of your story/this interview? ☐ YES ☐ NO

I understand that TCI may use and publish my image or likeness, story and/or quotes from this interview, and related media content (photo, audio, and/ or video) in an appropriate, fair, and respectful manner. I confirm that this multimedia content was created with my knowledge and consent. I understand that I will not receive compensation at this time or anytime in the future for use of this content.

____

Print Name

____

Signature Date

Supplement to: Ohkubo S, Mwaikambo L, Salem R, Ajijola L, Nyachae P, Kumar Sharma M. Lessons learned from the use of most significant change technique for adaptive management of complex health interventions. *Glob Health Sci Pract*. 2022;10(1):e2100624. https://doi.org/10.9745/GHSP-D-21-00624

Background Demographics

1. **Name of storyteller*:** ____
2. **Job title of storyteller:** ____
3. **Employer of storyteller*:** ____
4. **Location (city/town, country):** ____
5. **Gender of storyteller:** ____

** Do not record this information if the storyteller wishes to remain anonymous.*

Start Interview Questions

Now, I'd like to get started by asking you a few questions.

6. In the last month/quarter (please specify the timeframe), what do you think was the most significant change that occurred as a result of TCI?

   NOTE TO INTERVIEWER: If the interviewee is struggling to provide a response, you may want to inform them that this change can be of a personal nature; e.g., did they learn something new or have a change in attitude toward family planning? Or the change could be a facility- or systems-level change.

   PROBES:

   a. What was the situation like prior to TCI?
   b. What happened/what was the change? How did it happen? Who was involved?
   c. What was the situation like after the change?

7. Why do you think this is significant?

   PROBES:
   a. What difference has this made now or will make in the future?
   b. What has been the effect of this change at the individual, community, or city level?

8. What are the challenges that you've experienced or are aware of in implementing the TCI high impact approaches?
