# Supplementary material for: Lessons Learned From the Use of the Most Significant Change Technique for Adaptive Management of Complex Health Interventions
Source: Glob Health Sci Pract. 2022 Feb 28;10(1):e2100624. doi: 10.9745/GHSP-D-21-00624 (PMC8885358; doi:10.9745/GHSP-D-21-00624)
Supplement: 21-00624-Ohkubo-Supplement4.pdf [file 21-00624-Ohkubo-Supplement4.pdf]

Supplément à: Ohkubo S, Mwaikambo L, Salem R, Ajijola L, Nyachae P, Kumar Sharma M. Lessons learned from the use of most significant change technique for adaptive management of complex health interventions. *Glob Health Sci Pract*. 2022;10(1):e2100624. https://doi.org/10.9745/GHSP-D-21-00624

Formation sur le Changement le plus significatif (CPS) à l'intention du Personnel de programme TCI en Afrique de l'Ouest francophone

2018

Cette formation vise à la fois à renforcer les capacités du personnel des plateformes sur le processus de collecte, d'analyse et de partage des connaissances en matière de changement le plus significatif (CPS) à travers l'implication de toutes les parties prenantes de TCI et à élaborer un plan pour sa mise en œuvre au niveau des plateformes.

**Formation sur le Changement le plus significatif (CPS) à l'intention du Personnel de programme TCI en Afrique de l'Ouest francophone**		
**Jour I**	Thème de la session: Étape 1: Collecte d'histoires	Facilitateur
**9:00 – 9:30**	Mot d'ouverture	Schatzi
**9:30 – 10:00**	*Energizer* / introductions et présentation du programme	Sarah
**10:00 – 11:00**	Méthodologie TCI pour la collecte systématique des apprentissages<br>• Présentation de la technique CPS	Sarah
**11:00 – 11:15**	Pause-café	
**11:15- 1:00**	Étape 1: Collecte d'histoires<br>• Pourquoi les histoires et exemples d'histoires CPS[1]<br>• Techniques d'interview notamment l'obtention du consentement<br>• Revue du Guide d'interview relatif au CPS de TCI	Sarah<br>FWA Hub ? (slides 26-33)<br>Sarah
**1:00-2:00**	Déjeuner	
**2:00-3:15**	Exercice de collecte d'histoires entre participants<br>• Travail de groupe<br>• Discussions	Sarah
**3:15 – 3:30**	Pause-café	
**3:30-4:30**	Voir l'histoire: Techniques & éthiques en matière de photographie	FWA Hub ? (slides 47-75)
**4:30 – 5:00**	Exercice de collecte d'histoires entre participants (mêmes paires que précédemment)<br>• Travail de groupe<br>• Discussions	Sarah
**5:00 – 5:30**	Bilan	FWA Hub ?
**DEVOIR À FAIRE CHEZ SOI EN CAS DE NECESSITE DE PLUS DE TEMPS**	Sur la base des deux sessions pratiques, rassemblez les histoires que vous avez recueillies de votre partenaire.	Send to Sarah

[1] Aussi désigné par « histoires relatives ou liées au CPS »

Supplément à: Ohkubo S, Mwaikambo L, Salem R, Ajijola L, Nyachae P, Kumar Sharma M. Lessons learned from the use of most significant change technique for adaptive management of complex health interventions. *Glob Health Sci Pract*. 2022;10(1):e2100624. https://doi.org/10.9745/GHSP-D-21-00624

**JOUR II**	**Thème de la Session: Étapes 2-4**	**Facilitateur**
**9:00 - 9:15**	Rappel	
**9:15 – 11:15**	Opérationnalisation de l'Étape 1: Collecte des histoires<br>• Qui sont les narrateurs et les collecteurs ?<br>• Combien de fois collecterez-vous les histoires ?<br>• Quelles sont les plateformes existantes (réunions et autres points d'interaction) pour la collecte d'histoires	Facilitation de discussions de groupe
**11:15 – 11:30**	Pause-café	
**11:30 – 1:00**	Revue et opérationnalisation de l'Étape 2: Sélection des histoires les plus significatives	Facilitation de discussions de groupe
**1:00 – 2:00**	Déjeuner	
**2:00 – 3:30**	Session pratique à partir des histoires de la journée précédente	Travail de groupe
**3:30 – 3:45**	Pause-café	
**3:45 – 4:15**	Étapes finales du processus CPS<br>• Partage des résultats de la sélection d'histoires (lettre d'information, rapports, vidéos, etc.)<br>• Plans de données à l'action	Sarah
**4:15 – 5:00**	Élaborer un calendrier pour les 4 étapes du processus CPS ainsi que l'exercice de réflexion au niveau des plateformes	Sarah
**5:00 – 5:30**	Observations finales/mot de la fin	FWA Hub ?

**JOUR III**	**Thème de la Session: Planification des actions**	**Facilitateur**
**9:00 - 9:15**	Rappel	
**9:15 – 10:00**	Élaborer un calendrier pour les 4 étapes du processus CPS ainsi que l'exercice de réflexion au niveau des plateformes	
**10 :00-10 :30**	Temps pour compléter des thèmes restants, s'il y en a	
**10:30 – 10:45**	Pause-café	
**10:45 – 11 :30**	Questions / Feedback	
**11 :30-12 :00**	Observations finales/mot de la fin	

Le Changement Plus Significatif

Objectifs de la formation

- Comprendre l'objectif du CPS, sa valeur ajoutée par rapport aux autres approches de S & E, et comment le CPS fonctionne
- Effectuer la mise en œuvre du CPS
- Élaborer un plan opérationnel pour la mise en œuvre du CPS au niveau du plateforme FWA

Jour 1

- Intro au processus du CPS (4 étapes principales)
- Étape 1 du CPS: Collecte d'histoires
  - Technique d'interview
  - Obtention du consentement
    - Pratique
  - Techniques de photographie
  - Éthique de la photographie
    - Pratique
- Récapitulation de la journée

Jour 2

- Elaborer un plan d'action pour la mise en œuvre du CPS au niveau du plateforme FWA
  - Opérationnalisation de l'Etape 1
  - Revue et opérationnalisation de l'Etape 2: Sélection des histoires
  - Revue et Opérationnalisation des Etapes 3 et 4: Feedback et intégration du CPS aux plans de données à l'action
- Réfléchir sur la formation et identifier un plan de suivi avec le niveau Central (Global)

Commençons!

THE CHALLENGE INITIATIVE

Methodologie de TCI pour une acquisition systématique de connaissances

Il s’agit d’un business unusual !

« Si vous saviez ce qui allait se passer chaque jour, vous pourriez faire des choses incroyables. Vous pourriez devenir incroyablement riche, influencer le processus politique et cetera. Eh bien, il s'avère que la plupart des gens ne savent même pas ce qui s'est passé hier dans leur propre entreprise. Ainsi, beaucoup d'entreprises découvrent qu'elles peuvent avoir un énorme avantage concurrentiel simplement en découvrant ce qui s'est passé hier le plus tôt possible. »

-Steve Jobs, 1994

Que savez-vous du CPS?

- Avez-vous entendu parler du CPS?
- Que savez-vous du CPS?

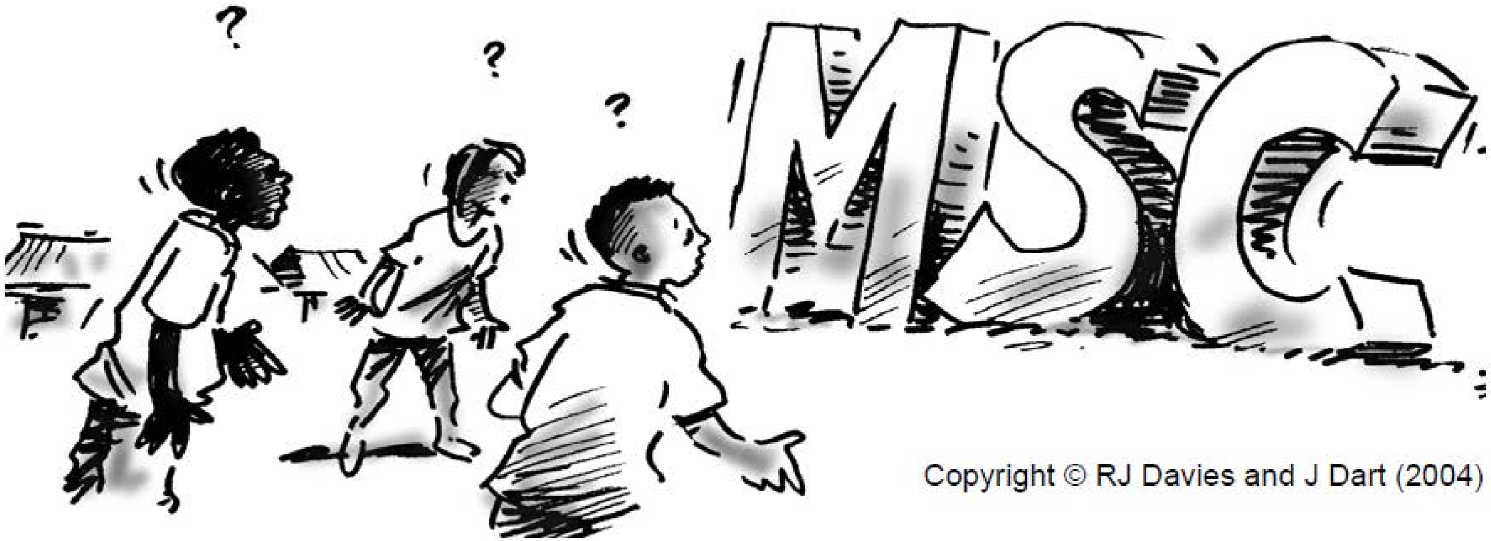

Qu’est-ce que la technique CPS?

- **Collecter des histoires** relatives aux changements significatifs
- **Sélectionner** des histoires les **plus significatives** par les parties prenantes
- **Partager** des histoires sélectionnées avec les parties prenantes
- **Utiliser** les informations pour améliorer les programmes

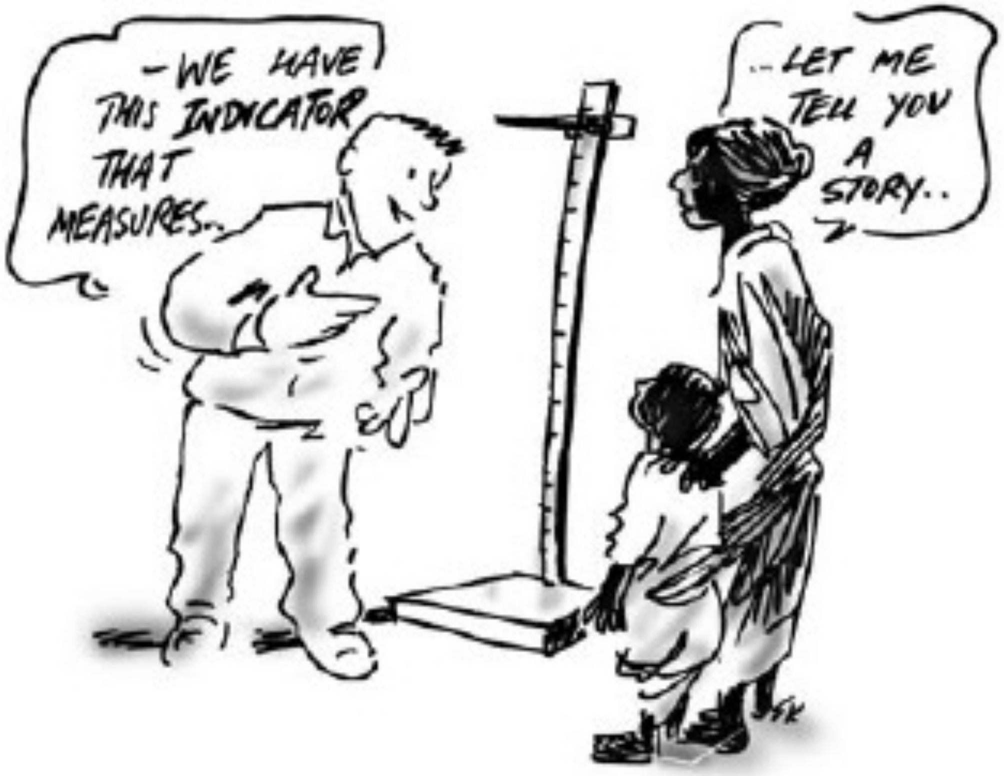

Image from *The Most Significant Change Technique: A Guide to Its Use*, by Rick Davies and Jess Dart.

Qu’est-ce que la technique CPS?

- Développée au début des années 1990 par Rick Davies.
- A été mise en œuvre dans **plus de 20 pays** par de nombreuses organisations (y compris USAID, Oxfam, Fondation Aga Khan, SIDA, DFID, CARE, Asian Development Bank et bien d'autres !)
- Approche participative de suivi et d’évaluation
- Collecter des histoires sur les changements significatifs, puis sélectionner les histoires les plus significatives et les **partager**
- Mise en œuvre **tout au long du cycle du projet**
- Aide à **gérer** un programme d'améliorations itératives ET **fournit des données** sur l'impact et les résultats.

Pourquoi le CPS?

- Permet d'identifier les **changements inattendus** dans les initiatives complexes.
- Les « histoires de changement significatif » **identifient clairement les valeurs de TCI** et nous permettent d'avoir une discussion pratique sur les valeurs les plus importantes.
- Il s'agit d'une forme participative de suivi qui est facile à communiquer à travers les cultures. **Tout le monde** peut raconter des histoires sur des événements qu'ils jugent importants.
- Donne **une image riche de ce qui se passe**, plutôt qu'une image trop simplifiée où les développements organisationnels, sociaux et économiques sont réduits à un seul chiffre.
- **Renforce les capacités** d'analyse de données et de conceptualisation de l'impact

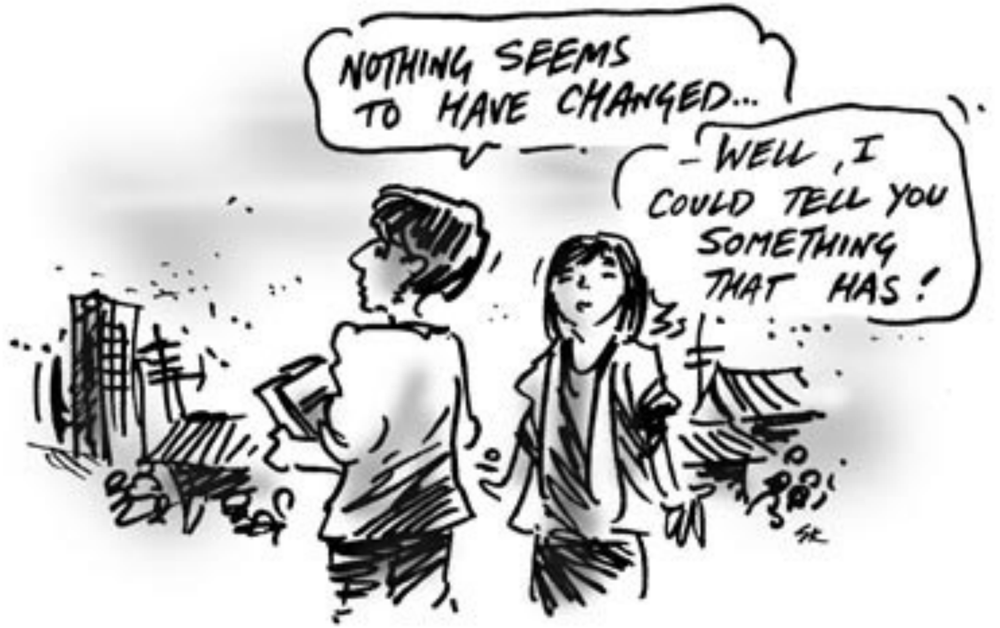

Pourquoi les histoires?

- Les gens racontent les histoires naturellement
- Les gens se souviennent des histoires
- Les histoires peuvent porter de difficiles messages qu'il peut être impossible de recueillir par des moyens autres que celles-ci
- Les histoires offrent de la richesse, la complexité et le contexte
- Les histoires permettent d'avoir des discussions plus profondes

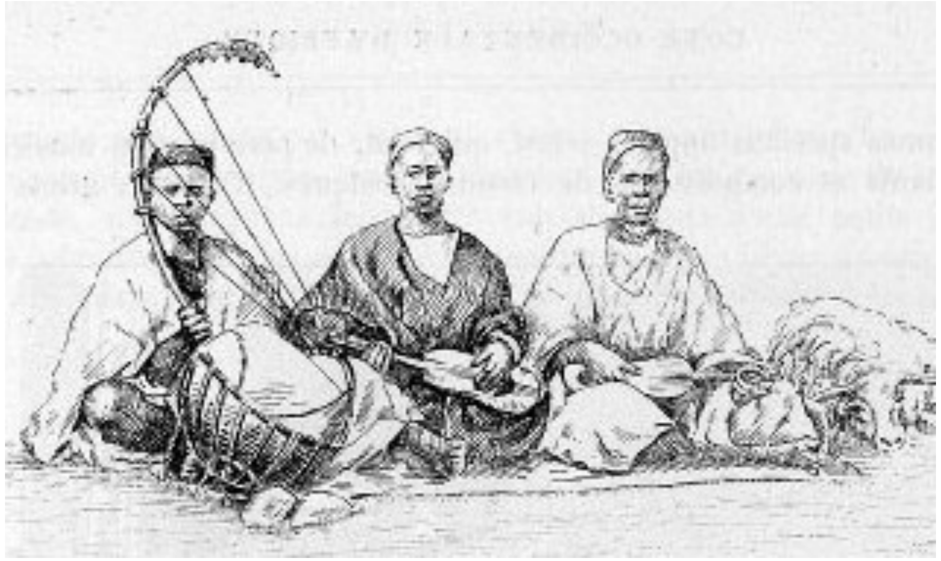

Questions de discussion

- Selon vous, quels sont les avantages et les défis du CPS en tant qu'outil d'apprentissage ?
- Quels sont les avantages et les défis liés à la mise en œuvre du CPS au sein de votre plateforme ?

Etape 1: Collecte des histoires

Exemple du Hub en Africa de l'Est

Histoire 3: L'approche orientation du site complet de TCI crée un impact considérable dans le Centre de santé Mukono IV

Domaine: ?
Titre de l'histoire: L'approche orientation du site complet de TCI crée un impact considérable dans le Centre de santé Mukono IV
Narratrice: Alex Namaala Lwasa, Infirmière, Municipalité de Mukono (Ouganda, Afrique de l'Est)

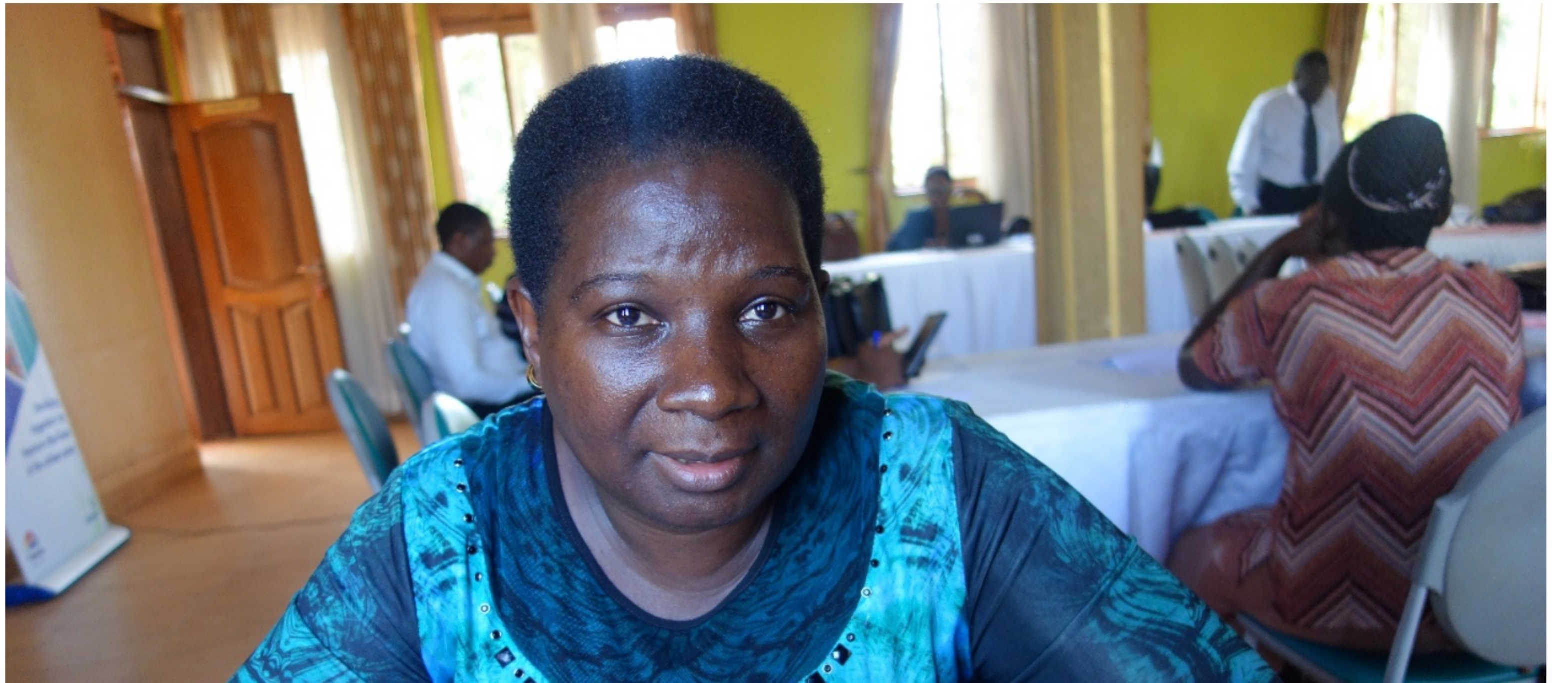

Comment on collecte une histoire relative au CPS !

C'est facile ☺

Collectez les histoires relatives au « changement significatif » (dans les geographies) tous les trimestres.

- En examinant le dernier trimestre, que pensez-vous être le changement le plus significatif dans *[domaine précis du changement]* ?
- Pourquoi ceci vous semble-t-il significatif ?
- Quel impact crée-t-il maintenant ou alors créera-t-il à l'avenir ?

Une histoire relative au CPS…

Est :

- Une interview ou une discussion axée sur des expériences/perspectives personnelles
- Une collecte de données quantitatives
- Une opportunité pour écouter et apprendre
- Racontée à la <u>première personne</u>

N'est pas :

- Une étude de cas
- Un article de blog
- Un article de journal
- Un rapport d'activité
- Une opportunité pour coacher ou pour faire un plaidoyer
- Racontée à la troisième personne

Techniques d’interview

Cliquer pour ajouter un texte

L’art d’interviewer

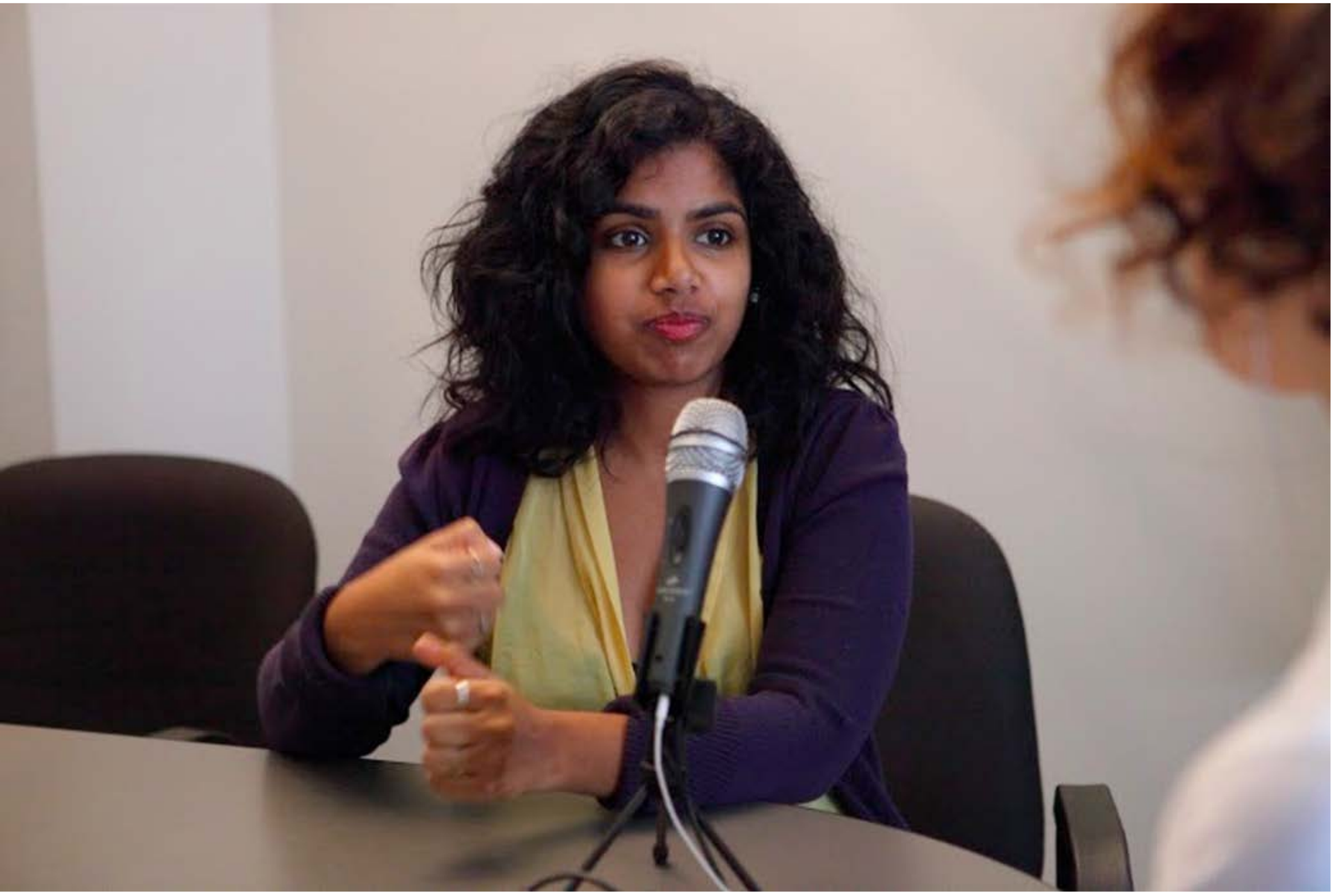

- Astuces pour interviewer
- Aspects techniques
- Défis courants

Mettez-vous à l’aise

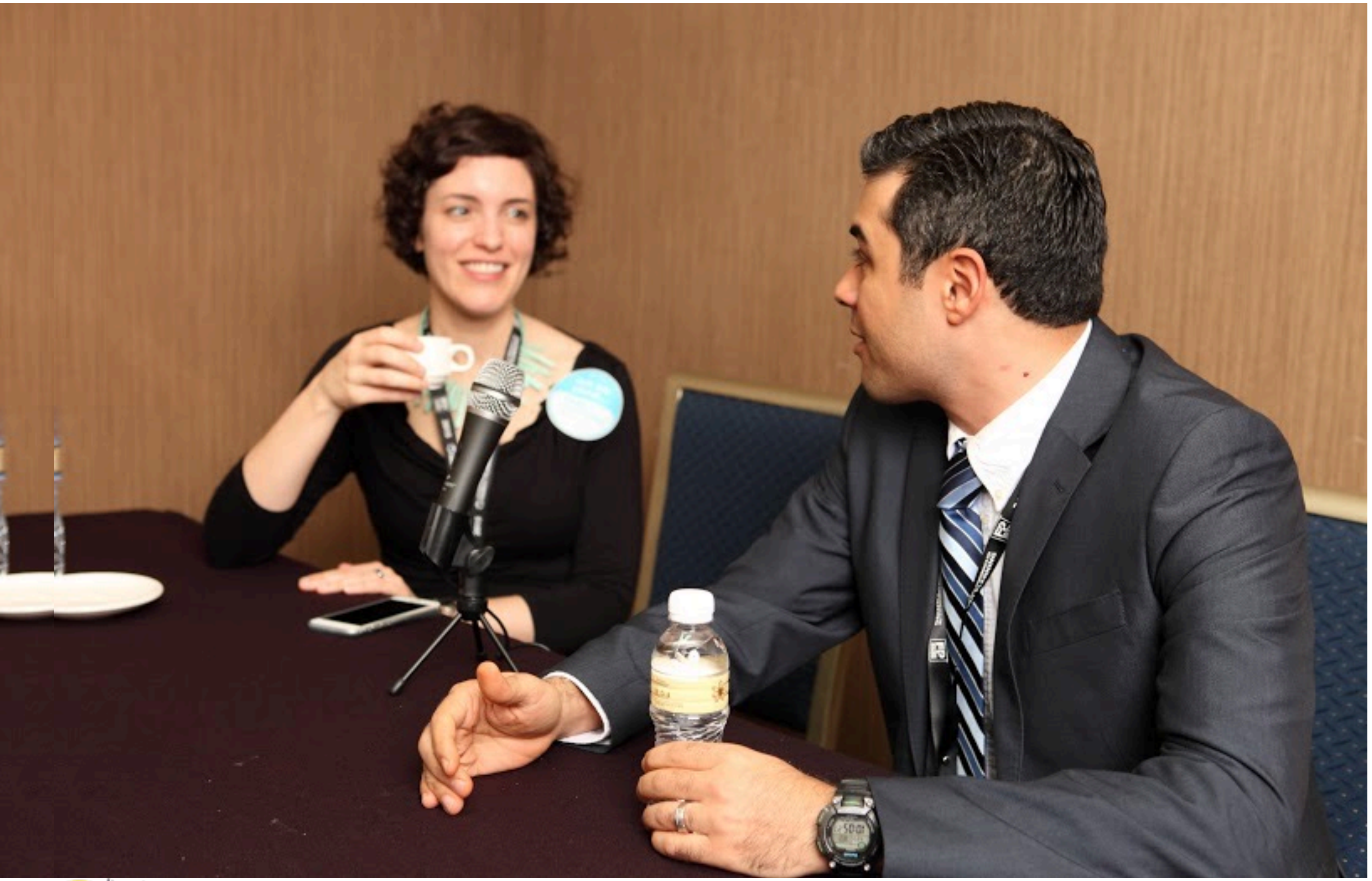

- Le calme
- Le contact visuel
- La sincérité
- Un cadre privé

Préparez-vous, mais soyez flexibles

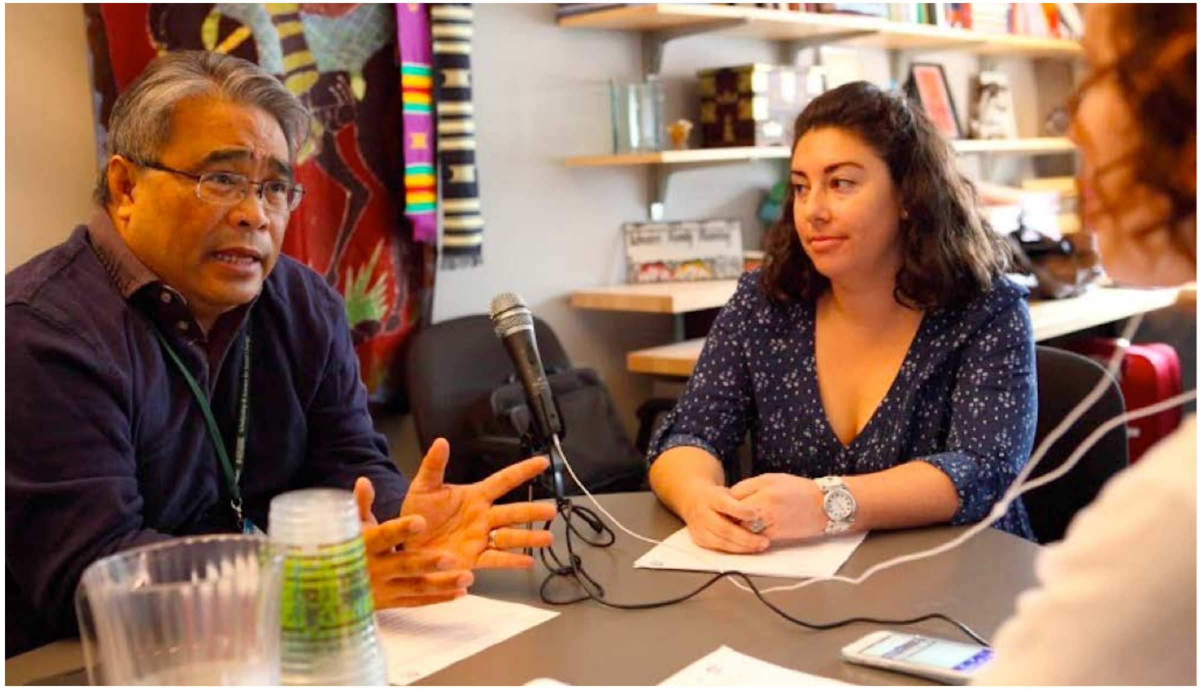

- Préparation
- Ouverture d’esprit et concentration
- Une écoute active !

Une interview en conformité avec les normes de l'éthique : protéger et respecter

- Obtenir un consentement
- Respecter les barrières personnelles et les normes culturelles
- Honnêteté et exactitude

- *Que ferez-vous si le narrateur identifie ou décrit dans son histoire quelqu'un qu'on pourrait facilement reconnaître ?*

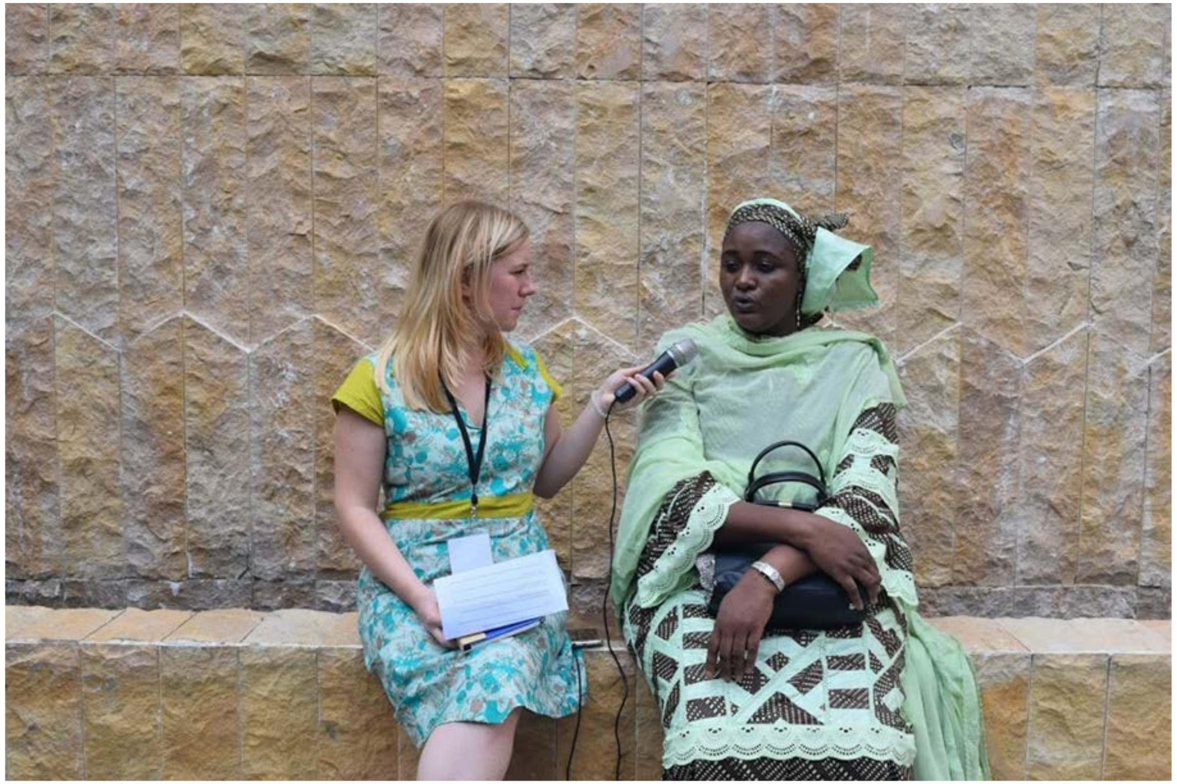

Aspects techniques

<u>Le matériel :</u>

- Téléphone portable
- Microphone à main et micro cravates ou microphones avec trépied
- Des écouteurs à boutons ou éliminateurs de bruit

<u>Les logiciels :</u>

- Voice Record Pro
- Google Drive ou Dropbox

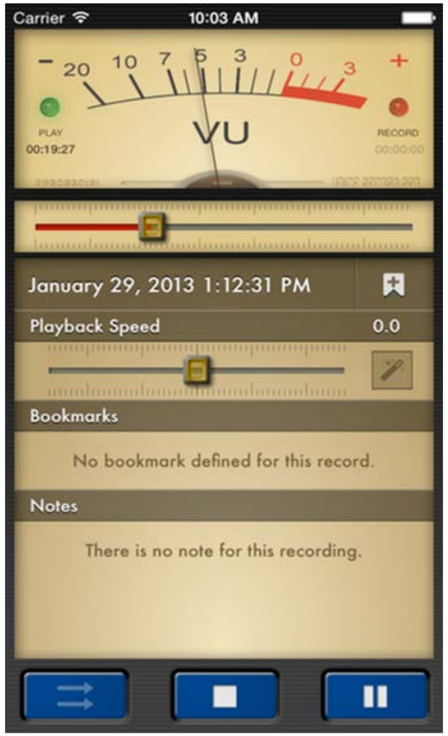

Qualité audio

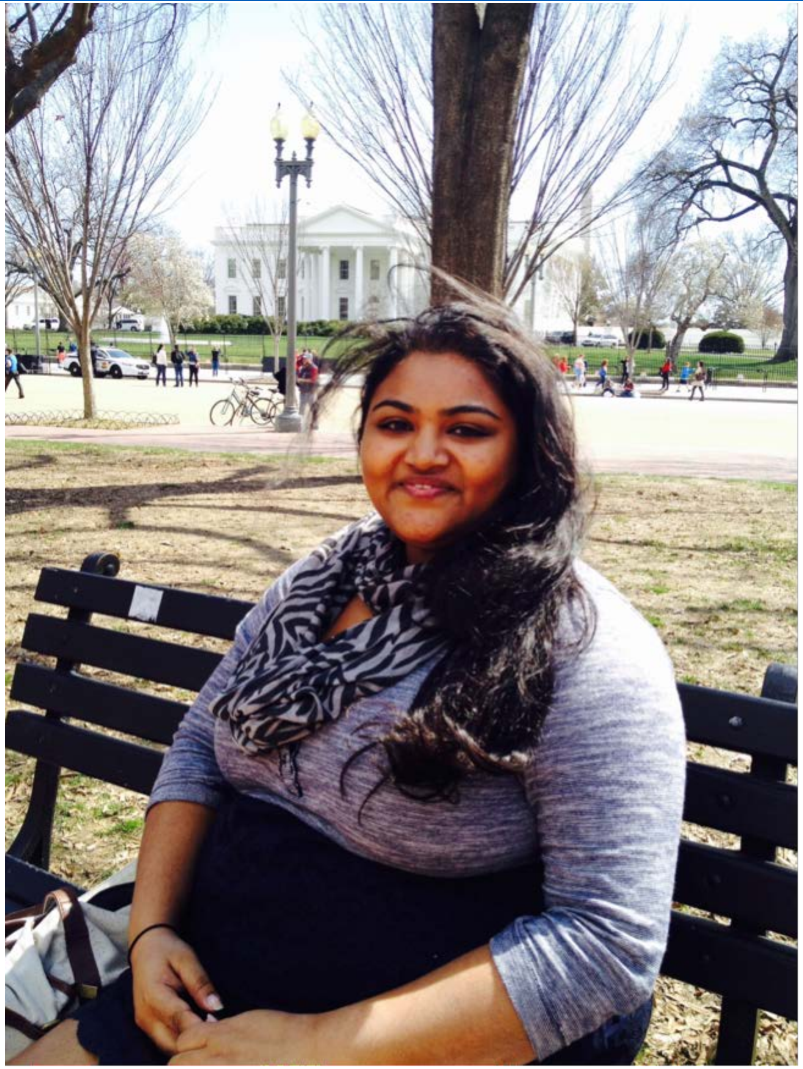

- Le lieu, le lieu, le lieu !
- La nécessité des microphones

Transcription du contenu

tran·scribe

/tran(t)ˈskrīb/

verb

put (thoughts, speech, or data) into written or printed form.
"each interview was taped and transcribed"

- N’apportez aucune complication.
- Des applications ou logiciels peuvent aider.
- Transcrire uniquement les interventions clés.
- Éditer pour embellir et non pour changer de sens.

Commençons!

Passons en revue le guide d'interview !

- Nous allons passer à la phase pratique dans un instant mais avant, passons en revue le guide pour voir comment il est structuré et pour mieux maîtriser son contenu

Que contient le Guide d'interview?

- Rappel sur le contenu d'une histoire et le but de la collecte des histoires CPS
- Conseils utiles lors de la préparation à la collecte d'histoires CPS
- Le guide d'interview fournira:
    1. Introduction or but de l'interview à lire au narrateur
    2. Formulaire de consentement
    3. Les informations sur le narrateur, quand et où l'histoire a eu lieu
    4. Questions d'interview
- Directives relatives à l'enregistrement
- Suivi des histoires
- Conseils en matière de photographie (que nous parcourrons de façons détaillée plus tard)

Du guide d'interview

Une histoire devrait donc comprendre trois parties centrales et devrait prendre fin en indiquant la raison pour laquelle le narrateur la considère comme significative (voir ci-dessous)

Un début	Un milieu	Une fin
Quelle était la situation *avant* que le changement ne se produise? Historique/contexte ex., avant leur participation au projet xxx ou avant qu'ils aient bénéficié d'un appui	Que s'est-il passé ? Nature et type d'appui/de soutien fournis	La situation *après*. Quelle différence cela a-t-il apporté?

\+

Pourquoi cela est-il significatif?
(saisit la perspective du narrateur de l'histoire et la raison pour laquelle il/elle valorise le changement)

Par conséquent, la question initiale CPS – « Au regard du dernier mois/trimestre, quel est selon vous le changement le plus significatif observé suite à la mise en œuvre de TCI ? » - forme la base de l'information que nous recherchons. Des questions supplémentaires d'approfondissement permettent de « capter » l'histoire dans sa totalité.

Un bon intervieweur = un bon chercheur

Si nécessaire, essayez une version modifiée du CPS et reformulez les questions de cette manière :

- Comment avez-vous été impliqué dans le projet ?
- Quels sont les changements significatifs que vous avez expérimenté grâce à ce projet ?
- Quels problèmes sont survenus dans la communauté à cause de ce projet ?
- De quels problèmes s'agissait-il ?

** Il peut s'avérer utile de demander à la personne interrogée quand elle a noté le changement pour la première fois, car ce changement n'est pas toujours lié à un événement ou une activité

Astuces de rédaction

- **Qui** est le narrateur ? Décrire sa profession, âge, arrière-plan (compléter les questions relatives au contexte démographique)
- **Quel** a été le changement suite à la mise en œuvre de TCI?
- **Quand** le changement s'est-il produit?
- **Où** le changement a-t-il eu lieu? (Commune ou plus spécifiquement lors d'une sensibilisation, d'un rendez-vous dans un centre, etc.)
- **Pourquoi** le changement est-il significatif du point de vue du narrateur? Quel pourrait en être l'impact au-delà du narrateur?
- **Comment** le changement s'est-il produit?

Conseils pour réaliser les interviews relatives au CPS

- Saisissez toujours le Qui, Quoi, Quand, Où, Pourquoi ...et Comment
- Menez des interviews afin de limiter les suppositions
- Lorsque vous aurez terminé, chaque histoire tiendra sur une ou deux pages
- Une histoire = ensemble des questions collectives concernant chaque domaine du changement

Jeu de rôle !

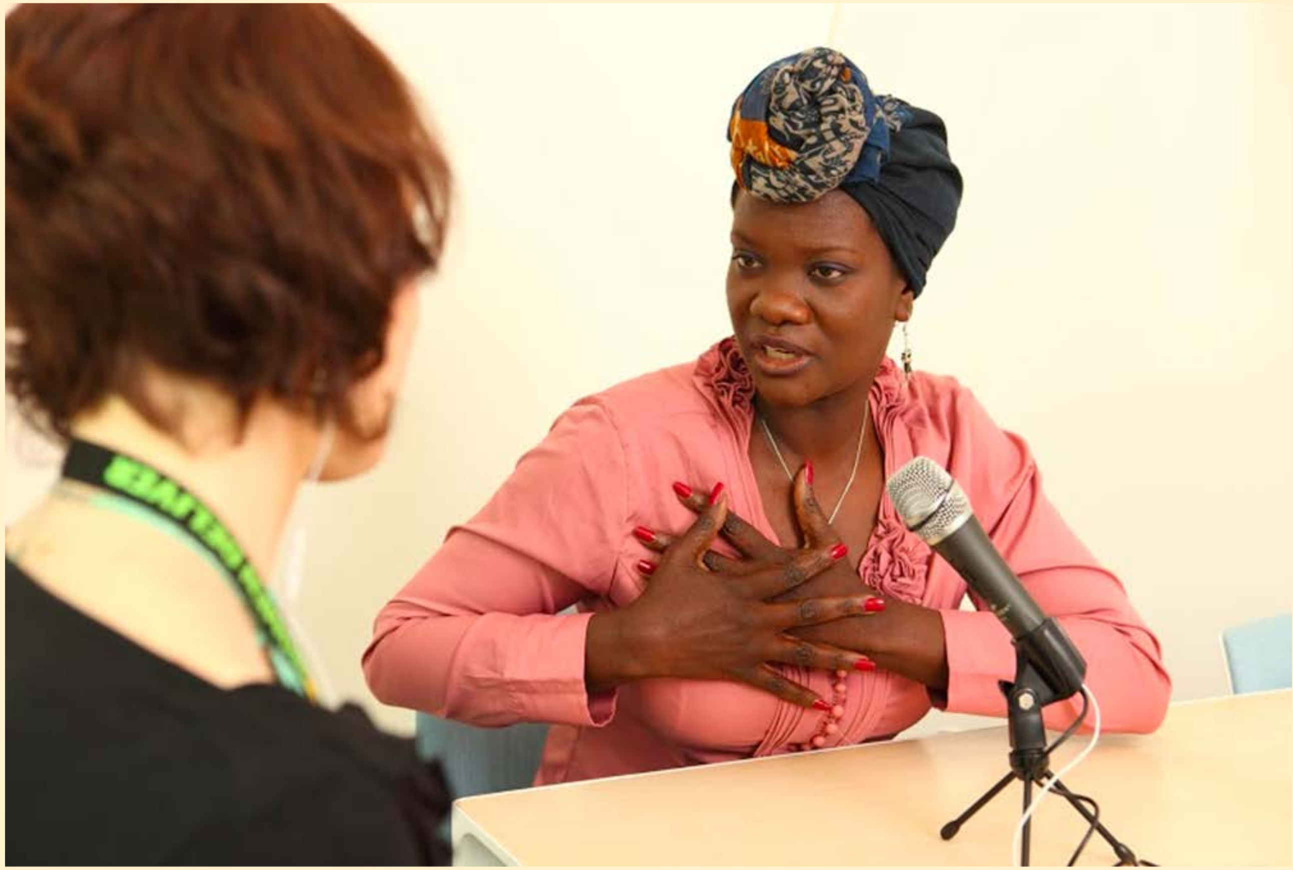

- Se mettre par groupes de deux
- Chaque paire doit se numéroter de 1 à 4
- Tour à tour, jouez le rôle de l'intervieweur et du narrateur
- Tout le monde joue l'introduction, le consentement, etc.
- Enregistrez-vous les uns les autres
- Le numéro de votre groupe constitue le domaine sur lequel vous poserez les questions et UNIQUEMENT les questions comprises dans ce domaine
- Ecoutez l'interview et relevez une phrase qui selon vous souligne l'importance de l'histoire qui vous a été contée

Comment s'est passé l'interview ?

- Les questions avaient-elles un sens ?
- Pensez-vous avoir écouté une bonne histoire ?
- Quelle est la meilleure partie de l'histoire ?
- Quelle a été la partie la plus difficile ?
- Que faites-vous si la question ne « cadre » pas ou ne passe pas avec le narrateur ?
- Avez-vous du changer la vocabulaire?
- Avez-vous des questions ?

Défis potentiels

Et s’il « n’y a aucun changement » ?

- Le changement se produit à tout moment, donc il est erroné de dire que rien n’a changé.
- Tous les changements ne sont pas majeurs et absolus, ainsi soyons attentifs
- Demandez à la personne interrogée d’identifier tous les changements qui se sont produits et de choisir les plus significatifs.

Que faire en cas de résistance ?

Vous interviewez un réalisateur de projet sur le terrain et ce dernier a un niveau supérieur.

Toutefois, il reste très formel et il est déterminé à camper sur ses positions. Il semble ne pas vouloir s’ouvrir, partager ses perspectives personnelles, ou raconter son histoire.

Comment décrochez-vous une histoire ?

Et si quelqu'un veut imposer son programme ?

Vous interviewez quelqu'un qui évoque une perspective importante pour la mise en œuvre du TCI

Toutefois, au lieu d'ouvrir le dialogue, de répondre à vos questions et de se focaliser sur l'objectif de l'initiative, cette personne se livre à un monologue concernant une question sensible et sans rapport avec l'initiative.

Que faites-vous ?

Etre témoin de l’histoire

Conseils relatifs à la photographie et techniques de narration visuelle d’histoires

Rôle de la Photographie

- Promouvoir la mission
- Documenter les questions locales
- Le contexte est important pour retenir l’attention de l’audience
- Rappeler aux bailleurs de fonds/défenseurs la valeur, l’avantage ainsi que la portée

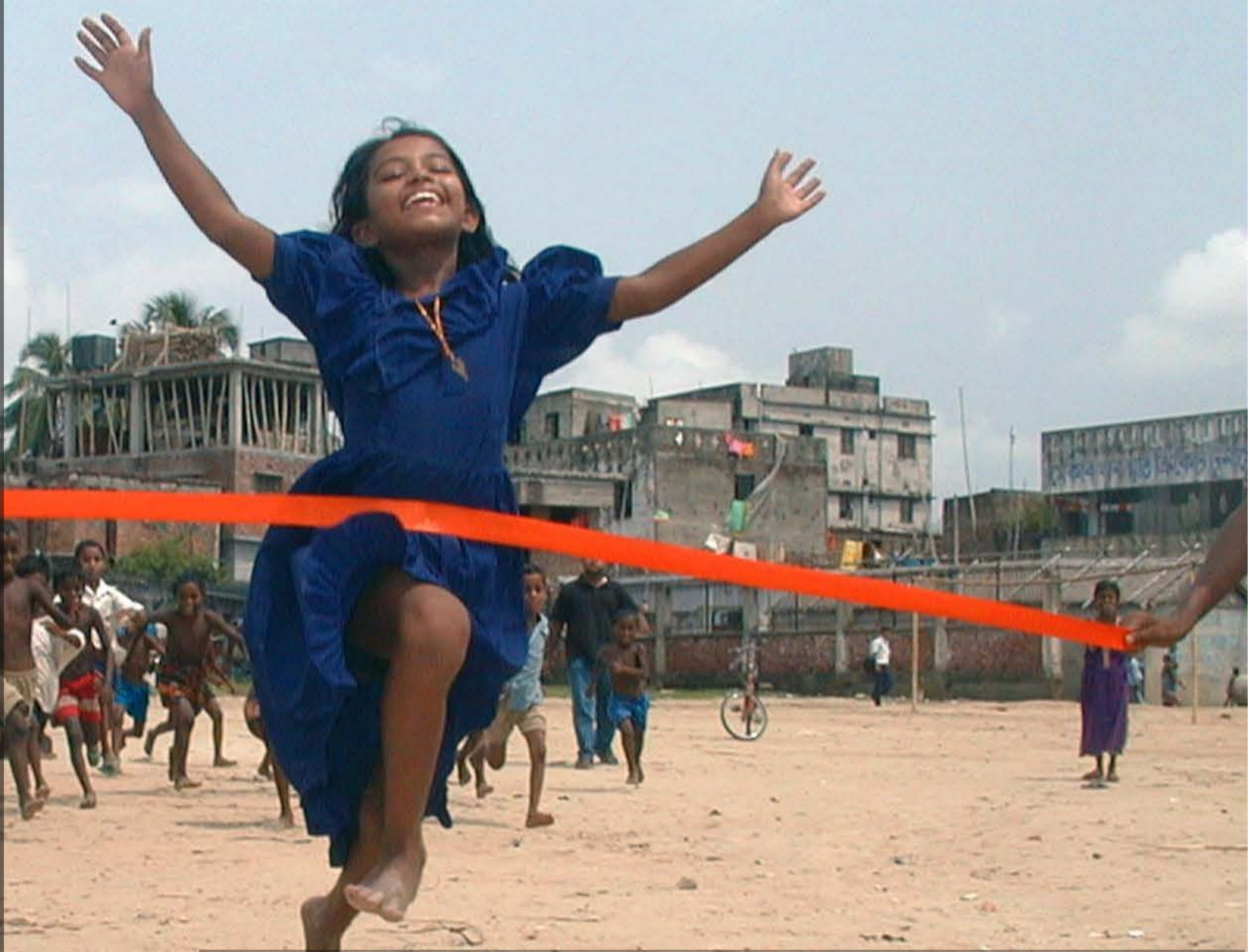

Reshma Akhter, âgée de 8 ans et issue des bas-quartiers de Rayerbazar de la ville de Dhaka au Bangladesh franchit la ligne d’arrivée lors d’une course © 2004 Syed Ziaul Habib Roobon, Reproduction autorisée par Photoshare

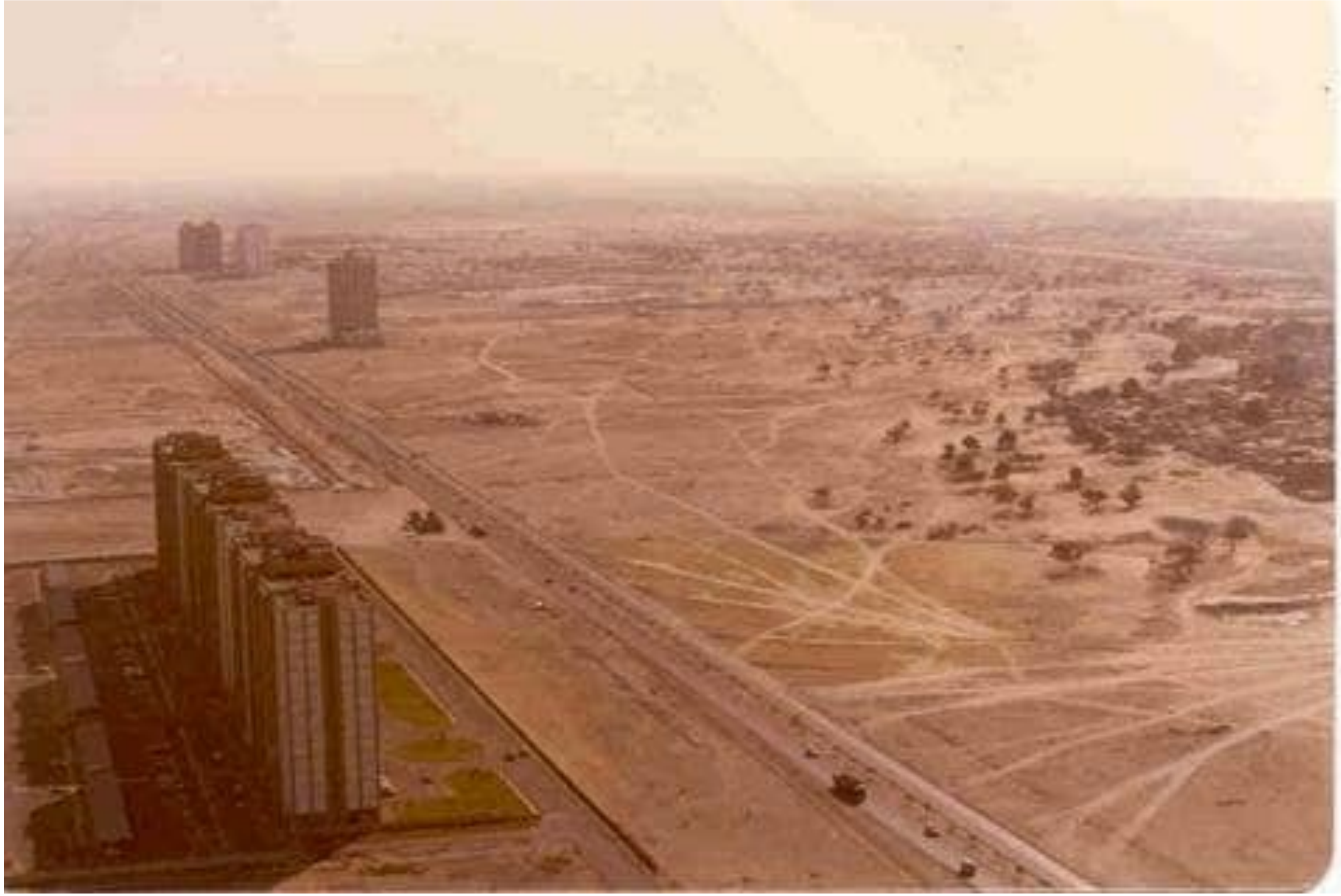

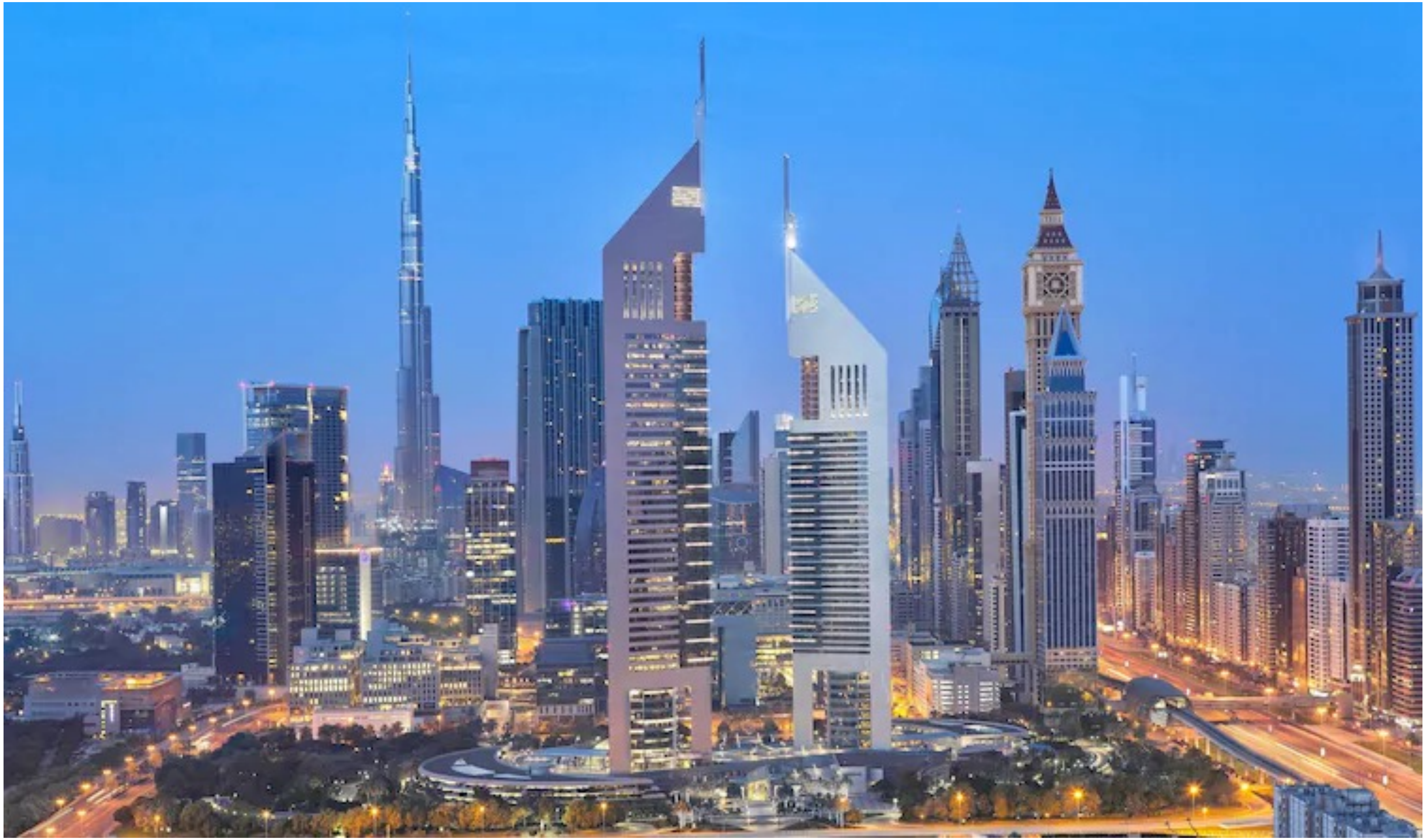

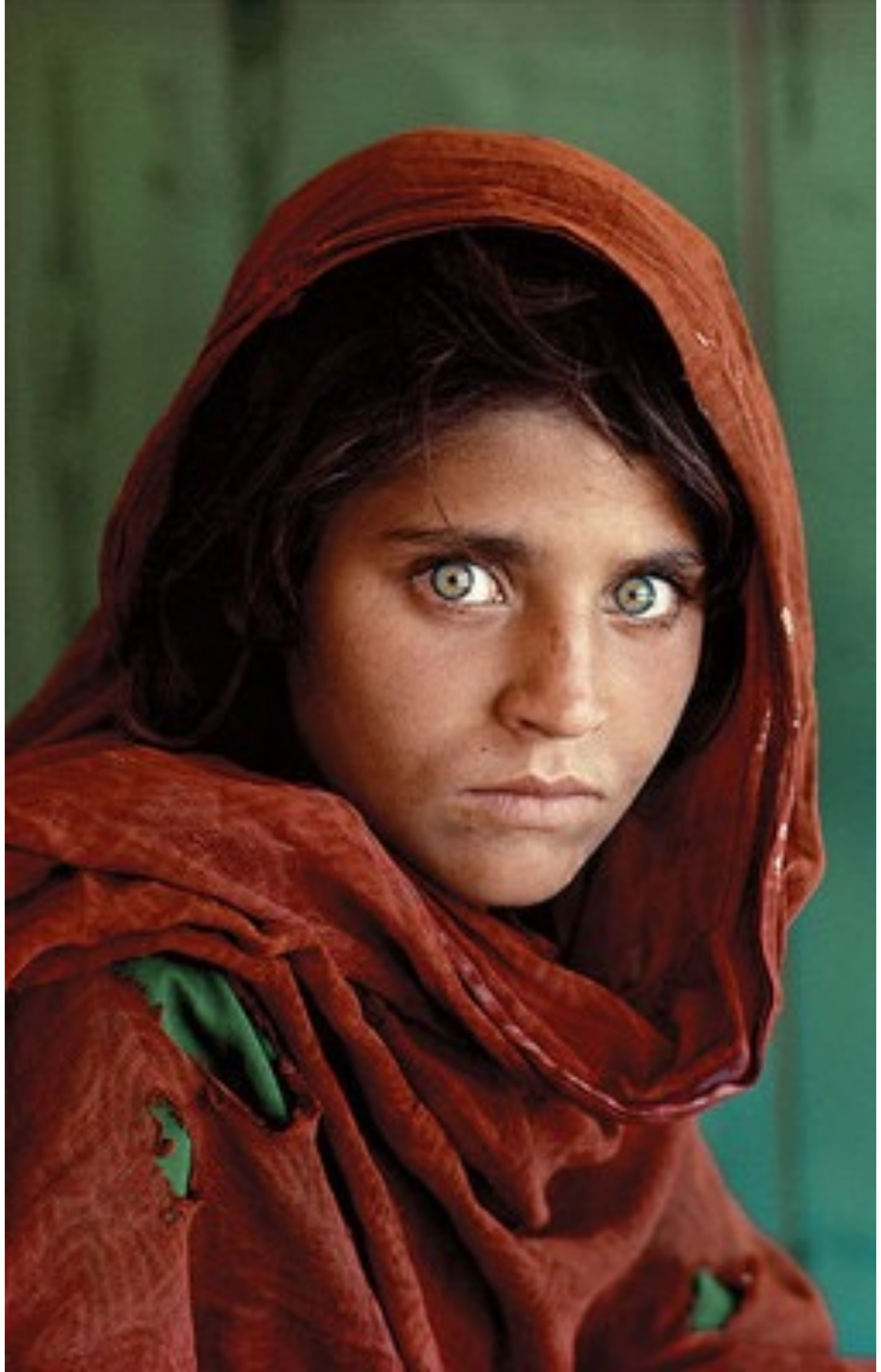

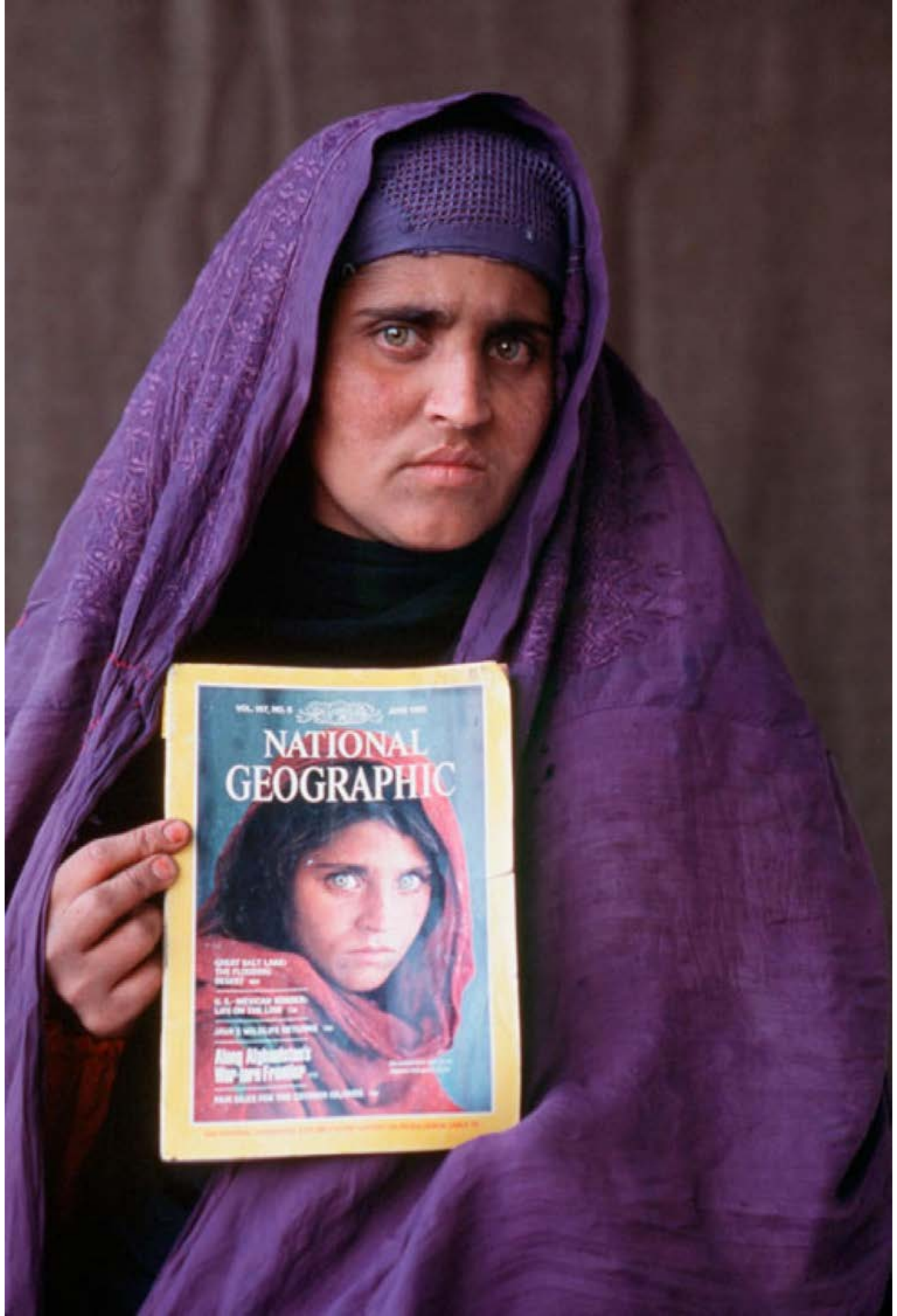
NATIONAL
GEOGRAPHIC

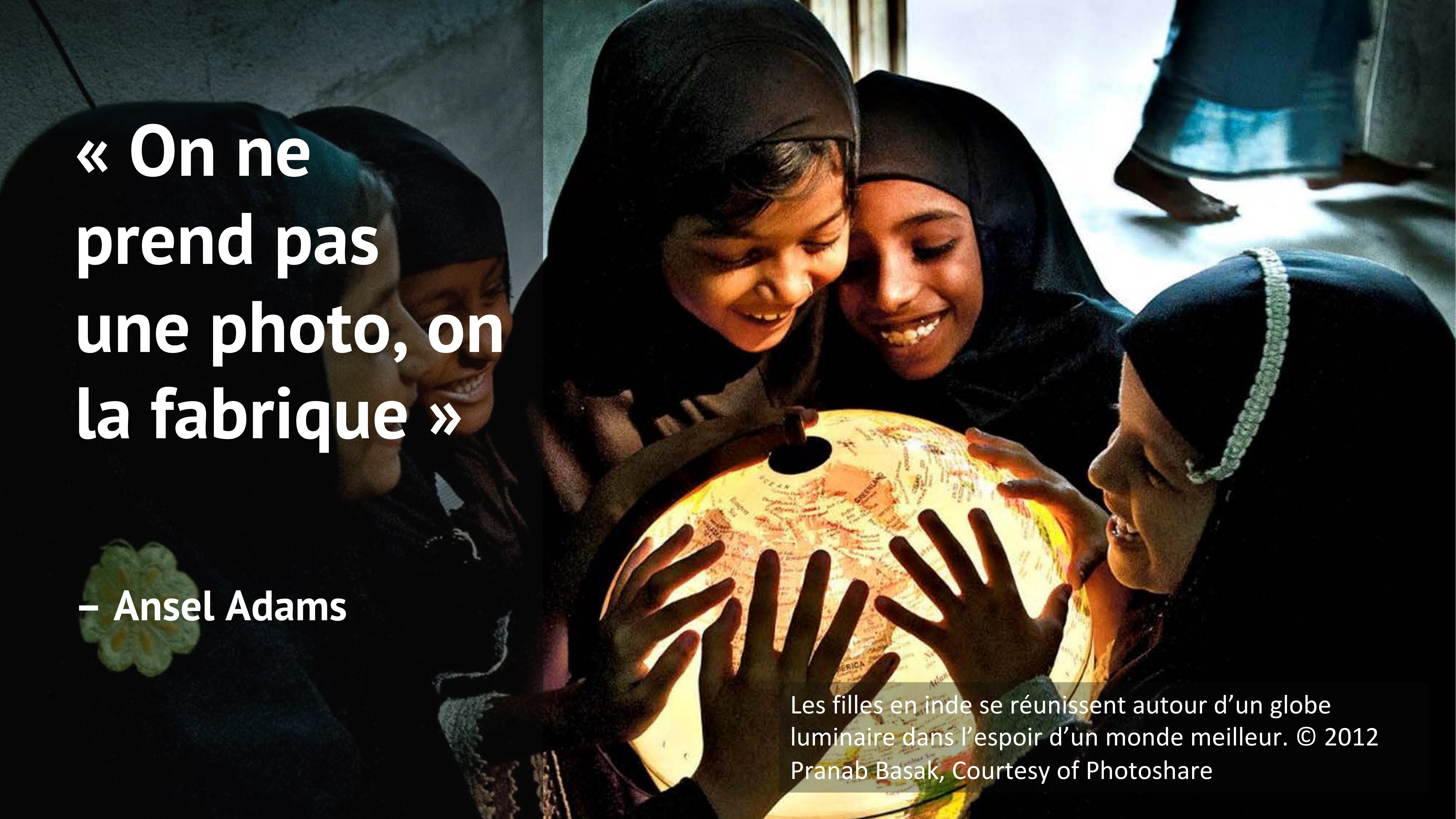

« On ne prend pas une photo, on la fabrique »

– Ansel Adams

Les filles en inde se réunissent autour d’un globe luminaire dans l’espoir d’un monde meilleur. © 2012 Pranab Basak, Courtesy of Photoshare

Réaliser une action photographique

- Répondre à la lumière
- Explorer la composition
- Utiliser la profondeur du champ pour capte l'attention

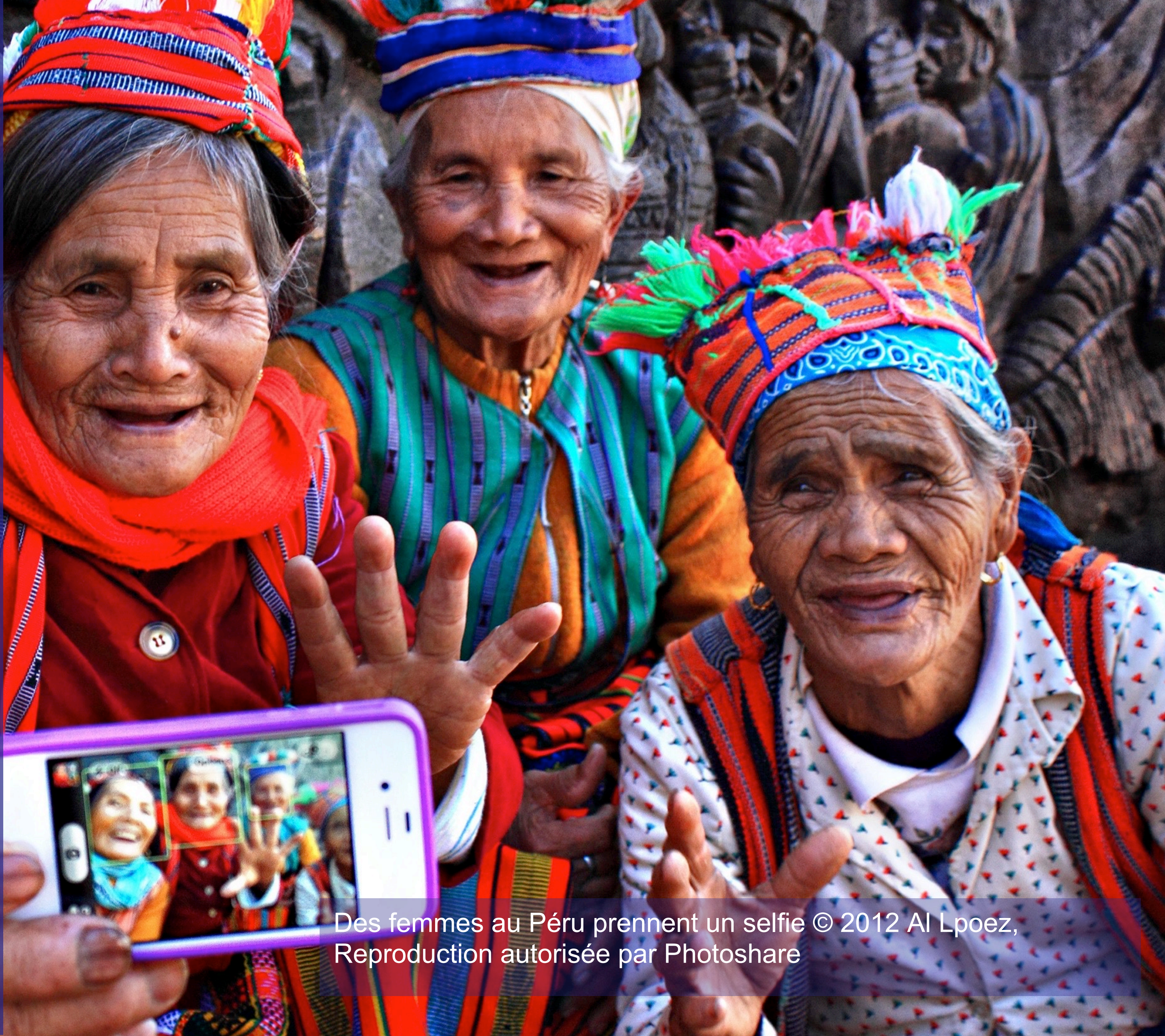

Des femmes au Péru prennent un selfie © 2012 Al Lpoez, Reproduction autorisée par Photoshare

Où est la lumière?

- Éviter le milieu de la journée
- Flash de remplissage, réflecteur, nuance
- couvert– Pas d’ombres fortes

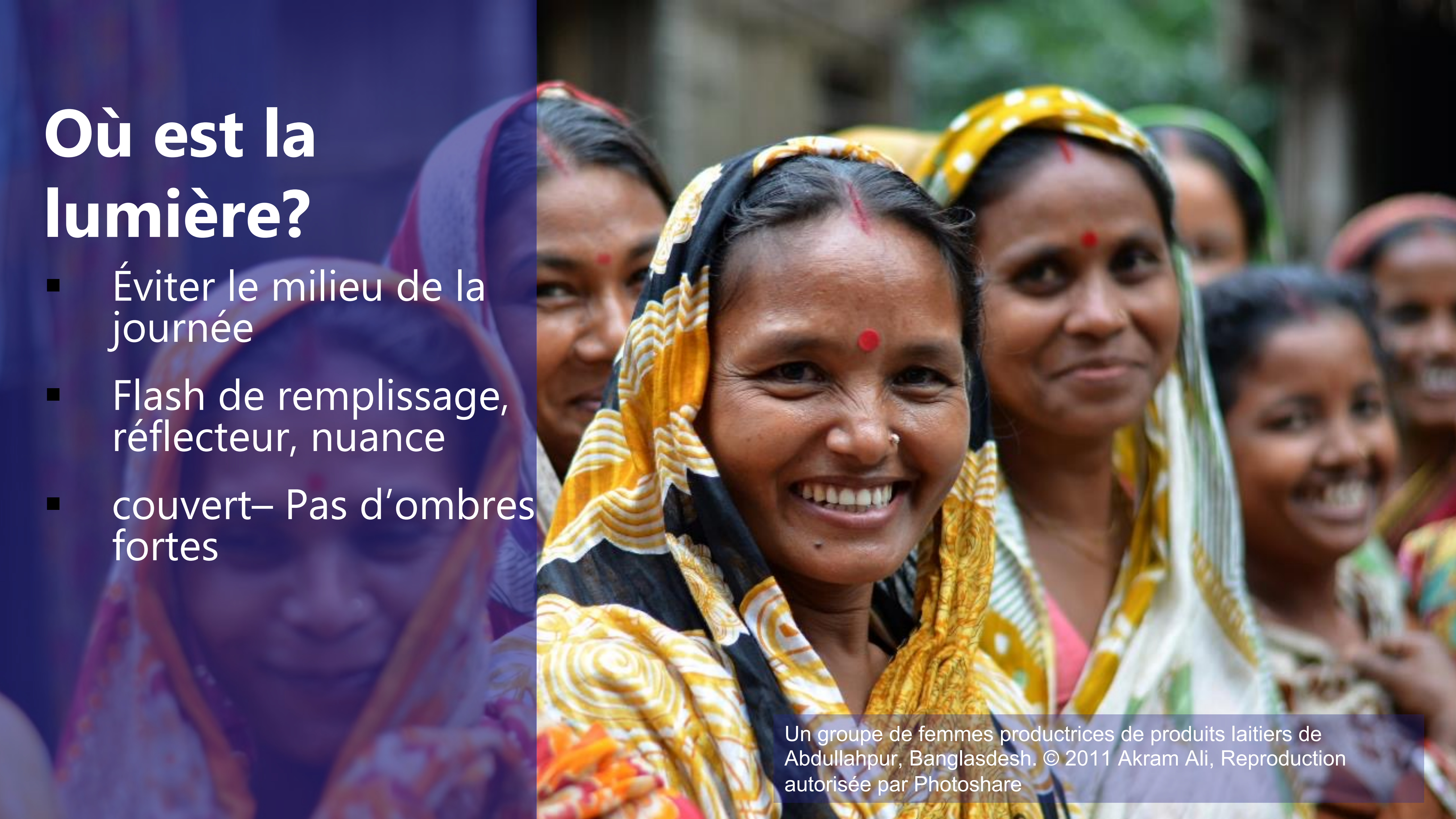

Un groupe de femmes productrices de produits laitiers de Abdullahpur, Banglasdesh. © 2011 Akram Ali, Reproduction autorisée par Photoshare

Contrôler la lumière

- Protéger les populations sensibles
- Un tableau moyen avec de la lumière

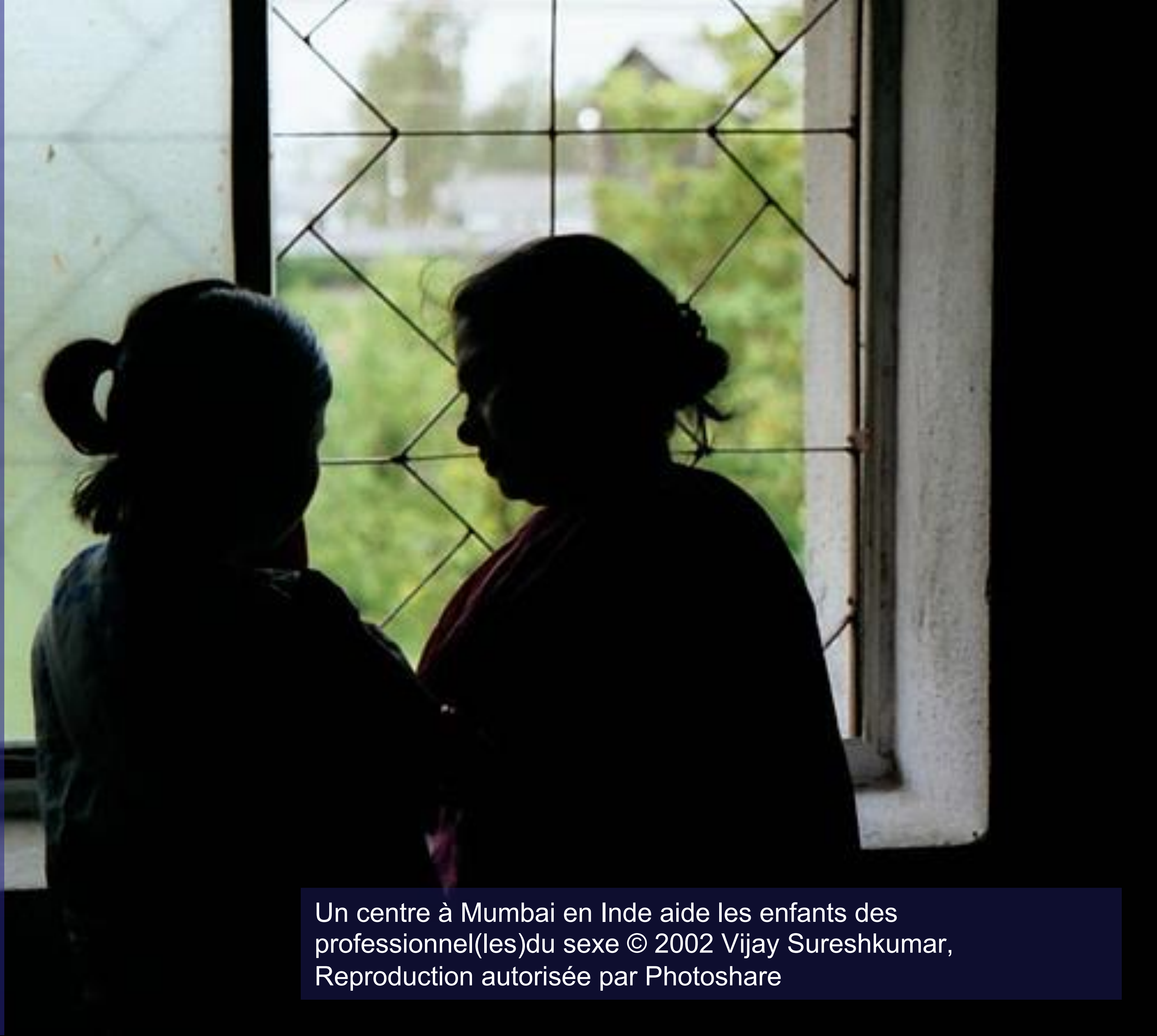

Un centre à Mumbai en Inde aide les enfants des professionnel(les)du sexe © 2002 Vijay Sureshkumar, Reproduction autorisée par Photoshare

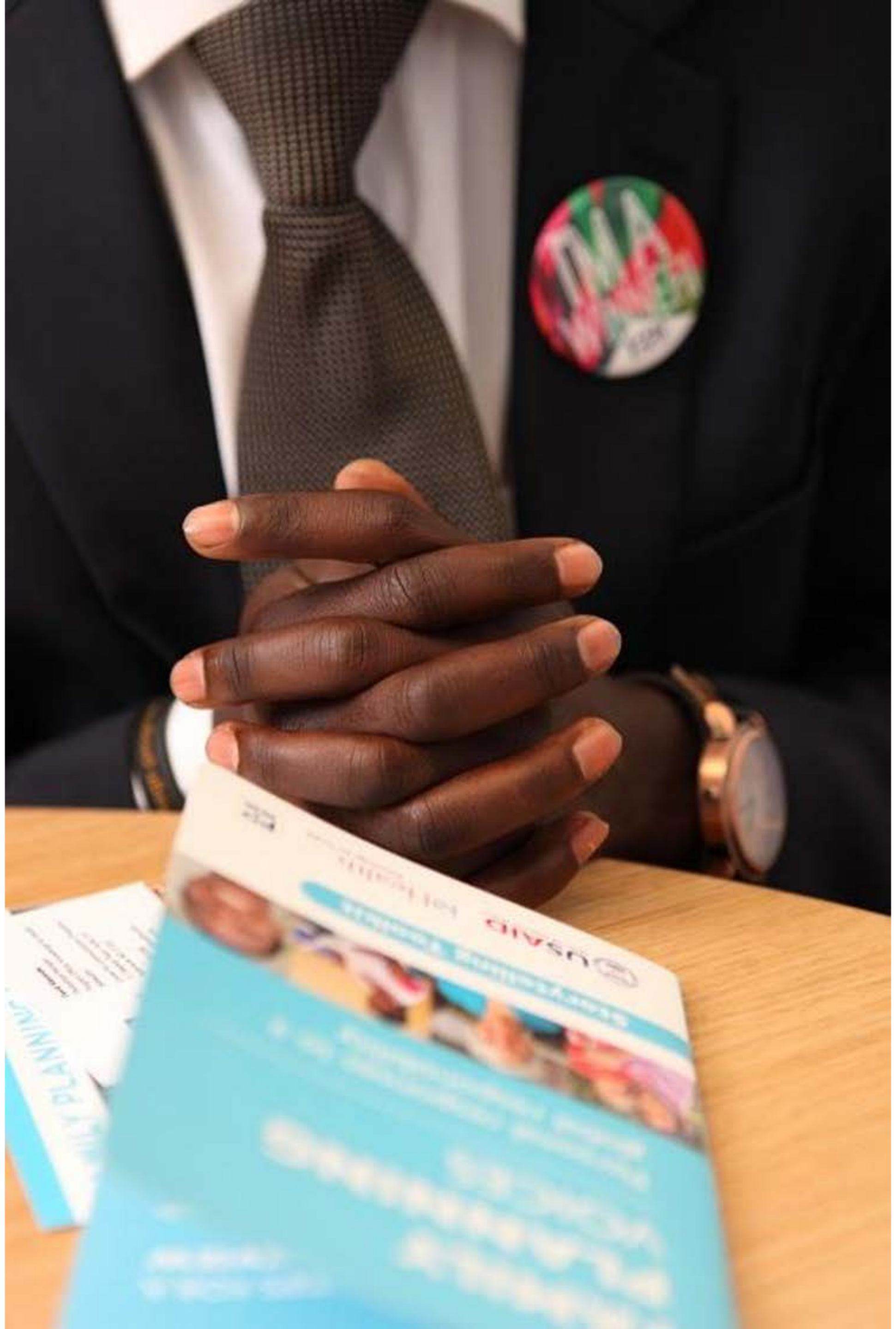

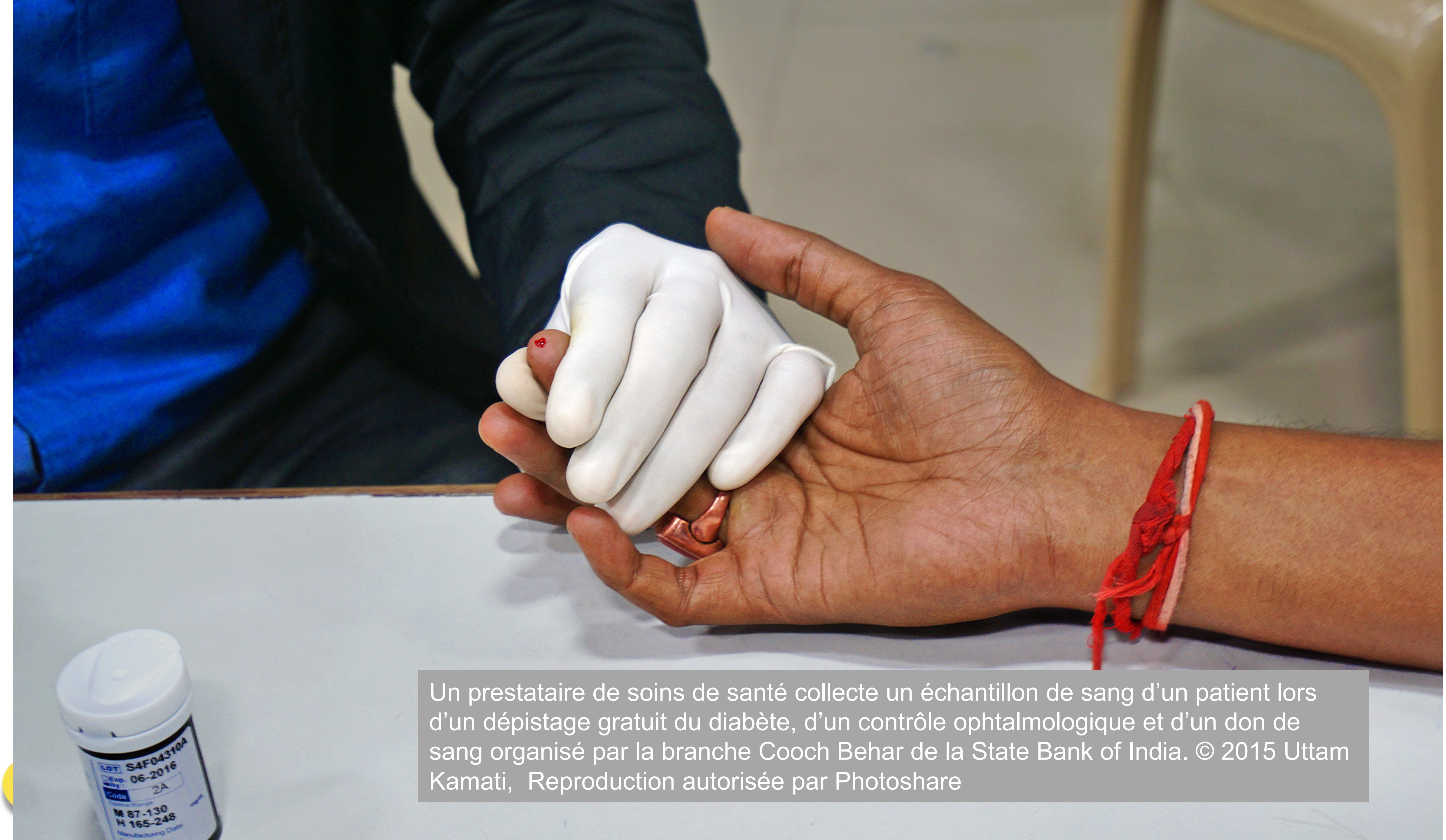

Un prestataire de soins de santé collecte un échantillon de sang d’un patient lors d’un dépistage gratuit du diabète, d’un contrôle ophtalmologique et d’un don de sang organisé par la branche Cooch Behar de la State Bank of India. © 2015 Uttam Kamati, Reproduction autorisée par Photoshare

Composition

- vue/action dynamique
- trouver un point inférieur et élevé ou un angle unique

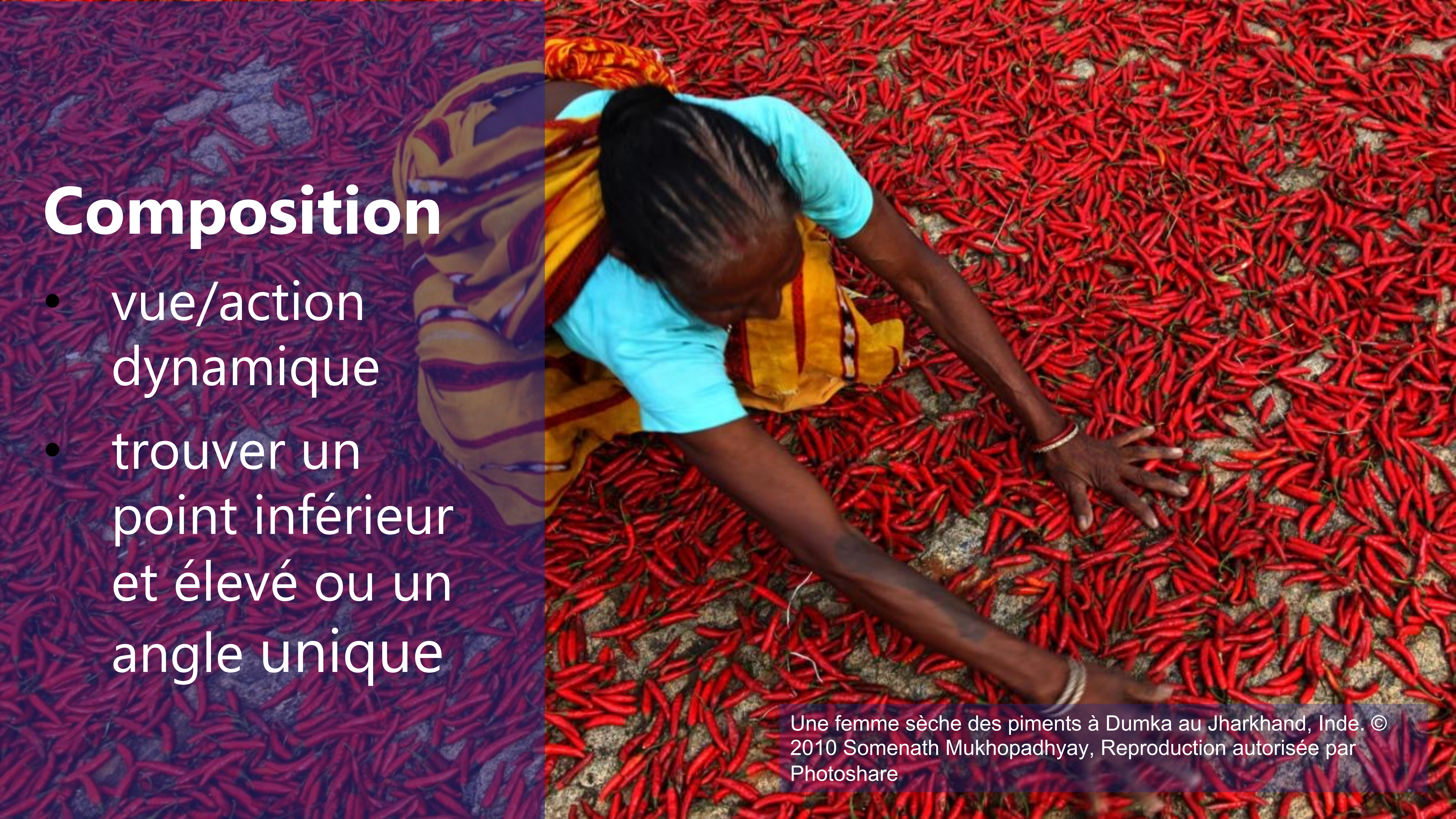

Une femme sèche des piments à Dumka au Jharkhand, Inde. © 2010 Somenath Mukhopadhyay, Reproduction autorisée par Photoshare

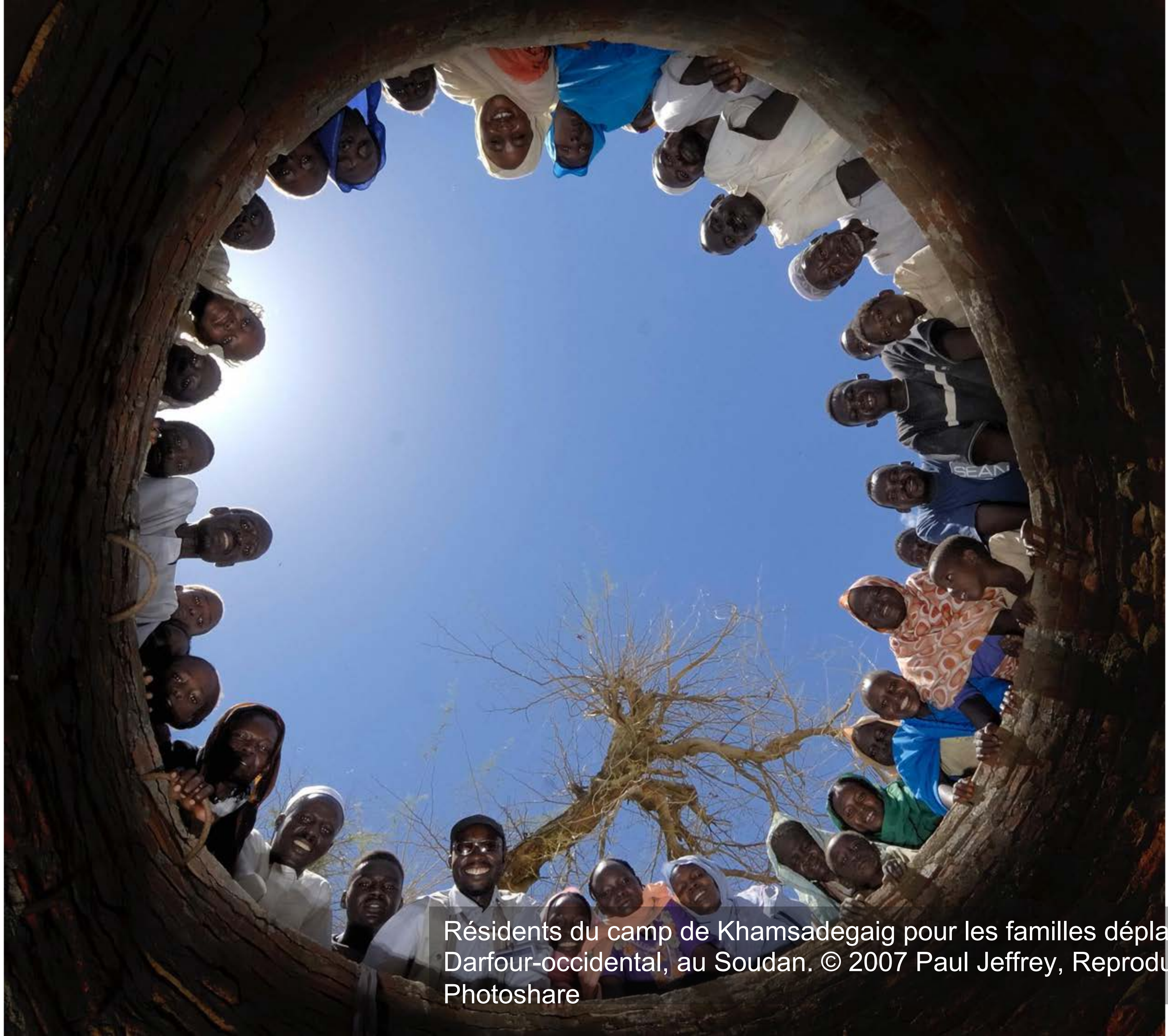

Résidents du camp de Khamsadegaig pour les familles déplacées internes au Darfour-occidental, au Soudan. © 2007 Paul Jeffrey, Reproduction autorisée par Photoshare

Hauteur des yeux

- Déterminer le niveau des yeux
- Éviter les distorsions
- Mettre l'accent sur le sujet

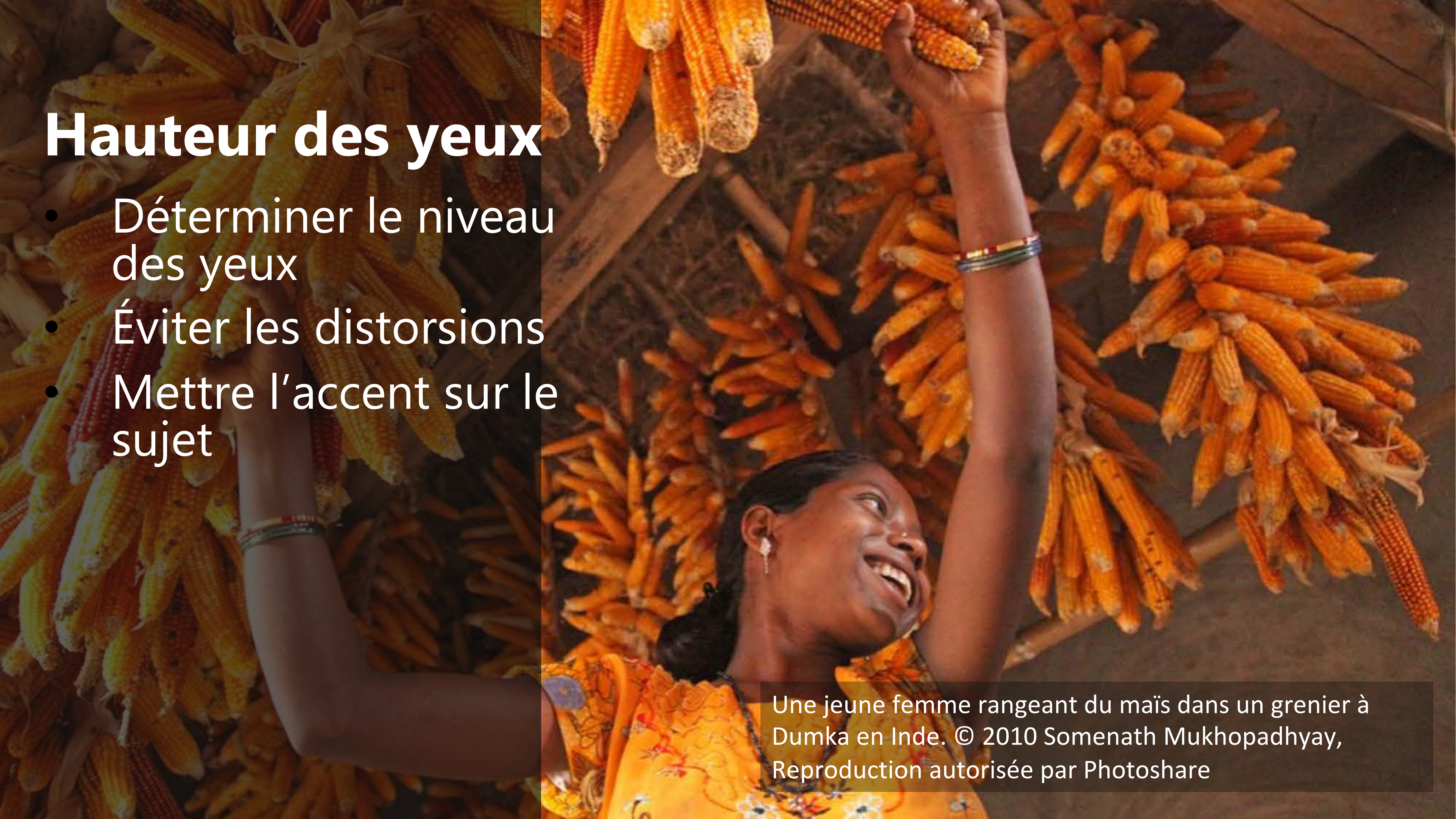

Une jeune femme rangeant du maïs dans un grenier à Dumka en Inde. © 2010 Somenath Mukhopadhyay, Reproduction autorisée par Photoshare

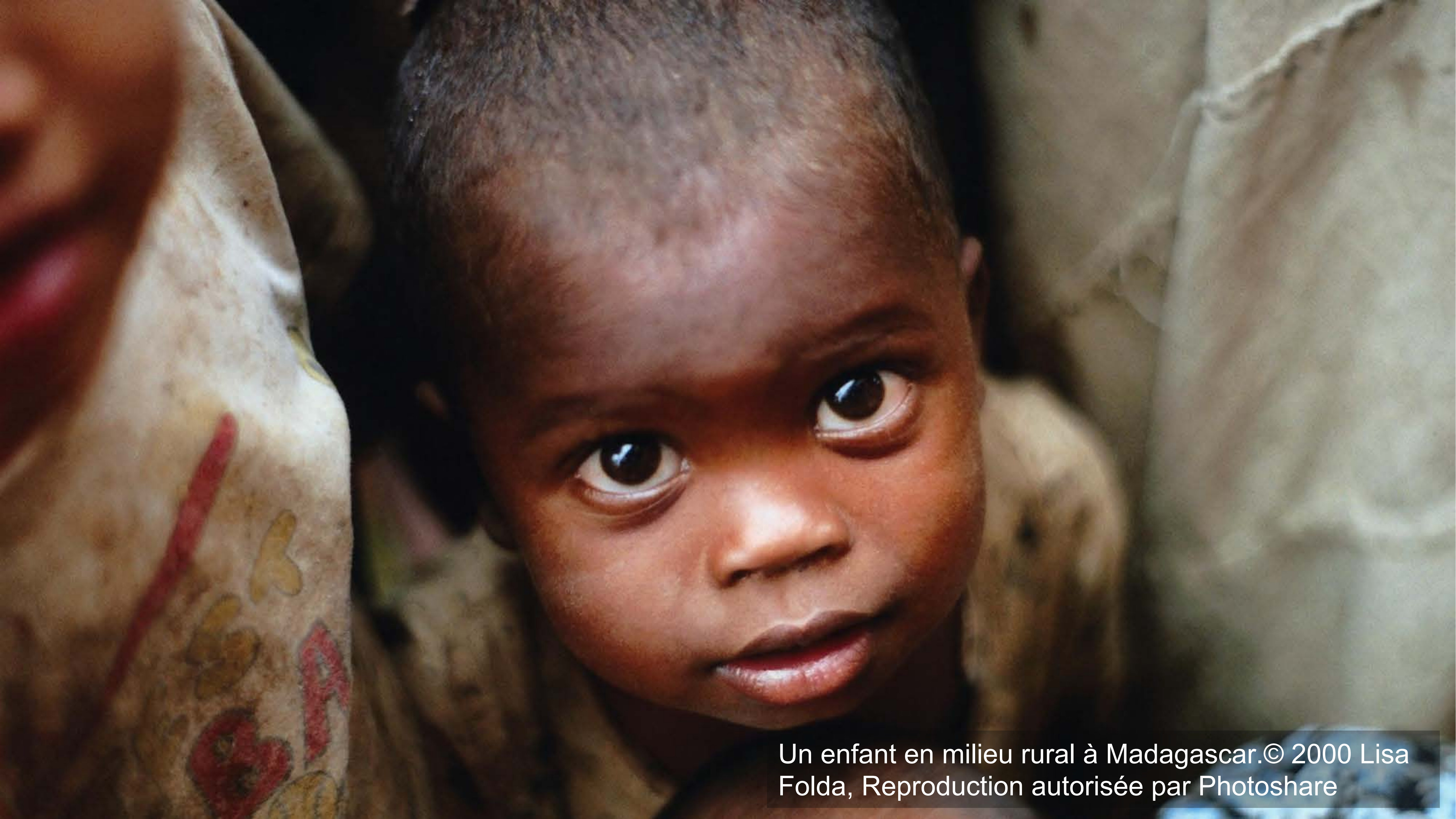

Un enfant en milieu rural à Madagascar.© 2000 Lisa Folda, Reproduction autorisée par Photoshare

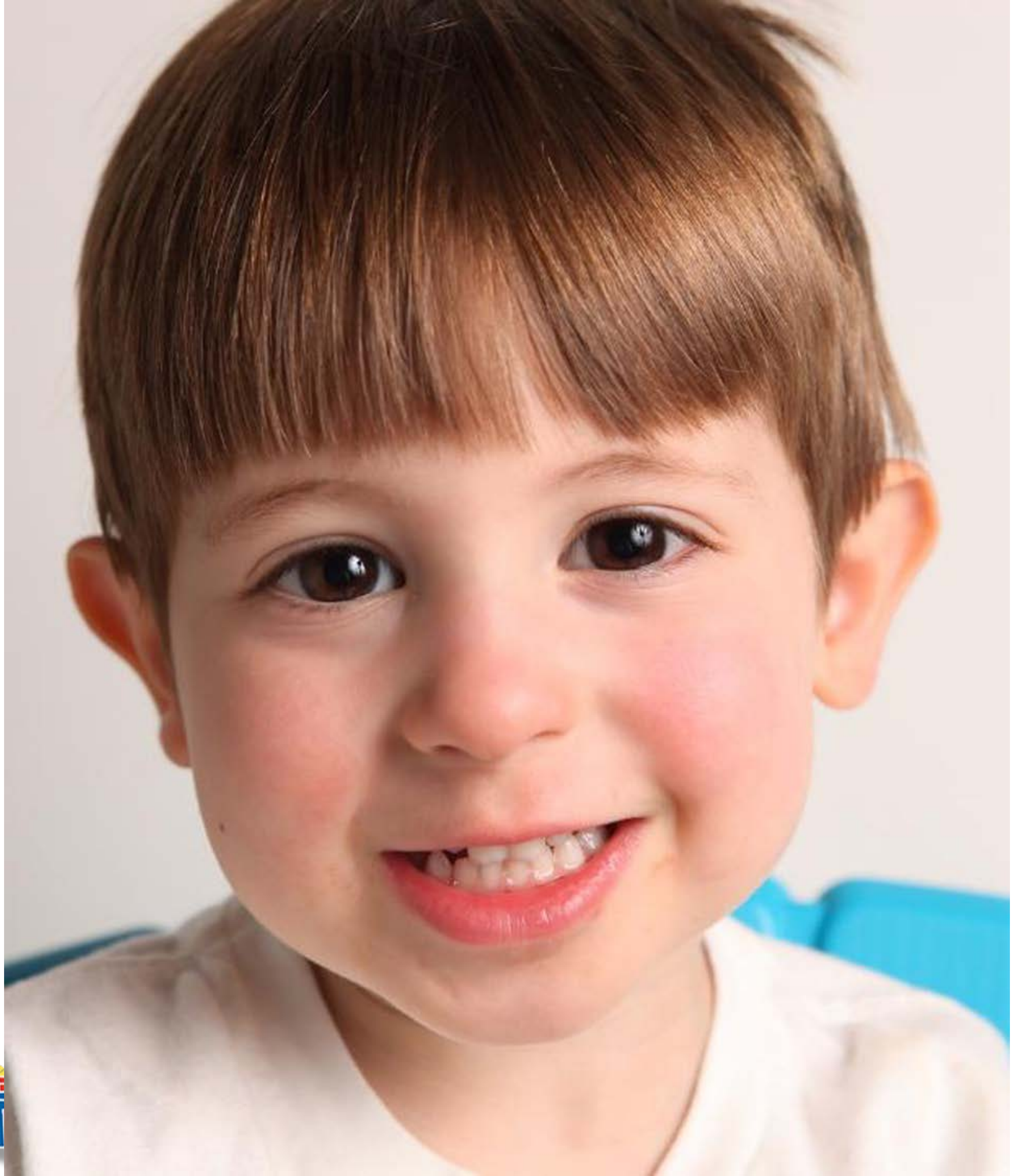

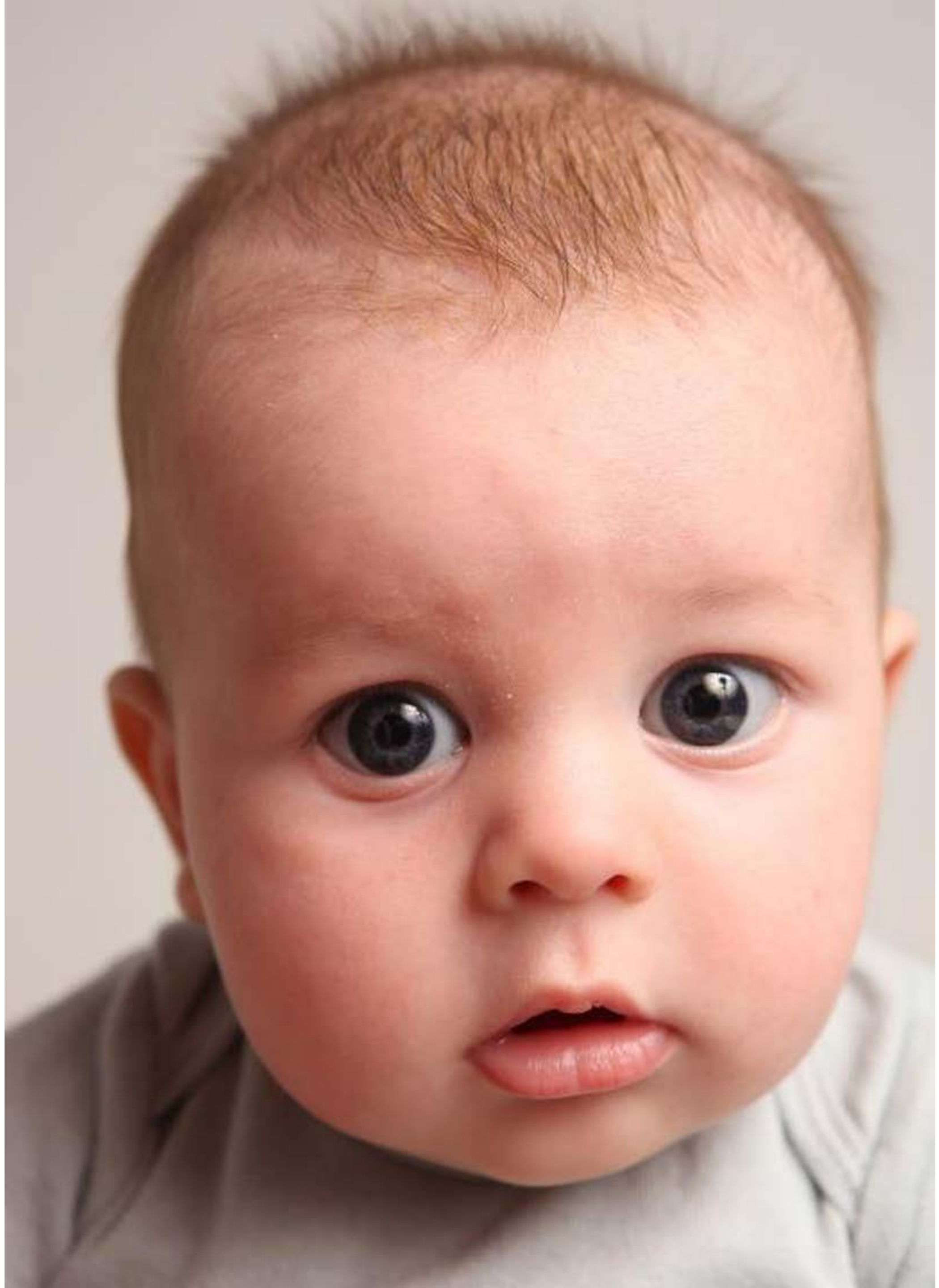

Se rapprocher

- Remplir le cadre
- Créer des connections et susciter l’adhésion

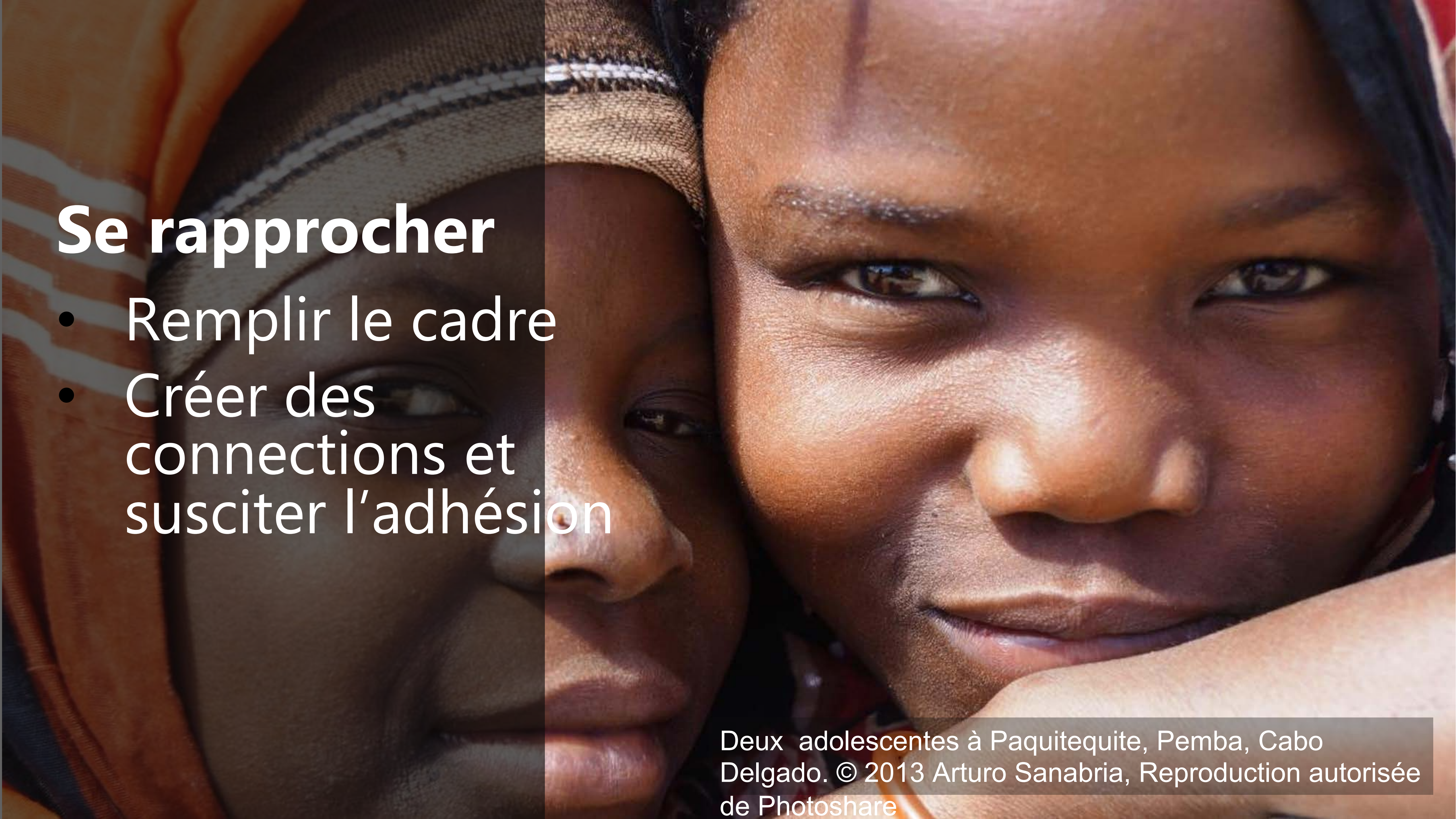

Deux adolescentes à Paquitequite, Pemba, Cabo Delgado. © 2013 Arturo Sanabria, Reproduction autorisée de Photoshare

Règle des tiers

- Les images sont plus captivantes lorsqu’elles sont placées sur une grille imaginaire

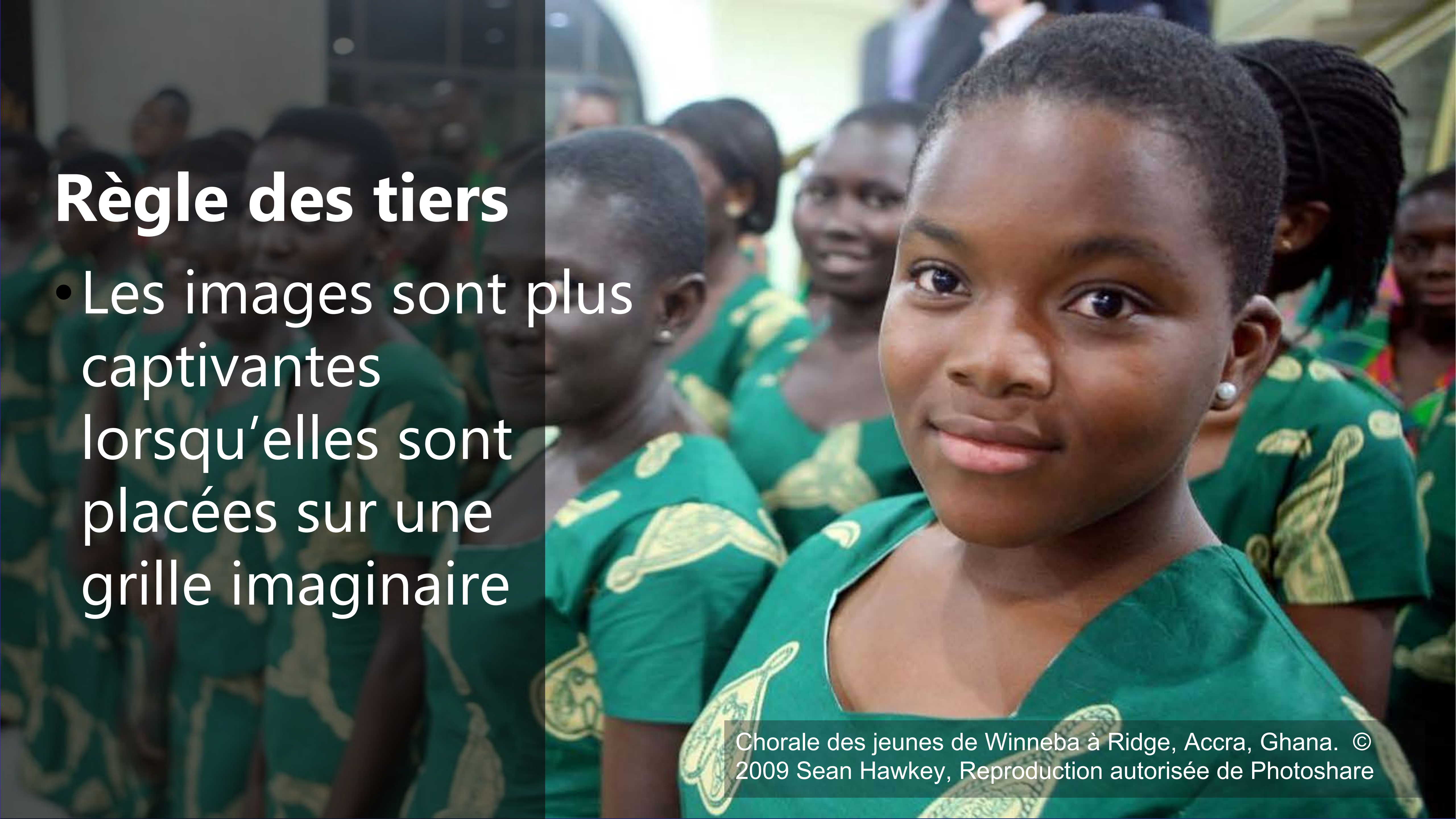

Chorale des jeunes de Winneba à Ridge, Accra, Ghana. © 2009 Sean Hawkey, Reproduction autorisée de Photoshare

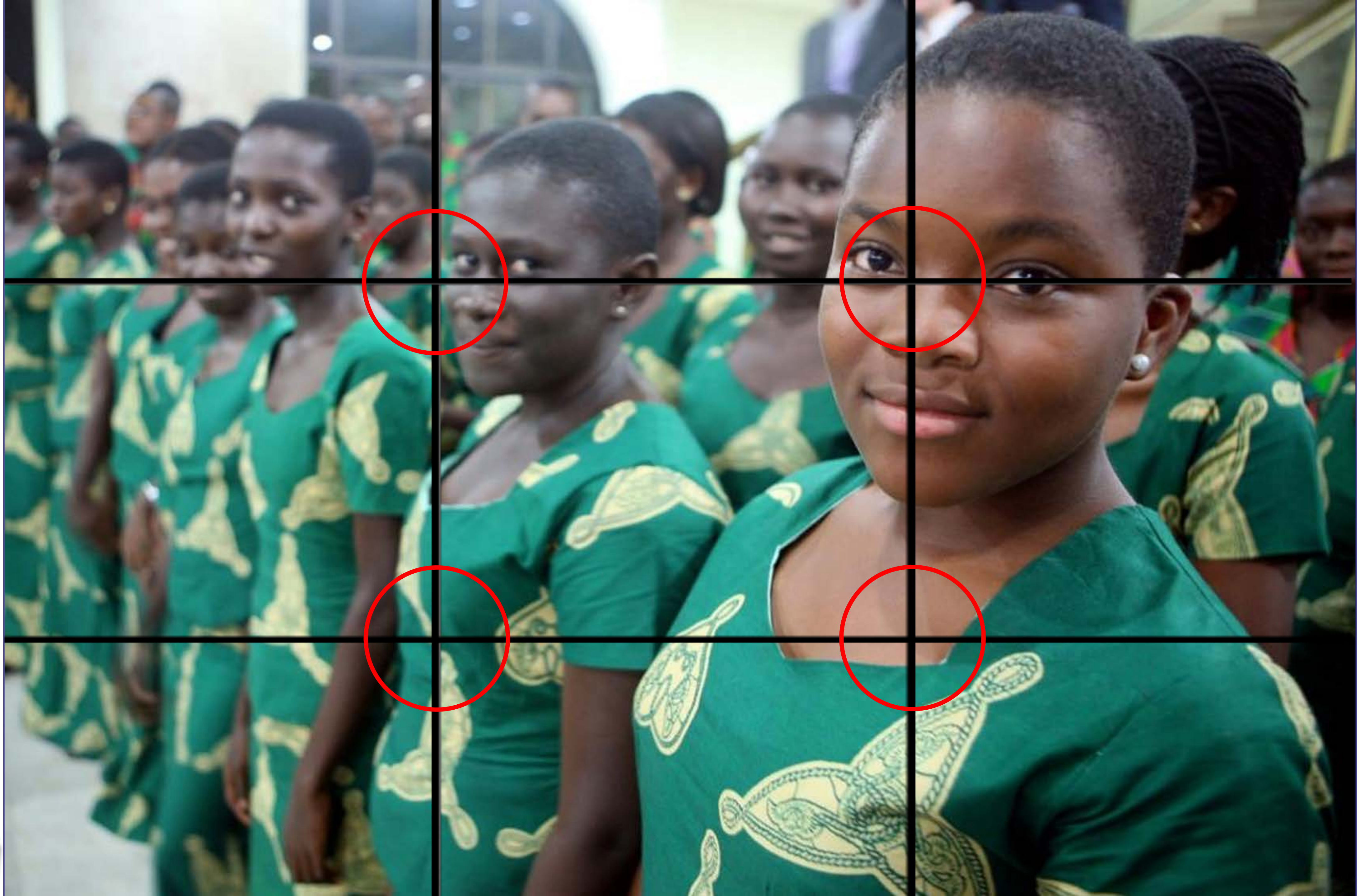

Portraits

- les portraits nécessitent une connexion personnelle
- respecter leur temps et espace

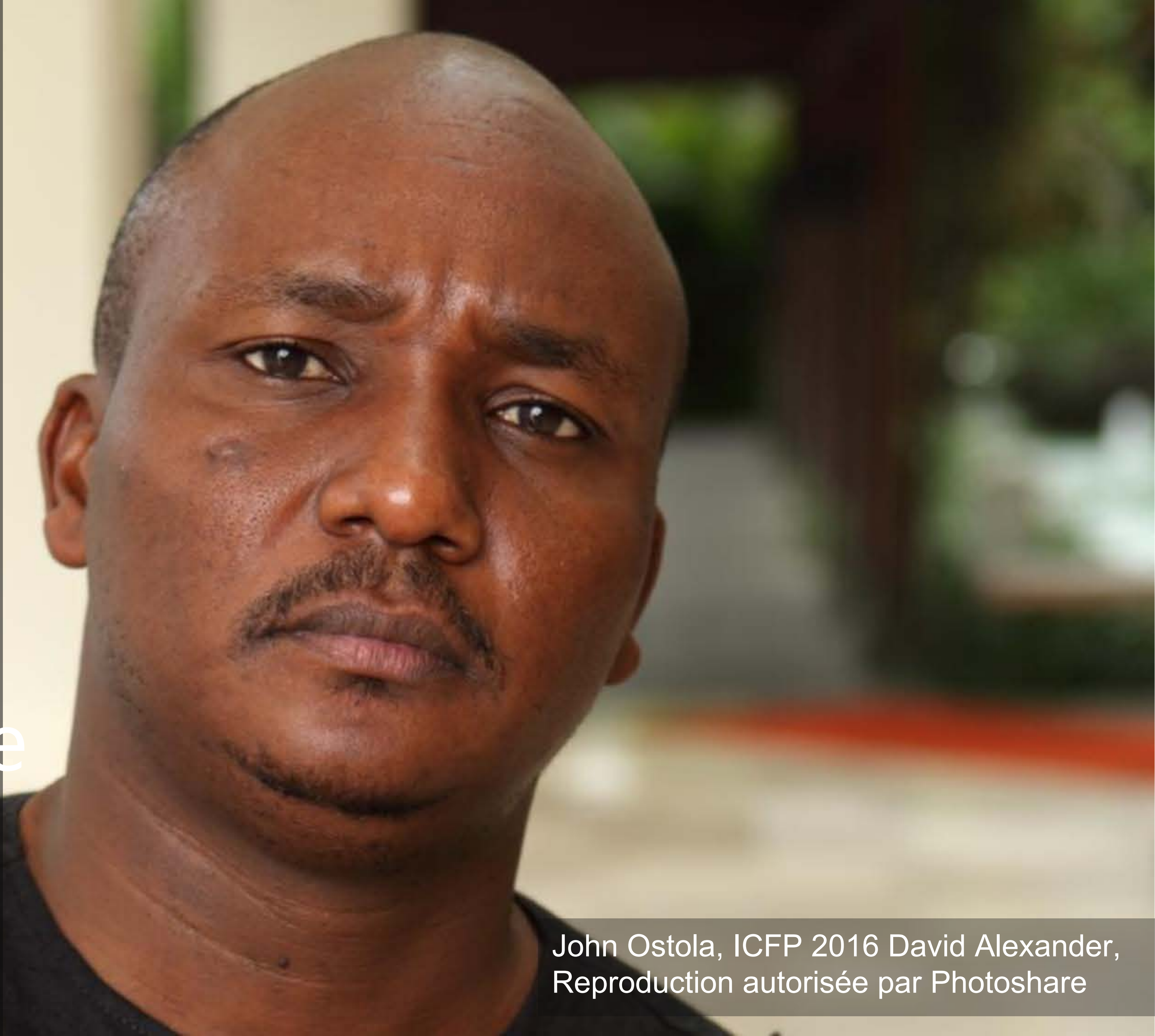

John Ostola, ICFP 2016 David Alexander, Reproduction autorisée par Photoshare

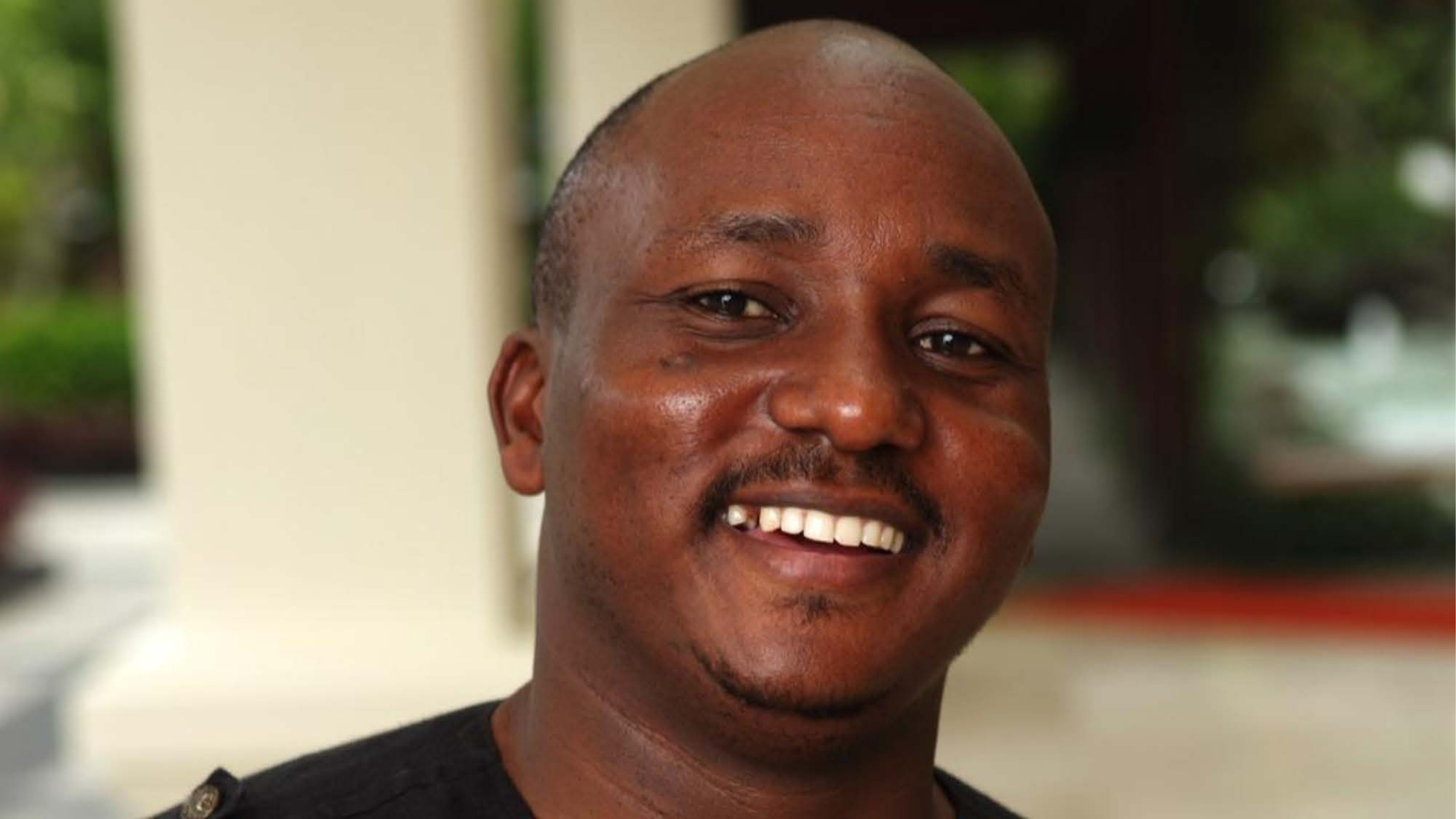

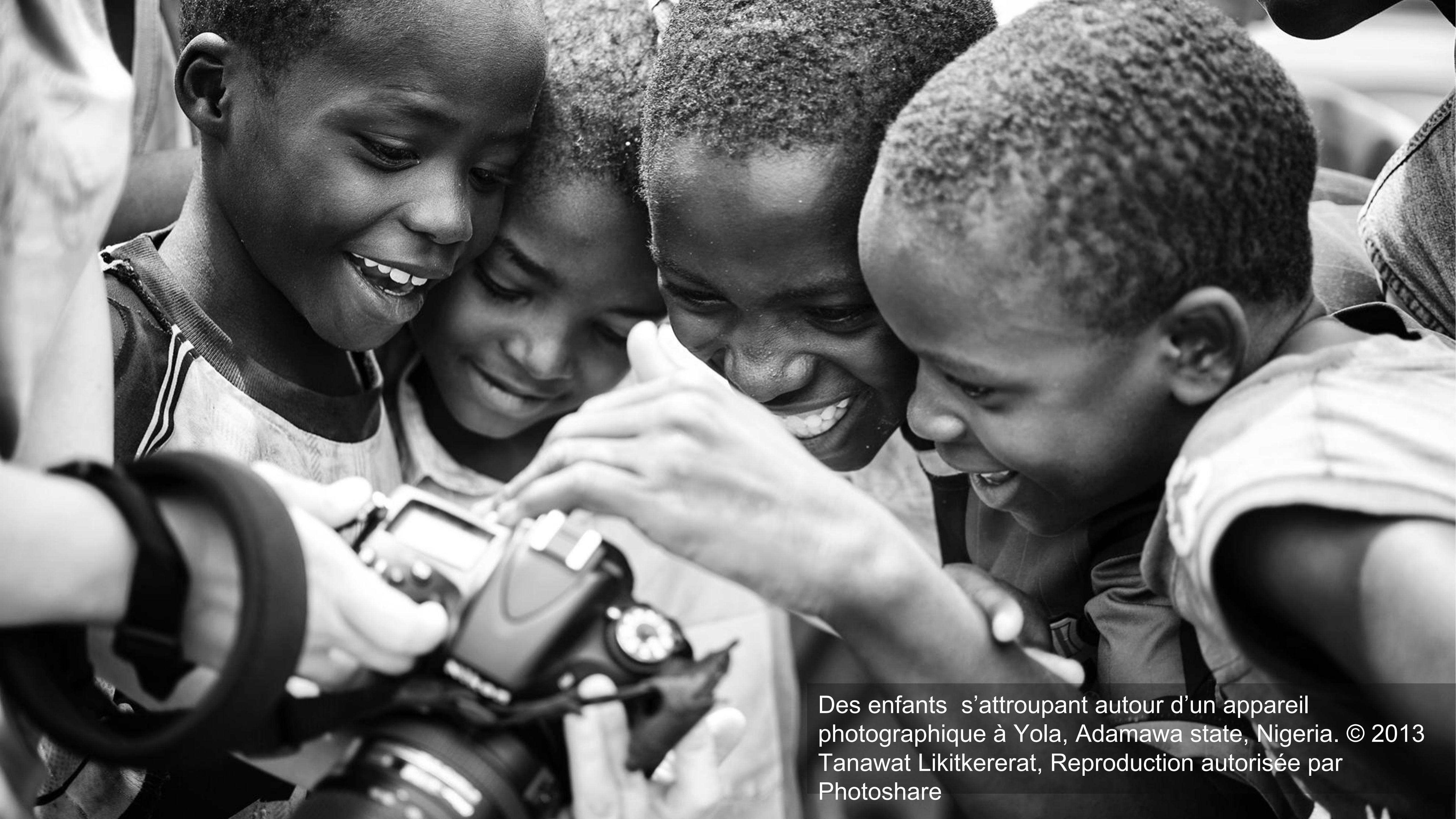

Des enfants s’attroupant autour d’un appareil photographique à Yola, Adamawa state, Nigeria. © 2013 Tanawat Likitkererat, Reproduction autorisée par Photoshare

Arrière-plans

- Complimenter le sujet
- Définir le récit

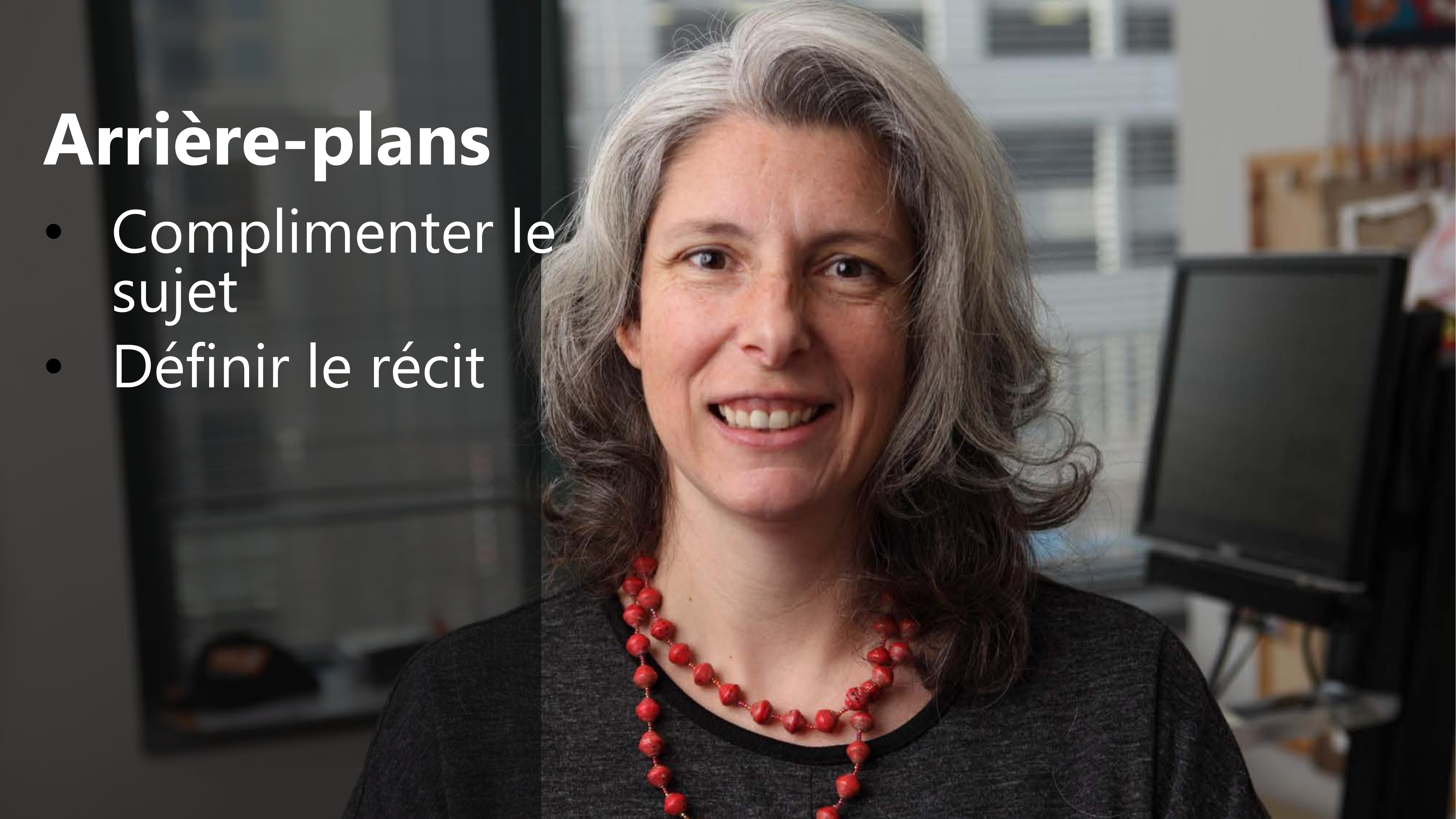

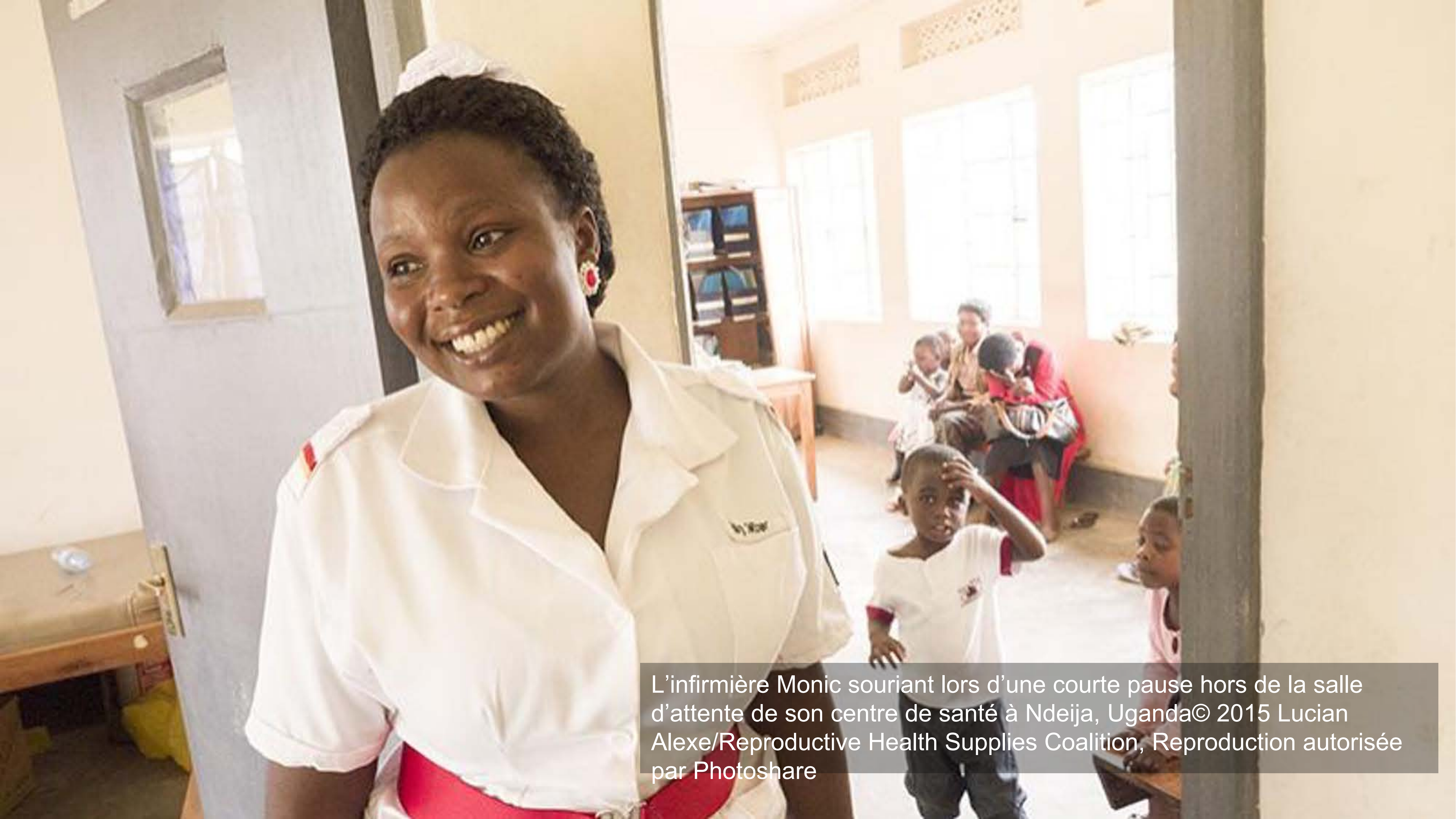

L’infirmière Monic souriant lors d’une courte pause hors de la salle d’attente de son centre de santé à Ndeija, Uganda© 2015 Lucian Alexe/Reproductive Health Supplies Coalition, Reproduction autorisée par Photoshare

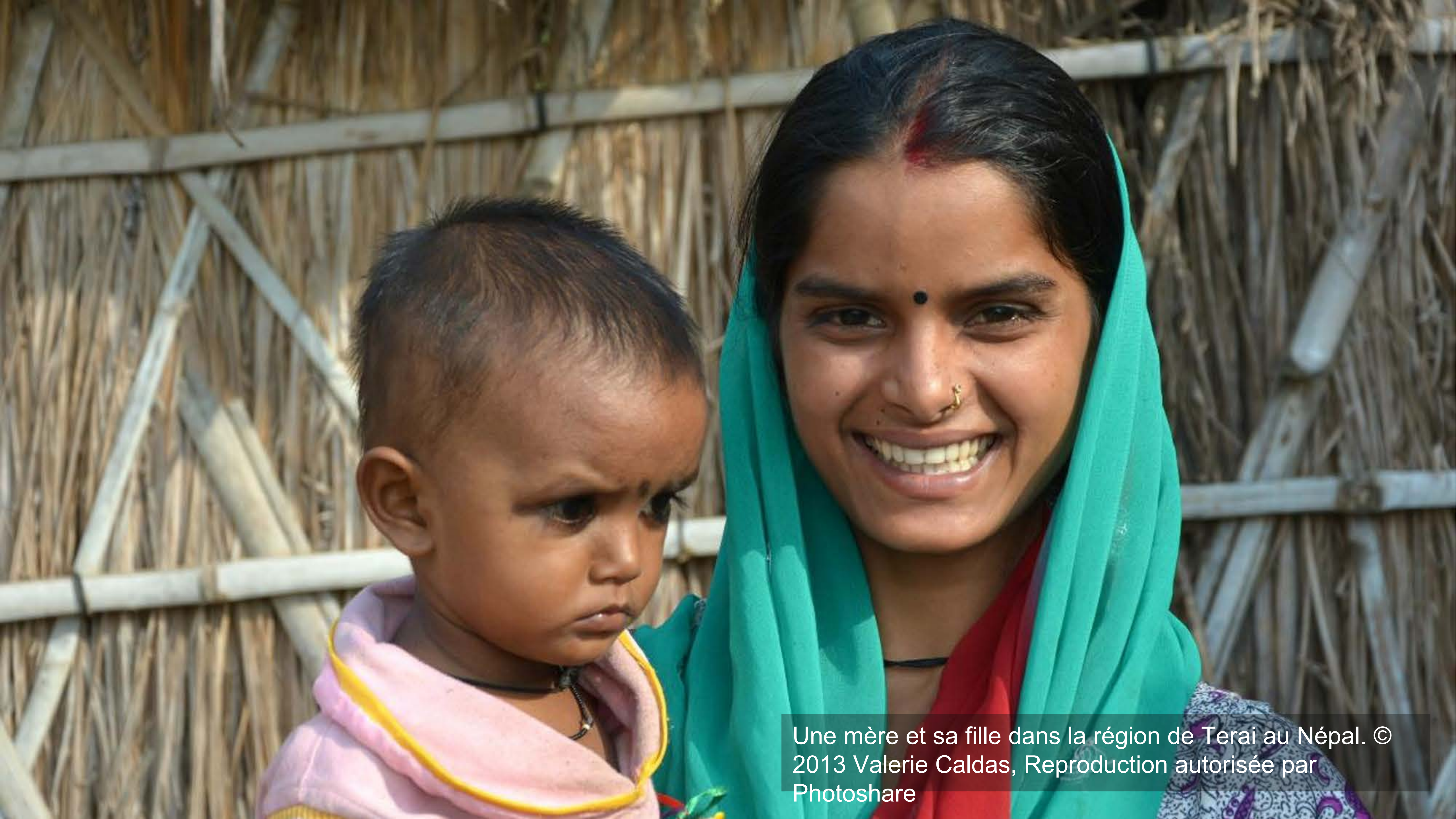

Une mère et sa fille dans la région de Terai au Népal. © 2013 Valerie Caldas, Reproduction autorisée par Photoshare

Matériel

- Appareils photo à mode manuel - automatique selon le besoin
- toujours mobile
- éclairage

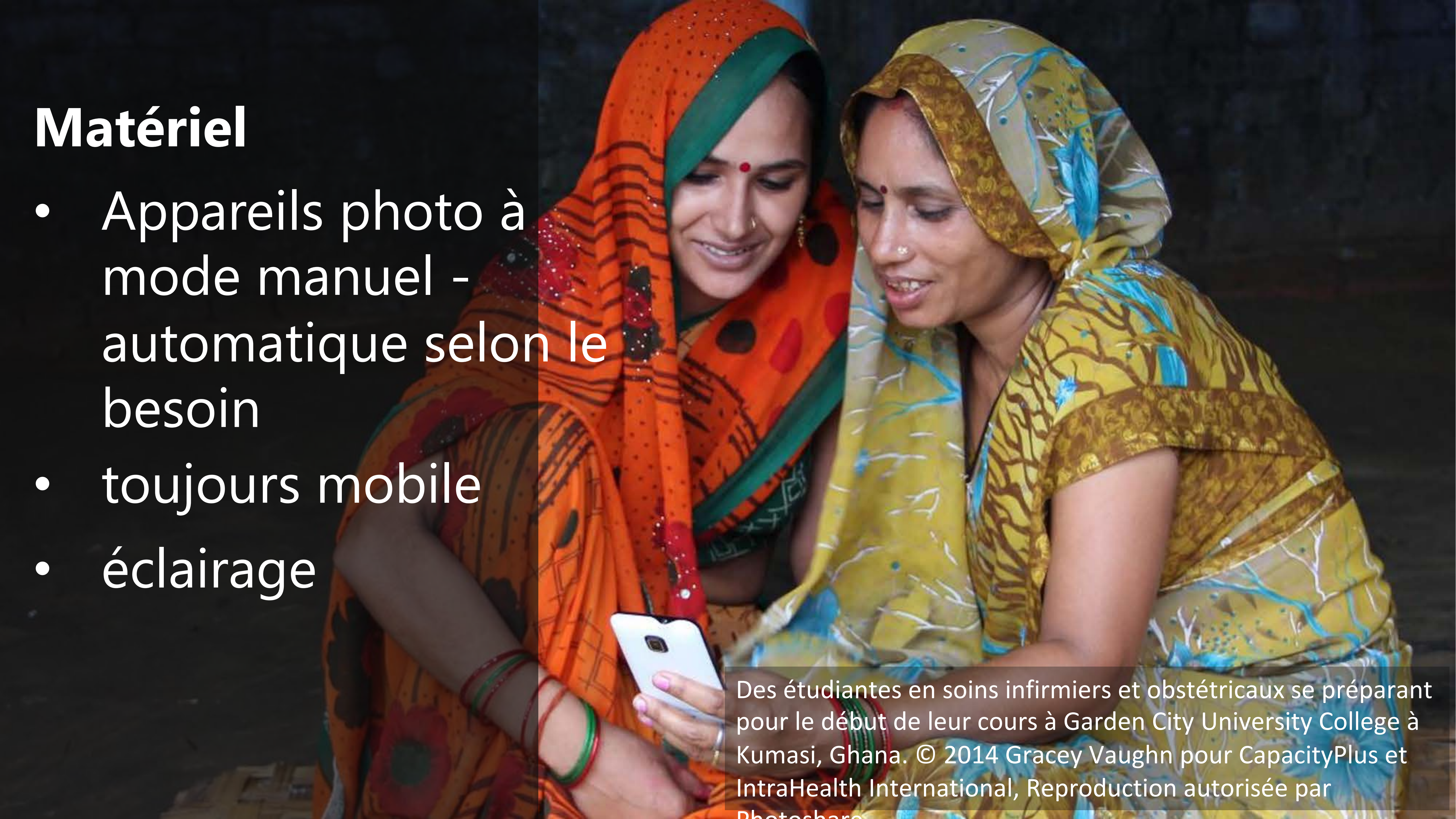

Des étudiantes en soins infirmiers et obstétricaux se préparant pour le début de leur cours à Garden City University College à Kumasi, Ghana. © 2014 Gracey Vaughn pour CapacityPlus et IntraHealth International, Reproduction autorisée par [illegible]

Lentille

- Investissement le plus observé

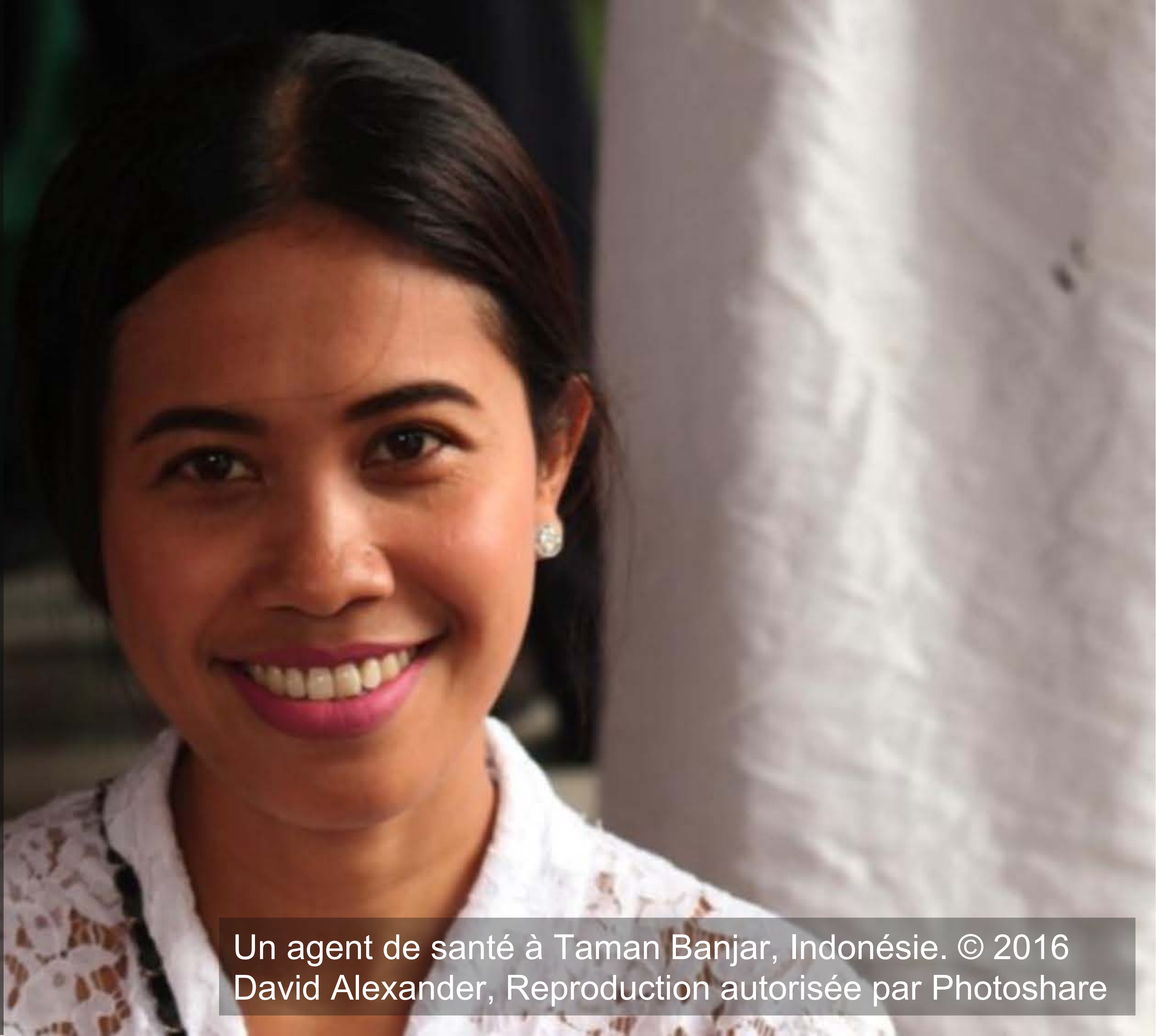

Un agent de santé à Taman Banjar, Indonésie. © 2016 David Alexander, Reproduction autorisée par Photoshare

Matériel d’éclairage

- réflecteur
- diffuseur
- Flash externe

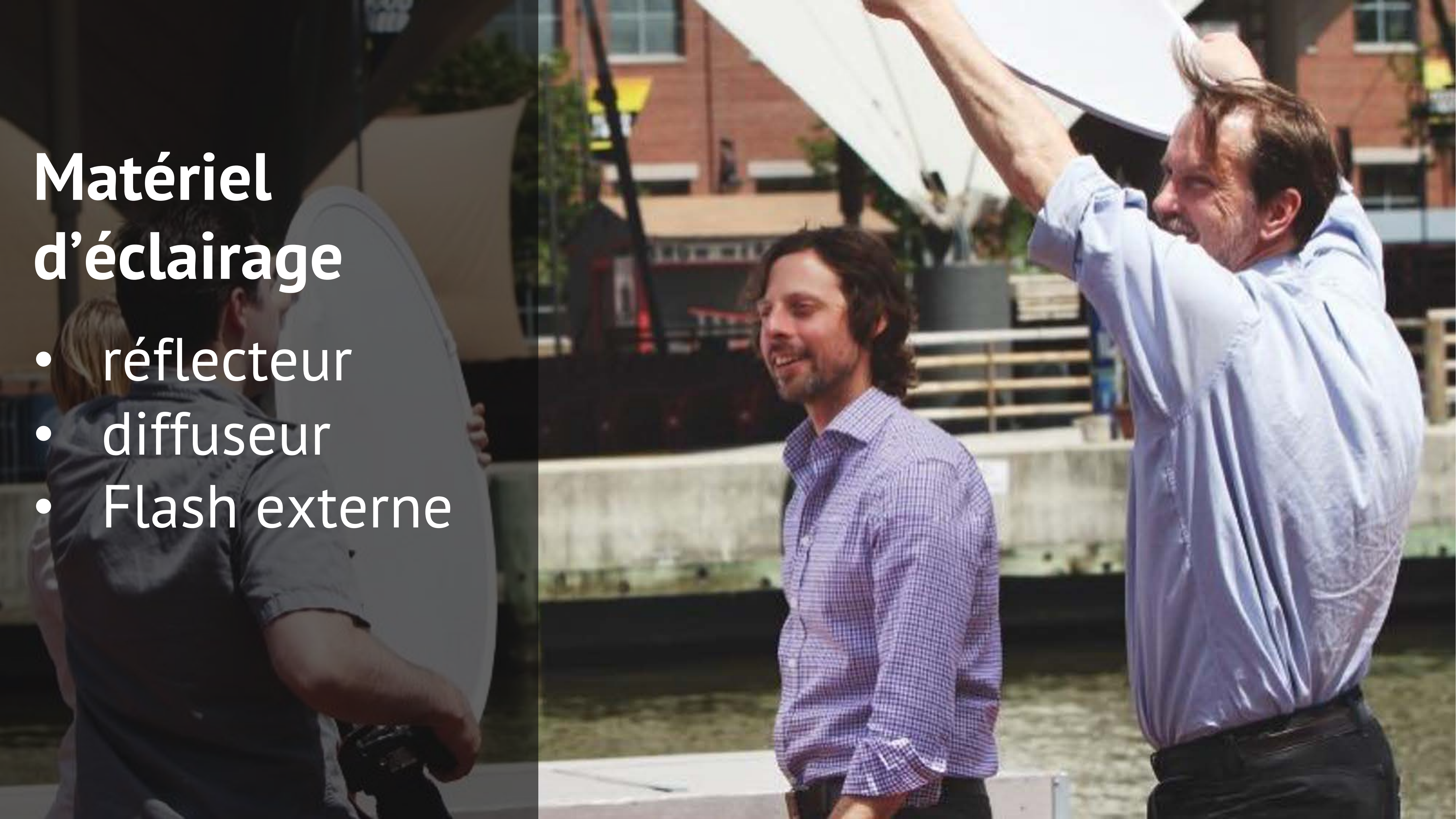

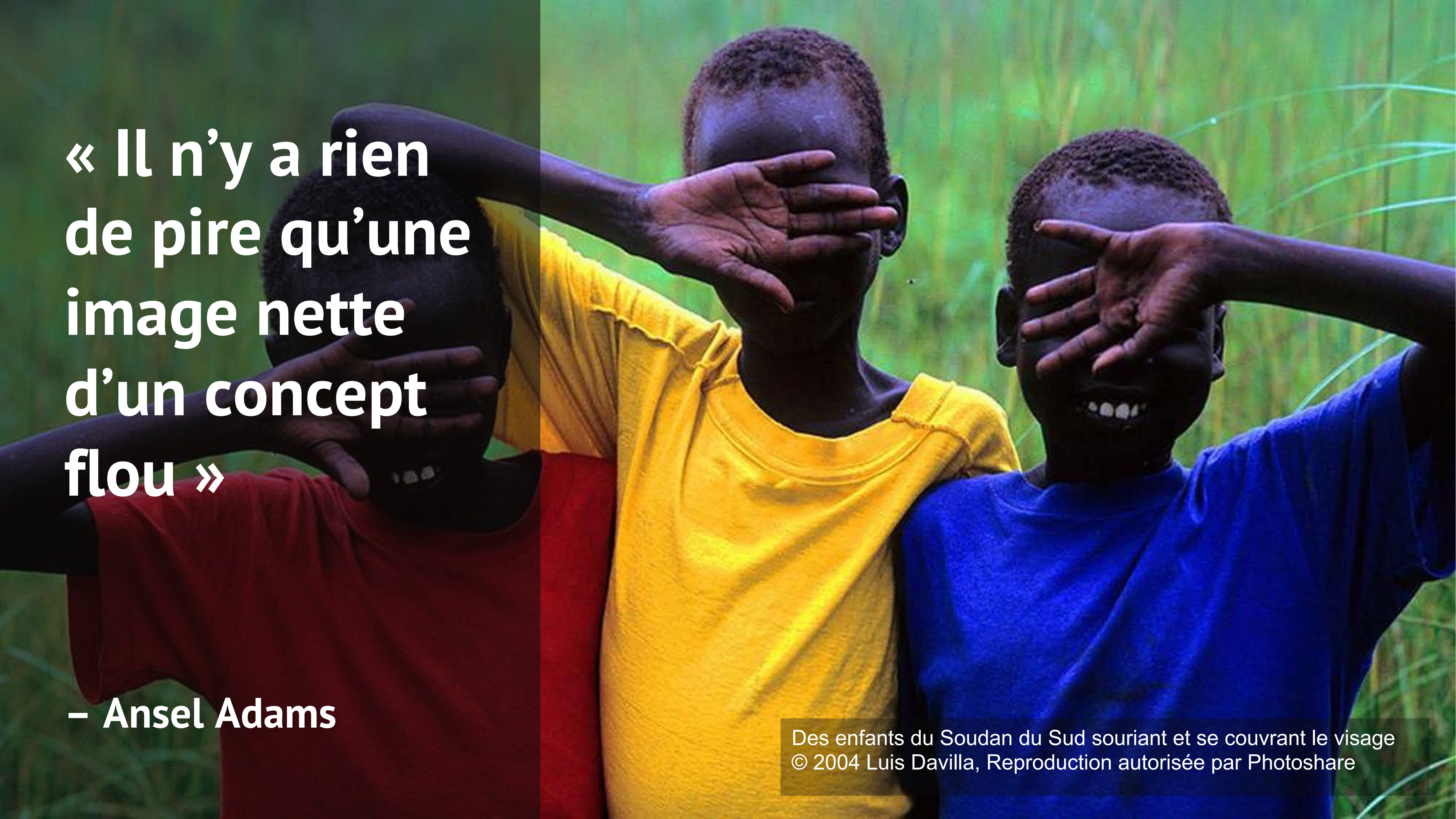

« Il n’y a rien de pire qu’une image nette d’un concept flou »

– Ansel Adams

Des enfants du Soudan du Sud souriant et se couvrant le visage
© 2004 Luis Davilla, Reproduction autorisée par Photoshare

Ethique en matière de photographie

Ethique

- Utilisation juste et
- Consentement éclairé

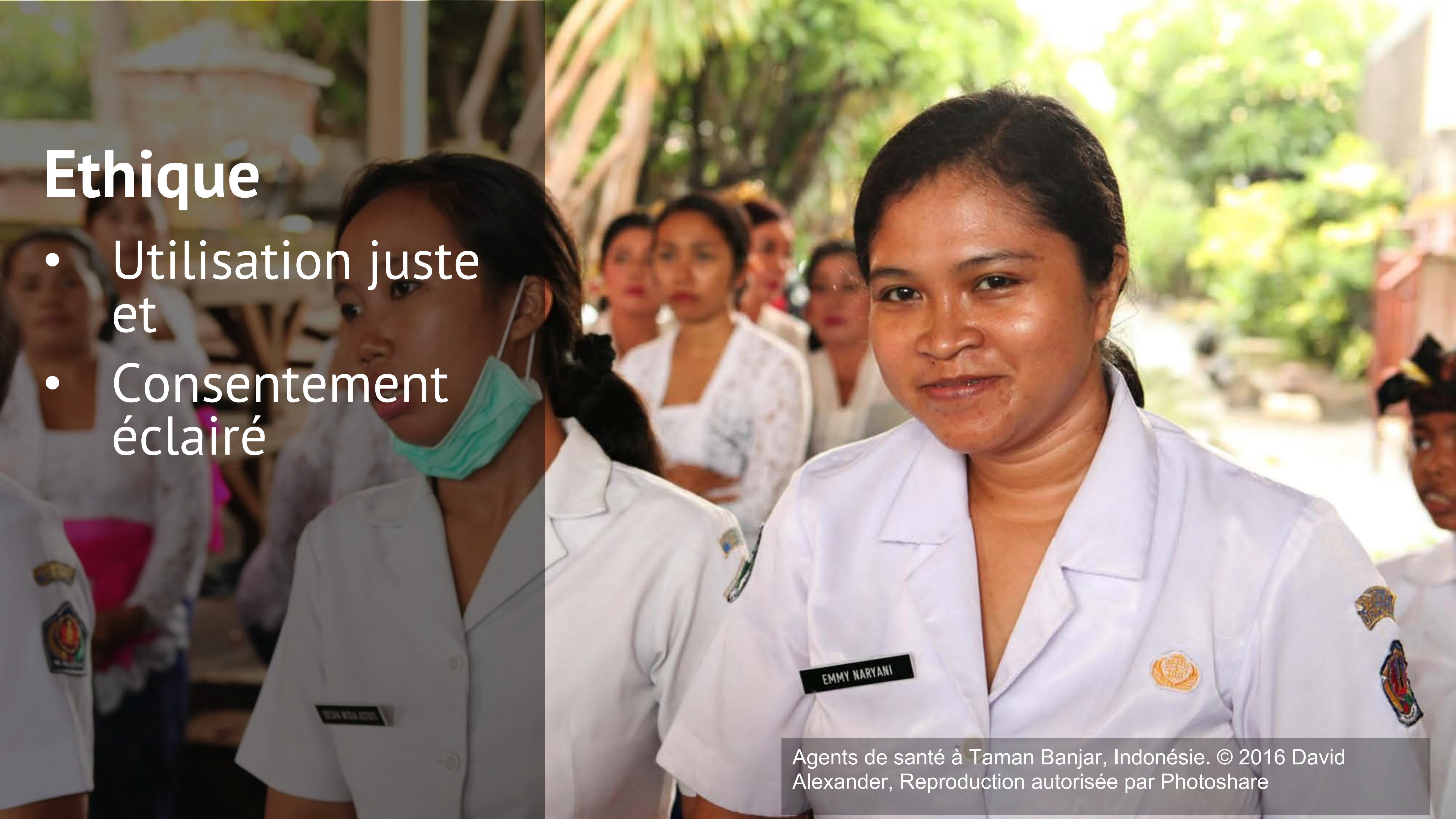

Agents de santé à Taman Banjar, Indonésie. © 2016 David Alexander, Reproduction autorisée par Photoshare

Editorial vs. Archive

- Éditorial: représentation précise de la situation réelle, de l’identité du sujet et de l’emplacement physique

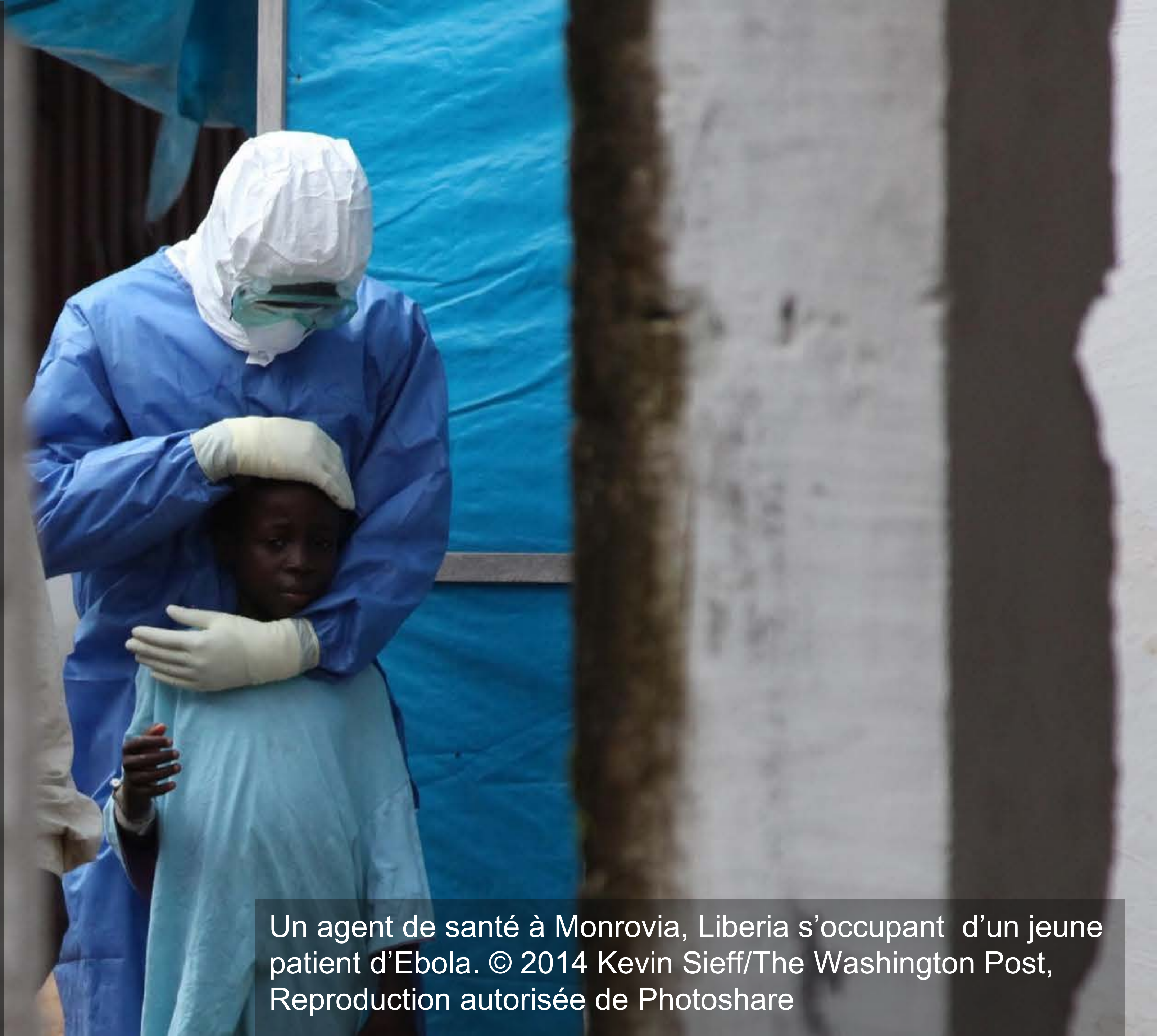

Un agent de santé à Monrovia, Liberia s’occupant d’un jeune patient d’Ebola. © 2014 Kevin Sieff/The Washington Post, Reproduction autorisée de Photoshare

Consentement éclairé

- Directives pour le consentement
- traducteurs
- alphabétisation
- protection des sujets

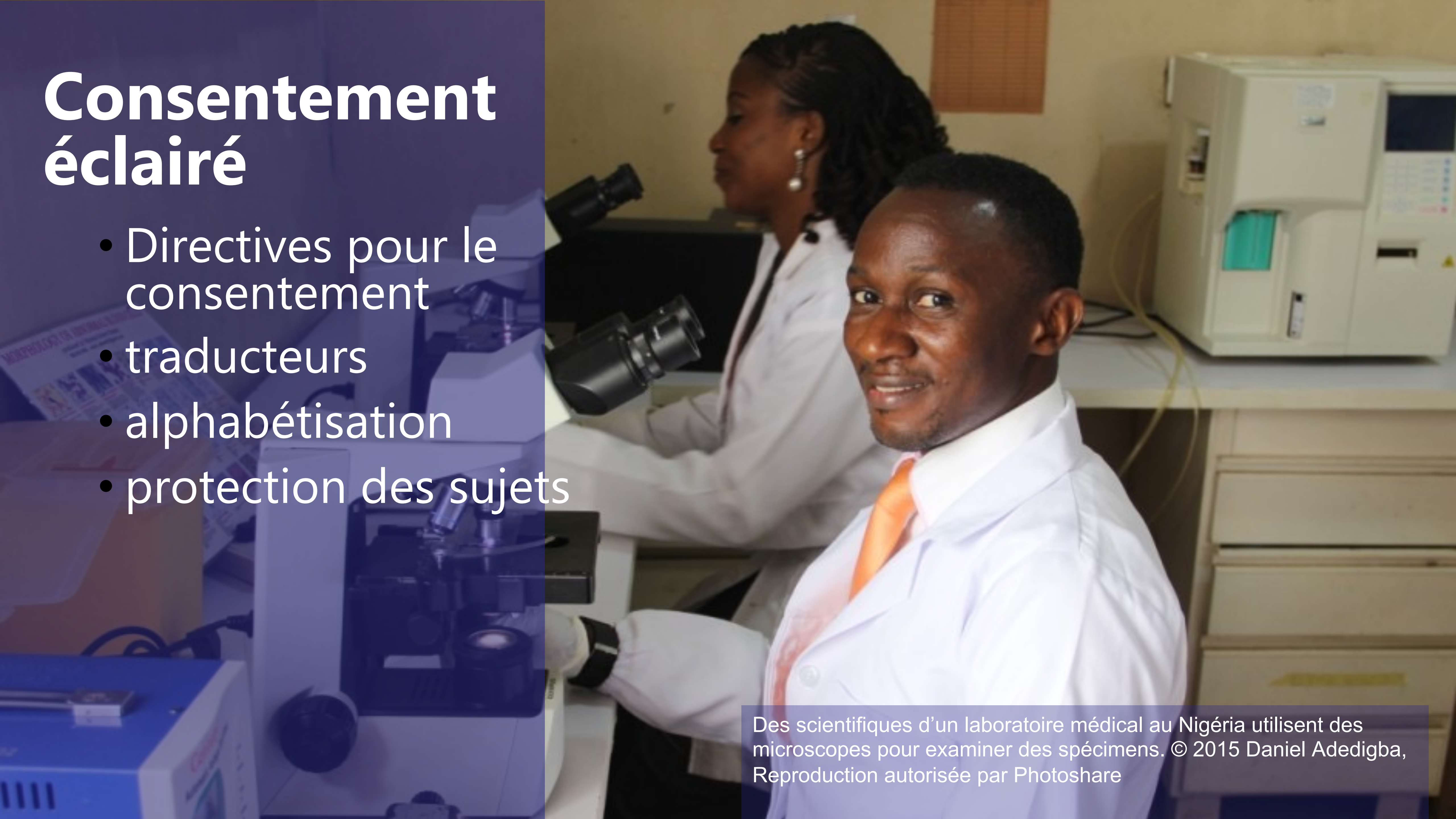

Des scientifiques d’un laboratoire médical au Nigéria utilisent des microscopes pour examiner des spécimens. © 2015 Daniel Adedigba, Reproduction autorisée par Photoshare

Consentement écrit vs. verbal

- Éthique éditoriale
  - Information personnelle
  - État de santé
  - État de comportement
  - Statut juridique
- consentement des enfants | des parents

- *les lois locales et les habitudes varient

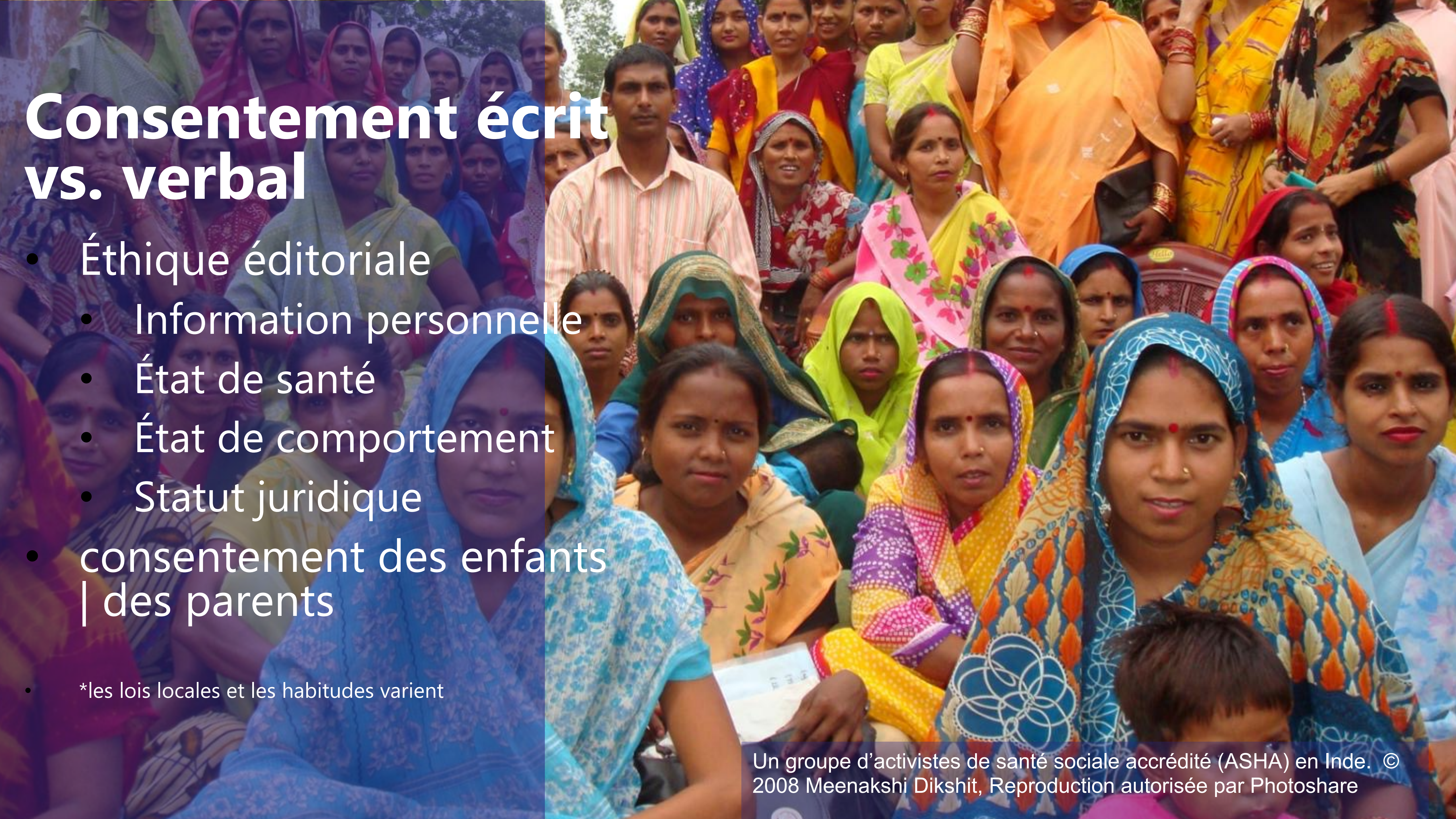

Un groupe d'activistes de santé sociale accrédité (ASHA) en Inde. © 2008 Meenakshi Dikshit, Reproduction autorisée par Photoshare

Consentement non nécessaire	Obtenir le consentement verbal	Consentement écrit encouragé
Personnes ne pouvant être reconnues en public (les visages et autres caractéristiques distinctives ne sont pas visibles)	Toutes les personnes quel que soit le lieu **dans la mesure du possible**	Prestataires et clients reconnaissables dans un **environnement clinique**
Personnalités publiques en public (ex. célébrités, personnel du ministère de la santé lors de lancements de campagne).	Parents, tuteurs ou enseignants des enfants.	Personnes reconnaissables ou non-reconnaissables dans un environnement où les **informations personnelles, privées s**ont exposées dans la photo ou documentées dans la légende correspondante notamment:<br>• Etat de santé (ex. personnes séropositives, personnes vivant avec le SIDA/les IST, tuberculose, maladies diarrhéiques, ayant pratiqué l'avortement, etc.)<br>• Comportement sanitaire (ex. travail du sexe, orientation sexuelle, alcool et utilisation de la drogue, utilisation de préservatifs, mutilation génitale féminine, etc.)<br>• Comportement criminel (ex. auteur ou victime d'acte de violence sexiste, etc.)
Foules en public (ex. public lors d'un concert à l'air libre).	Directeurs/Gestionnaires de cliniques ou d'autres programmes.	

- Respect de la vie privée

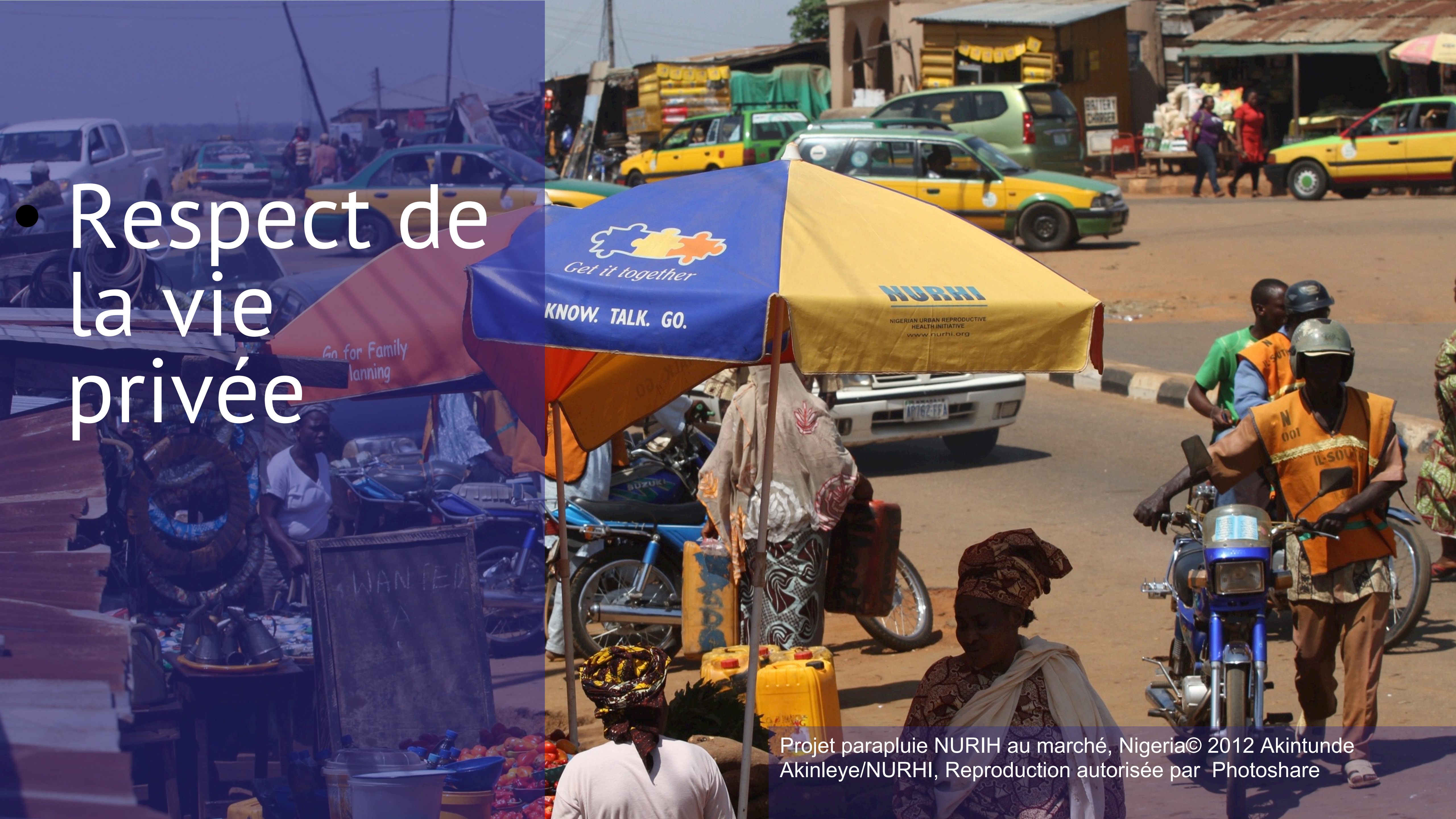

Projet parapluie NURIH au marché, Nigeria© 2012 Akintunde Akinleye/NURHI, Reproduction autorisée par Photoshare

- Respect de la vie privée

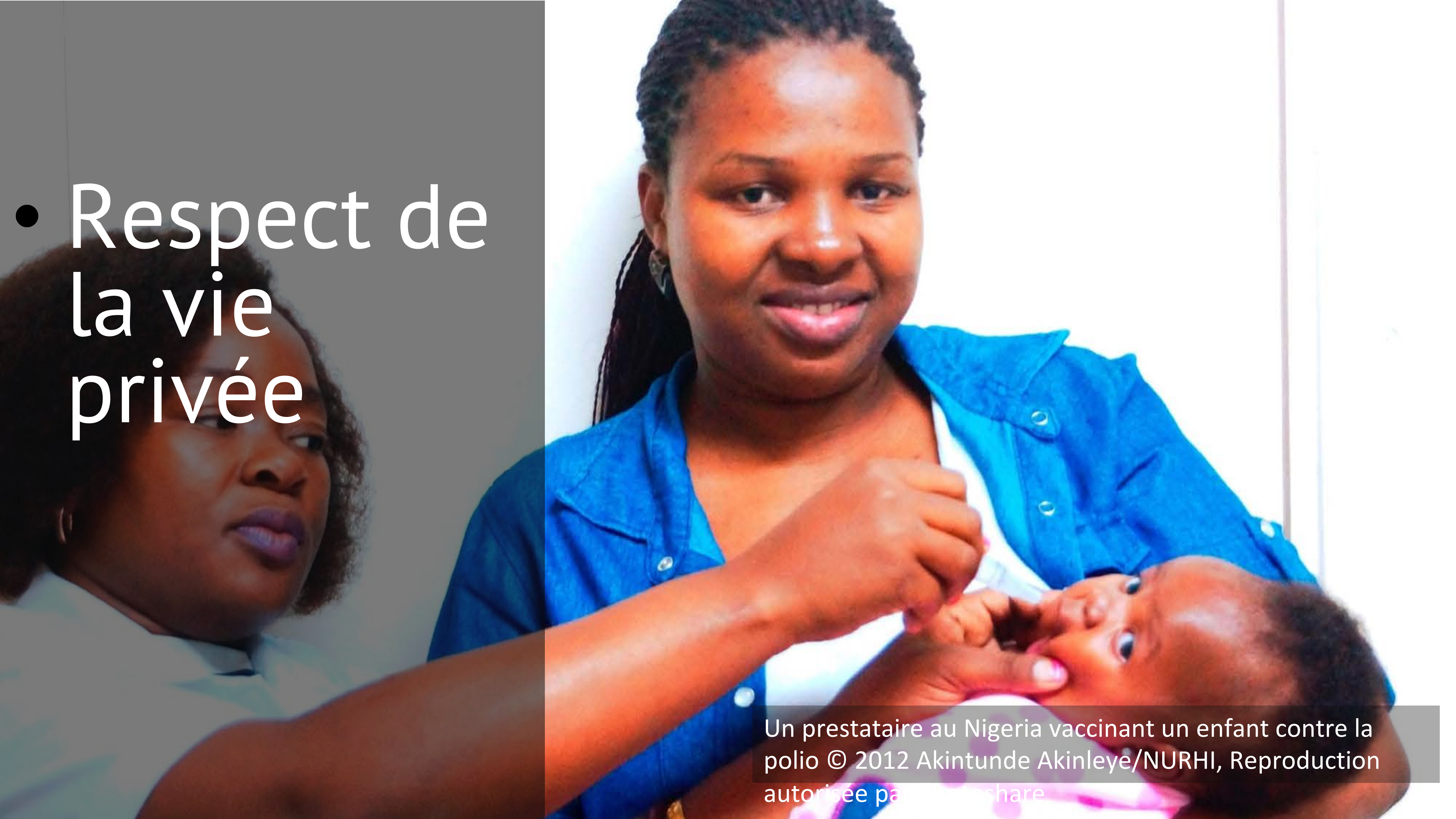

Un prestataire au Nigeria vaccinant un enfant contre la polio © 2012 Akintunde Akinleye/NURHI, Reproduction auto[illegible]sée pa[illegible]share

Exercice …

- Choisissez un partenaire
- Revoyez la section Annexe de votre guide de collecte d'histoires relatif aux conseils en matière de photographie
- Exercez-vous à prendre des photos l'un de l'autre à l'aide d'un appareil photo numérique ou de votre smartphone
- Prenez plusieurs photos de votre sujet (au moins 10 photos différentes les unes des autres) sous diverses perspectives et prenez en compte différentes compositions et arrière-plans

Echange et Débat

ECHANGE

- Laquelle des photos prises par ton partenaire as-tu le plus apprécié et pour quelle raison?

DEBAT

- Qu’avez-vous appris aujourd’hui et qu’est-ce que vous emporterez avec vous sur le terrain? Au sujet de l’interview relative au CPS ET à la photographie…
- Quels défis, selon vous, vont se poser et comment allez-vous les surmonter?
- Quel est votre plus grand atout ou force que vous pensez pouvoir vous aider dans le cadre de la collecte du CPS?

Jour 2

Programme de ce jour

- Elaborer un plan d'action pour la mise en œuvre du CPS au niveau des plateformes
  - Opérationnalisation de l'Etape 1
  - Revue et opérationnalisation de l'Etape 2: Sélectionner les histoires
  - Revue et opérationnalisation des Etapes 3 et 4: Feedback et intégration du CPS aux plans de données à l'action
- Mener des réflexions sur la formation et identifier un plan de suivi

Rappel des Etapes relatives au CPS

1. **Collecter les histoires** relatives au changement significatif
2. **Sélection des histoires les plus significatives** par les parties prenantes
3. **Partage** des histoires sélectionnées avec l'ensemble des parties prenantes
4. **Utiliser** les informations pour améliorer les programmes

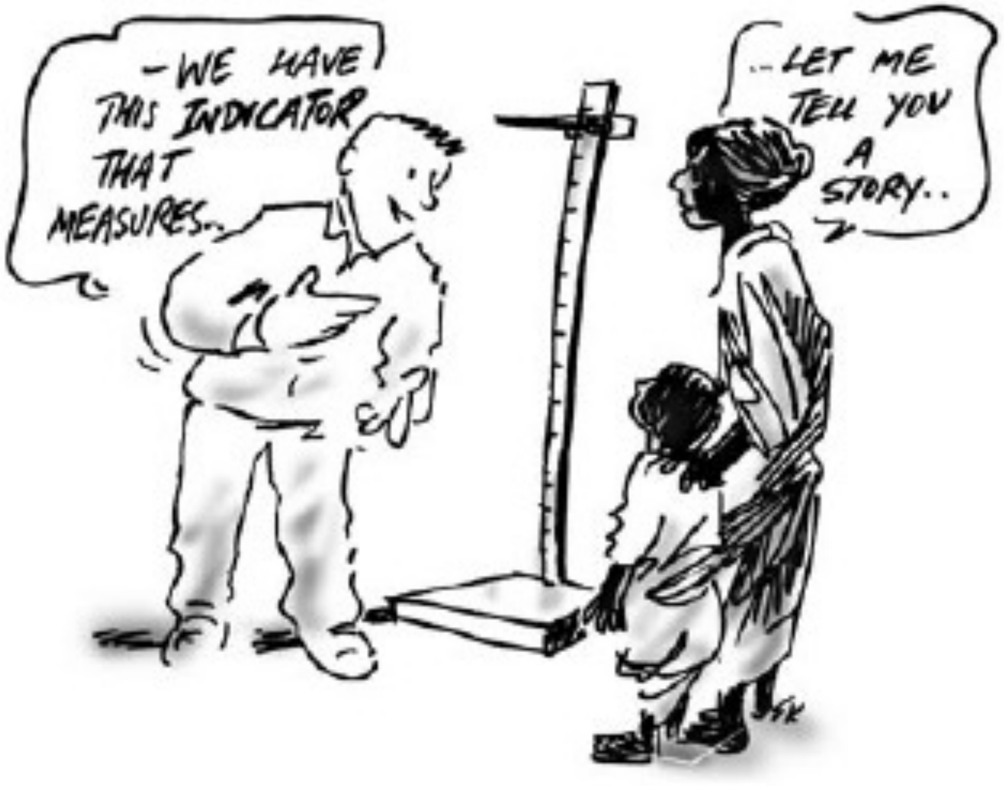

Image tirée de *The Most Significant Change Technique: A Guide to Its Use*, by Rick Davies and Jess Dart.

Opérationnalisation de l’Etape 1: la Collecte des Histoires

Qui sont les narrateurs ?

- Il s’agit de ceux qui dirigent le programme (c’est-à-dire les échanges interentreprises ; les personnes qui tiennent les rênes et qui peuvent faire la lumière sur les politiques, les finances et la viabilité)
- Les personnes qui mettent le programme en application (c’est-à-dire qu’elles sont en première ligne de défense ; d’habitude, elles interagissent avec les clients ou d’autres ONG ; des personnes qui peuvent nous informer de l’impact du programme)

Plan d'action

Liste d'exemples

Gestionnaires de programme

Liste d'exemples

Réalisateurs de projet

Qui sont les collecteurs d’histoire?

- Quelles sont les réunions et d’autres opportunités existantes déjà qui peuvent permettre de collecter ces histoires? Tiennent-elles régulièrement des rencontres?
- Quelles sont les personnes qui ont accès aux gestionnaires de programme et agents d’exécution via ces plateformes?

Quels sont nos objectifs/cibles? Commencer à élaborer un plan d’action

- Référez-vous à « Planification du travail pour la collecte d’histoires » dans le Plan d’action
- Discussion de groupe

Revue & Opérationnalisation de l'Etape 2, la Sélection des Histoires

Etape 2. Sélectionner les histoires les plus significatives

- Processus de sélection itératif afin de réduire le volume d’histoires valorisé.
- Implique à la fois les plateformes et le niveau Central (Global).
- Favorise des discussions entre les Plateformes et le niveau Central sur ce qui est le plus valorisé.
- **Le principal résultat du CPS n'est pas nécessairement la sélection des histoires CPS, mais plutôt le dialogue et l'émergence de valeurs!**
- Différentes façons de sélectionner les histoires (p. ex. règles de la majorité, vote itératif avec discussion, notation, vote à bulletin secret)

Exemple de processus de sélection hiérarchique

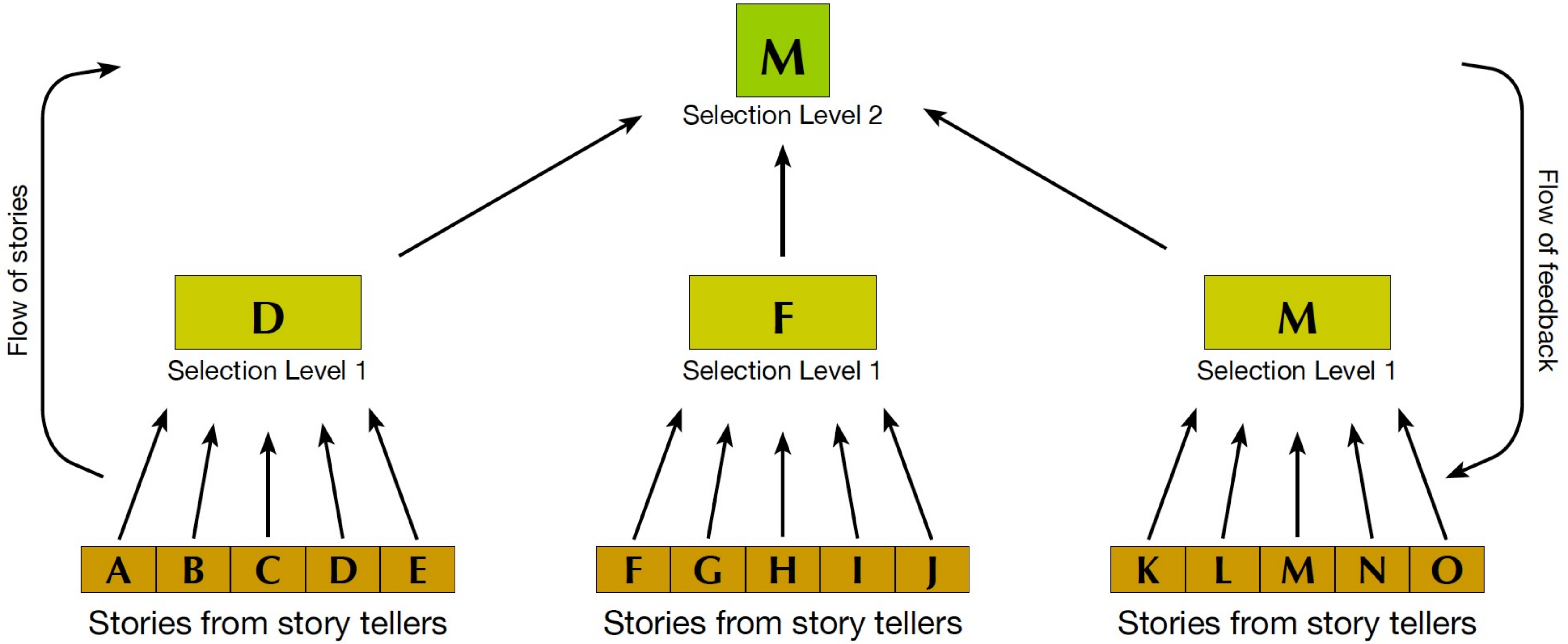

Qui peut faire partir des équipes de sélection? Quand peuvent-elles se rencontrer?

- Prenez en compte plusieurs perspectives mais essayez de les ajuster selon les disponibilités
- Devez-vous envisager des solutions alternatives pour les personnes qui voyagent souvent ?
- Ce groupe tient-il déjà des rencontres ?
  - Existe-il déjà une plateforme/rencontre à laquelle la sélection pourrait être incorporée?
  - A quelle fréquence se rencontrent-ils et/ou pourraient-ils se réunir?
- Considérez le nombre d'histoires: Il est irréaliste de s'attendre à ce que le personnel tienne des rencontres et travaille sur la sélection d'histoires relatives au CPS pendant plus de deux heures. En général, un groupe peut parcourir 10 histoires en 30 minutes.

Comment faire la sélection

- Asseyez-vous ensemble pour revoir une pile d'histoires sous chaque domaine
- Lire des histoires individuellement ou à haute et intelligible voix
- Le groupe tient une conversation profonde sur les histoires qui devraient être choisies
- Le groupe décide quelles histoires sont jugées les plus significatives
- Réduire la pile à une histoire par domaine
- Justifier les raisons du choix du (des) groupe (s)

Comment prenons-nous une décision?

- **La loi de la majorité** (rapide et facile, mais ne favorise pas de discussion sur la valeur)
- **Le vote itératif** (peut prendre beaucoup de temps mais favorise des jugements de qualité)
- **La notation** (idéale pour une réunion à distance, mais est plus discriminatoire et offre une possibilité limitée de dialogue)
- **La pré-notation, suivie de vote de groupe** (meilleure lorsque le temps de la réunion est réduit)
- **Le vote à bulletin secret** (s'il est utile de garder l'anonymat, mais nécessite encore une discussion!)
- *Nous vous encourageons à essayer les différentes méthodes afin de voir laquelle marche le mieux pour vous !*

Qu’entend-on par « plus » significatif?

- A quoi le compare-t-on pour faire de lui le PLUS significatif?
  - Rappelez-vous que ce processus est SUBJECTIF! Et non un calcul de « vérité ». Nous devons juger en fonction de notre point de vue et de l'interprétation.
- Référez-vous aux questions du programme d'apprentissage. Que voulons-nous comprendre pour savoir si ou comment nous atteignons nos objectifs?
- Choisir une méthode où chaque personne donne par écrit sa justification et son point de vue pouvant entraîner une réflexion plus critique
- ****Une histoire n’est pas invalide si elle n’est pas sélectionnée.**

Comment justifier votre choix

- Joindre la justification du choix à chaque histoire PS sélectionnée
- Utiliser plus de quelques mots. Donner en quelques phrases les raisons du choix

Pourquoi?

- Si les sélections doivent passer par un autre tour du processus de sélection ou d'examen par d'autres lecteurs
- Pour que tous les autres lecteurs comprennent pourquoi l'histoire est importante
- Pour aider à encadrer le feedback des histoires aux narrateurs, aux autres parties prenantes, aux autres plateformes et à globale afin que tout le monde comprenne le contexte.

Il est temps de passer à la pratique de la sélection d’histoires!

- Par table, effectuez une revue des histoires recueillies hier
- Décidez de comment vous allez procéder à la sélection d’histoires
  - La loi de la majorité
  - Le vote itératif
  - La notation
  - La pré-notation, puis vote de groupe
  - Le vote à bulletin secret
- Individuellement ou ensemble, lisez toutes les histoires collectées
- Sélectionnez l'histoire la plus significative
- Justifiez les raisons pour lesquelles vous avez sélectionné cette histoire

Rapport sur le processus de sélection

- Chaque groupe partage les histoires les plus significatives dans chaque domaine et les raisons de la sélection
- Qu'en est-il du processus?
  - Quelles méthodes de sélection chaque groupe a-t-il décidé d'utiliser?
  - Qu'est-ce qui a bien marché?
  - Qu'est-ce qui n'a pas bien fonctionné? Et comment proposez-vous de surmonter ces défis?
  - Voulez-vous utiliser cette méthode à nouveau?
- Avez-vous des questions?

Décider de votre plan

- Puis identifiez: quelles sont les personnes qui doivent faire partie de l'équipe de sélection/d'évaluation (pg 2)?
  - Rappelez-vous de prendre en compte différentes perspectives mais aussi les disponibilités
- Quand l'équipe devrait-elle se rencontrer sur la base des plans de travail de collecte des histoires?
- Quel processus de sélection/approche êtes-vous susceptible d'adopter (pg 3)?

Dernières étapes du processus CPS

Etape 3: Pourquoi repartager les histoires?

- Donner le feedback des histoires aux narrateurs et autres membres du personnel du projet
  - Peut encourager les gens à participer au processus CPS à l'avenir
  - Montre aux narrateurs que d'autres personnes ont lu et se sont appropriées leurs histoires
  - Un feedback sur la raison pour laquelle une histoire a été sélectionnée peut élargir ou remettre en question les points de vue des participants sur ce qui est important, ce sur quoi leur attention peut être porté dans le futur
  - Encourage le dialogue entre le sommet et la base de l'organisation

Feedback (et productions de contenu supplémentaire)

- Rapide feedback:
  - Aux personnes ayant partagé les histoires CPS
  - En amont de la chaîne organisationnelle, au prochain comité de sélection
- Productions
  - Verbale (Peut demander au narrateur de l'histoire retenue de partager son histoire)
  - Ecrite (Peut partager un profil en lien avec l'histoire sélectionnée – photo & citation)
  - Audio/visuelle (Peut partager le fichier audio ou développer un autre produit)
- Produits d'information supplémentaires afin d'assurer une large diffusion des histoires sélectionnées:
  - Webinaires, assistance par les pairs, groupes d'étude, mini-universités résidentielles.
  - Bulletins d'informations du niveau Central
  - Vidéo et photos d'interviews (et de la mise en œuvre ou des résultats « en action ») pour aider à raconter en retour les histoires CPS aux narrateurs, à travers les communes, les régions et les plateformes !

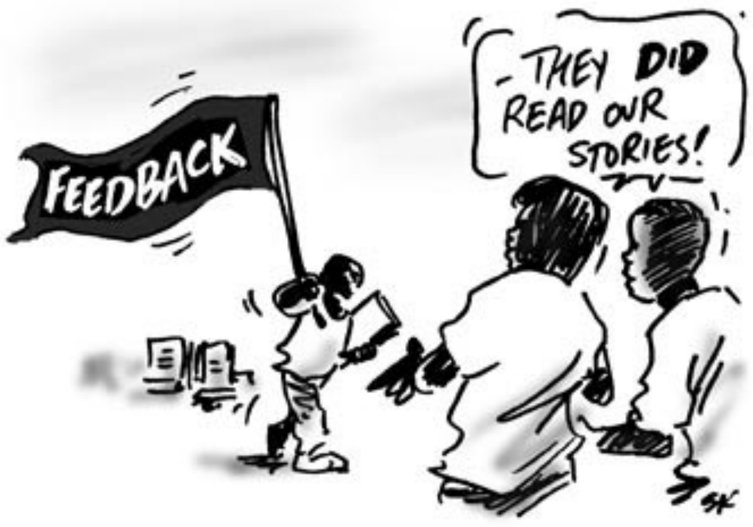

Image from *The Most Significant Change Technique: A Guide to Its Use*, by Rick Davies and Jess Dart.

Etape 4: Intégrer le CPS dans les plans de données à l'action

- L'intégration de CPS (données qualitatives) dans les données relatives au processus de prise de décision déjà établies (qui incluent des données quantitatives) permet de donner une image plus complète de l'impact et des défis du programme
- Le personnel de la plateforme chargé du S & E peut développer une stratégie avec le personnel de la plateforme GC sur la meilleure façon de planifier et d'intégrer le CPS avec d'autres données du programme
- Data viz est un excellent moyen de « montrer » les progrès!

Quelles sont les plateformes/rencontres existantes pour partager les données quantitatives de TCI

- Quelles sont les plateformes et rencontres se tenant déjà avec les parties prenantes TCI qui peuvent permettre une revue des progrès et données?
- Comment les histoires CPS peuvent-elles être incorporées à ces plateformes?

Ressources

- MSC Technique User Guide, by Rick Davies and Jess Dart: http://mande.co.uk/wp-content/uploads/2018/01/MSCGuide.pdf
- Jess Dart – Most Significant Change Part 1-5 + Q&A: https://youtu.be/H32FTygl-Zs
