# Supplementary material for: Lessons Learned From the Use of the Most Significant Change Technique for Adaptive Management of Complex Health Interventions
Source: Glob Health Sci Pract. 2022 Feb 28;10(1):e2100624. doi: 10.9745/GHSP-D-21-00624 (PMC8885358; doi:10.9745/GHSP-D-21-00624)
Supplement: 21-00624-Ohkubo-Supplement2.pdf [file 21-00624-Ohkubo-Supplement2.pdf]

Supplément à: Ohkubo S, Mwaikambo L, Salem R, Ajijola L, Nyachae P, Kumar Sharma M. Lessons learned from the use of most significant change technique for adaptive management of complex health interventions. *Glob Health Sci Pract*. 2022;10(1):e2100624. https://doi.org/10.9745/GHSP-D-21-00624

Guide d'interview TCI relatif au CPS

Introduction

TCI procède à la collecte d'histoires auprès de plusieurs parties prenantes afin de nous aider à mieux comprendre ce que TCI accomplit actuellement et comment nous pouvons répliquer les modèles de réussite et améliorer l'initiative ainsi que notre appui envers vous. Nous aimerions vous poser des questions sur tout changement significatif dont vous avez été témoin ou que vous avez vécu lors de la mise en œuvre du programmes suite à votre implication dans TCI. Nous nous intéressons a toute changement, mais nous nous intéressons particulièrement aux changements en manière de connaissance/attitudes, pratique, engagement/systèmes, et accès/qualité. Nous sommes intéressés à la fois par des changements positifs et négatifs en ce sens que ceux-ci nous permettront d'apprendre, adapter et en fin de compte mettre à l'échelle les services de planification familiale de haute qualité de façon durable. Il n y a pas de réponses justes ou fausses. Nous voulons simplement en savoir plus sur votre perspective.

L'interview ne devrait pas excéder 15 – 30 minutes et est totalement volontaire. Vous ne recevrez aucune sanction si vous décidez de ne pas y participer ou si vous vous y retirez à n'importe quel moment.

En répondant aux questions, veuillez être le plus précis possible même si vous pensez que je connais le contexte de votre histoire. Puisque nous comptons partager ces histoires avec des professionnels de la santé publique de par le monde, veuillez fournir le plus de détails possible sur votre contexte afin que d'autres personnes puissent bien comprendre votre histoire. Je prendrai des notes lors de notre conversation et aimerais l'enregistrer également. D'abord, j'aimerais obtenir votre consentement avant de débuter l'interview.

Formulaire de consentement

Nous pourrions utiliser votre histoire dans les rapports destinés à nos bailleurs de fonds et la partager avec d'autres parties prenantes TCI de ce pays ou dans d'autres régions TCI. Il est possible que nous partagions votre histoire avec une audience plus grande à travers des ressources et publications mondiales comme le site internet de TCI et les articles de journal.

Consentez-vous à ce que:

- Votre nom et celui de votre organisation soient associés à l'histoire? ☐ YES ☐ NO
- Votre image ou quelque chose du genre soit associée à l'histoire ? ☐ YES ☐ NO
- Un enregistrement audio de votre histoire/interview soit effectué ? ☐ YES ☐ NO

Je comprends que TCI peut utiliser et publier mon image, mon histoire et/ou des citations de cette interview ainsi que du contenu médiatique (photo, audio et/ou vidéo) de manière appropriée, juste et respectueuse. Je confirme que le présent contenu médiatique a été créé en toute connaissance de cause et avec mon consentement. Je comprends que je ne recevrai aucune compensation soit maintenant ou dans l'avenir pour l'utilisation de ce contenu.

____

Nom en majuscules

____

Signature Date

Supplément à: Ohkubo S, Mwaikambo L, Salem R, Ajijola L, Nyachae P, Kumar Sharma M. Lessons learned from the use of most significant change technique for adaptive management of complex health interventions. *Glob Health Sci Pract*. 2022;10(1):e2100624. https://doi.org/10.9745/GHSP-D-21-00624

Données démographiques de base

1. Nom du narrateur*: ____

2. Fonction du narrateur: ____

3. Employeur du narrateur*: ____

4. Lieu (ville, pays): ____

5. Sexe du narrateur: ____

** Ne pas enregistrer ces informations si le narrateur souhaite garder l'anonymat*.

Commencer les questions d'interview

À présent, j'aimerais commencer en vous posant quelques questions.

6. Quel a été selon vous le changement le plus significatif qui ait eu lieu au cours du dernier mois/trimestre (veuillez préciser la période) suite à la mise en œuvre de TCI ?

NOTE À L'INTERVIEWEUR: Si la personne interviewée a du mal à donner une réponse, vous pouvez lui dire que ce changement peut être personnel. Ex : a-t-elle appris quelque chose de nouveau ou un changement dans son comportement à l'égard de la planification familiale s'est-il produit ? Ou le changement pourrait avoir trait à un établissement sanitaire ou aux systèmes.

APPROFONDISSEMENT:

a. Quelle était la situation avant TCI ?

b. Que s'est-il passé/quel est le changement qui est survenu? Comment cela s'est-il produit? Quelles sont les personnes qui étaient impliquées?

c. Comment était la situation après le changement ?

7. Pourquoi selon vous cela est significatif?

APPROFONDISSEMENTS:

a. Quelle différence cela a-t-il apporté ou apportera-t-il dans le futur?

b. Quel a été l'impact de ce changement au niveau individuel, communautaire ou de la ville?

8. Quels sont les défis les plus importants auxquels vous êtes confrontés ou dont vous êtes conscient dans la mise en œuvre en faveur de TCI dans le dernier trimestre ?
