# Supplementary material for: Lessons Learned From the Use of the Most Significant Change Technique for Adaptive Management of Complex Health Interventions
Source: Glob Health Sci Pract. 2022 Feb 28;10(1):e2100624. doi: 10.9745/GHSP-D-21-00624 (PMC8885358; doi:10.9745/GHSP-D-21-00624)
Supplement: 21-00624-Ohkubo-Supplement3.pdf [file 21-00624-Ohkubo-Supplement3.pdf]

Supplement to: Ohkubo S, Mwaikambo L, Salem R, Ajijola L, Nyachae P, Kumar Sharma M. Lessons learned from the use of most significant change technique for adaptive management of complex health interventions. *Glob Health Sci Pract*. 2022;10(1):e2100624. https://doi.org/10.9745/GHSP-D-21-00624

Most Significant Change (MSC) Training for TCI Program Staff

2018

The objective is to build the capacity of Hub staff on the MSC process for collecting, analyzing, and sharing learnings across all TCI stakeholders and develop a plan for implementation at hub level.

**Most Significant Change (MSC) Training for TCI Program Staff**		
**Day I**	Session Theme: Step 1: Story Collection	Facilitator
**9:00 – 9:30**	Opening remarks	
**9:30 – 10:00**	Energizer/introductions and agenda walk through	
**10:00 – 11:00**	TCI methodology for systematic collection of learnings<br>• Overview of MSC technique	
**11:00 – 11:15**	Tea	
**11:15- 1:00**	Step 1: Collecting stories<br>• Why storytelling and examples of MSC stories<br>• Interviewing techniques including obtaining consent<br>• Review TCI MSC Interview Guide	
**1:00-2:00**	Lunch	
**2:00-3:15**	Practice collecting stories from each other<br>• Group work<br>• Discussion	
**3:15 – 3:30**	Tea	
**3:30-4:30**	Seeing the story: Photography techniques & ethics	
**4:30 – 5:00**	Practice collecting photos from each other (same pairs as earlier)<br>• Group work<br>• Discussion	
**5:00 – 5:30**	Wrap up	
**HOMEWORK IF MORE TIME IS REQUIRED**	Based on the two practice session, pull together the story that you collected from your partner	

Supplement to: Ohkubo S, Mwaikambo L, Salem R, Ajijola L, Nyachae P, Kumar Sharma M. Lessons learned from the use of most significant change technique for adaptive management of complex health interventions. *Glob Health Sci Pract*. 2022;10(1):e2100624. https://doi.org/10.9745/GHSP-D-21-00624

Most Significant Change (MSC) Training for TCI Program Staff		
**DAY II**	Session Theme: Steps 2-4 & Action Planning	Facilitator
**9:00 - 9:15**	Refresh memory	
**9:15 – 11:15**	Operationalizing Step 1: Collecting stories<br>• Who are your storytellers and collectors?<br>• How often will we collect stories?<br>• What existing platforms (such as meetings and other points of interaction) exist for collecting stories?	Facilitated group discussion
**11:15 – 11:30**	Tea Break	
**11:30 – 1:00**	Reviewing and Operationalizing Step 2: Selecting the most significant stories	Facilitated group discussion
**1:00 – 2:00**	Lunch	
**2:00 – 3:30**	Practice session with stories from yesterday	Group work
**3:30 – 3:45**	Tea	
**3:45 – 4:15**	Final steps of the MSC process<br>• Feedback results of story selection (newsletter, reports, videos, etc.)<br>• Data-to-action plans	
**4:15 – 5:00**	Developing a timeline for the 4-step MSC process & the Hub-level pause & reflect exercise	
**5:00 – 5:30**	Closing remarks	

Most Significant Change (MSC) Technique

Training Slides

Training Objectives

- Introduce the Most Significant Change (MSC) technique, its purpose and added value
- Understand how MSC works: Familiarize yourself with the four main steps
- Practice implementing MSC
- Create a state-specific operational plan for implementing MSC

Day 1

- Intro to MSC process (4 key steps)
- Step 1: Collecting stories
  - Interviewing technique
    - In-depth practice
  - Photography techniques & ethics
    - In-depth practice
- Review of the day

Day 2

- Develop an action plan to rollout implementation of MSC at the state level
  - Operationalizing Step 1
  - Review and Operationalization of Step 2: Selecting stories
  - Review and Operationalization of Steps 3 and 4: Feedback and Integrating MSC into Data Review Meetings
- Introduce Pause & Reflect
- Reflect on the training and identify follow-up plan

Day 1

TCI's methodology for systematic collection of learnings

It’s business unusual!

“If you knew what was going to happen in advance every day you could do amazing things. You could become insanely wealthy, influence the political process, et cetera. Well, it turns out that most people don’t even know what happened yesterday in their own business. So, a lot of businesses are discovering they can take tremendous competitive advantage simply by finding out what happened yesterday as soon as possible.”

-Steve Jobs, 1994

Using mixed methods to capture learnings

Quantitative data

- TCI dashboard
  - HMIS data
  - Survey data
    - PMA Agile and Local Data Collection Solutions (NURHI2 Flexitrack & Facility Surveys)
- TCI-U Google analytics
- Annual online survey of TCI-U registered users
- Project records

Qualitative data

- Most Significant Change (MSC)
- Pause & Reflect
- Other in-depth interviews

What do you know about MSC?

- Have you heard about MSC?
- What do you know about MSC?

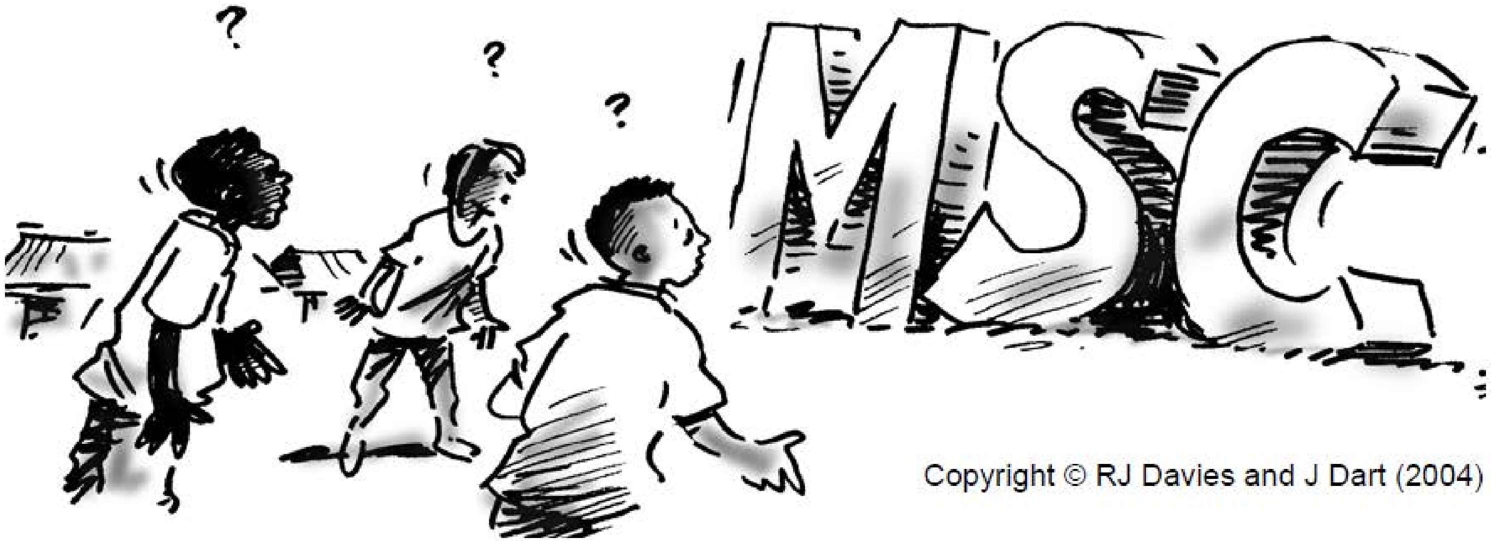

Copyright © RJ Davies and J Dart (2004)

What is MSC?

- The MSC technique is used to gain understanding of **what is being achieved** and **how it is being achieved**

- It is a **participatory** monitoring and evaluation technique

- It provides qualitative evidence to support quantitative evidence

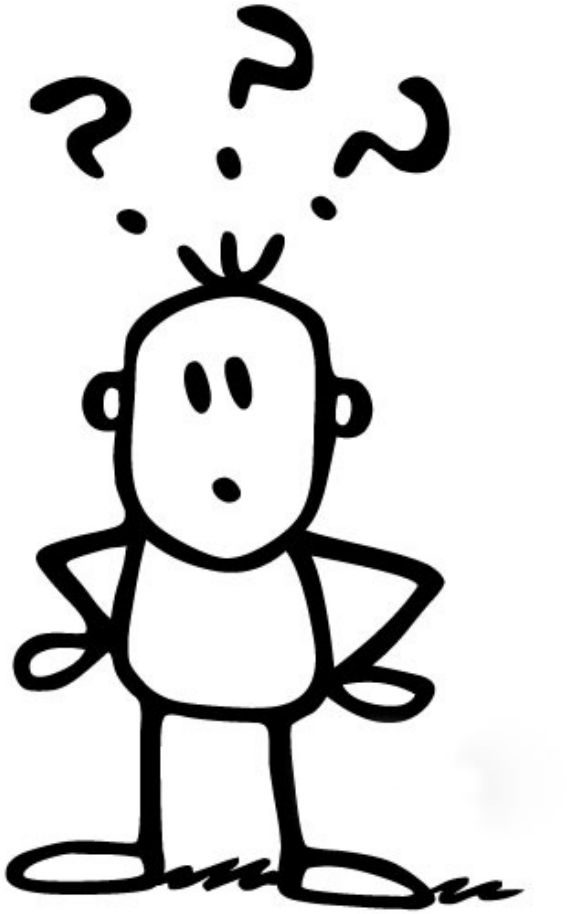

It is a Story!

- People tell stories naturally
- People remember stories
- Stories can carry difficult messages that might otherwise be impossible to collect
- Stories provide richness, complexity, and context
- Stories provide a basis for further discussions

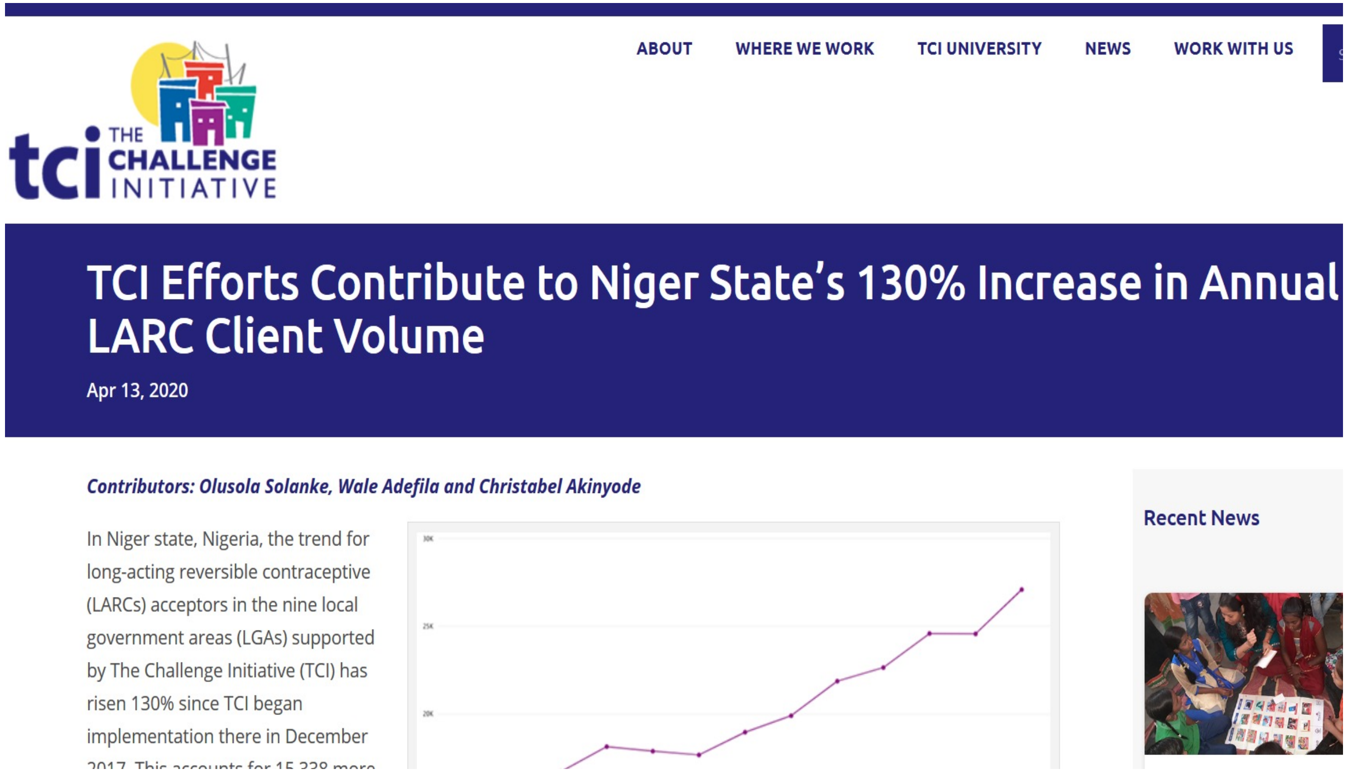

Four Basic Steps

- **Collect stories** about significant changes
- **Select the most significant** stories by stakeholders
- **Feedback** the selected stories to all stakeholders
- **Use** the information to improve programs

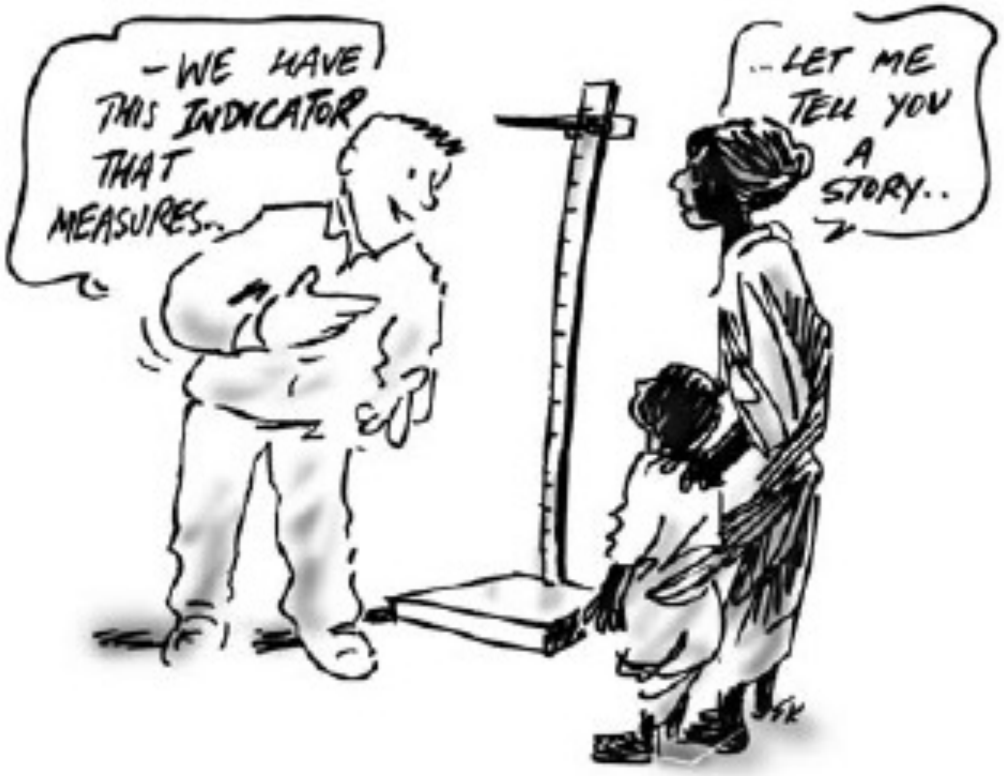

Image from *The Most Significant Change Technique: A Guide to Its Use*, by Rick Davies and Jess Dart.

3 Whats and 1 Why!

Interview TCI stakeholders, asking:

- What the situation was like before TCI?
- What were the changes as a result of TCI?
- What was the most significant change and Why do you think it is significant?

What & Why

Domains of Change (Stories of Interest)

Domain of Change	Mapped to Sustainability Pillars	Illustrative TCI Examples
Knowledge, Attitudes/Mindsets, and Practice	Pillar 2: Capacity Strengthening	• Increased knowledge of the TCI model<br>• Increased knowledge of proven family planning programming approaches<br>• Increased knowledge of best practices for program implementation<br>• Shifts in mindsets of the roles of implementers and coaches<br>• Rapid adoption/adaptation of TCI tools and proven approaches in service delivery, demand generation, advocacy
Political and Financial Commitments	Pillar 1: Political & Financial Commitments	• Financial commitment of geographies<br>• Funds that are actually spent<br>• Positive statements of government officials and community and religious leaders
Systems	Pillar 3: Institutionalization	• Local ownership of programs<br>• Institutionalization of approaches<br>• Data use for decision making
Access and Quality	Pillar 4: Sustained Demand (FP Impact)	• Social mobilization to improve awareness of services<br>• Advocacy with religious leaders to improve individual and societal perceptions of family planning<br>• Expanding method choice<br>• Reducing commodity stock-outs

Measuring sustainable scale up

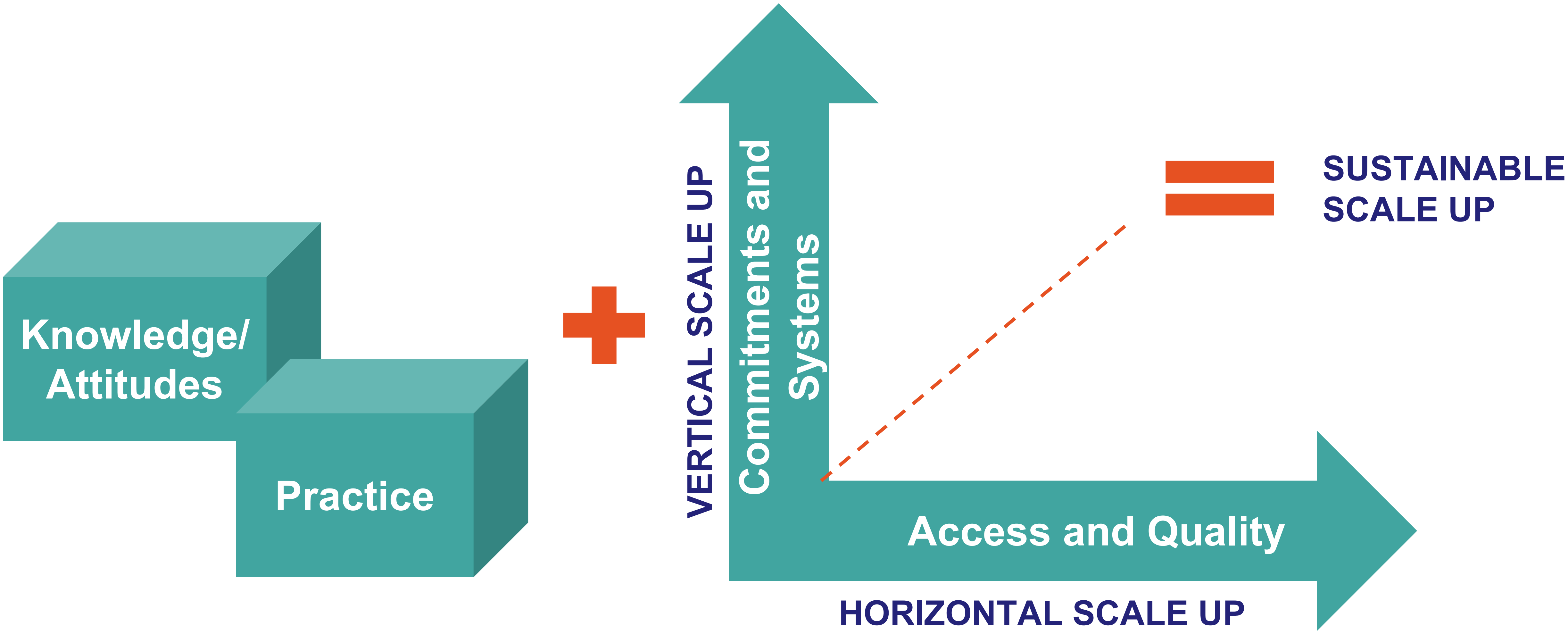

Discussion questions

- In your opinion, what are the benefits and challenges to MSC as a learning tool?

- In your opinion, what are the benefits and challenges to implementing MSC at your Hub?

Step 1: Collecting Stories

TCI Nigeria Example

"I found out about the social malpractices that were harmful to our health only after attending Suaahara trainings," says Kalawati, "For the first time in my life, I had the opportunity to attend training on Integrated Nutrition and Sanitation."

"None of the mistakes that I had committed while giving birth to my older daughters were repeated at the birth of my fourth daughter," she says. She underwent antenatal checkups at four, six, eight and nine months. "I did not miss any of these – iron and folic tablets, deworming, tetanus toxoid immunization, iodine salts, supplementary foods such as green vegetables and pulses," she adds.

TCI Nigeria Example

Mamta reported from her visit with a field program assistant (FPA) in Fiorzibad:

“When the FPA initially introduced herself to the ASHA, the ASHA complained of being overwhelmed and preoccupied with her other tasks. She said she didn't have time to bother with family planning counseling. She asked the FPA: ‘Why are you creating pressure on us?’ A month later and the interaction couldn't be more different, the ASHA has come to appreciate the FPA and TCIHC for its coaching. She explained that with what she's learned from the FPA ‘it has made life easier; she can identify clients who have FP needs and do quality IPC calls.’”

TCI Nigeria Example

Phoebe Yakubu, a family planning provider in Kaduna, commented on the outreach, "Before the exercise I only knew of the injectables and pills...now I know many more methods. I really appreciate the experience now when I go out holding my [Get it Together] bag and cap, people respect me. They say 'Ah madam, we are coming to your clinic."

Yakubu says, "...as a result of all the mobilization and awareness created, even the regionals now understand that it is important for a woman to do family planning because family planning is not about stopping childbirth, but helping her to space her child and to improve the relationship between the men, women, and their children. I pray this organization [NURHI] will be strengthened because every month we have up to 50 or more clients for family planning alone."

How to collect a MSC story!

It's simple ☺

Collect "significant change" stories at community/ward, LGA and state levels on a quarterly basis:

- *What the situation was like before TCI?*
- *Looking back over the last quarter, what were the changes as a result of TCI?*
- *What was the most significant change and Why do you think it is significant?*
  - *What difference has this made now or will make in the future?*

An MSC story...

Is:

- An interview or discussion about personal experiences/perspectives
- Qualitative data collection
- An opportunity to listen and learn
- Told in first person

Is not:

- A case study
- A blog post
- A journal article
- An activity report
- An opportunity to coach or advocate
- Told in third person

Must Haves and May Haves

Must Have	May Have
• Consent<br>• Responses to key questions<br>• Quotes<br>• Photo (as separate file)	• Supporting Data

Interviewing Techniques

The art of the interview

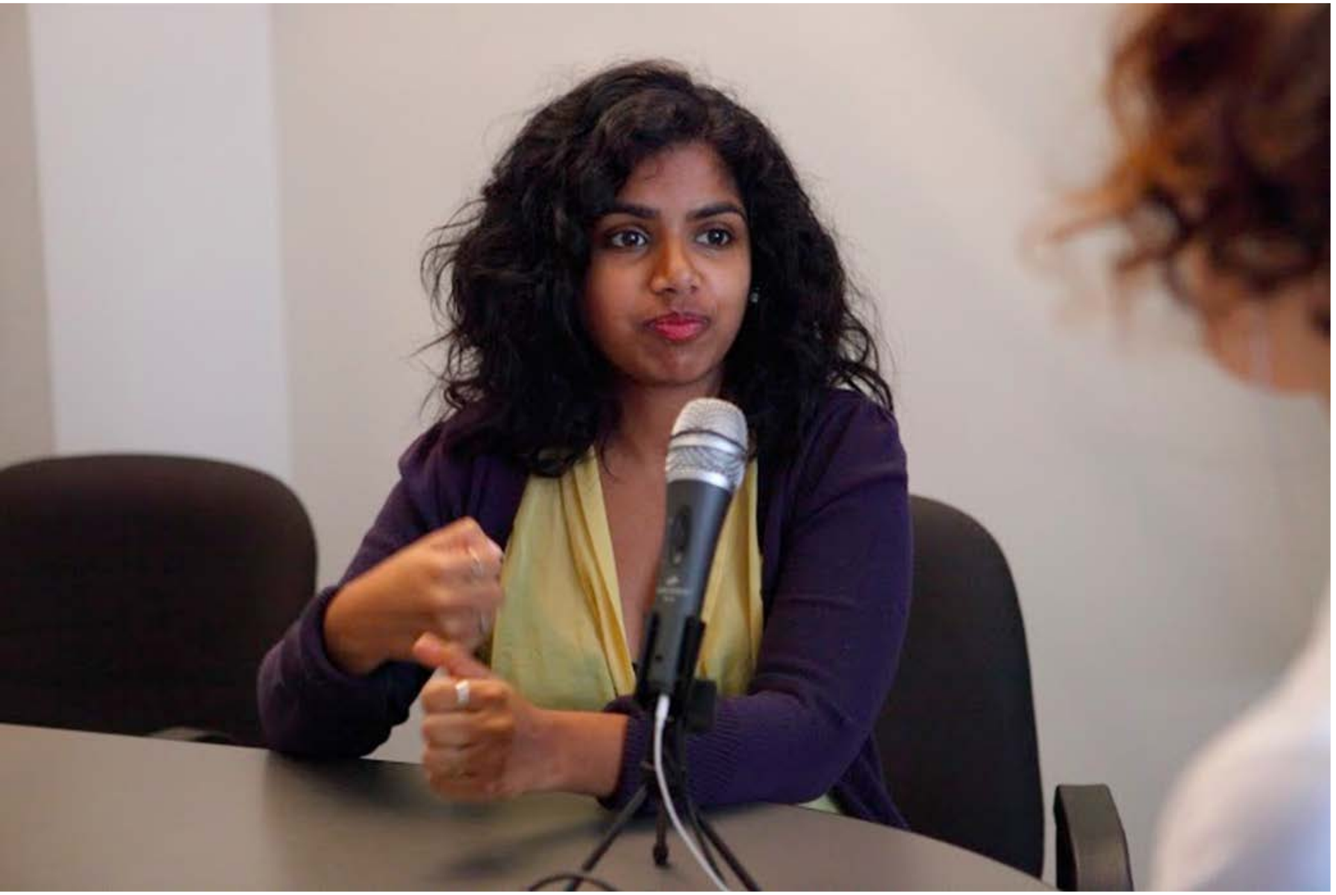

- Interview tips
- Technical considerations
- Common challenges

Make yourselves comfortable

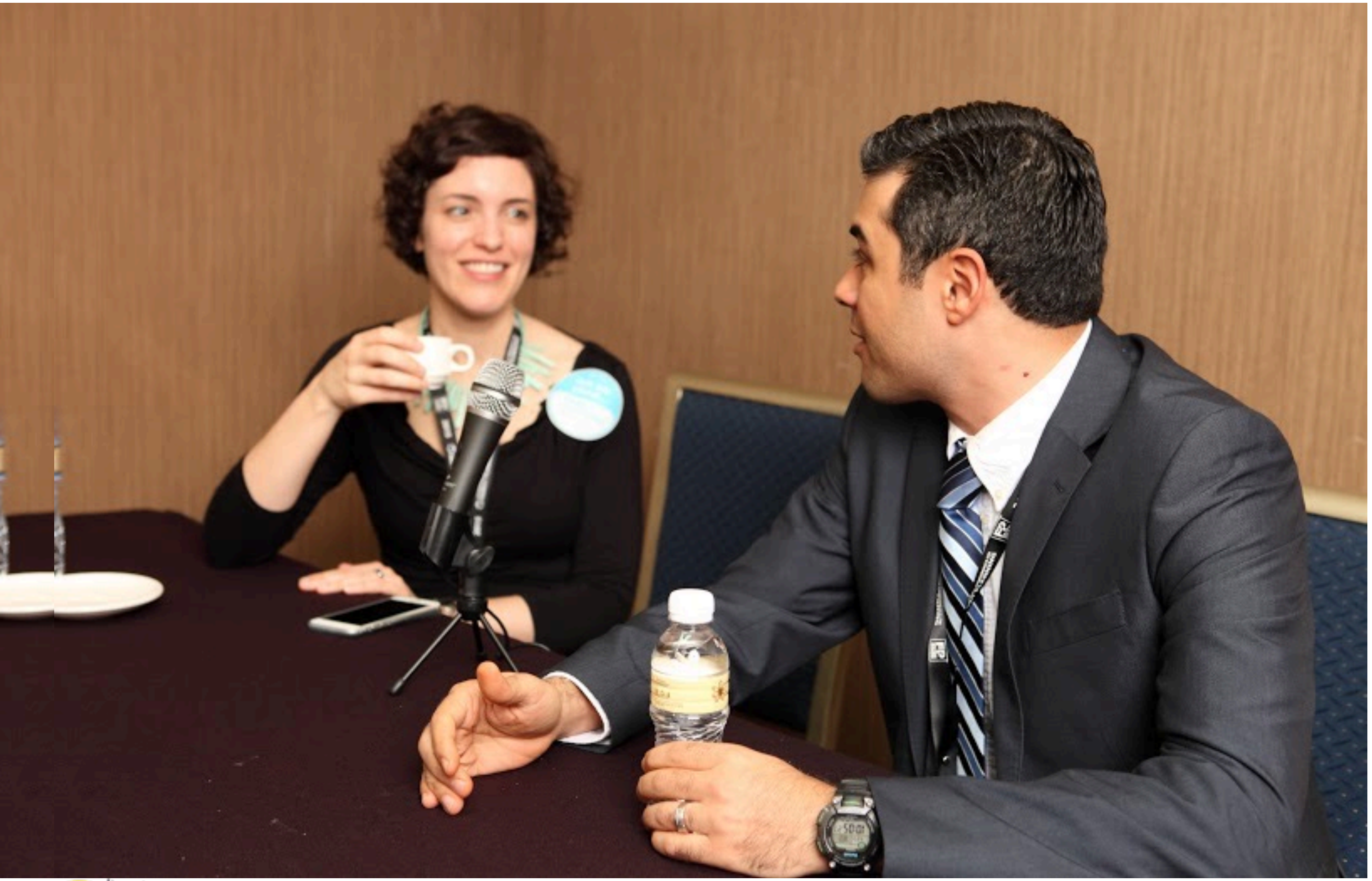

- Calm
- Eye contact
- Sincerity
- Privacy

Be prepared but flexible

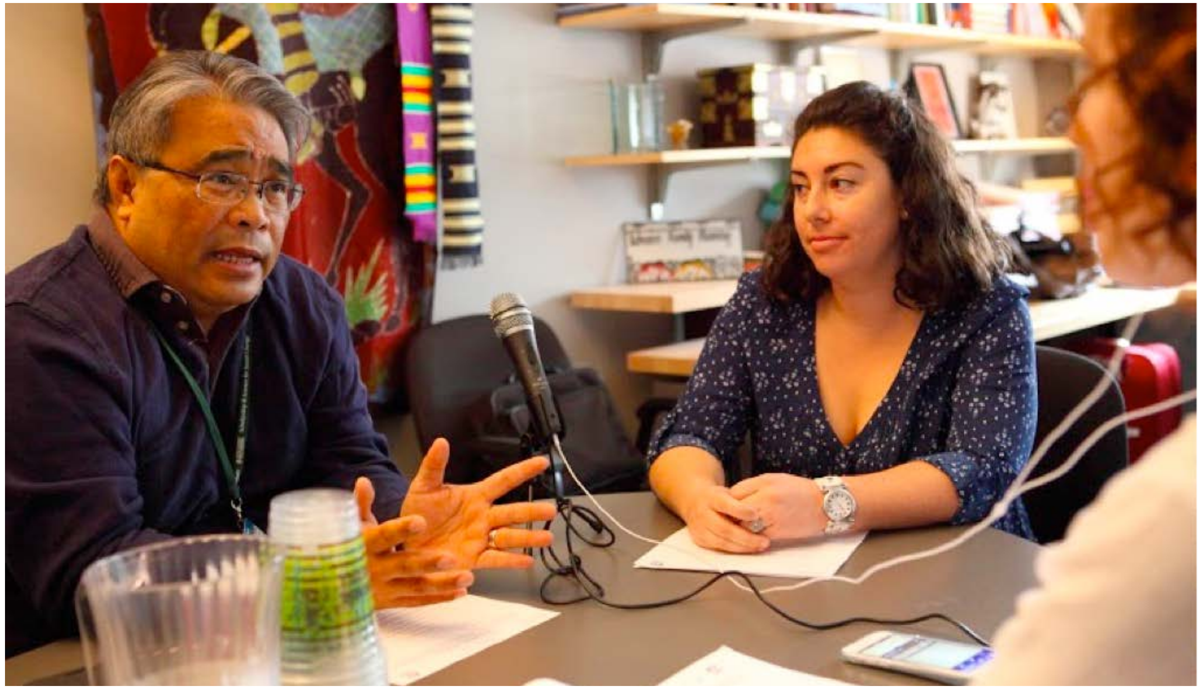

- Prepare
- Be open minded, but focused
- Listen, actively!

Ethical Interviewing: Protect & Respect

- Obtain consent
- Respect for personal boundaries and cultural norms
- Honesty and accuracy

- *What do you do if a storyteller identifies or describes someone in their story who could easily be identified?*

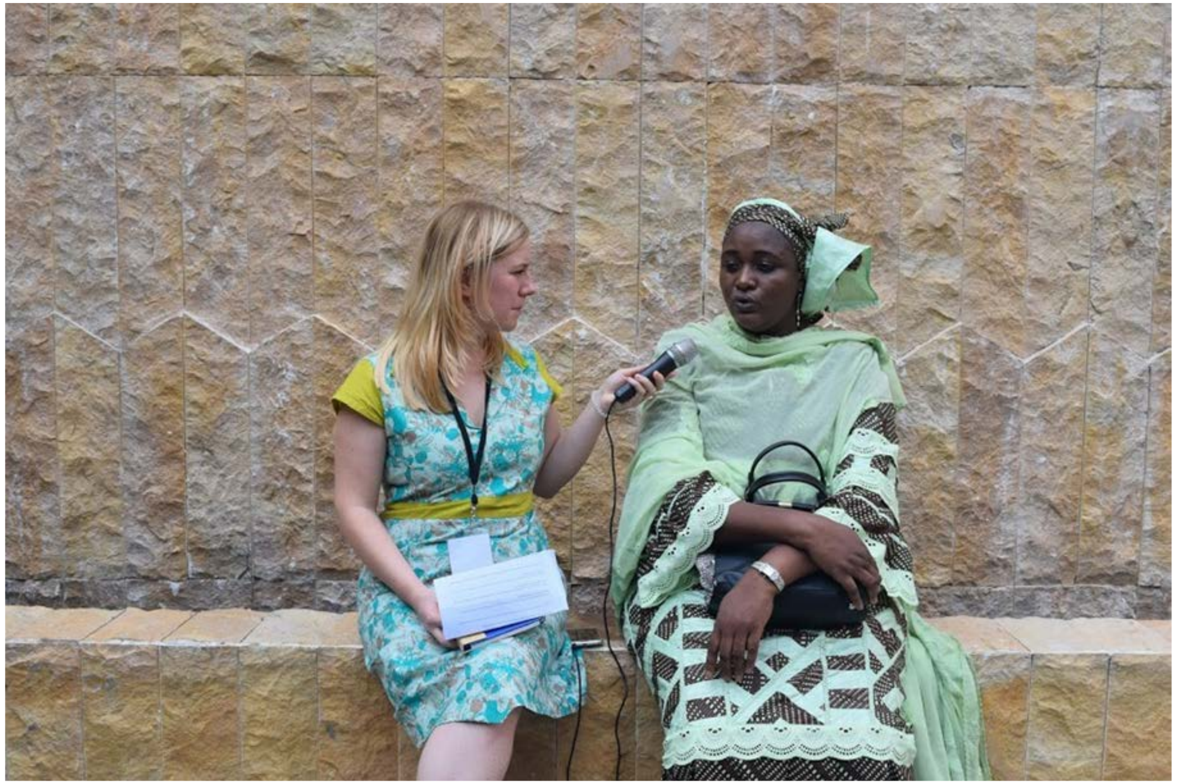

Technical Considerations

Hardware

- Mobile phone
- Hand-held microphone & tripod OR lapel microphones
- Ear buds OR noise-canceling headphones

Software

- Voice Record Pro
- Google Drive or Dropbox

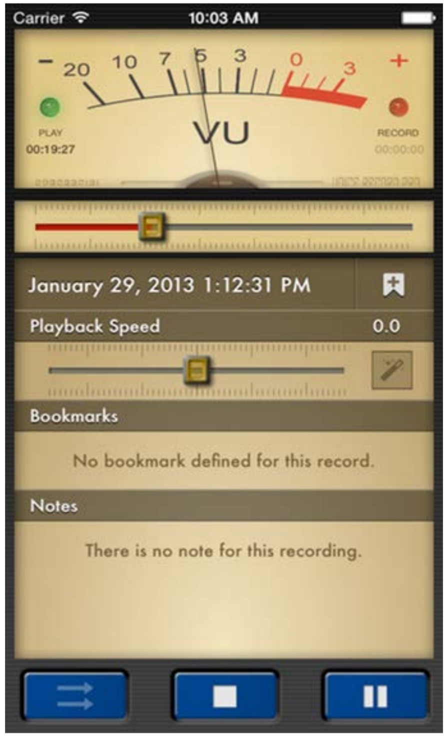

Audio Quality

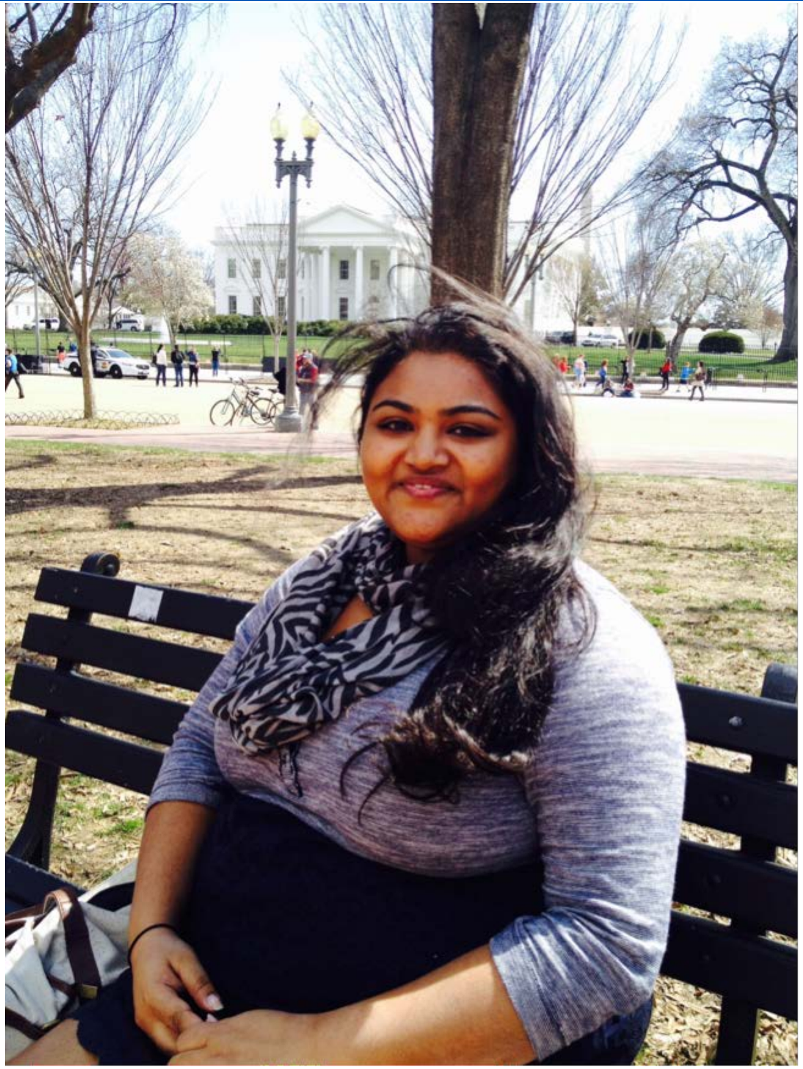

- Location, location, location!
- Microphones matter

Transcribing content

tran·scribe

/tran(t)ˈskrīb/

verb

put (thoughts, speech, or data) into written or printed form.
"each interview was taped and transcribed"

- Keep it simple.
- Apps or software can help.
- Transcribe key passages only.
- Edit to polish, but don’t change meaning.

Let’s Get Started!

Let’s review the interview guide together!

- We will practice in a moment but let’s first look over the guide and see how it’s structured and get familiar with its content

What’s in the Interview Guide?

- Reminder about what is in a story and purpose of collecting MSC stories
- Helpful tips when preparing for MSC story collection
- Interview guide to provide:
  1. Introduction or purpose of interview to read to the storyteller
  2. Consent form
  3. Information about the storyteller, when and where story took place
  4. Interview questions
- Audio recording guidance
- Story tracking
- Photography tips (which we will cover in detail later)

A good interviewer = a good researcher

If necessary, try a modified version of MSC and rephrase questions along these lines:

- How have you been involved in TCI?
- What are the important changes that have resulted from this initiative for you?
- What are the important changes that have occurred in the community as a result of this initiative?
- What problems were there?

**It can be helpful to probe on when the respondent first noticed the change was occurring so that it is not always just tied with an event or activity

MSC Interviewing Tips

- Always capture Who, What, When, Where, Why … and How
- Use probes to reduce assumptions
- When completed, each story will usually be less than 1 page, some may be up to 2 pages
- A story = the collective questions

Writing tips

- **Who** is the storyteller? Describe their occupation, age, background (complete the Demographic Background Questions)
- **What** was the change as a result of TCI?
- **When** did the change occur?
- **Where** did the change occur? (Geography or more specifically at an outreach, facility appointment, etc.)
- **Why** is the change significant from the storyteller's point of view? What might be the impact beyond the storyteller?
- **How** did the change take place?

Role Play!

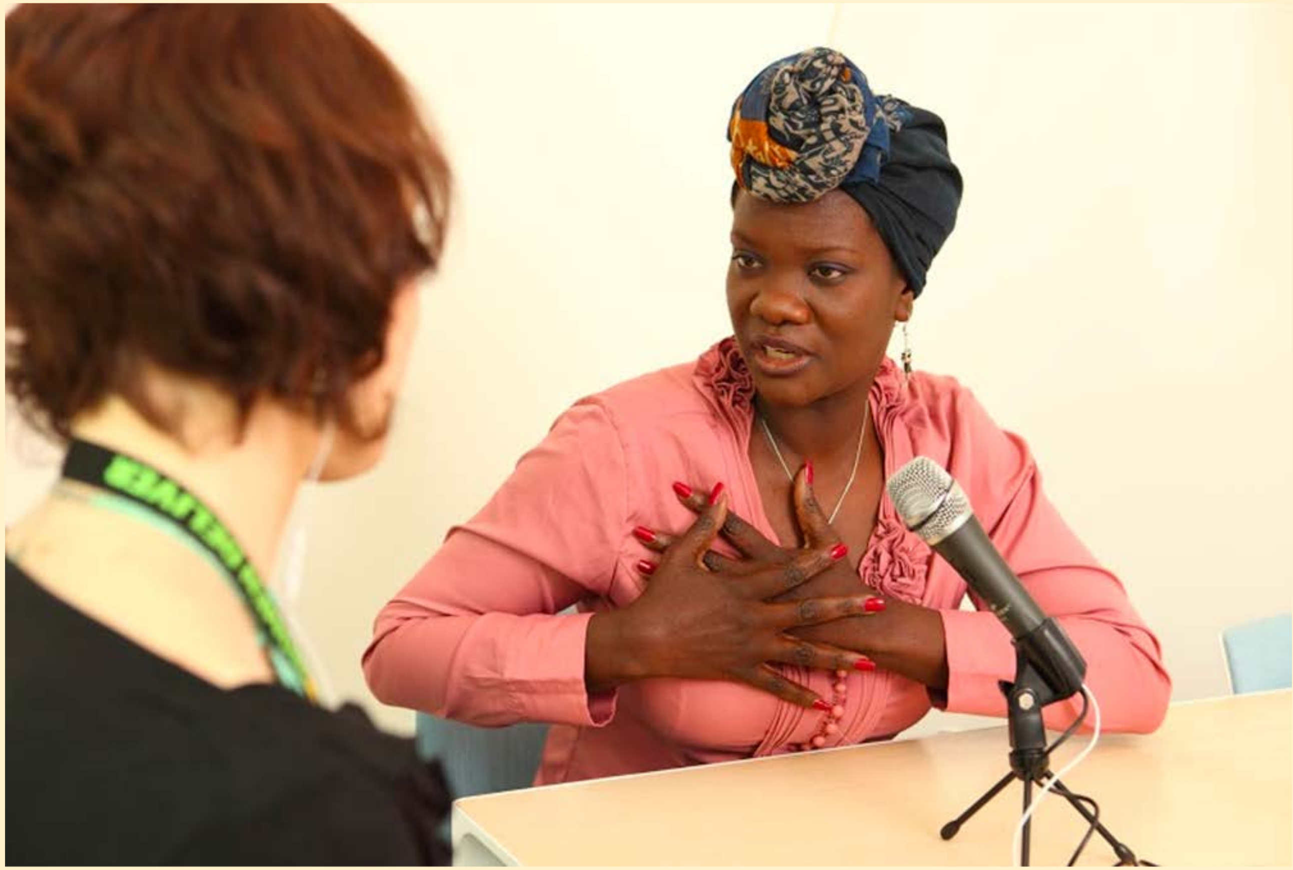

- Divide into pairs
- Take turns playing the role of interviewer and storyteller
- Everyone conducts the intro, consent, etc.
- Record each other and take notes
- Listen to the interview & pull out one direct quote that you think highlights the significance of the story told to you

How did your interview go?

- Did the questions make sense?
- Do you feel like you got a good story?
- What was the best part?
- What was the most challenging part?
- Do you have any questions?

Potential Challenges

Vocabulary/Phrasing

- Should we say “story” or “testimony/narrative”?
  - It will likely depend on your interviewee and what you sense they will be more responsive to
- What do you do if the question doesn’t quite “work” or resonate with the storyteller?
  - Remember, you can rephrase the question like we recommended earlier

What if there is “no change”?

- Change is taking place all the time, so it is never true to say that nothing has changed.
- Not all changes are big or absolute, so let’s take a closer look.
  - Ask respondents to look for any changes at all and then to identify those they think are the most significant, in relative terms, of all the changes they have noted.

Resistance

You're interviewing an implementer in the field who is in a senior position.

This person is very formal and determined to stick to talking points. She seems unwilling to open up, share her personal perspective, or tell stories.

How do you get a story?

Agenda pushing

You're interviewing someone who brings an important perspective to implementing TCI.

However, rather than engaging in a dialogue, answering your questions, and focusing on the purpose of the initiative, this person launches into a monologue about a sensitive or unrelated issue.

What do you do?

Seeing the Story

Photography tips and techniques for visual storytelling

Photography's Role

- promote mission
- document local issues
- context is critical for engaging viewers
- remind funders/supporters of the value, benefit, and reach

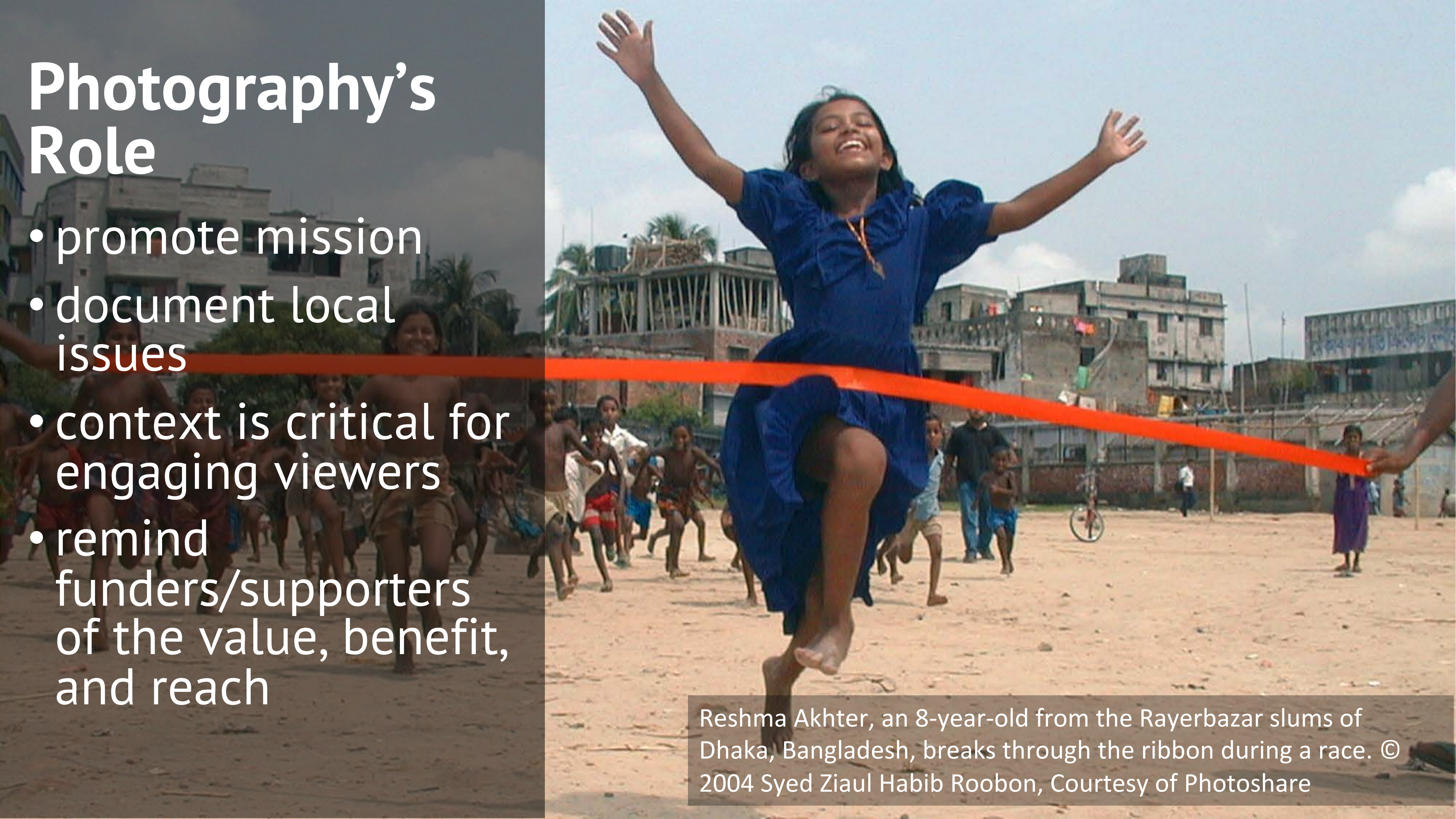

Reshma Akhter, an 8-year-old from the Rayerbazar slums of Dhaka, Bangladesh, breaks through the ribbon during a race. © 2004 Syed Ziaul Habib Roobon, Courtesy of Photoshare

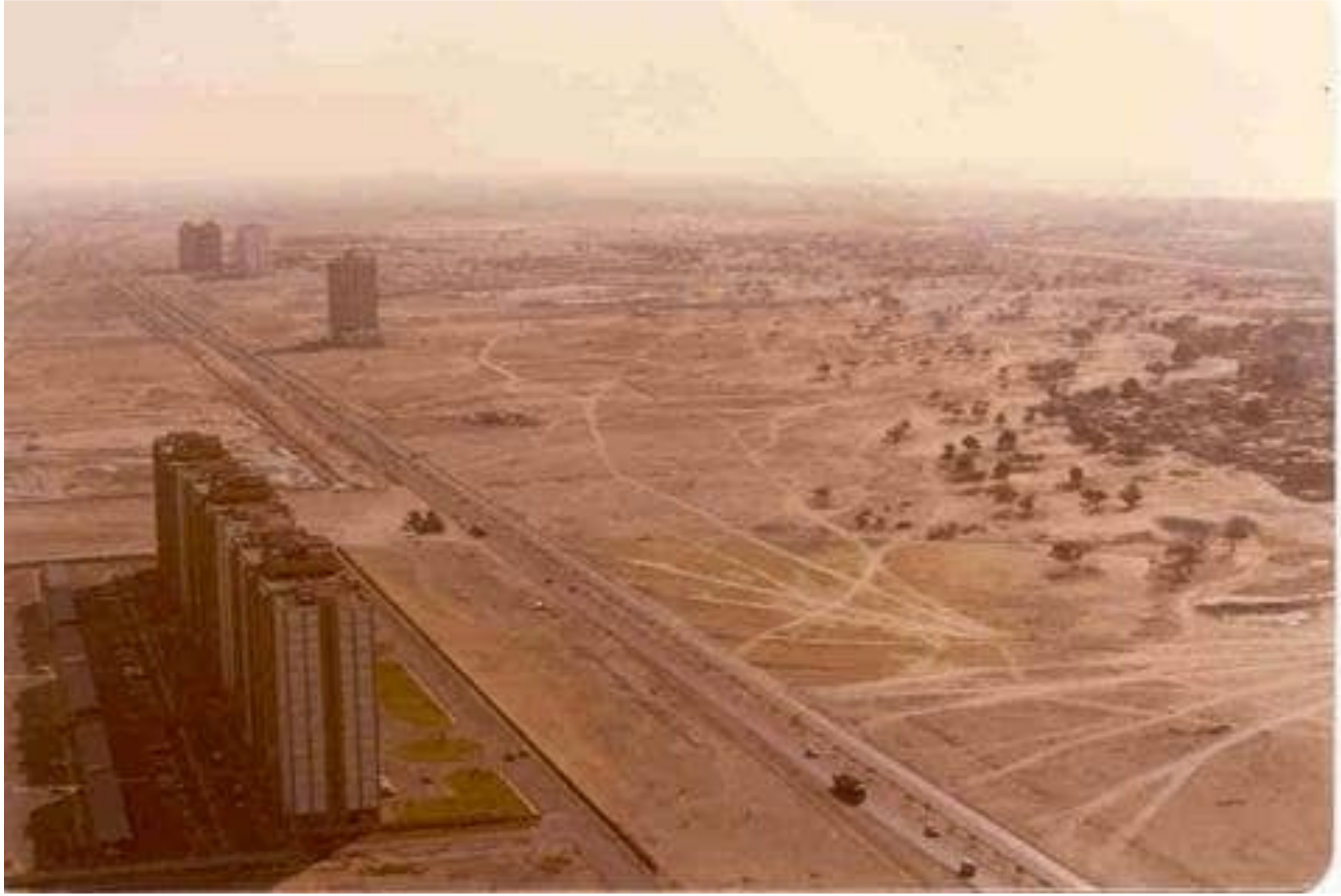

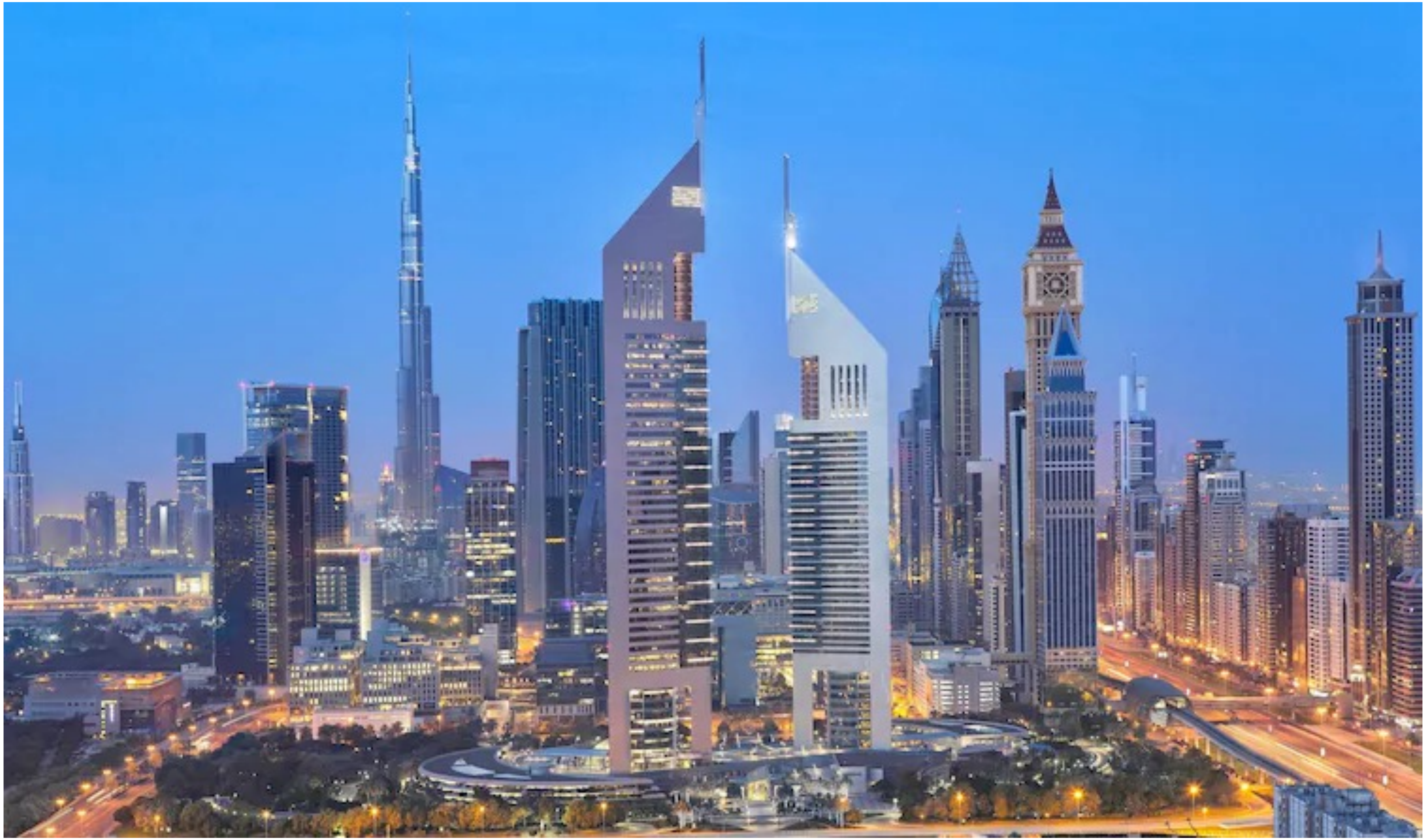

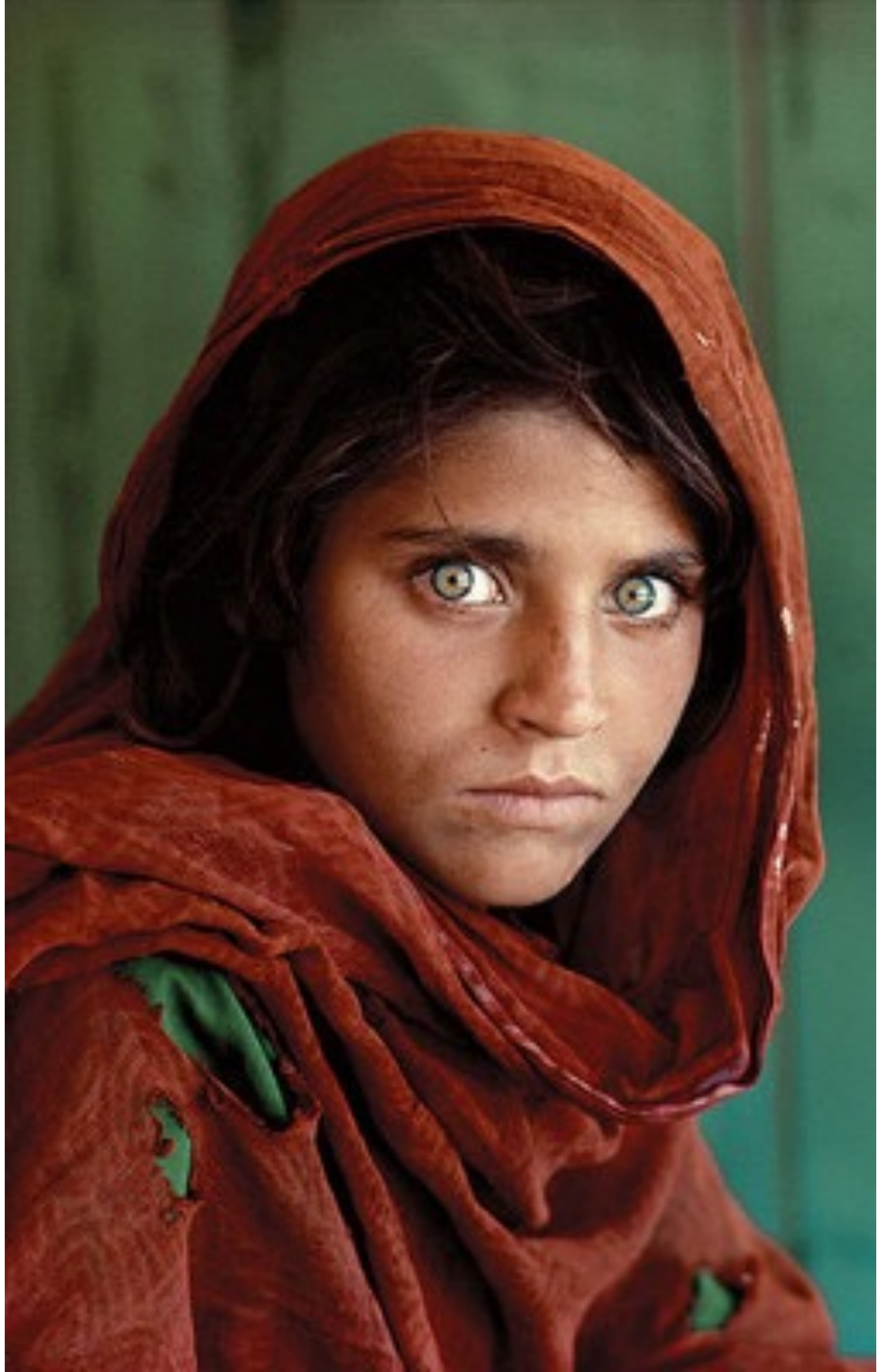

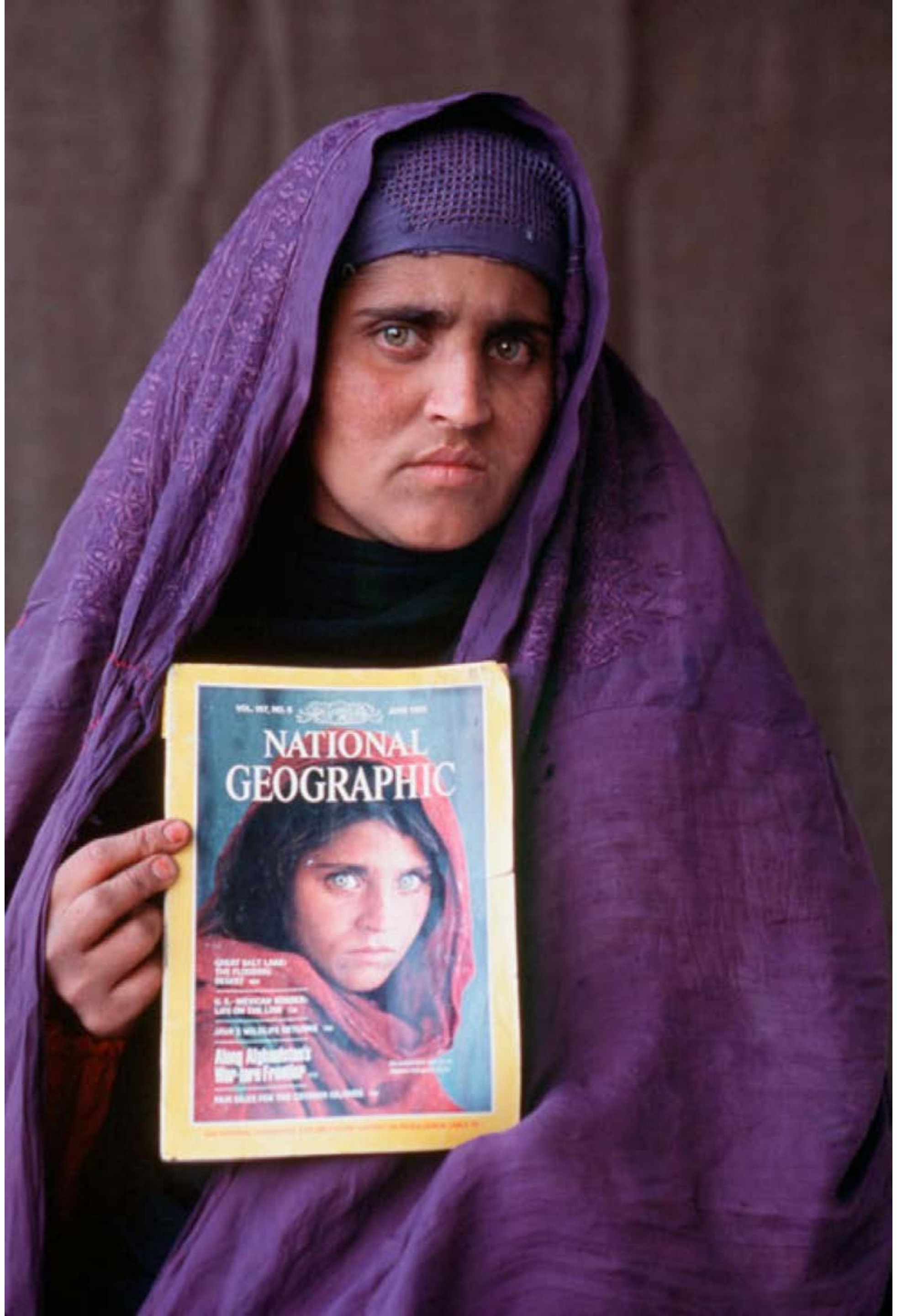
NATIONAL
GEOGRAPHIC

Make a Photography Move

- respond to the light
- explore the composition
- Use depth of field to focus attention

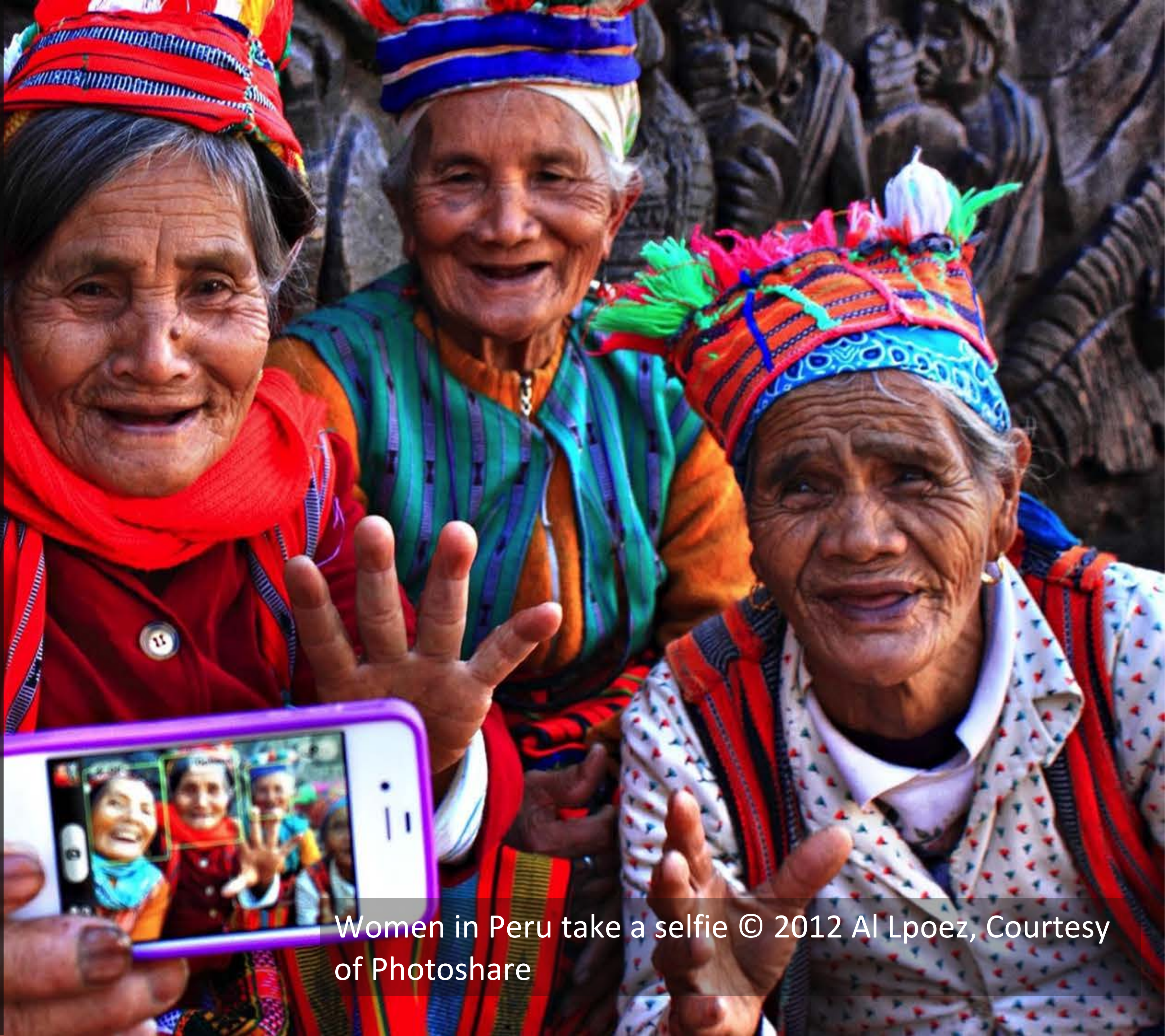

Women in Peru take a selfie © 2012 Al Lpoez, Courtesy of Photoshare

Where is the Light?

- avoid mid-day
- fill flash, reflector, shade
- overcast– no harsh shadows, no squinting

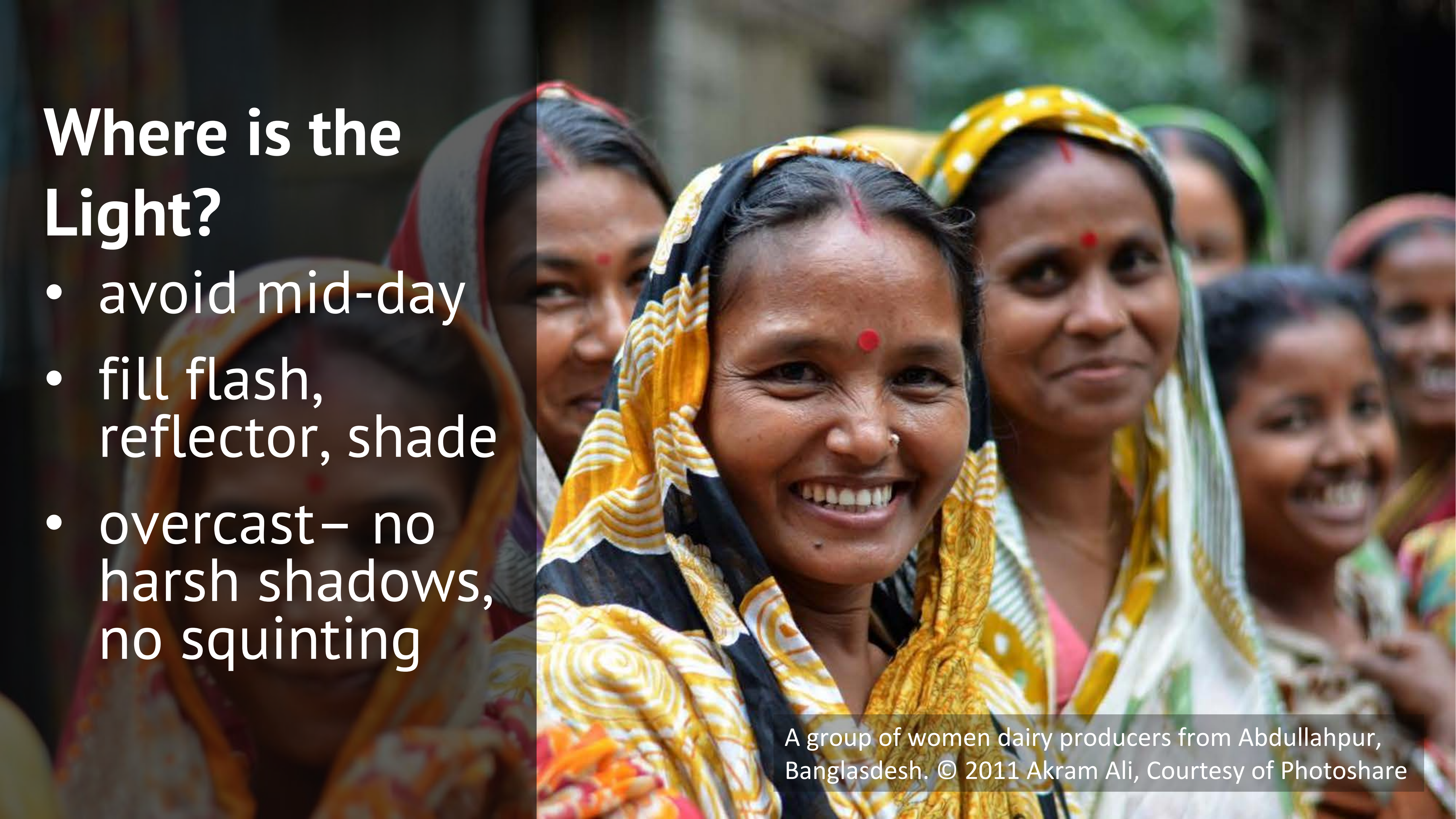

A group of women dairy producers from Abdullahpur, Banglasdesh. © 2011 Akram Ali, Courtesy of Photoshare

Control the light

- protect sensitive populations
- a medium - painting with light

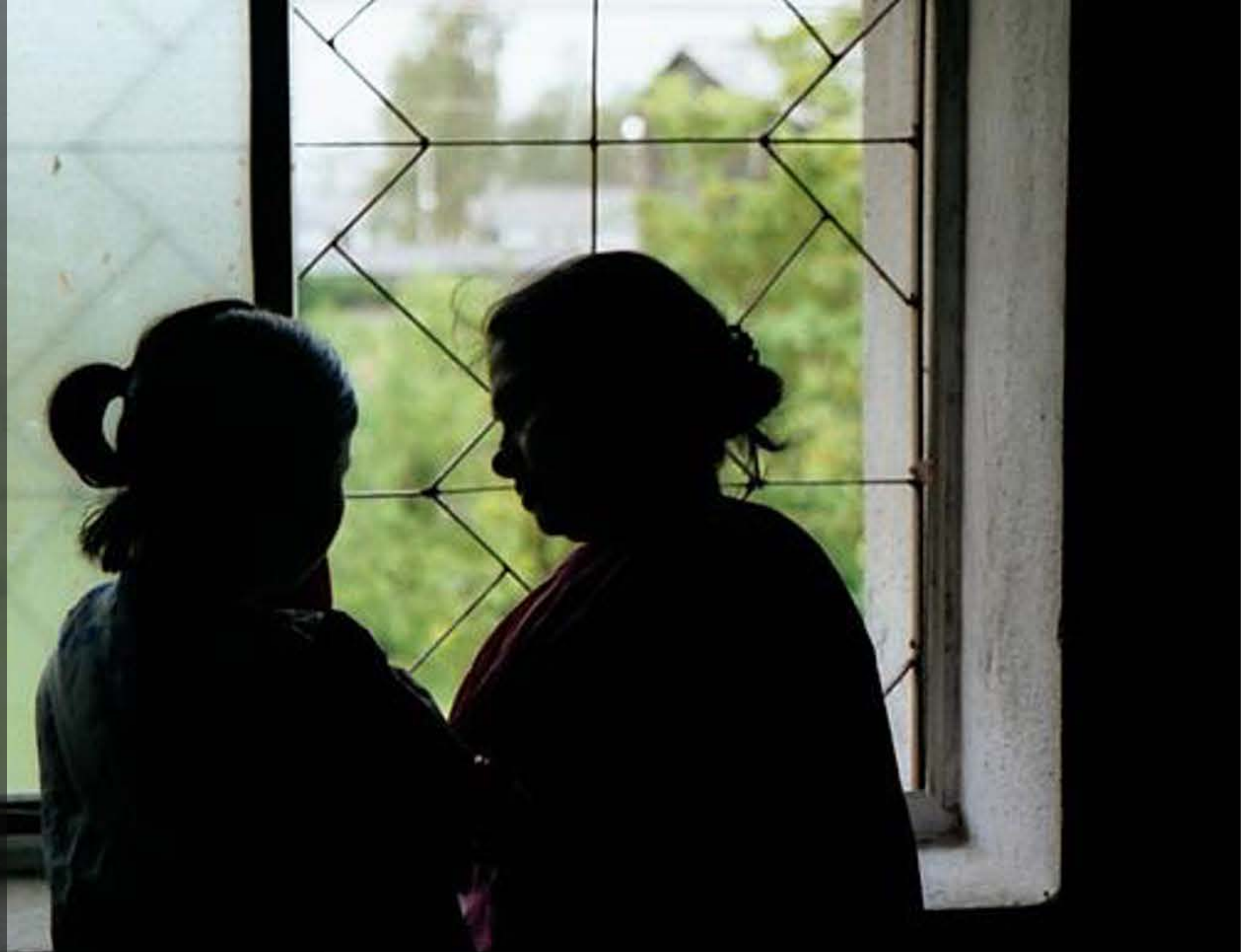

A center in Mumbai, India helps children of sex workers.
© 2002 Vijay Sureshkumar, Courtesy of Photoshare

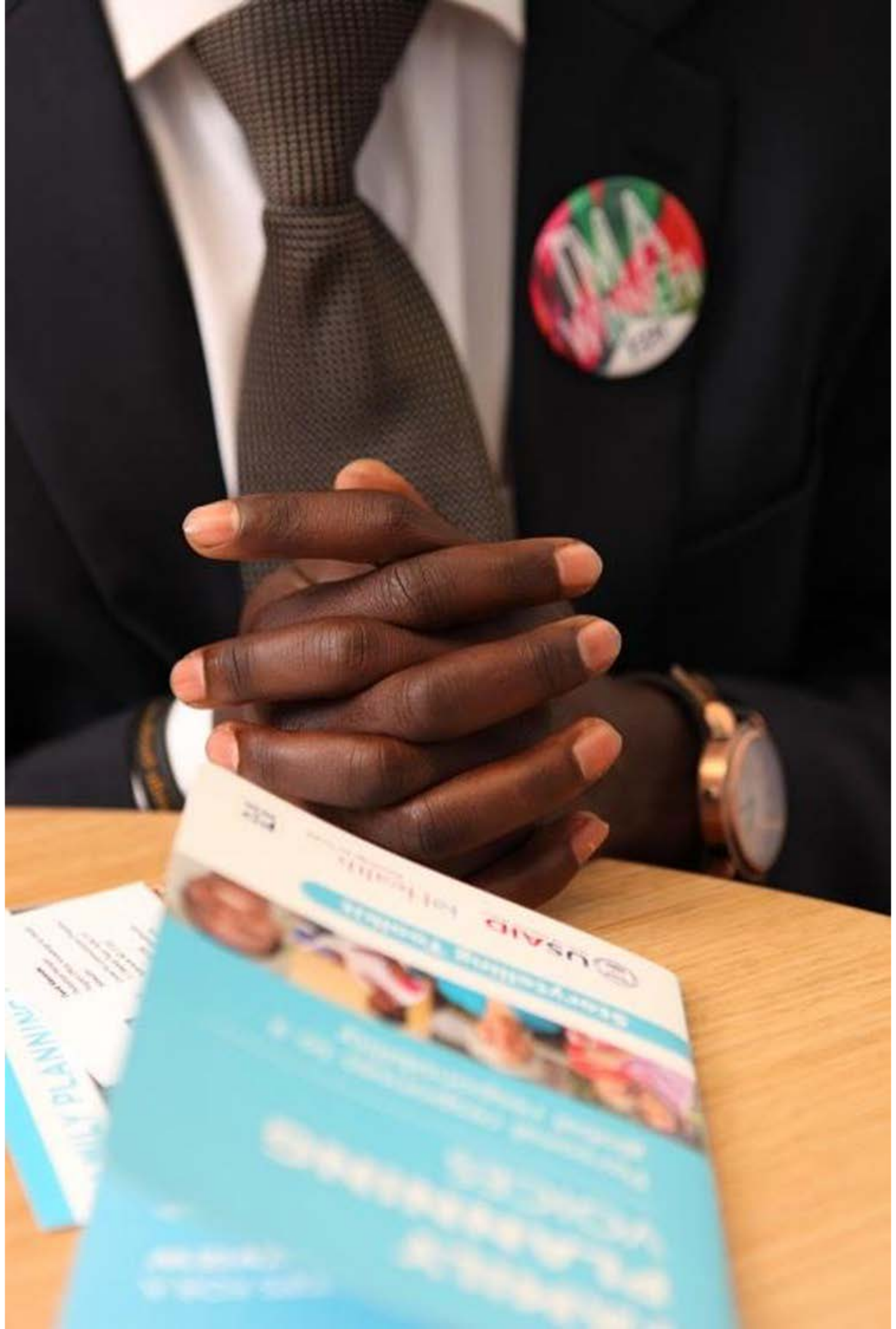
USAID

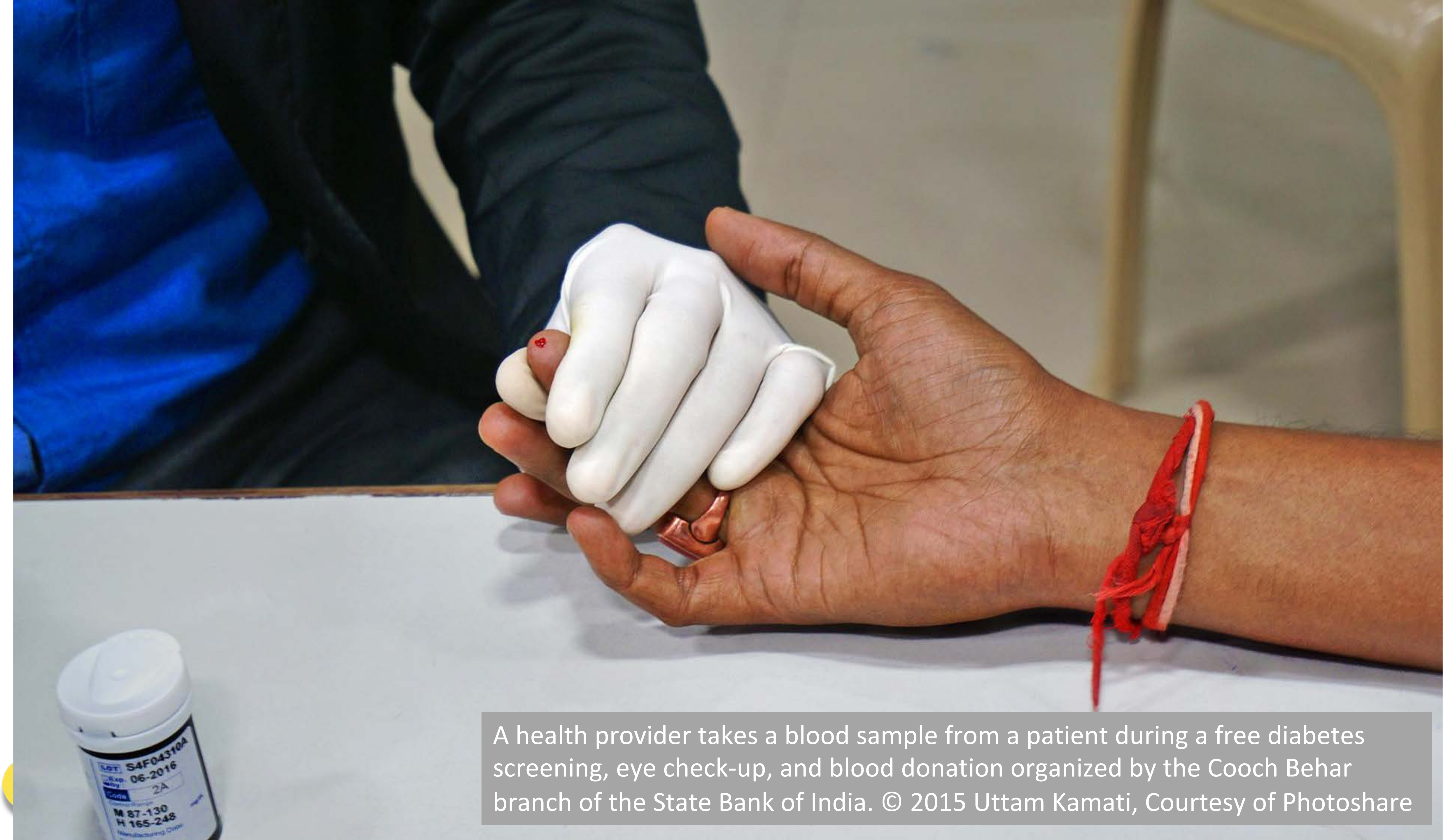

A health provider takes a blood sample from a patient during a free diabetes screening, eye check-up, and blood donation organized by the Cooch Behar branch of the State Bank of India. © 2015 Uttam Kamati, Courtesy of Photoshare

Composition

- dynamic view/action,
- find a low and high point, or unique angle

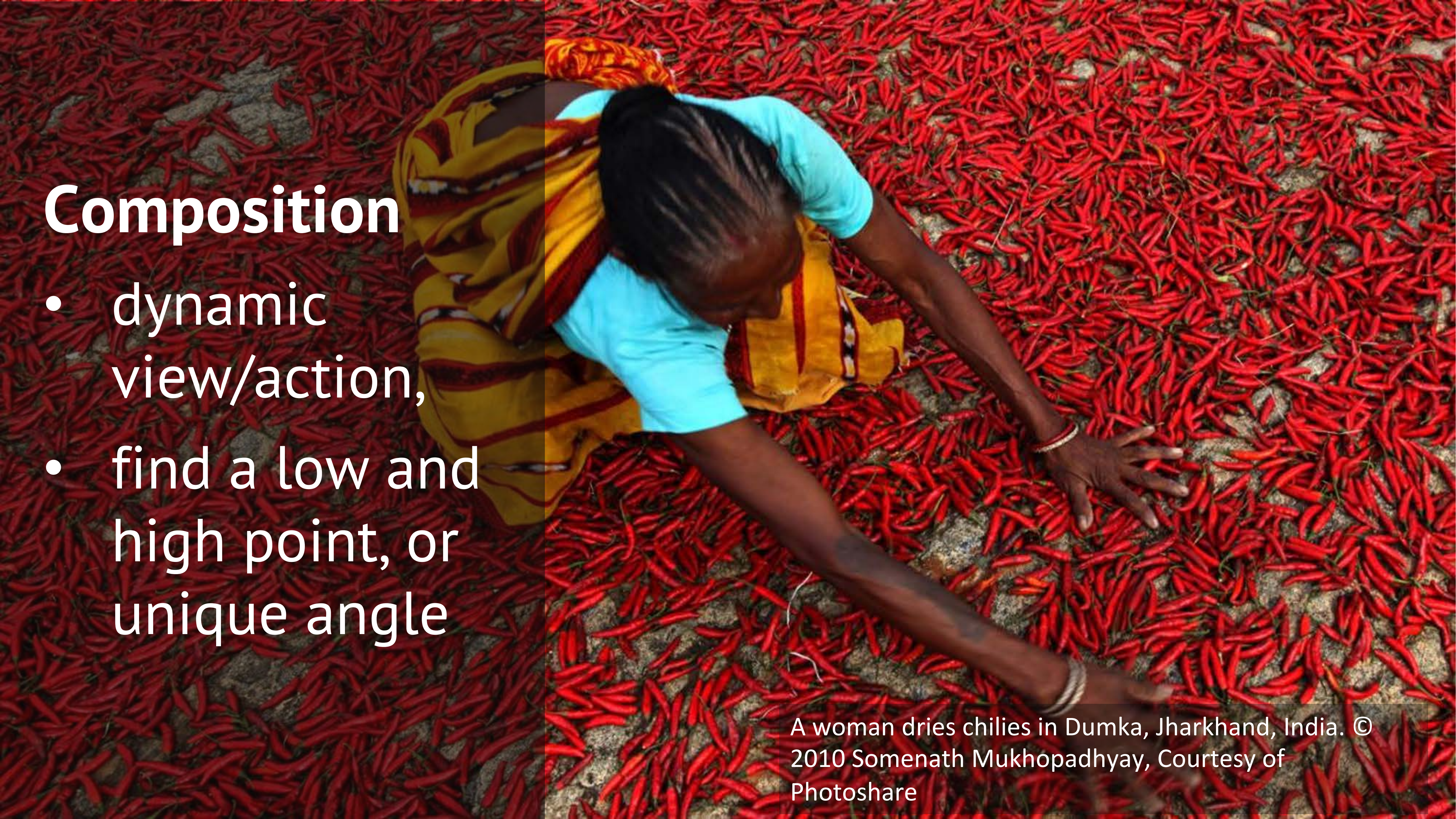

A woman dries chilies in Dumka, Jharkhand, India. © 2010 Somenath Mukhopadhyay, Courtesy of Photoshare

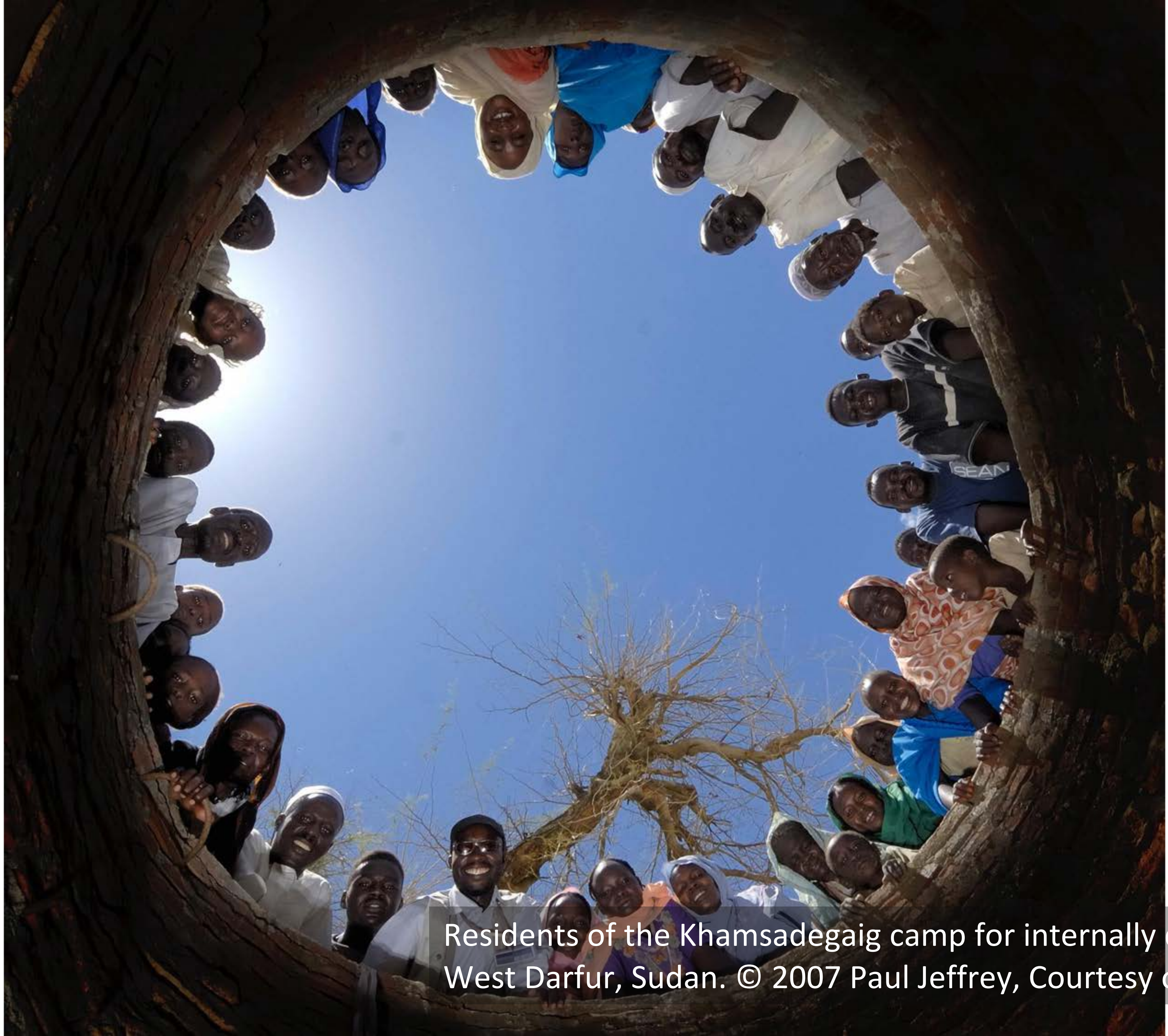

Residents of the Khamsadegaig camp for internally displaced families in West Darfur, Sudan. © 2007 Paul Jeffrey, Courtesy of Photoshare

Eye Level

- determine the eye level
- avoid distortions
- emphasize the subject

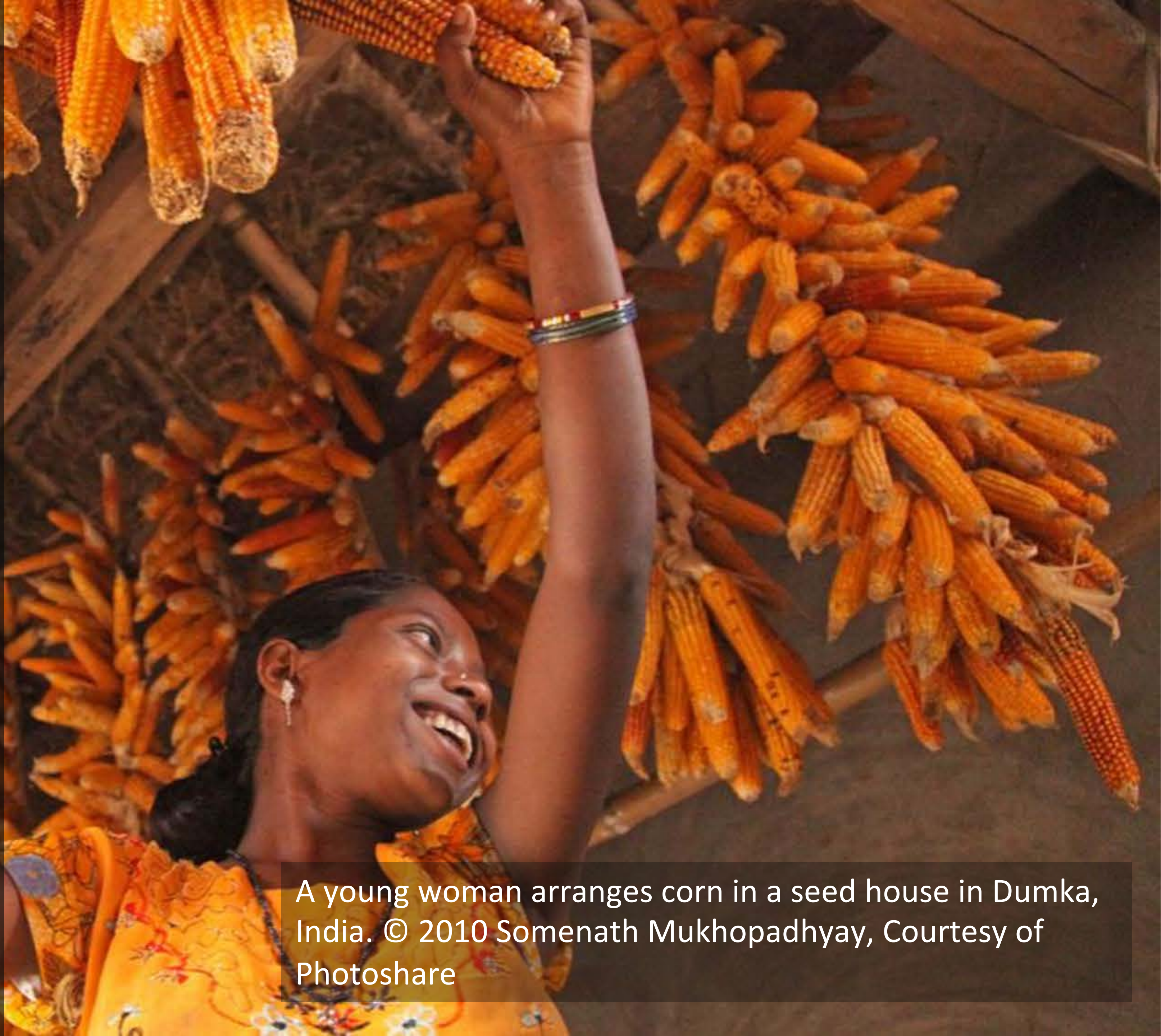

A young woman arranges corn in a seed house in Dumka, India. © 2010 Somenath Mukhopadhyay, Courtesy of Photoshare

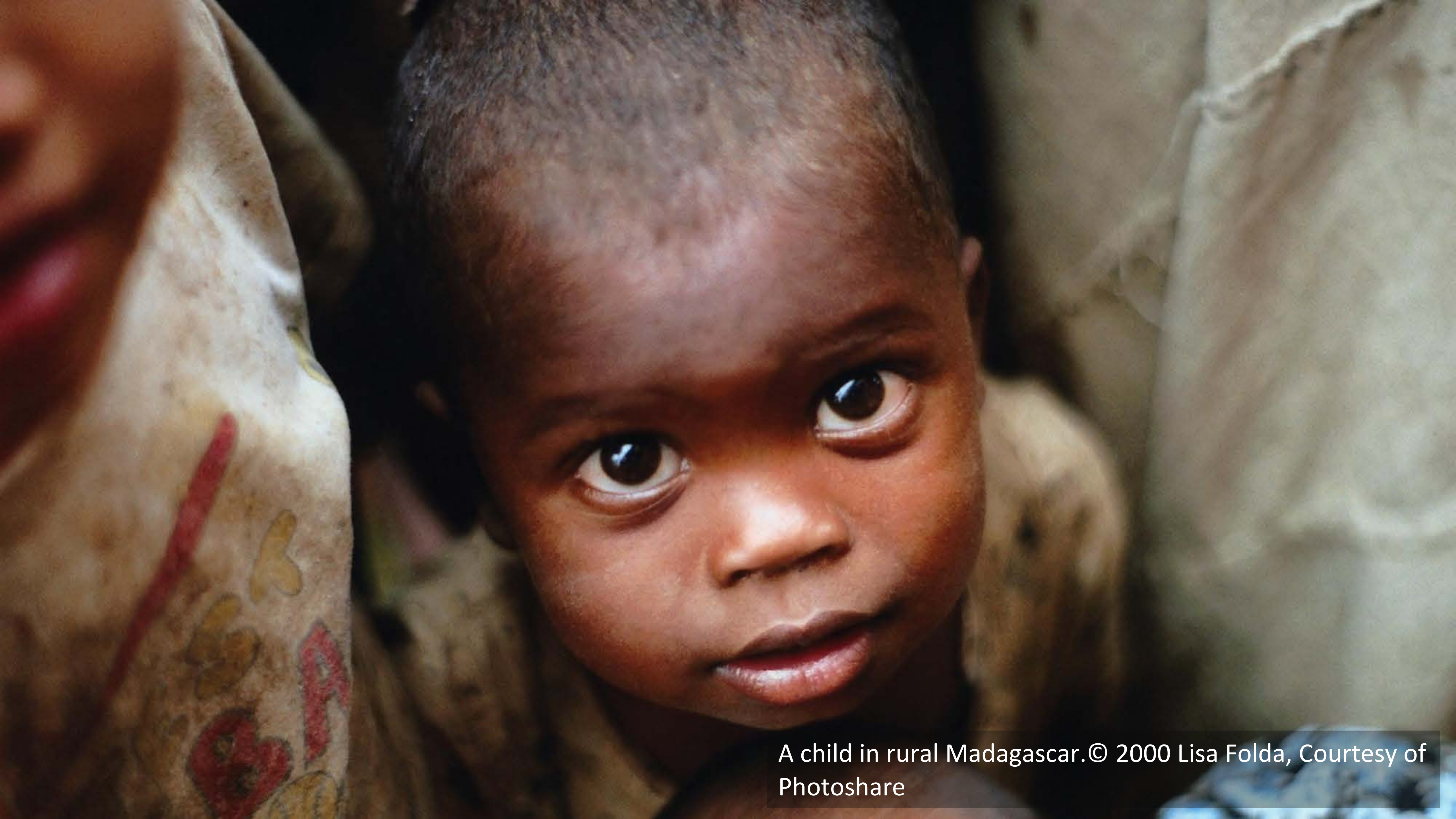

A child in rural Madagascar.© 2000 Lisa Folda, Courtesy of Photoshare

Get Closer

- fill the frame
- create connections and engagement

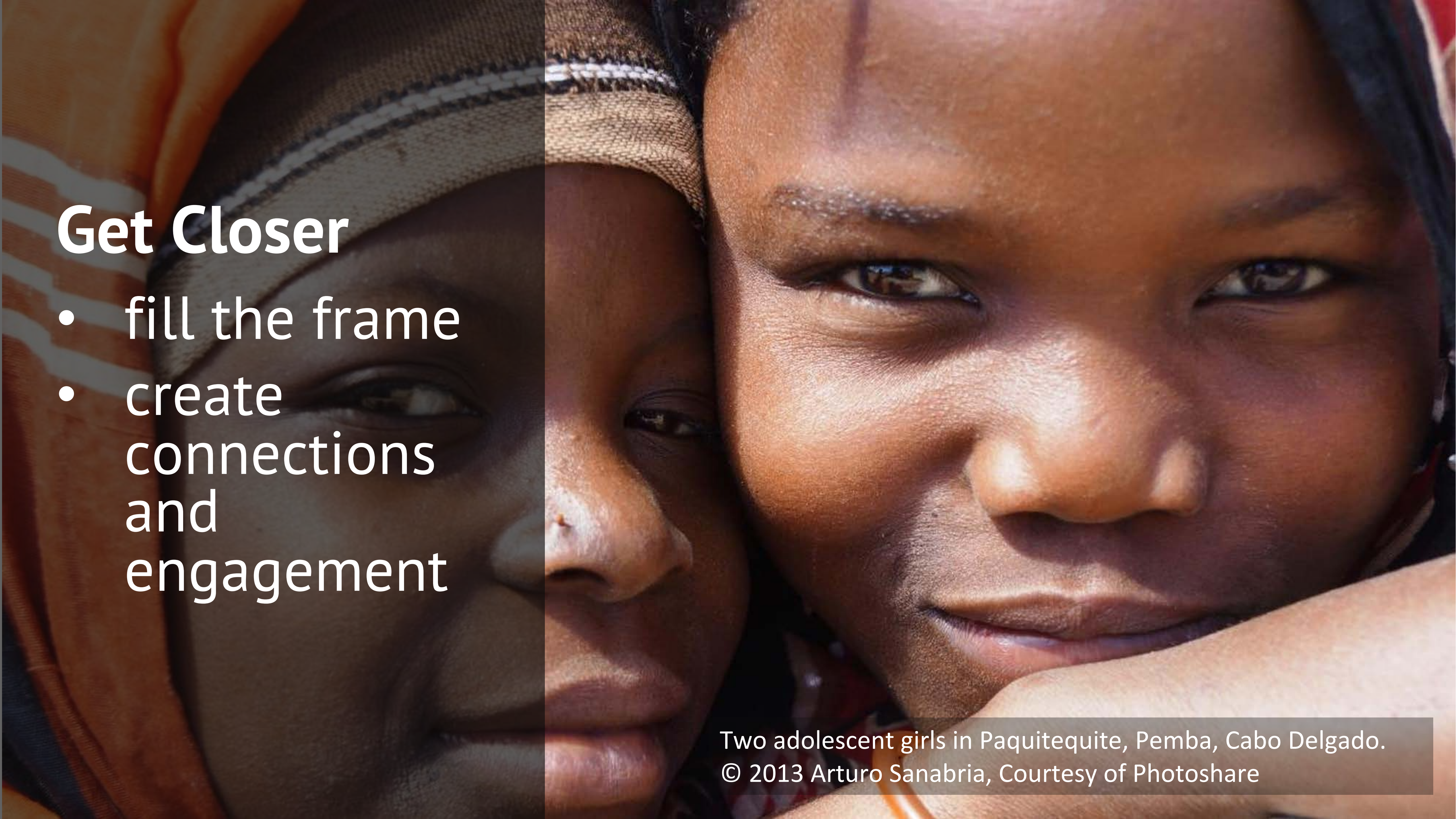

Two adolescent girls in Paquitequite, Pemba, Cabo Delgado.
© 2013 Arturo Sanabria, Courtesy of Photoshare

Rule of Thirds

- images most engaging when aligned along an imaginary grid

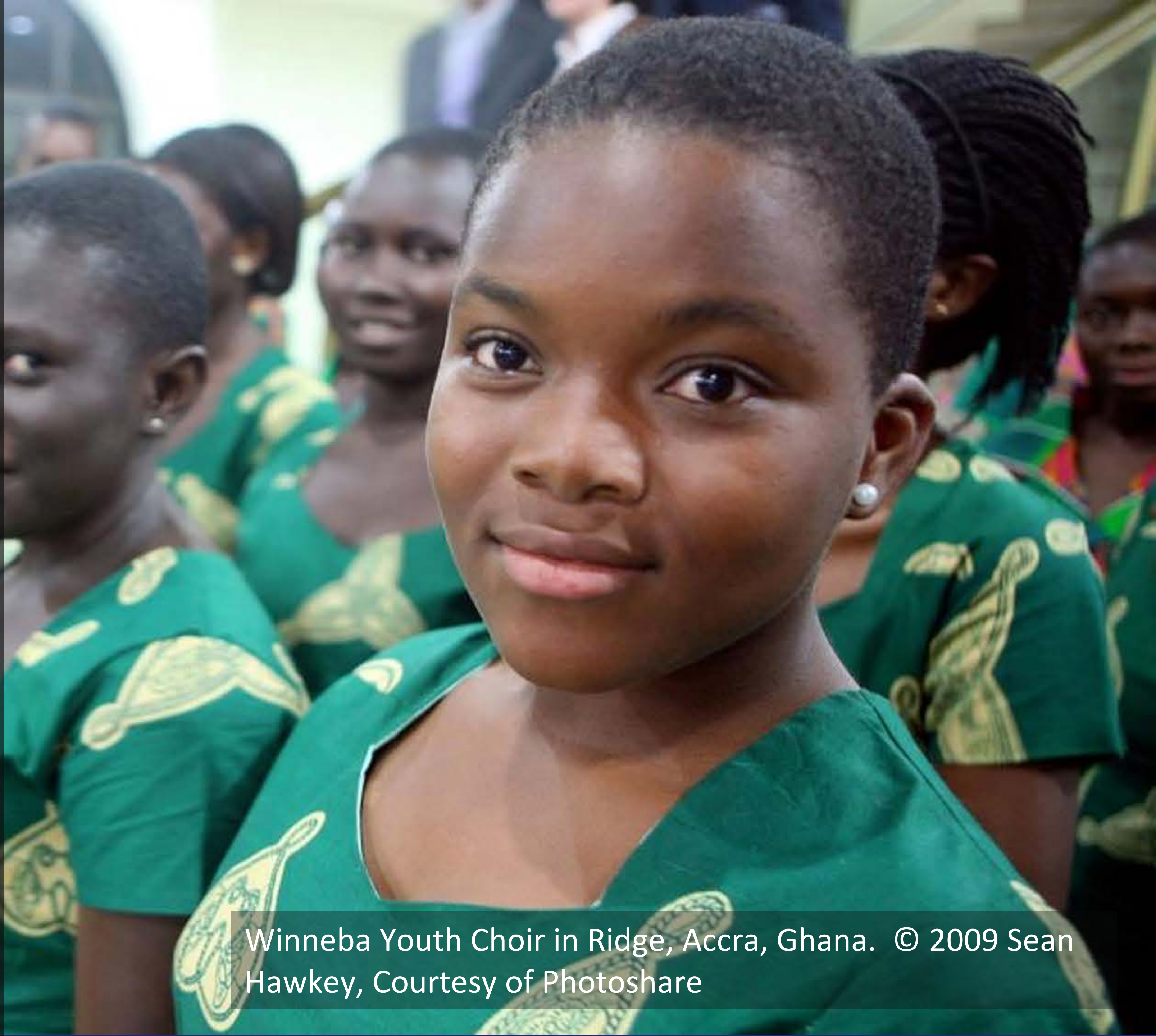

Winneba Youth Choir in Ridge, Accra, Ghana. © 2009 Sean Hawkey, Courtesy of Photoshare

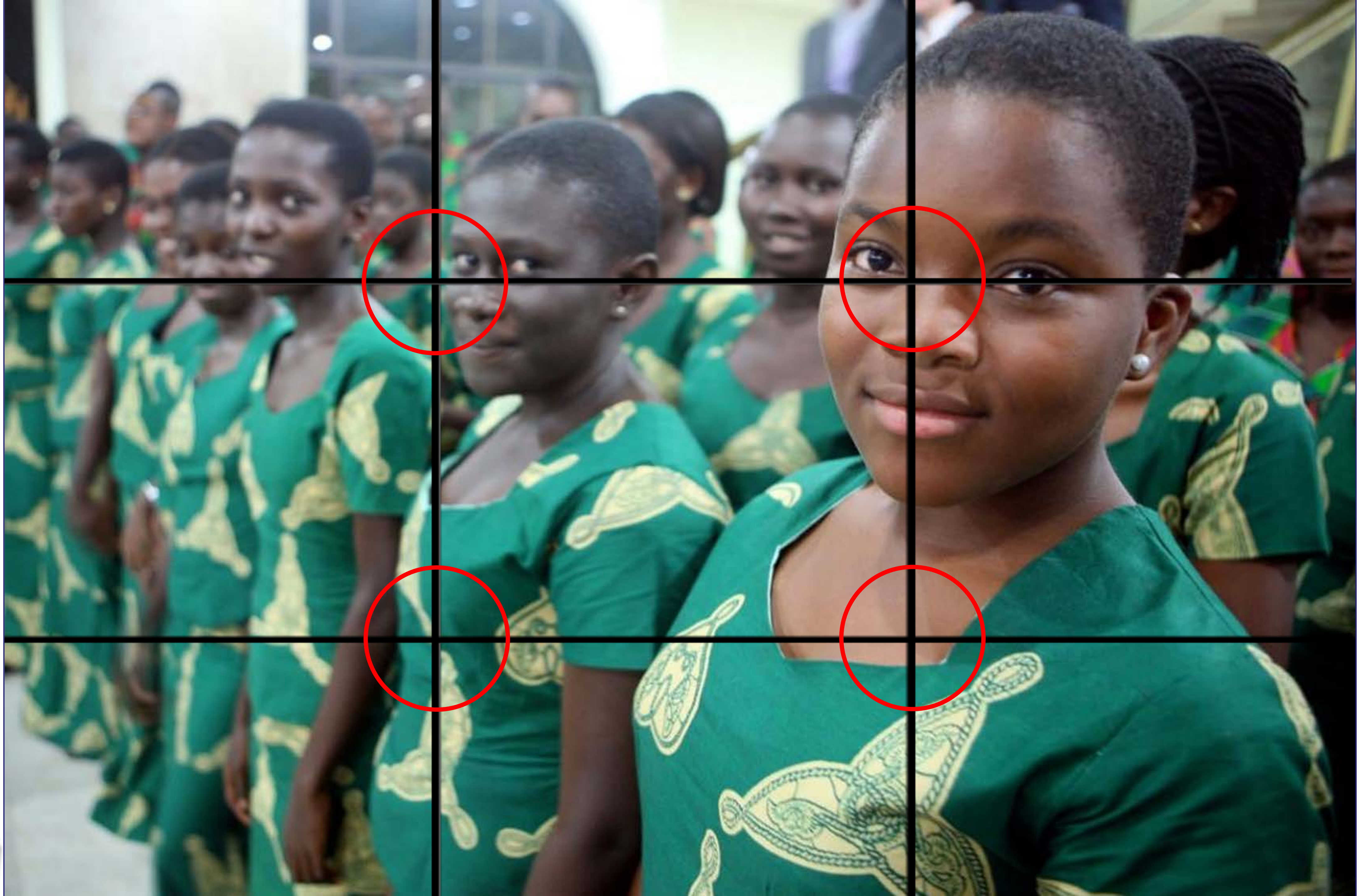

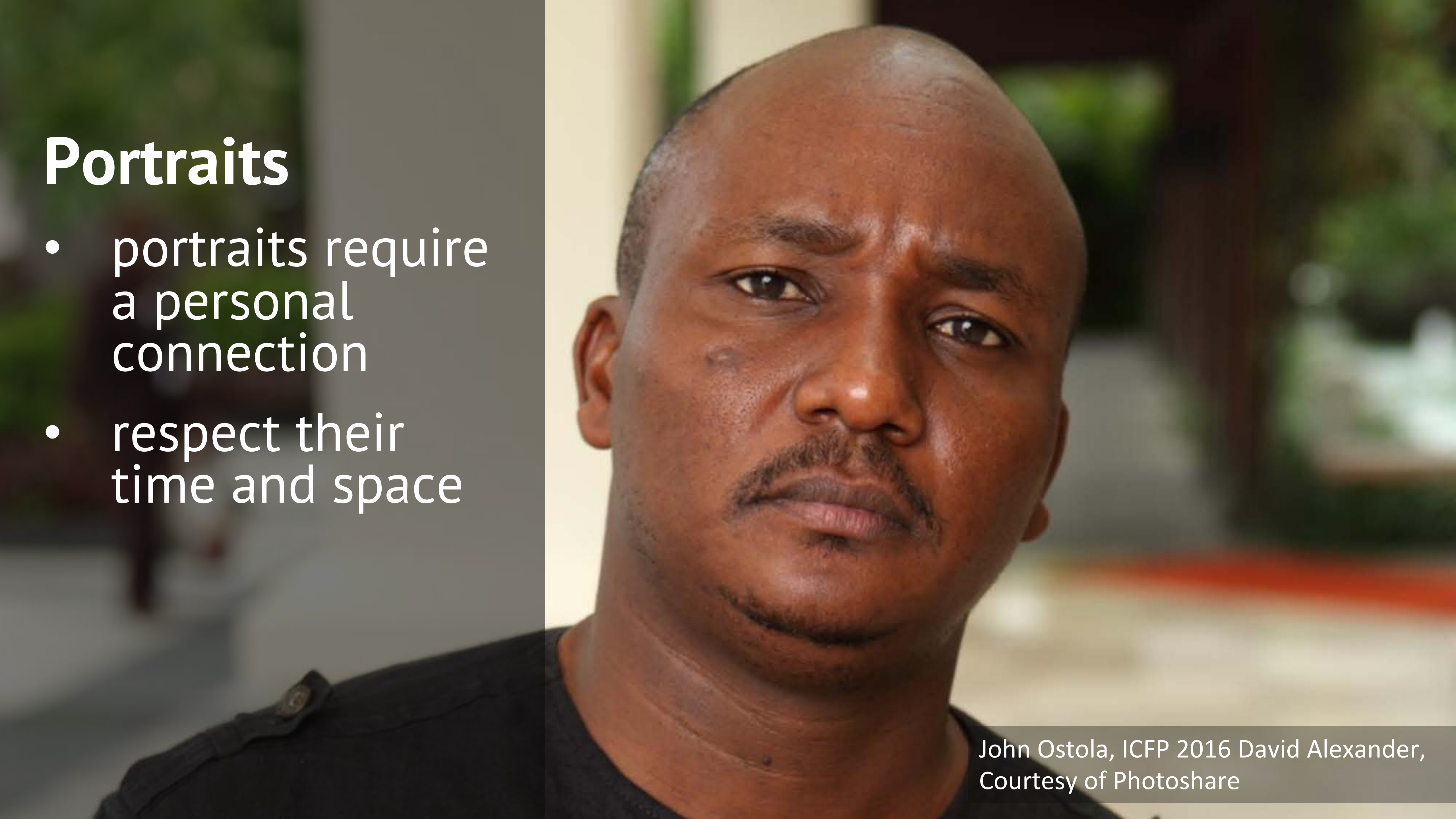

Portraits

- portraits require a personal connection
- respect their time and space

John Ostola, ICFP 2016 David Alexander, Courtesy of Photoshare

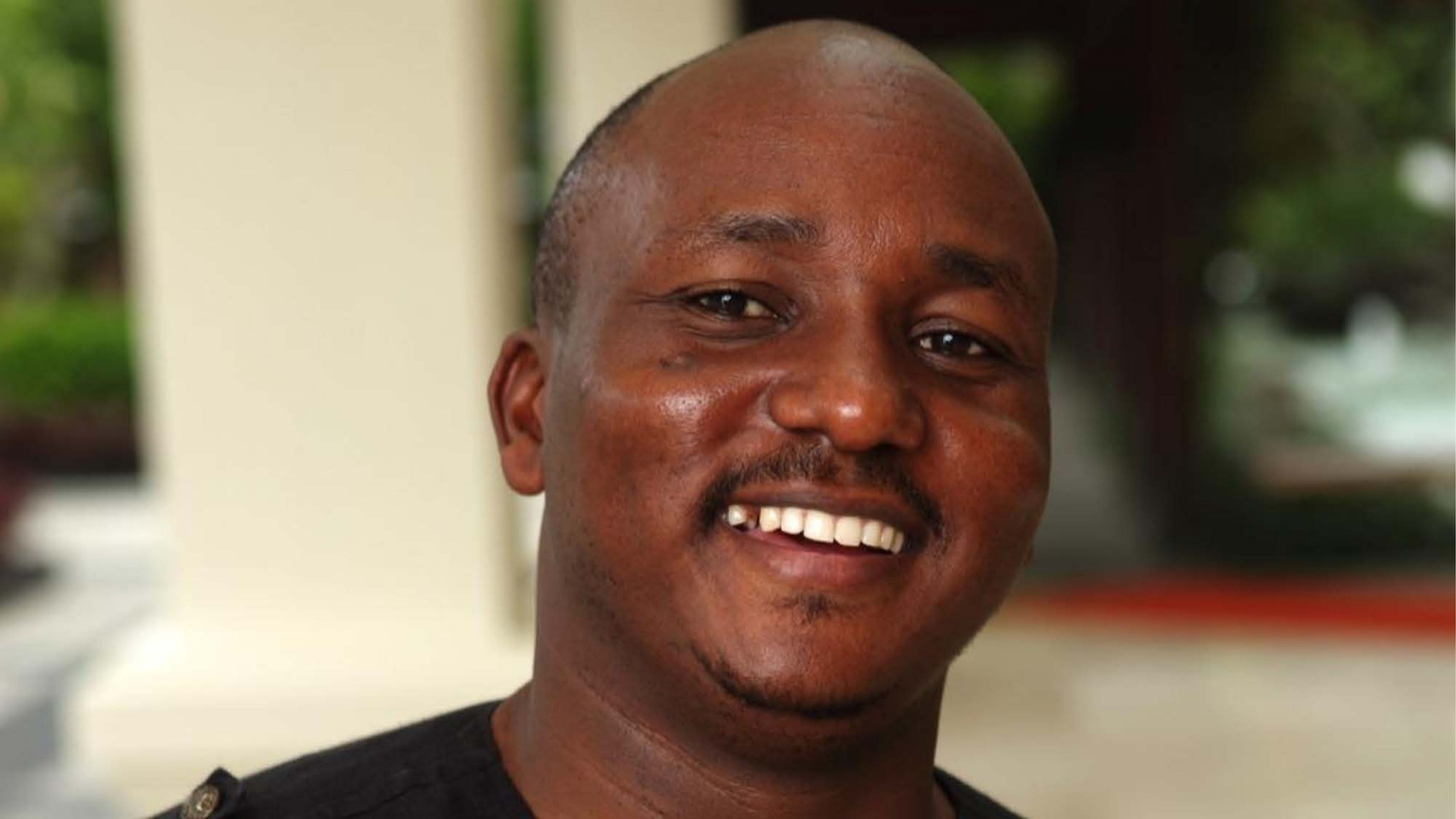

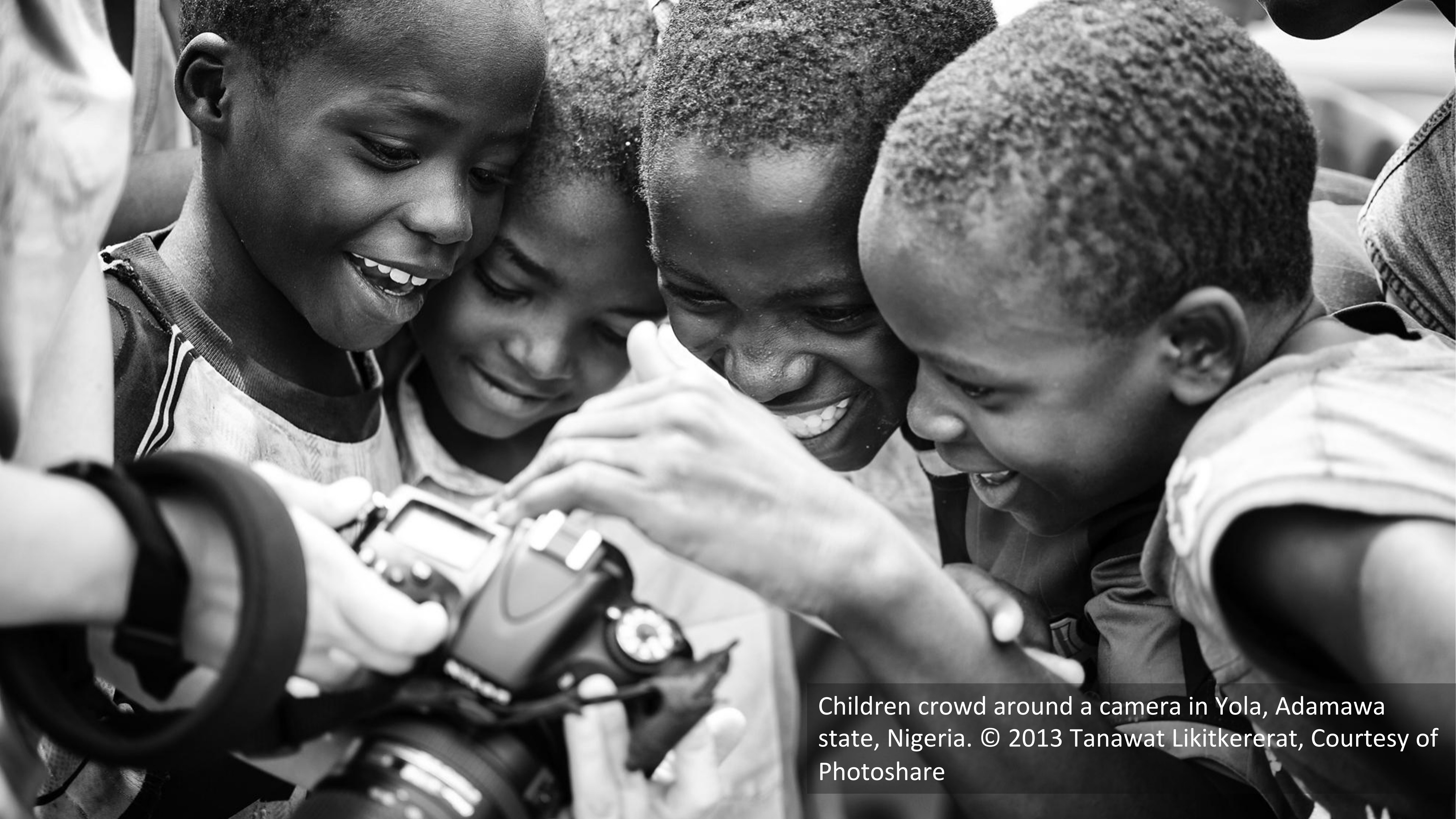

Children crowd around a camera in Yola, Adamawa state, Nigeria. © 2013 Tanawat Likitkererat, Courtesy of Photoshare

Backgrounds

- compliment the subject
- define the narrative

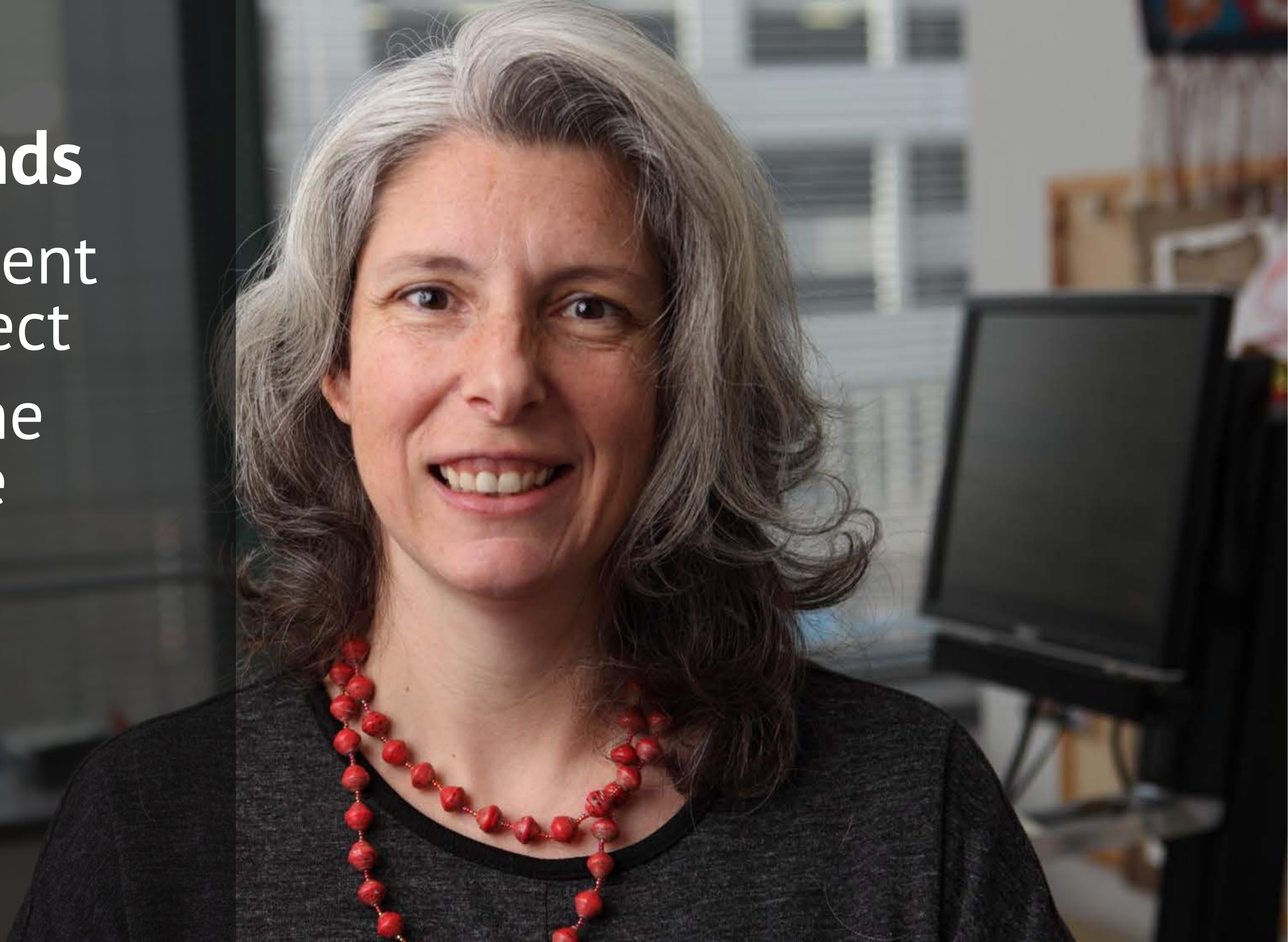

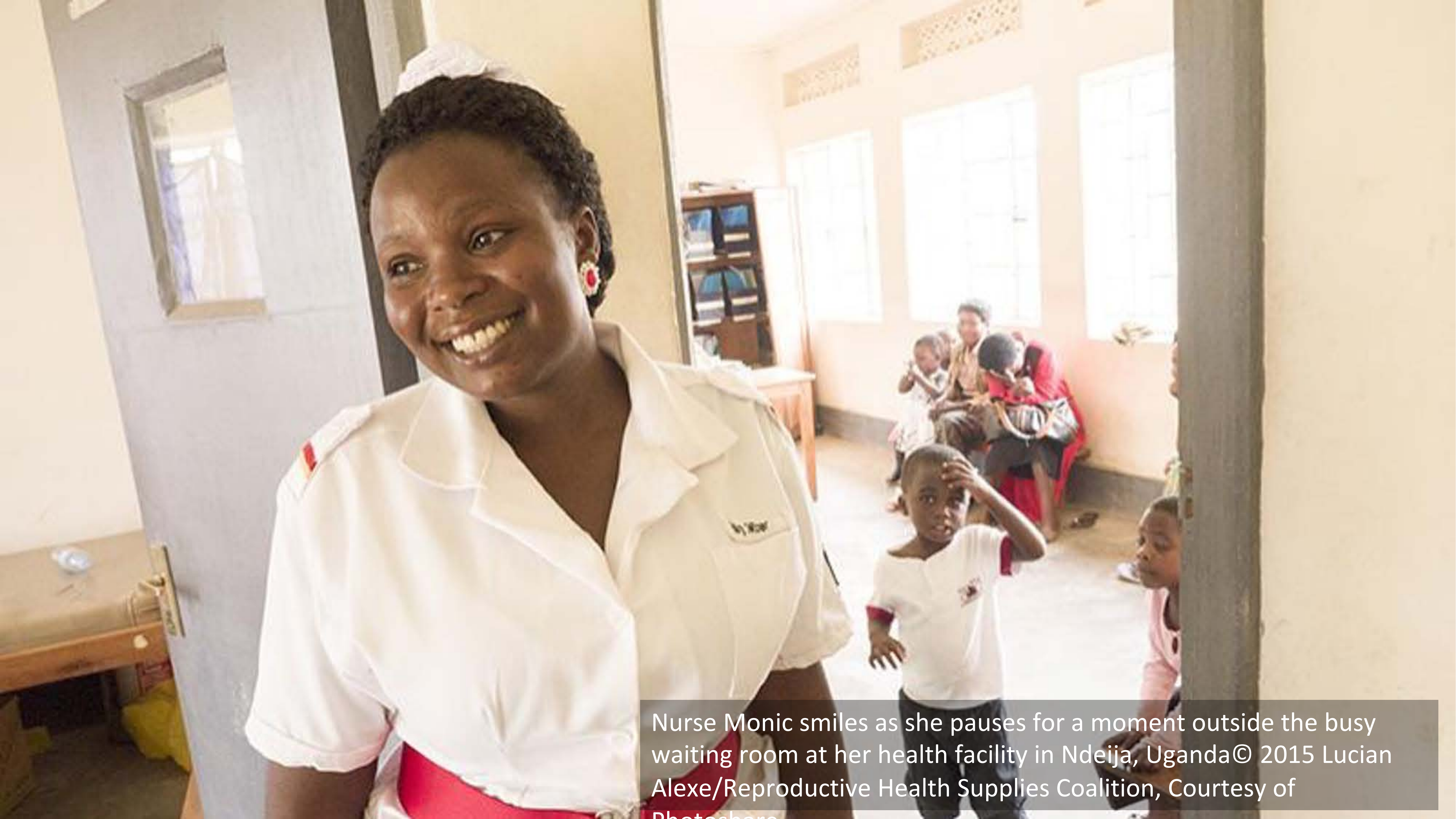

Nurse Monic smiles as she pauses for a moment outside the busy waiting room at her health facility in Ndeija, Uganda© 2015 Lucian Alexe/Reproductive Health Supplies Coalition, Courtesy of [illegible]

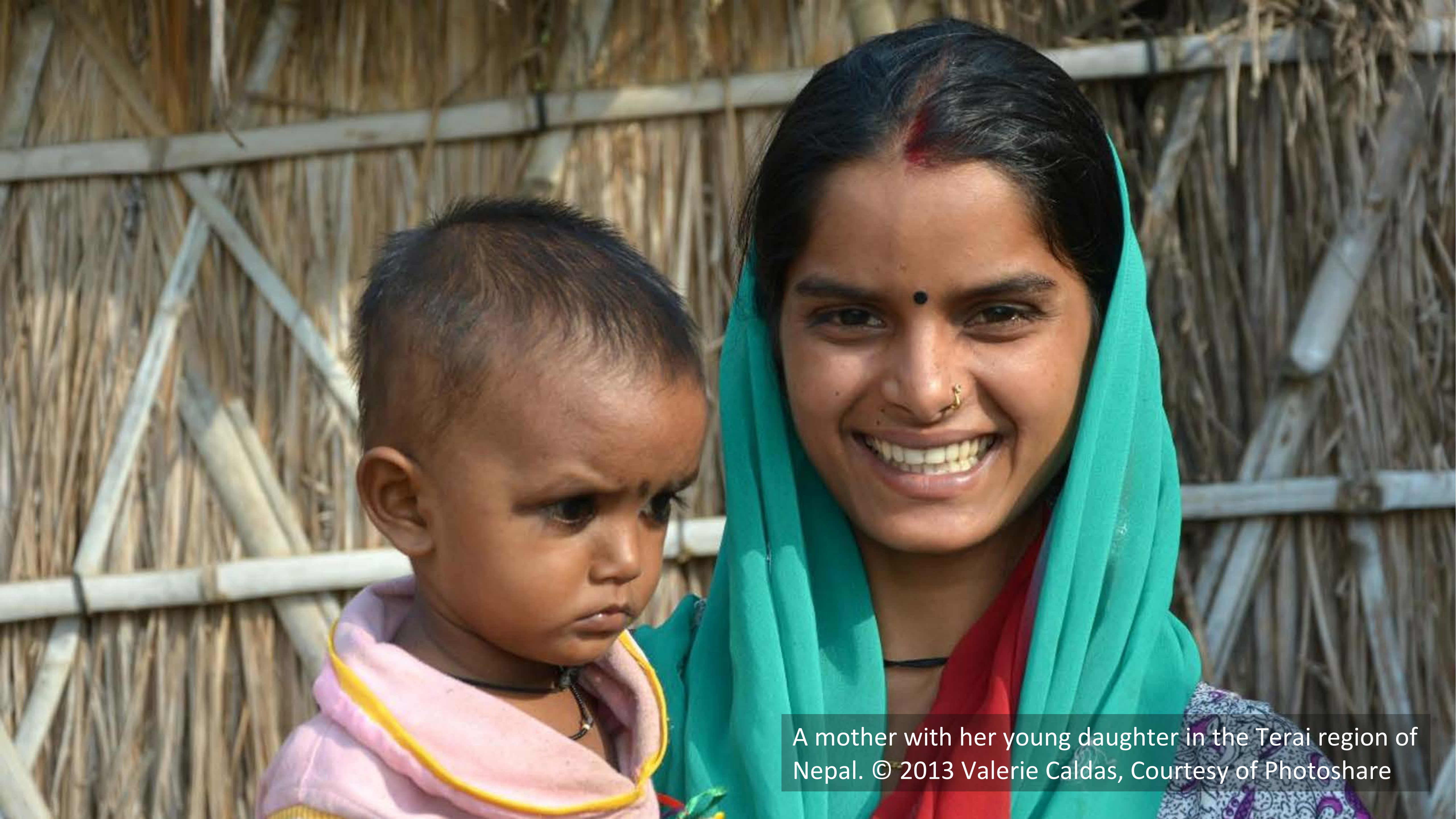

A mother with her young daughter in the Terai region of Nepal. © 2013 Valerie Caldas, Courtesy of Photoshare

Equipment

- cameras manual control – auto when needed
- always mobile
- lighting

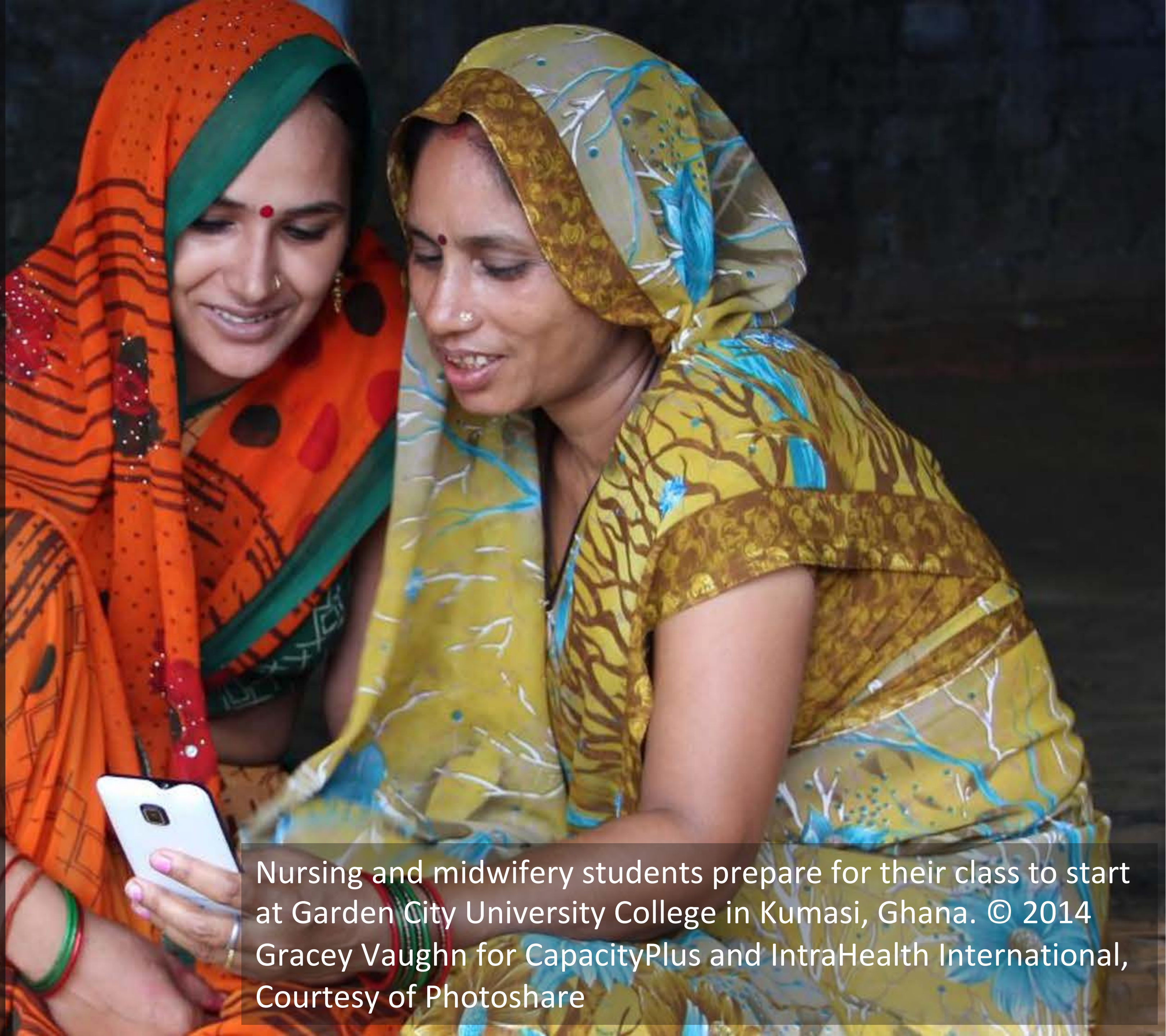

Nursing and midwifery students prepare for their class to start at Garden City University College in Kumasi, Ghana. © 2014 Gracey Vaughn for CapacityPlus and IntraHealth International, Courtesy of Photoshare

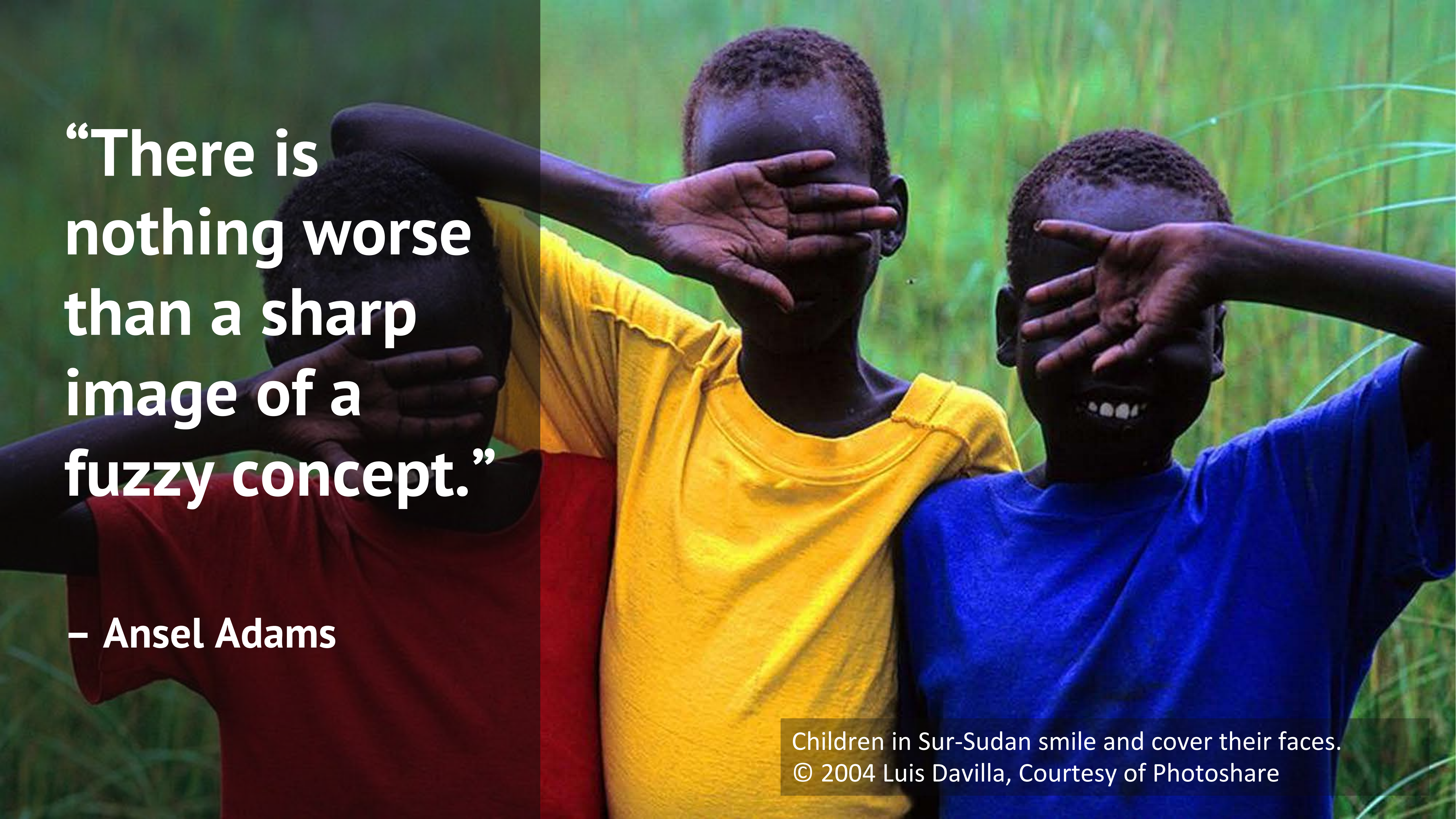

“There is nothing worse than a sharp image of a fuzzy concept.”

– Ansel Adams

Children in Sur-Sudan smile and cover their faces.
© 2004 Luis Davilla, Courtesy of Photoshare

Photography Ethics

Ethics

- fair use and
- informed consent

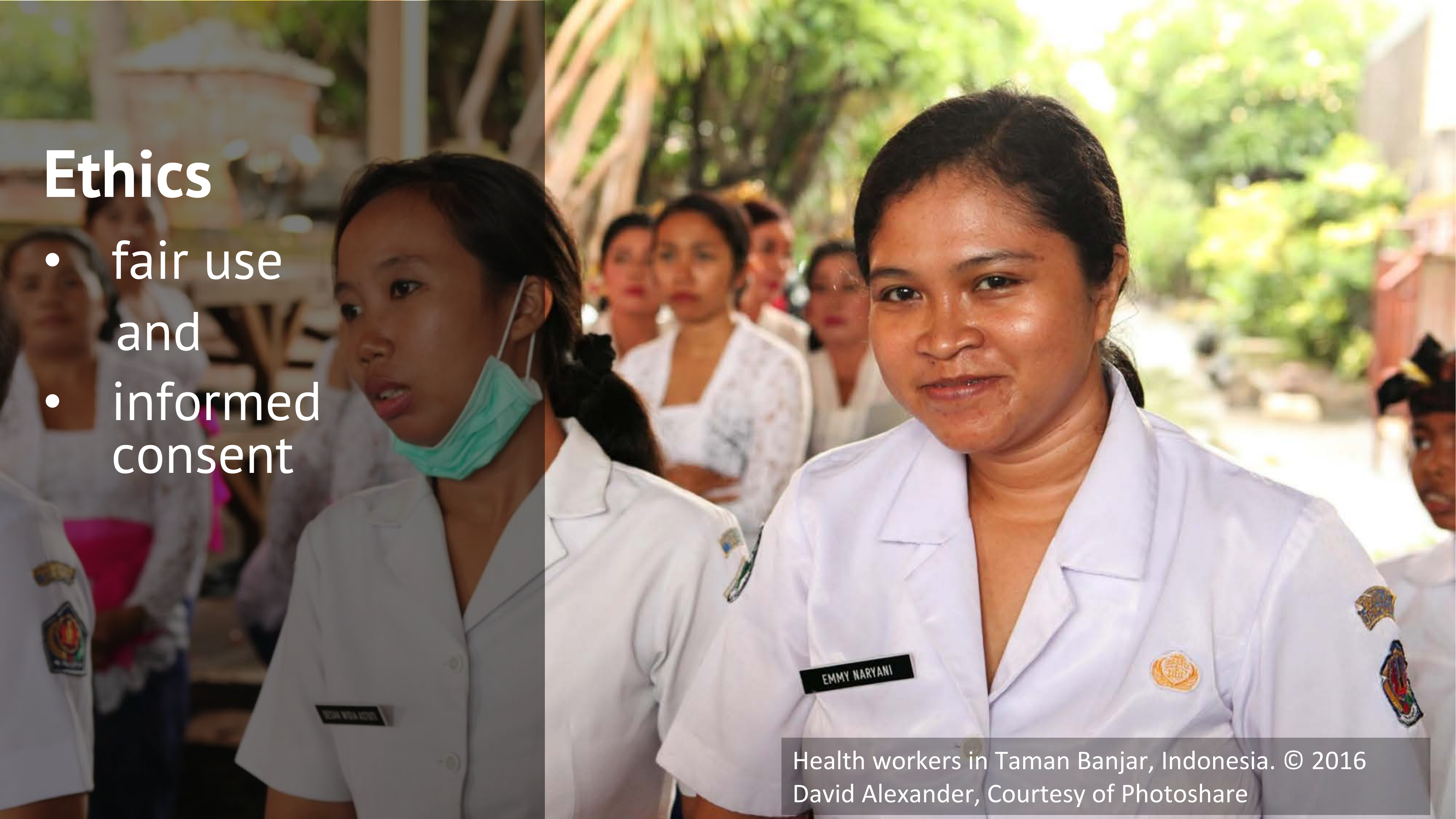

Health workers in Taman Banjar, Indonesia. © 2016 David Alexander, Courtesy of Photoshare

Editorial vs. Stock

- editorial: accurate representation of the real situation, subject identity, and physical location

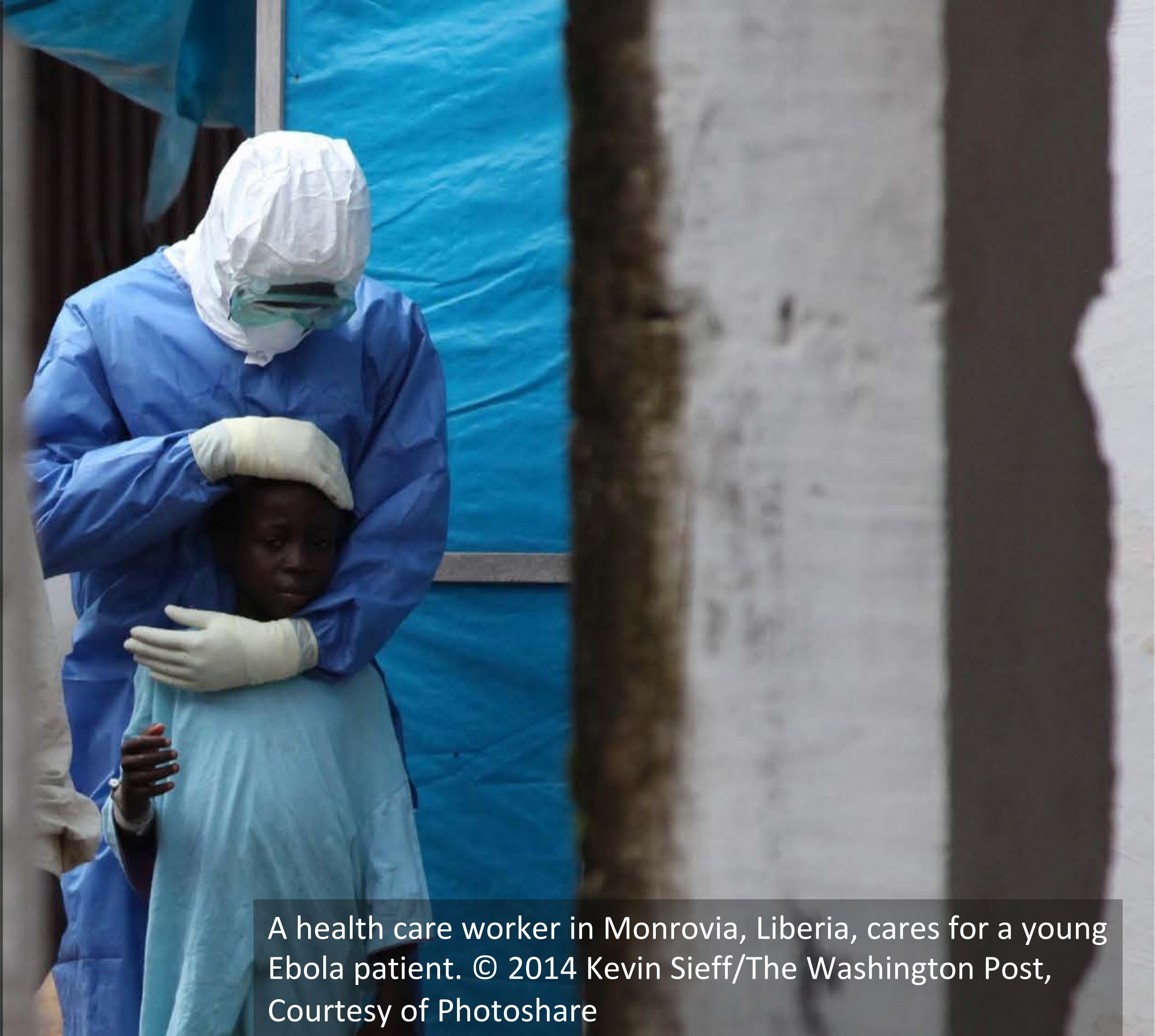

A health care worker in Monrovia, Liberia, cares for a young Ebola patient. © 2014 Kevin Sieff/The Washington Post, Courtesy of Photoshare

Informed Consent

- consent guidelines
- translators
- literacy
- protection of photo subjects

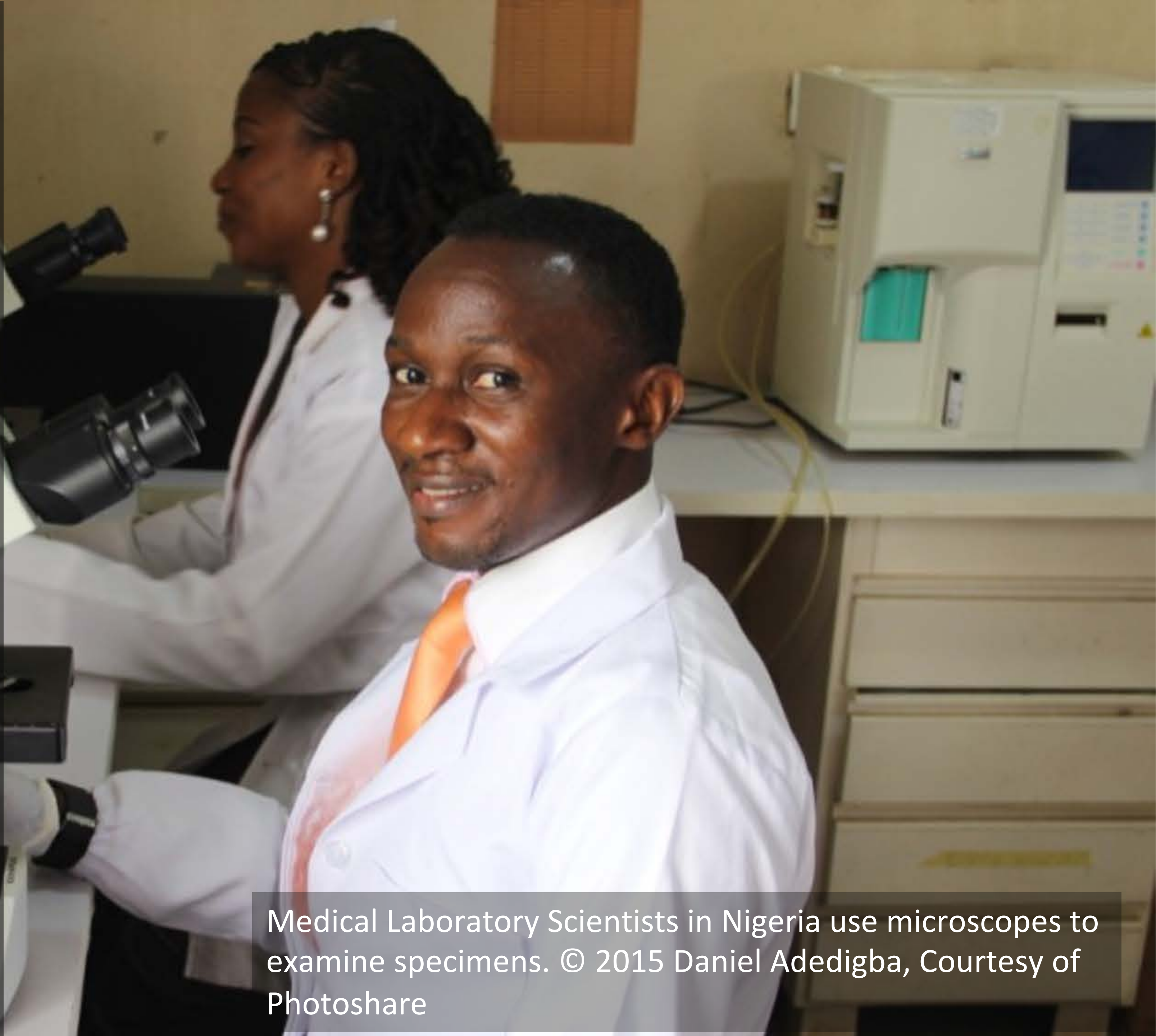

Medical Laboratory Scientists in Nigeria use microscopes to examine specimens. © 2015 Daniel Adedigba, Courtesy of Photoshare

Verbal vs. Written Consent

- editorial ethics
  - personal information
  - health status
  - behavior status
  - legal status
- children | parental consent

- *local laws and customs vary

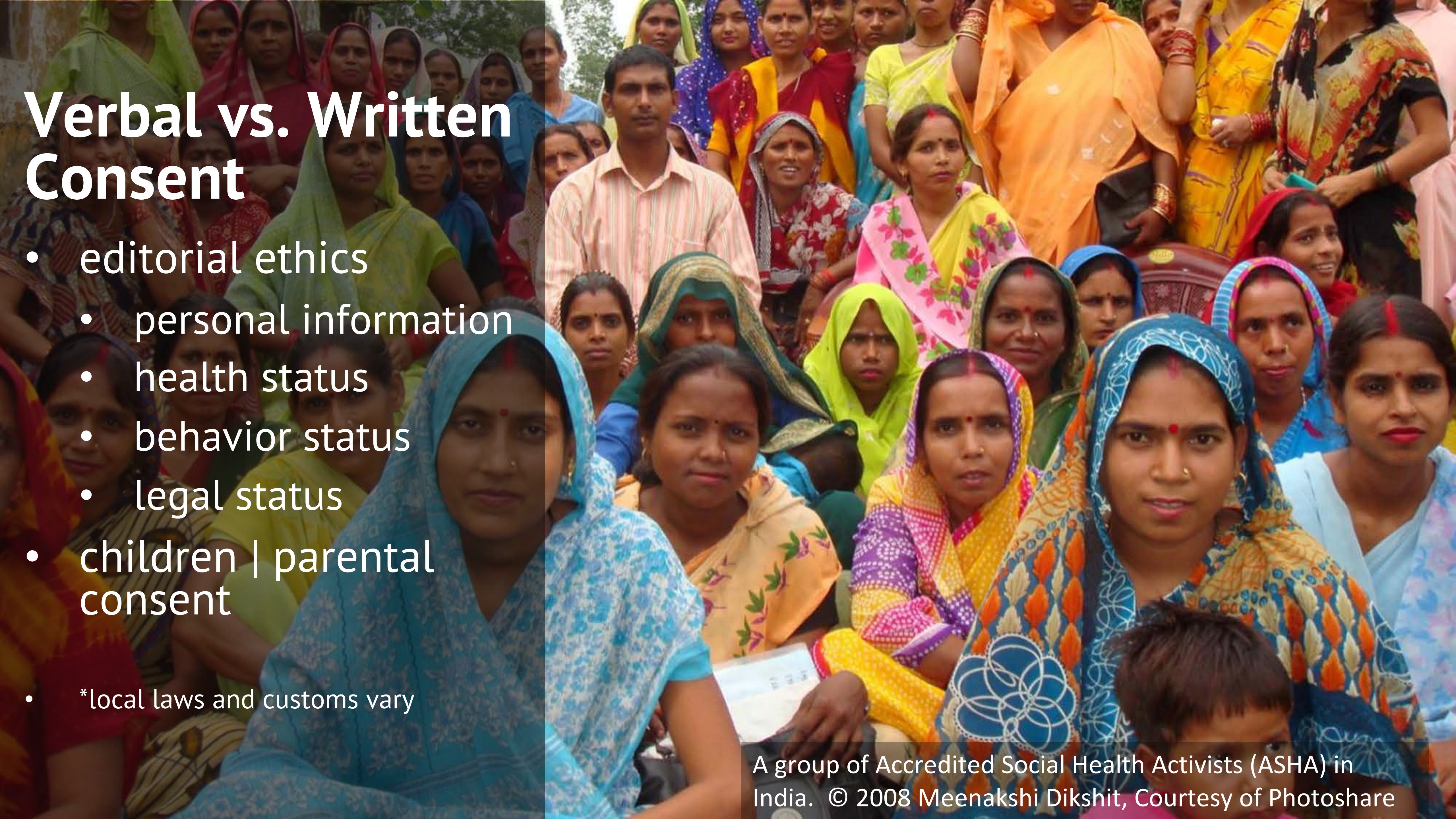

A group of Accredited Social Health Activists (ASHA) in India. © 2008 Meenakshi Dikshit, Courtesy of Photoshare

Consent not Needed	Obtain Verbal Consent	Written Consent Encouraged
Non-recognizable individuals in public (faces and all other identifying features are obscured).	All individuals in all settings **when possible**.	Recognizable providers and clients in **clinical settings**.
Public figures in public (e.g. celebrities, MOHs at campaign launches).	Parents, guardians, or teachers of children.	Recognizable or non-recognizable individuals in any setting where **personal, private information** is exposed in the photo or documented in the corresponding caption, such as:<br>• Health status (e.g. HIV-positive persons, persons living with AIDS/STIs, abortion history, TB, diarrheal disease, etc.)<br>• Health behavior (e.g. sex work, sexual orientation, alcohol and drug use, contraceptive use, female genital cutting, etc.)<br>• Criminal behavior (e.g. perpetrator or victim of gender-based violence, etc.)
Crowds in public (e.g. an audience at outdoor concert).	Directors/Managers of clinics or other service programs.	

- expectation of privacy

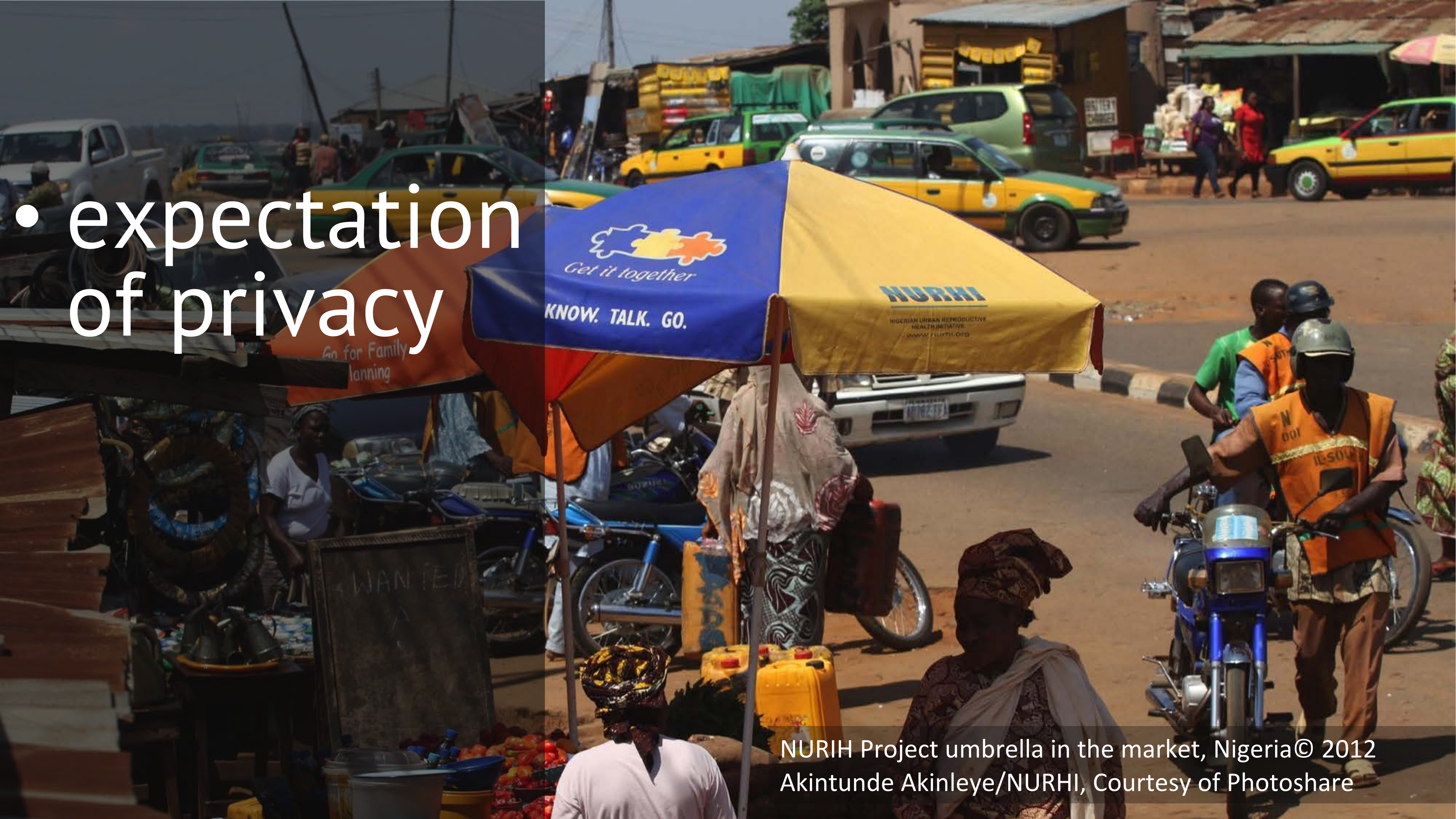

NURIH Project umbrella in the market, Nigeria© 2012 Akintunde Akinleye/NURHI, Courtesy of Photoshare

- expectation of privacy

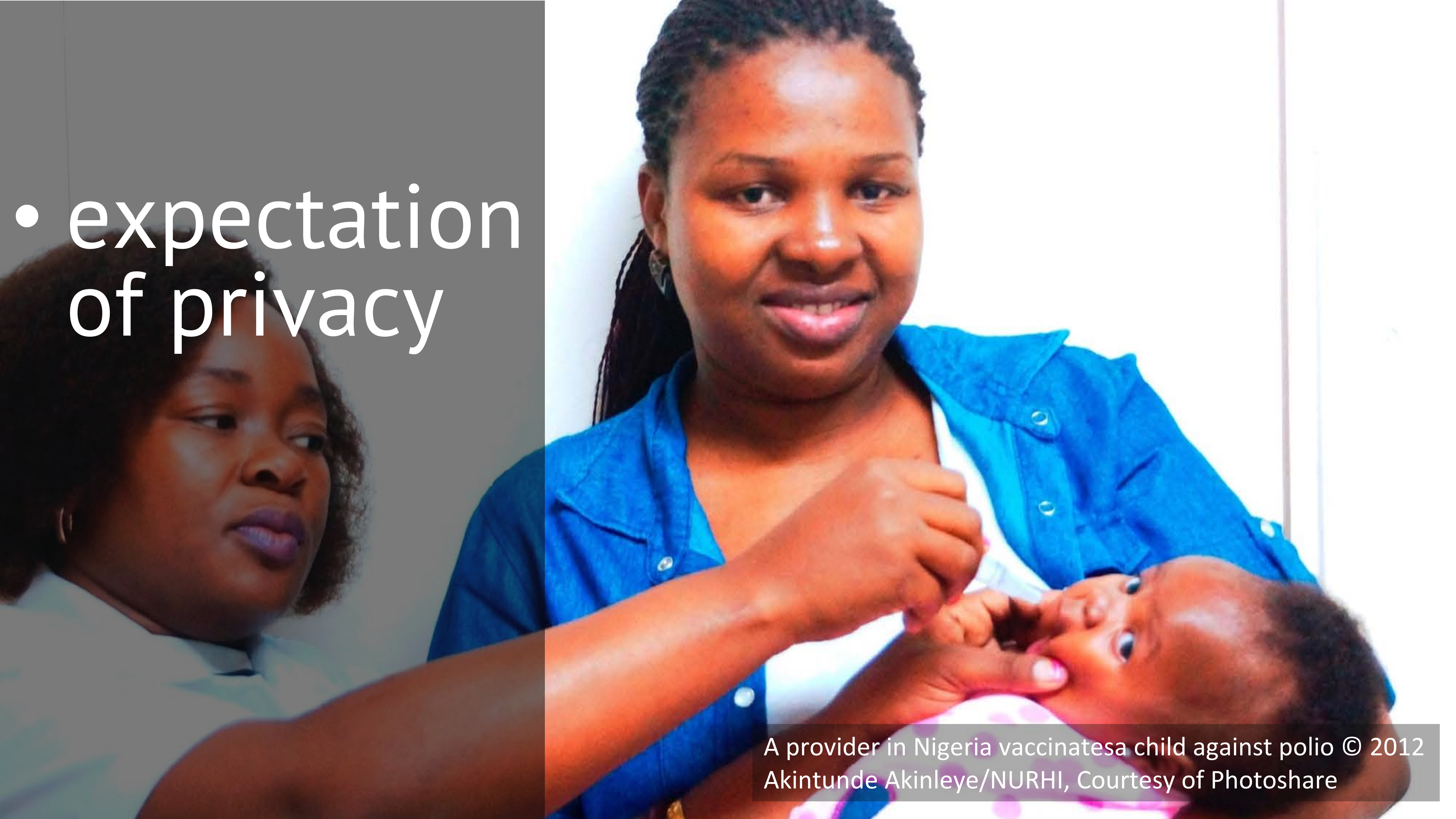

A provider in Nigeria vaccinatesa child against polio © 2012 Akintunde Akinleye/NURHI, Courtesy of Photoshare

Practice …

- Choose a partner
- Review the Appendix section of your story collection guide on Photography Tips!
- Practice taking photos of each other utilizing a digital camera or your smartphone
- Take a variety of photos (at least 10 unique photos) from different perspectives of your subject and consider various compositions and backgrounds

Share and Discuss

SHARE

- Which photo did you like the most which was taken by your partner and why?

DISCUSS

- What did you learn today that you will take with you into the field? About interviewing for MSC AND photography…
- What challenges do you think will arise and how will you overcome them?
- What is your biggest asset or strength that you think will help you in MSC collection?

Day 2

A few reminders for MSC stories

- Make sure to document the Background questions in your story
- Ensure opening and closing quotation marks so that the reader knows what is directly coming from the interviewee/storyteller
- Try to transcribe quotes related to the change AND why the change is significant in the interviewee/storyteller's opinion
- Spell out acronyms in brackets [ ] if they are used in the interview
- Don't forget photo captions (Caption: Brief description of photo including location. Credit: Photographer's name/ project/organization & date)
- Give the story a title

Today's agenda

- Develop an action plan to rollout implementation of MSC at the Hub level
  - Operationalizing Step 1
  - Review and Operationalization of Step 2: Selecting stories
  - Review and Operationalization of Steps 3 and 4: Feedback and Integrating MSC into Data Review Meetings
- Introduce Pause & Reflect
- Reflect on the training and identify follow-up plan

Recall the 4 Key MSC steps

1. **Collect stories** about significant changes
2. **Select the most significant** stories by stakeholders
3. **Feedback** the selected stories to all stakeholders
4. **Use** the information to improve programs

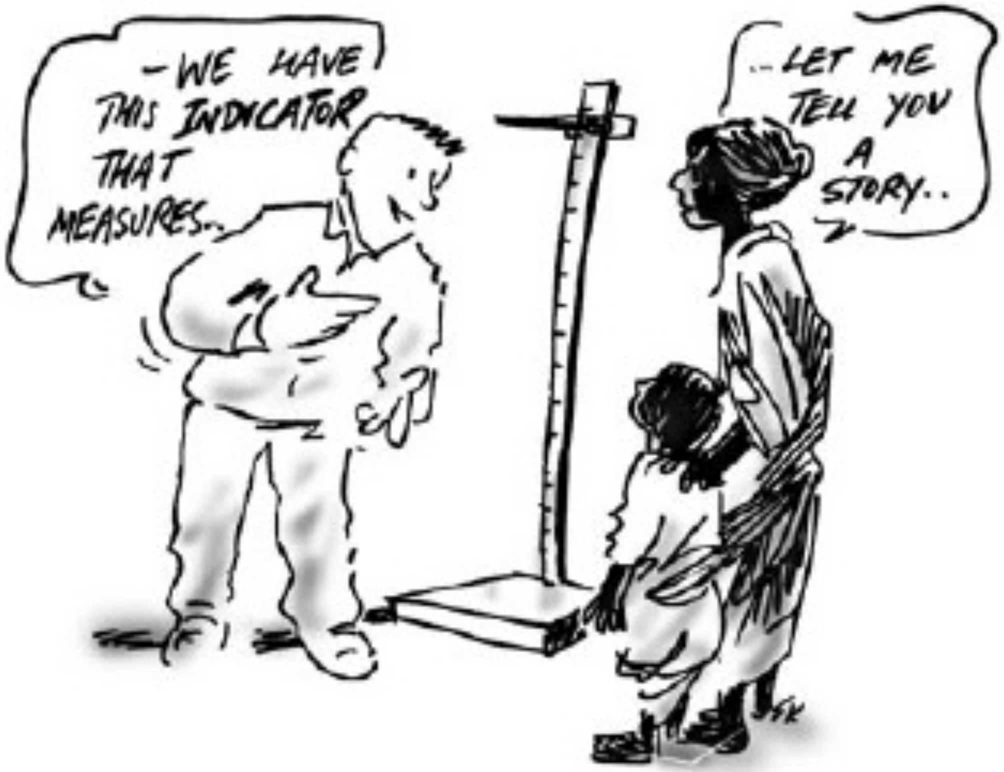

Image from *The Most Significant Change Technique: A Guide to Its Use*, by Rick Davies and Jess Dart.

Operationalizing Step 1

Possible TCI Stakeholders to Interview

Anyone directly involved in managing or implementing TCI proven interventions:

- State and LGA officials
- Ward officials
- Ministry of health and/or department
- FP/RH Coordinators
- Doctors, nurses, midwives
- Members of QITs
- Social mobilizers/CHEWs
- PPMVs
- Members of ACG/AWGs
- Members of Interfaith forum/groups
- Traditional leaders
- Members of SBCC Committees
- LPAY Ambassadors and those that interact with them (CSOs, AHDO, etc.)
- M&E/HMIS Officers

Group discussion

- Identify potential interviewees/storytellers and when it might make sense to interview them based on implementation plans

Frequency of story collection

At minimum, we recommend:

- Collecting MSC interviews at least quarterly
  - The number collected will depend on the status of program implementation and exposure to the high-impact interventions
- Capitalizing on already existing opportunities (coaching sessions, monthly meetings with implementation teams/ACGs, during D4D/data-related meetings, all staff meetings at Hub… others?) to collect these stories and also feed the selected stories back

Reviewing & Operationalizing Step 2

Step 2. Select the most significant stories

- Iterative selection process to reduce the large volume to more widely valued stories
- Happens at hub headquarter and global levels
- Opens door for discussion about what is most valued by the initiative
- Key output of MSC is not necessarily the selection of the MSC stories, it's about the dialogue and surfacing of values!
- Different ways to select stories

How do we reach a selection decision?

- **Majority Rules** (quick and easy, but miss out on discussion about the value)
- **Iterative voting** (can be time consuming but fosters good quality judgments)
- **Scoring** (best for remote meeting, but is more discriminatory and provides limited opportunity for dialogue)
- **Pre-scoring, then a group vote** (best when short for meeting time)
- **Secret ballot** (if it is helpful to keep anonymity, but still requires discussion!)

Test out different ways to see which works best for you!

Structure of selection team meeting

- Sit down together to review a pile of stories under each domain
- Read stories individually or out loud
- The group holds an in-depth conversation about which stories should be chosen
- The group decides which stories are felt to be most significant
- Reduce the pile to one story per domain
- Document the reasons for the group's choice(s)

What is "most" significant?

- Remember that this process is SUBJECTIVE! And NOT a calculation of "truth." We have to judge based on our perspective and interpretation.
- Refer to the domains of change and sustainability pillars. What are we seeking to understand in terms of changes?
- Document the justification for choice to each MSC story selected in a few sentences.

****A story is not invalid if it is not selected.**

Time to practice selecting stories!

- By table, review the stories collected from yesterday
- Decide how you will select the stories
  - Majority rules
  - Iterative voting
  - Scoring
  - Pre-scoring, then group vote
  - Secret ballot
- Individually or together, read all the stories collected
- Select the most significant story
- Document why you selected that story

Report out on selectin process

- Each group share most significant stories in each domain and reasons for selection
- What about the process?
  - Which selection methods did each group decide to use?
  - What worked well?
  - What did not work well? And how do you propose overcoming these challenges?
  - Would you use that method again?
- Do you have any questions?

Final steps of the MSC process

Step 3: Why feed back stories?

Feed back stories to storytellers and other project staff

- Can encourage people to participate in the MSC process in the future
- Shows storytellers that others have read and engaged with their stories
- Feedback about why a story was selected can expand or challenge participants' views of what is significant, influence where they place their attention in the future
- Encourages dialogue up and down the partnership

Feedback (and additional content outputs)

- Prompt feedback:
  - To those who provided the MSC stories and throughout the initiative
- Outputs:
  - Verbal (Can ask the storyteller of the select story to share their story)
  - Written (Can share a profile on the selected story – photo & quote)
  - Audio/visual (Can share the audio file or develop another output)
- Additional information products to share selected stories more broadly:
  - Webinars, study groups, rmini-universities
  - Newsletters
  - Video and photos from interviews to help tell the MSC stories back to the storytellers, across geographies, regions, and Hubs!

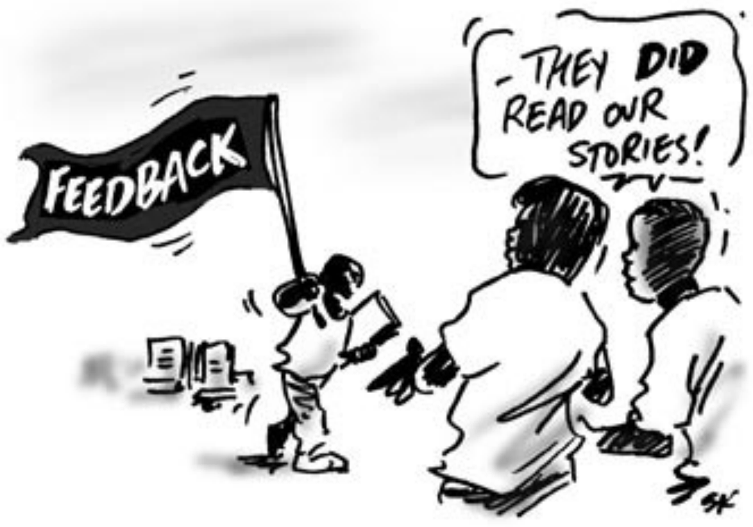

Image from *The Most Significant Change Technique: A Guide to Its Use*, by Rick Davies and Jess Dart.

Step 4: Integrating MSC into data review meetings

- Integrate MSC (qualitative data) into already established data review meetings
- Combine the quotes from the MSC stories with HMIS data
- Great opportunity for strong collaboration between the RM&E, Communication, and KM staff

Group discussion

- What platforms and meetings already occur with TCI stakeholders to review progress and data?
- How can MSC stories be incorporated into these platforms?

Resources

- TCI MSC Story Guide for Collecting MSC Stories from Implementers & Program Managers
- MSC Tracker (Excel file)
- TCI Guide for Collecting MSC Stories From Hub "Pause & Reflect" Sessions
- TCI MSC Action Plan
- MSC Technique User Guide, by Rick Davies and Jess Dart: http://mande.co.uk/wp-content/uploads/2018/01/MSCGuide.pdf
- Jess Dart – Most Significant Change Part 1-5 + Q&A: https://youtu.be/H32FTygl-Zs

Quarterly
Pause & Reflect Exercise

Pause. Reflect. Act

- TCI's **Pause & Reflect** (P&R) exercises are aimed at improving **how** we do our work (to streamline and 'right-size', identify promising practices, learn from mistakes and avoid future pitfalls, etc.) – that is, to help us manage adaptively

- Change is constant! As a result, governments and organizations leading health and development programs should "manage adaptively through continuous learning."

- Adaptive management is an **intentional**, **iterative** process for making decisions and adjustments by **learning from outcomes of the decisions** that were previously taken and changes that resulted from those decisions.

- Pause and reflect facilitates adaptive management

Benefits of Pause and Reflect (P&R)

- Ensure continuous questioning, introspection and progress towards better solutions/strategies
- Help TCI to stay focused on continuous improvement and meeting the cities' ever-changing needs in a dynamic urban context
- Promote openness and honesty in facing challenges head-on and generating alternative solutions without blame or judgement
- Enable identification and development of recommendations to use to update work plans and improve program implementation
- Ultimately lead to better interactions between the various levels of TCI operations – city, hub and global – and a strengthened TCI model

How Could Technical Teams Use P&R?

TCI Hub	Technical Teams
• The hub will have quarterly pause and reflect sessions to introspect, learn, decide and inform programming<br>• The hub will also document its lessons and disseminate them through articles and we	• Reflect on what works and doesn’t work in the thematic area<br>• Change strategies for reaching goals<br>• Document lessons and successes for external audiences<br>• Plan webinars to share successful strategies<br>• Can be incorporated into routine meetings

How is it Conducted?

- Focus Group Discussions (based on use of the MSC questionnaire)
- After Action Reviews (AAR)
- Data Reviews: Coming Up with Headlines
- Fail Fairs/Failure Panels
- Learning Exchange Visits/Study Tours
- Peer Assists

Difference Between MSC and P&R

MSC	P&R
**What is it**: Qualitative data source used to collect stories to document change in knowledge, attitudes/mindsets/ practice; political and financial commitments; health systems; and service access and quality over time as a result of TCI program	**What is it:** Quarterly meeting to reflect on what is working and not working to support programmatic decision-making and focus and inform adaptive management practices (doing certain things more, stopping others, etc.)
**Who's involved:** Hub trained staff collect stories from TCI stakeholders and then select which are most significant. Global team then makes its selection from those selected by hubs.	**Who's involved:** Program management discussions at hub headquarter and global levels.
**How used:** Stories used to document change to support quantitative data and inform data for decision-making.<br><br>Some stories adapted for communications products, blog posts, PASS bulletins, annual booklet, reports, etc.	**How used:** Notes documented and shared with Global so that common themes and learnings are identified across hubs and examples of adaptive management are collected.
**For more information:** https://tciurbanhealth.org/courses/program-design/lessons/most-significant-change/	**For more information:** https://tciurbanhealth.org/courses/program-design/lessons/pause-and-reflect/
